# Mendelian randomization combined with single-cell sequencing analysis revealed prognostic genes related to myeloid cell differentiation in prostate cancer and experimental verification

**DOI:** 10.3389/fimmu.2025.1619194

**Published:** 2025-09-23

**Authors:** Jianbai Chen, Jianxin Qiu, Wei Zhang, Zhiyong Nie, Xiaoping Gao, Gongquan Xu, Leiming Kang, Zhiming Zhang

**Affiliations:** Department of Urology, Tangdu Hospital, Fourth Military Medical University, Xi’an, China

**Keywords:** prostate cancer, myeloid cell differentiation, prognostic genes, Mendelian randomization, single-cell sequencing analysis, experimental verification

## Abstract

**Background:**

Myeloid cell differentiation (MCD) has an important correlation with prostate cancer (PCa), but the mechanism of action of the former in the latter is still under investigation. This study designed to investigate the prognostic genes related to MCD in PCa and the associated mechanisms.

**Methods:**

The related data were downloaded from public databases. Differentially expressed genes (DEGs) were intersected with MCD related genes (MCDRGs) to acquire candidate genes. Candidate prognostic genes with a causal relationship to PCa were further obtained through Mendelian randomization (MR). Prognostic genes were acquired by univariate Cox regression analysis and Least Absolute Shrinkage and Selection Operator (LASSO) analysis. Then, the risk model was built based on prognostic genes. Immune infiltration, nomogram model, and drug sensitivity were employed to investigate the roles of prognostic genes in PCa. The manifestation of prognostic genes in key cells was also investigated by single-cell sequencing (scRNA-seq) analysis. Finally, the manifestation of prognostic genes were authenticated by *in vitro* experiments.

**Results:**

The 23 candidate prognostic genes had a causal relationship with PCa. The 5 prognostic genes (NR3C1, BMP2, RACGAP1, TLR3, FASN) were identified. The risk models suggested that high risk group (HRG)’s survival rate was inferior to that of low risk group (LRG). The nomogram indicated that prognostic genes could effectively predict the survival status of PCa patients. There were 18 immune cells that suggested notable differences between the HRG and the LRG. The HRG and LRG suggested notable differences in sensitivity to 86 drugs such as AZD8186. Epithelial cells were considered as key cells. Only FASN was consistently active during critical cell differentiation. The *in vitro* results were consistent with the results of bioinformatics analysis, indicating that the analysis results were reliable.

**Conclusion:**

This study identified 5 prognostic genes and a risk model, suggesting a fresh thought on the subsequent development of PCa related drugs.

## 1 Introduction

Prostate cancer (PCa) represents one of the most prevalent malignancies in the male genitourinary system, globally ranking as the second most frequently diagnosed cancer among males (1, 2). Its pathogenesis is multifactorial, involving age, genetic predisposition, racial disparities, and dysregulated gene expression, with approximately 20% of cases exhibiting aberrations in DNA repair pathways (1, 3). Although localized PCa in early stages can be effectively managed through radical prostatectomy or radiotherapy, biochemical recurrence occurs in 20-40% of treated patients (1, 3). Notably, individuals progressing to metastatic castration-resistant PCa (mCRPC) demonstrate a 5-year survival rate below 30%, underscoring the critical need for advanced therapeutic strategies (2, 3).

Current clinical management strategies encompass androgen deprivation therapy (ADT), novel androgen receptor pathway inhibitors, chemotherapy, and immune checkpoint inhibitors (ICIs) (3–5). However, these therapeutic approaches demonstrate suboptimal efficacy against metastatic lesions and biochemical recurrence, coupled with a substantial toxicity burden that often limits their clinical utility (3–5).

In-depth investigation of the molecular mechanisms underlying PCa represents a critical avenue to overcome current therapeutic limitations. scRNA-seq technologies have unveiled substantial heterogeneity within cancer-associated fibroblasts (CAFs) in the tumor microenvironment, which promote oncogenic niche formation through secretion of specific cytokines, a biological feature positively correlated with tumor progression (6, 7). Genomic studies have identified key driver events including PTEN deletion and BRCA1/2 mutations that induce homologous recombination repair (HRR) pathway dysfunction (3, 8, 9). PARP inhibitors developed based on these molecular characteristics demonstrate significant clinical benefits in BRCA-mutant patients, extending median radiographic progression-free survival (rPFS) to 7.4 months (3). Prognostic models systematically integrating 10 CAFs core regulatory genes such as THBS1 and LDHA exhibit robust discriminative power for stratifying patient survival outcomes through risk scoring (3). These findings not only elucidate the evolutionary biology of PCa but also provide critical theoretical foundations for developing targeted therapies and advancing personalized medicine. Therefore, identification of novel prognostic biomarkers and establishment of precision prediction frameworks will be pivotal to resolving therapeutic challenges in advanced PCa.

Myeloid cells, as central orchestrators of the tumor immune regulatory network, critically determine immune evasion, tumor progression, and clinical outcomes through their differentiation states and functional plasticity (10, 11). Accumulating evidence demonstrates that myeloid-derived suppressor cells (MDSCs) in solid tumor microenvironments drive oncogenesis through dual mechanisms, direct suppression of CD8+ T cell antitumor activity via effector molecules including arginase-1 (ARG1), inducible nitric oxide synthase (iNOS), and reactive oxygen species (ROS), and facilitation of metastatic dissemination through pro-angiogenic mediators such as VEGF (10, 12). Notably, LOX-1 surface expression on MDSCs exhibits significant inverse correlations with circulating tumor DNA burden and overall survival rates (10, 12). Mechanistically, tumor-derived oxidized lipids potentiate myeloid immunosuppressive capacity by activating the STAT3 signaling axis and CSF1R pathway, thereby inducing metabolic reprogramming that sustains immune tolerance (11, 12).

Previous study has indicated that PCa cells elicit functional reprogramming of myeloid lineages (including THP-1 and HL-60) through stress protein secretion, particularly heat shock protein 27 (Hsp27), manifesting as surface marker polarization and VEGF secretion dysregulation, thereby modulating tumor-immune crosstalk (13). Notably, therapeutic interventions targeting myeloid surface receptors like the CD47-SIRPα axis enhance macrophage-mediated tumor phagocytosis compared to controls and improve anti-tumor immune responses in preclinical studies (11). However, the precise molecular targets governing myeloid cell differentiation (MCD) in PCa progression remain poorly characterized, particularly key signaling pathways such as JAK/STAT or NF-κB signaling pathways and immunoregulatory cytokine networks (14). Moreover, the bidirectional regulatory network between neoplastic cells and myeloid populations involving cytokine crosstalk and surface receptor interactions requires comprehensive investigation to delineate their co-evolution mechanisms.

Mendelian randomization (MR) leverages the random assortment of genetic variants during gametogenesis to emulate randomized controlled trials, effectively circumventing confounding biases and reverse causation inherent in conventional observational studies (15, 16). ScRNA-seq offers high-throughput resolution of whole transcriptomes at individual cell resolution, enabling precise mapping of immune cell heterogeneity and functionally distinct subsets within tumor microenvironments. Pioneering studies have employed scRNA-seq to construct pan-cancer endothelial cell atlases, uncovering tumor-specific endothelial subpopulations orchestrating pro-angiogenic and immunosuppressive programs (5, 17, 18). In PCa pathobiology investigations, Miao et al. integrated scRNA-seq with bulk transcriptomics to identify MXRA8-mediated tumor progression through dysregulating oxidative stress pathways in prostate tumor niches (16), while Ye et al. established MR-based causal relationships between genetically proxied CD25+ naïve B cell abundance and PCa risk (19). Nevertheless, the synergistic application of MR framework with scRNA-seq technologies to decipher MCD-PCa crosstalk mechanisms remains substantially underexplored, representing a critical knowledge gap in the field.

In this study, we employed integrated multi-omics analyses (incorporating bulk transcriptomic and scRNA-seq data) to identify prognostically significant genes causally linked to MCD through MR framework, ultimately constructing a clinical-grade prognostic signature. Bioinformatics interrogation systematically delineated the molecular regulatory circuitry underlying these candidate genes in PCa progression. Furthermore, single-cell resolution analysis uncovered their expression dynamics within disease-associated cell subpopulations, while reverse transcription-quantitative polymerase chain reaction (RT-qPCR) experiments provided preliminary validation of their potential roles in tumorigenesis. To further validate the functional roles of candidate genes in PCa progression, we performed complementary *in vitro* assays: Western blot analysis to examine protein expression, Ki67 immunofluorescence staining to assess cell proliferation, and scratch-wound assays to evaluate cell migratory capacity, aiming to verify their regulatory effects on PCa cell malignant behaviors. Our findings provide multi-dimensional evidence elucidating MCD-associated molecular networks in PCa, establishing both conceptual and experimental foundations for developing personalized therapeutic strategies and targeted drug discovery.

## 2 Method

### 2.1 Data collection

The transcriptome data (TCGA-PRAD) on gene expression matrix and relapse information of PCa were extracted from The Cancer Genome Atlas (TCGA) database (https://tcga-data.nci.nih.gov/tcga/). The TCGA-PRAD dataset (access time: November 20th, 2024) included 502 PCa tissue samples (397 samples with relapse information) and 52 paracancer (control) tissue samples. Among the 397 samples, 70% samples (278 samples) were employed as the training set (TCGA-PRAD-train), and 30% samples (119 samples) were employed as the validation set (TCGA-PRAD-validation). The single-cell RNA sequencing (scRNA-seq) data (GSE141445) of PCa was scoured from Gene Expression Omnibus (GEO) database (https://www.ncbi.nlm.nih.gov/geo/). The GSE141445 (GPL24676, access time: November 20th, 2024) included 13 PCa tissue samples. Validation dataset GSE116918 contained 248 samples with complete recurrence information. The MR data (EBI-A-GCST90018905) of PCa and eQTL data were acquired from the Integrative Epidemiology Unit (IEU) Open Genome-wide Association Study (GWAS) database (https://gwas.mrcieu.ac.uk/). The “EBI-A-GCST90018905” included 24,119,306 SNPs from 211,227 Europeans (case: control=11,599: 199,628). The 423 MCD-related genes (MCDRGs) used in the study were obtained by merging and removing duplicates from three myeloid cell differentiation-related datasets: GOBP_NEGATIVE_REGULATION_OF_MYELOID_CELL_DIFFERENTIATION (95 genes), GOBP_POSITIVE_REGULATION_OF_MYELOID_CELL_DIFFERENTIATION (104 genes), and GOBP_MYELOID_CELL_DIFFERENTIATION (423 genes), which were downloaded from the MCD related genes (MCDRGs) were acquired from Molecular Signatures Database (MSigDB) (https://www.gsea-msigdb.org/gsea/msigdb/human/search.jsp) and relevant references (20) (**Supplementary Table 1**).

### 2.2 Differential expression analysis

To acquire differentially expressed genes (DEGs) between PCa and control samples in TCGA-PRAD, “DESeq2” package (v 3.4.1) was carried out (PCa *vs* control) (|log_2_Fold Change (FC)| > 0.5, *P* < 0.05) (21). On the basis of the log_2_FC value, DEGs were visualized and the top 10 up/down-regulated gene names were labeled by the volcano plot utilizing “ggplot2” package (v 3.4.1) (22). Similarly, the expressions of the top 10 up/down-regulated genes between PCa and control groups were displayed by the heat plot utilizing “ComplexHeatmap” package (v 2.14.0) (23).

### 2.3 Identification and functions of candidate genes

DEGs were intersected with MCDRGs to acquire candidate genes via “ggvenn” package (v 1.7.3) (24). To probe the organic activities and signal pathways involved in candidate genes, based on background gene set of “org.Hs.eg.db” package (v 3.16.0) (25), Gene Ontology (GO) (*P* < 0.05) and Kyoto Encyclopedia of Genes and Genomes (KEGG) (*P* < 0.05) enrichment analyses were carried out utilizing “clusterProfiler” package (v 4.7.1.3) (26). Subsequently, to determine the interactions of proteins encoded by candidate genes, candidate genes were uploaded to STRING database (https://cn.string-db.org/) (interaction score > 0.4). The results were imported into Cytoscape software (v 3.9.1) and Protein-Protein Interaction (PPI) network was constructed (27).

### 2.4 MR analysis

To probe the causal dependence between candidate genes and PCa and acquire candidate prognostic genes, MR analysis was performed with PCa as outcome event and candidate genes as exposure factors utilizing “TwoSampleMR” package (v 0.6.1) (28). The MR consisted of 3 assumptions: (1) instrumental variables (IVs) were linked with exposure factors; (2) IVs could only influence outcomes by exposure factors; (3) IVs were not linked with potential confounders. To obtain effective IVs, exposure factors and outcome event were read and IVs were screened via “extract_instruments” function. The screening criteria were as follows: (1) IVs strikingly linked with exposure factors (*P* < 5×10^−6^); (2) IVs that exhibited linkage disequilibrium were removed with R^2^ = 0.001, kb=10, and clump=TRUE; (3) IVs that were strikingly linked with outcome were removed with rsq=0.8 and proxies=TRUE; (4) IVs whose F statistic < 10 were removed ( $\text{F}=\frac{\left(\text{N}-\text{K}-1\right)}{\text{K}}/\frac{{\text{R}}^{2}}{1-{\text{R}}^{2}}$ , R^2^ represented the cumulative explanatory variance of SNPs, N represented the number of the samples, and K represented the number of SNPs). Then, the effect alleles and effect sizes were unified via “harmonCIe_data” function. The 5 algorithms of the “mr” function were utilized to conduct MR analysis for each exposure factor and outcome, which included Inverse Variance Weighted (IVW) (29), Weighted Mode (30), MR Egger (31), Simple Mode (28), and Weighted Median (32). MR analysis mainly relied on IVW results. Exposure factors with SNP > 2 and *P* < 0.05 were considered as the exposure factors that had a causal relationship with PCa. The odds ratio (OR) > 1 suggested that the exposure factor was a contributing factor to the risk of PCa, while an OR < 1 indicated that it was a protective factor. Notably, the scatter plot was drawn to further identify the correlation between exposure factors and outcome in combination with SNP-exposure effects and SNP-outcome effects via “mr_scatter_plot” function. The forest plot was drawn to evaluate the diagnostic power of the estimated exposure factors of each SNP site on the outcome via “mr_forest_plot” function. The funnel plot was drawn to judge whether the analysis was random and adhered to Mendel’s second law random grouping in combination with the β and standard error (SE) of each IV via “mr_funnel_plot” function.

Moreover, to evaluate the reliability of MR analysis results, sensitivity analysis was applied. Heterogeneity test was performed via “mr_heterogeneity” function (*P >* 0.05, Cochran’s Q test). Horizontal pleiotropy test was performed to find out whether confounding factors existed via “mr_pleiotropy_test” and “MR-Egger” functions (*P* > 0.05). To observe whether the SNPs of each IV caused considerable changes in the outcome, Leave-one-out (LOO) analysis was performed via “mr_leaveoneout” function.

To verify that the results of the forward analysis were not interfered by reverse causality and determine the validity of the causal sequence between the outcome and the exposure factors, Steiger test was carried out via “steiger_filtering” function (Steiger-dir=TRUE, *P* < 0.05).

At last, the candidate genes examined by sensitivity analysis and Steiger test were specified as candidate prognostic genes.

### 2.5 Identification of prognostic genes and construction of prognostic models

To identify prognostic genes, in TCGA-PRAD-train, univariate Cox regression analysis was performed on the basis of candidate prognostic genes via “survival” package (v 3.5-3) (33)(*P* < 0.2) (34, 35) and the result was presented via “forestplot” package (v 2.0.1) (36). Genes with consistent hazard ratios (HRs) or ORs in both univariate Cox regression analysis and MR analysis were utilized for proportional hazards (PH) assumption test via “cox.zph” function (*P* > 0.05). After that, Least Absolute Shrinkage and Selection Operator (LASSO) was implemented utilizing “glmnet” package (v 4.1.4) (37)(10-fold cross-validation). Finally, genes that had passed through the above analyses in sequence were defined as prognostic genes to construct a risk model.

In TCGA-PRAD-train, on the basis of the expression of prognostic genes and the risk coefficients gained from LASSO regression, PCa patients were scored with the following formula.


$$\text{risk score}=\overset{\text{n}}{\underset{\text{i}=1}{\sum }}\text{coef }({\text{gene}}_{\text{i}})\times \text{expr }({\text{gene}}_{\text{i}})$$

Expr represented the expression level of each prognostic gene and coef signified the risk coefficient of each prognostic gene. Notably, according to the median of risk score, PCa patients were categorized into high risk group (HRG) and low risk group (LRG) and risk score distribution and survival status of patients were displayed. According to relapse time and relapse status of PCa patients, the “survminer” package (v 0.4.9) (38) was implemented to draw and compare survival curves of HRG and LRG (*P* < 0.05). The Area Under Curve (AUC) values of receiver operating characteristic (ROC) curves at 1, 2, and 3 years were employed to evaluate the accuracy of risk model utilizing “survivalROC” package (v 1.18.0) (39) (AUC > 0.6). The validation was performed in the GSE116918 dataset. Additionally, the heat plot was drawn to display the expression levels of prognostic genes in HRG and LRG. Using the same method, 2 risk models were constructed in TCGA-PRAD-validation and all samples with relapse information of TCGA-PRAD to validate the accuracy of the above-mentioned model.

### 2.6 Construction of nomogram model

To evaluate the ability of prognostic genes to predict PCa recurrence rates, the “regplot” package (v 1.1) (40) was employed to build the nomogram model for PCa in the TCGA-PRAD-train. Each prognostic gene was scored separately, and the scores were added together to obtain the total scores. The higher the total scores, the higher the recurrence rate of the patient. Calibration curves built via “rms” package (v 6.5.0) (41) and ROC curves built via “survivalROC” package (v 1.18.0) (AUC > 0.6) were applied to compute the effectiveness of the nomogram model in clinical prediction at 1, 2, and 3 years for PCa.

### 2.7 Immune infiltration analysis

To estimate the tumor purity and the status of stromal and immune cells in the malignant tumor tissues within the tumor microenvironment of PCa patients. The data were normalized using the R package estimate (v 1.0.13, https://R-Forge.R-project.org/projects/estimate/), and the stromal score, immune score, and ESTIMATE score were calculated using the ESTIMATE algorithm. Wilcoxon test was employed to contrast the scores mentioned above between HRG and LRG (*P* < 0.05). For further evaluation of the situation of immune cells in the development process of PCa, based on single sample Gene Set Enrichment Analysis (ssGSEA) algorithm, in the TCGA-PRAD-train, “GSVA” package (v 1.46.0) (42) was applied to compute the enrichment scores of 28 immune cells (43) in the HRG and LRG. Wilcoxon test was implemented to contrast differences in immune cell infiltration between HRG and LRG (*P* < 0.05). What’s more, to understand the link between prognostic genes and differential immune cells, in the TCGA-PRAD-train, “psych” package (v 2.2.9) (44) was performed (|correlation (cor)| > 0.3, *P* < 0.05).

### 2.8 Pathways and GeneMANIA analysis

To determine the biological pathways involved in the occurrence and development of PCa in the HRG and LRG, in the TCGA-PRAD-train, “DESeq2” package (v 3.4.1) was employed to perform differential expression analysis between HRG and LRG and log_2_FC values were calculated. The log_2_FC values were sorted in descending order. Then, Gene Set Enrichment Analysis (GSEA) was performed via “clusterProfiler” package (v 4.7.1.3) (|Normalized Enrichment Score (NES)| > 1, *P* < 0.05). The top 5 pathways with notable P-values were presented. Besides, GeneMANIA database (http://genemania.org) was applied to predict the genes related to the functions of prognostic genes and their involved functions.

### 2.9 Drug sensitivity analysis

To probe the drug sensitivity of the HRG and LRG, drugs related to tumors were obtained from Genomics of Drug Sensitivity in Cancer 2 (GDSC2) database (https://www.cancerrxgene.org/). In the TCGA-PRAD-train, “pRRophetic” package (v 0.5) (45) was employed to determine the half maximal inhibitory concentration (IC50) of each tumor sample. Wilcoxon test was employed to contrast the differences in drug sensitivity between the HRG and LRG (*P* < 0.05). The result was displayed via “ggplot2” package (v 3.4.1).

### 2.10 Construction of molecular regulatory network

To explore the upstream regulatory factors of prognostic genes and their interaction relationships, based on NetworkAnalyst online website (https://www.networkanalyst.ca/NetworkAnalyst/uploads/ListUploadView.xhtml), Transcription Factors (TFs) were predicted by TRRUST database (https://www.grnpedia.org/trrust/) and miRNAs were predicted by miRTarBase database (v 9.0) (https://mirtarbase.cuhk.edu.cn/) and TarBase (v 9.0) (https://dianalab.e-ce.uth.gr/tarbasev9). Finally, the TF-mRNA-miRNA network was constructed utilizing Cytoscape software (v 3.9.1).

### 2.11 ScRNA-seq analysis

All scRNA-seq analyses were performed via “Seurat” package (v 5.0.1) (46). To ensure the accuracy and reliability of scRNA-seq data, in the GSE141445, the data was filtered via “PercentageFeatureSet” function. The screening criteria were as follows: (1) the number of genes in the cells ranged from 200 to 3,000; (2) genes with an expression level ranging from 200 to 4,531 and covered by at least 3 cells were retained. Then, the data after filtering were normalized via “NormalizeData” function. The 2,000 highly variable genes (HVGs) were acquired and the top 10 most mutated genes were labeled via “FindVariableFeatures” function and the result was presented via “LablePoints” function. Moreover, the samples of GSE141445 were subjected to normalization processing via “Scale Data” function. The HVGs were subjected to principal component analysis (PCA) via “runPCA” function. The P-value when the principal components (PCs) ranged from 1 to 30 was calculated via “Jackstraw” function. The values of the sudden variance drops when different values were taken for the PCs were calculated and the result was displayed via “Elbowplot” function. When *P* < 0.05, the PCs at the inflection point in the variance elbow plot were used for subsequent analysis. Additionally, unsupervised clustering analysis was applied via “FindClusters” and “FindNeighbors” functions (resolution=0.1). Notably, cells were clustered and result was visualized via “RunTSNE” function. Furthermore, to determine the cell types of the cell clusters, cell clusters were commented on the basis of marker genes (47). The expression patterns of marker genes in different cell clusters and cells were displayed through bubble plots.

### 2.12 Identification of key cells, cell communication, and pseudo-time analysis

To gain key cells associated with PCa development, in the GSE141445, the manifestation of prognostic genes in cells were displayed through bubble plots. The cells with the highest gene expression were labeled as key cells. Besides, the distribution of prognostic genes in cells was also presented.

In the GSE141445, the “CellChat” package (v 1.6.1) (48) was employed to explore the communication networks among different cells. The pairing of ligands and receptors among cells was presented through bubble diagrams using “ggplot2” (v 3.4.1). Furthermore, to investigate the differentiation of key cells, key cells were subjected to secondary clustering using the same method as that in 2.11. Then, the developmental trajectories of key cells were analyzed via the “Monocle” package (v 2.22.0) (49). The “DDRTree” package (v 0.1.5) (50) was employed to draw cell trajectory diagram. Finally, the expression of prognostic genes during the differentiation process of key cells was also explored.

### 2.13 Prognostic genes expression and reverse transcription quantitative PCR

To clarify the manifestation of prognostic genes, in the PCa tissue and paracancer tissue samples of TCGA-PRAD, Wilcoxon test was employed to compare the differences in manifestation of prognostic genes between PCa samples and control samples (*P* < 0.05). Subsequently, to verify the accuracy of the above results, RT-qPCR was performed. A total of 5 pairs of tissue samples were obtained at Tangdu hospital, including 5 PCa and 5 paracancer (control). Informed consent was obtained from all patients for the use of PCa tissue samples in this study. The informed consent form needed to be signed and filled out by all participants, while the ethical approval agency was The Clinical Ethics Committee of Tangdu Hospital of Air Force Medical University (permission number: TDLL-KY-202405-18). Then, total RNA of 5 pairs of tissue samples was extracted by TRizol reagent (Vazyme, Nanjing, Jiangsu). The RNA concentrations were computed by NanoPhotometer N50. Subsequently, mRNA was converted to cDNA by Hifair^®^ III 1st Strand cDNA Synthesis SuperMix for qPCR test kit (Yeasen, Shanghai). Finally, RT-qPCR was carried out. The primers of reaction reagents, reaction conditions and genes were arranged in **Supplementary Table 2**. The internal reference gene was GAPDH, which was employed to normalize the results. The expression levels of prognostic genes were calculated by 2^-ΔΔCt^. The results were calculated by GraphPad Prism software (v 5.0) (51).

### 2.14 Western blotting

Cells were lysed in RIPA buffer (P0013B, Beyotime) to extract proteins, and protein concentrations were determined using a BCA assay kit (P0012, Beyotime). Samples were separated by SDS-PAGE (12%, Invitrogen) and transferred to PVDF membranes, which were then blocked with BSA for 2.5 h. After washing, membranes were incubated overnight at 4°C with primary antibodies, including anti-BMP2 (ab284387, 1:1000, Abcam) and anti-FASN (ab128870, 1:1000, Abcam). The next day, membranes were washed three times and incubated with HRP-conjugated goat anti-rabbit IgG secondary antibody (G-21234, 1:5000, Invitrogen) at room temperature for 2 h. Chemiluminescent substrate (ECL) was applied evenly to the membranes, and signals were captured using a ChemiDoc XRS+ imaging system (Bio-Rad, USA). Band intensities were analyzed with ImageJ, and protein levels were quantified as the ratio of the target band intensity to that of β-actin (ab272085, 1:1000, Abcam).

### 2.15 Ki67 staining

Vector, OE-BMP2, sh-NC, and sh-FASN cells were seeded and allowed to adhere for 24 h. Cells were washed twice with phosphate-buffered saline (PBS) and fixed with 4% paraformaldehyde for 20 min. Permeabilization was performed with 0.3% Triton X-100 for 15 min, followed by blocking with 6% donkey serum for 30 min. Cells were incubated overnight at 4°C with primary antibody against Ki67 (1:200 in PBS). The next day, cells were incubated with a fluorescent secondary antibody (1:500 in PBS) for 1 h at 37°C in the dark. After three PBS washes, nuclei were counterstained with DAPI for 10 min. Ki67 expression was assessed using a fluorescence microscope.

### 2.16 Scratch-wound assay

Vector, OE-BMP2, sh-NC, and sh-FASN cells (1×10^6 cells/well) were seeded in 6-well plates and allowed to adhere for 24 h. Once confluence exceeded 90%, a straight scratch perpendicular to the horizontal axis was made using a sterile 20-µL pipette tip. To remove debris, cells were rinsed with serum-free DMEM. Plates were incubated, and scratch closure was monitored at 24 h and 48 h after wounding under a high-power microscope. Migrating cells within the scratch area were quantified using ImageJ.

### 2.17 Cell culture and viral preparation

Human prostate cancer PC3 cells (ATCC^®^ CRL-1435™) were maintained in RPMI-1640 medium (Gibco) supplemented with 10% fetal bovine serum (FBS; Corning) and 1% penicillin/streptomycin (HyClone) at 37°C with 5% CO_2_. Lentiviral particles expressing shRNA targeting human fatty acid synthase (FASN; target sequence: shRNA1:5′-GCATGGAGCGTATCTGTGAGAACTCGAGTTCTCACAGATACGCTCCATGTTTTTT-3′ shRNA2: 5′-GCTACGACTACGGCCCTCATTCTCGAGAATGAGGGCCGTAGTCGTAGCTTTTTT-3′) or non-targeting scramble control (shRNA-NC: 5′-GCACCCAGTCCGCCCTGAGCAAATTCAAGAGATTTGCTCAGGGCGGACTGGGTGCTTTTT-3′) in pLKO.1 vector were packaged in HEK293T cells using psPAX2 and pMD2.G plasmids. Viral supernatants were concentrated by ultracentrifugation (26,000 × g, 2.5 h) and titers determined via qPCR.

The human BMP2 ORF (NM_001200) was cloned into pLV-CMV-3flag-zsgreen vector via NotI and NheI site. Lentivirus production and titration followed procedures (titer ≥5×10^7^ TU/mL). PC3 cells were transduced at MOI=20 with polybrene supplementation.

Cells were seeded in 6-well plates (5×105 cells/well) and incubated for 24 h to reach 50–60% confluency. Transduction was performed by replacing medium with viral suspension (MOI=20) containing 8 μg/mL polybrene (Sigma).

### 2.18 Statistical analysis

R software (v 3.9.1) was implemented to apply bioinformatics analyses. Wilcoxon test and t test were utilized to compare the disparities between 2 groups (*P* < 0.05).

## 3 Results

### 3.1 Identification and functions of candidate genes in PCa

**Figure 1:**
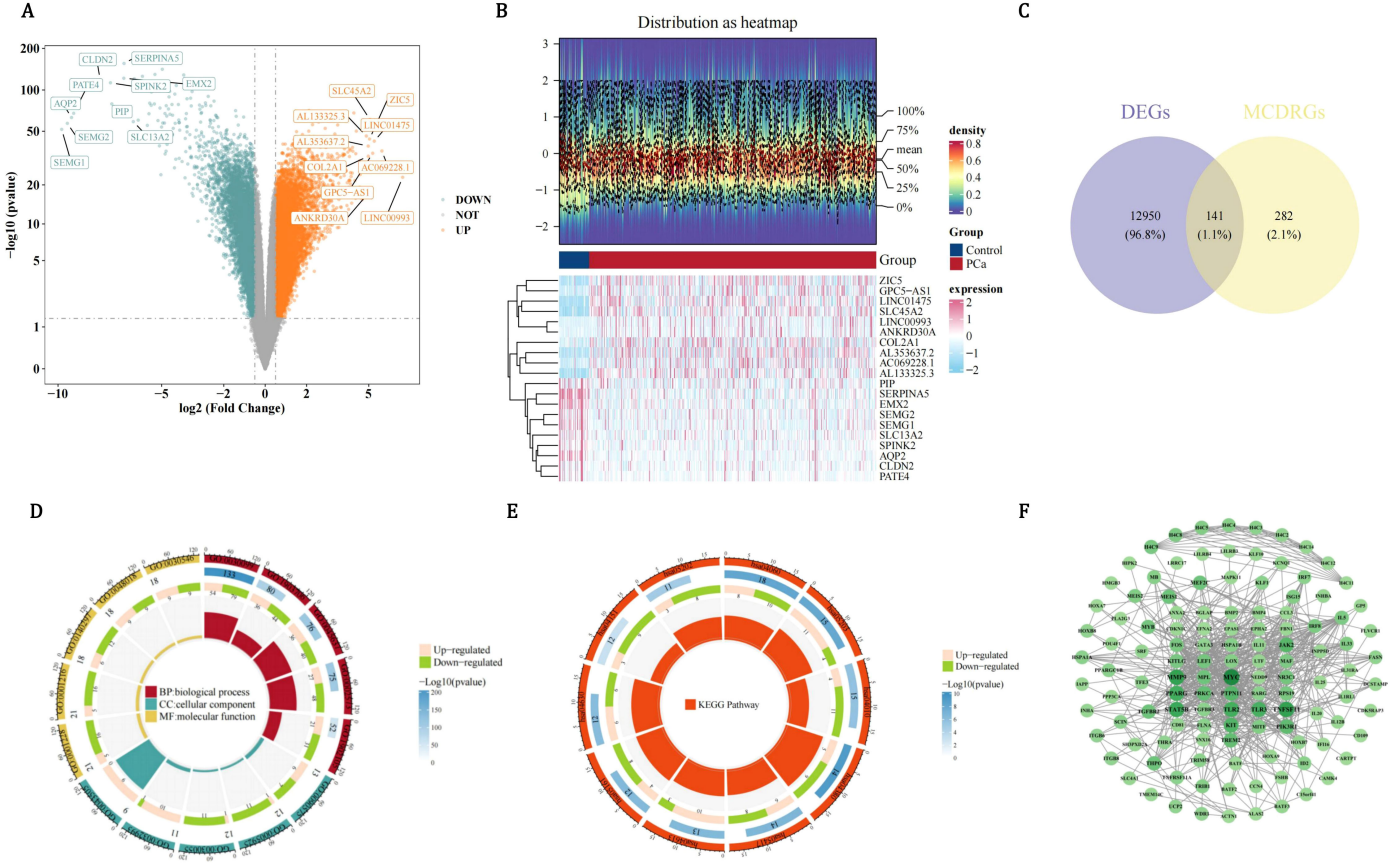
Multi-dimensional characterization of differentially expressed genes (DEGs) and functional annotation of candidate genes in pca. **(A)** Volcano plot of DEGs in pca. Orange dots represent upregulated genes, glaucous dots represent downregulated genes. **(B)** Heatmap analysis of top differentially expressed genes in PCa versus control groups. **(C)** Venn map to obtain a total of 141 candidate genes shared by DEGs and MCDRGs. **(D)** Functional enrichment of 141 candidate genes across GO categories. **(E)** Functional enrichment of candidate genes in KEGG pathways. **(F)** PPI network constructed using the 122 candidate genes.

By differential expression analysis, 13,091 DEGs were acquired, comprising 7,935 DEGs with up-regulated expression and 5,156 DEGs with down-regulated expression. All DEGs and the top 10 up/down-regulated gene names were presented (**Figure 1A**). Besides, the expressions of the top 10 up/down-regulated genes in PCa and control groups were also displayed (**Figure 1B**). After that, 141 candidate genes were acquired (**Figure 1C**). GO analysis indicated that candidate genes were enriched in 1,758 functions, including 1,619 biological process (BP) items such as MCD, 37 cellular component (CC) items such as chromosome centromeric core domain, and 102 molecular function (MF) items such as cytokine receptor binding (**Figure 1D**, **Supplementary Table 3**). KEGG analysis suggested that candidate genes were enriched in 85 pathways such as osteoclast differentiation (**Figure 1E**, **Supplementary Table 4**). Subsequently, PPI network indicated that there were interactions among proteins encoded by 122 candidate genes such as MYC, H4C11, and MMP9 (**Figure 1F**).

### 3.2 Candidate prognostic genes with a causal relationship with PCa

In order to investigate the causal relationship between DE-MCDRGs and PCa outcomes, MR analysis was conducted using 141 candidate genes as exposure factors and PCa as the outcome. Firstly, the 46 candidate genes had a substantially causal relationship with PCa based on the IVW method (*P* < 0.05), including 23 risk factors (OR > 1) and 23 protective factors (OR < 1) (**Table 1**). The positive slope of the scatter plot indicated that the gene was a risk factor, while a negative slope indicated that it was a protective factor. The intercept close to 0 suggested the absence of confounding factors (**Supplementary Figure 1**). For protective factors, the effect size of each SNP was less than 0. In contrast, for risk factors, the effect size of each SNP was greater than 0 (**Supplementary Figure 2**). The symmetrical arrangement of SNPs around each exposure indicated that MR affirmed Mendel’s second law of randomization (**Supplementary Figure 3**). So 46 candidate genes were used for subsequent analysis. Notably, among the 46 candidate genes, heterogeneity was calculated for 35 of them. The P-values of 34 candidate genes were greater than 0.05, indicating the absence of heterogeneity. Since the P-value of SLC4A1 was less than 0.05, SLC4A1 was analyzed with random effects IVW (**Supplementary Table 5**). Meanwhile, among the 46 candidate genes, the P-values of 33 candidate genes were greater than 0.05, suggesting the lack of horizontal pleiotropy and the reliability of the results. However, the P-values of the remaining 13 candidate genes were less than 0.05, suggesting the presence of horizontal pleiotropy. To ensure the reliability of the results, these 13 candidate genes were excluded (**Supplementary Table 6**). LOO analysis demonstrated that the removal of any SNP had minimal impact on the results (**Supplementary Figure 4**). Steiger test indicated that only the P-values of 33 candidate genes were all less than 0.05 and the Steiger-dir values were all TRUE (**Supplementary Table 7**). Finally, taking all of the above screening results into account, only 23 candidate genes could be labeled as candidate prognostic genes.

**Table 1:** Detailed information of 46 candidate genes.

id.exposure	id.outcome	outcome	exposure	method	nsnp	b	se	pval	lo_ci	up_ci	or	or_lci95	or_uci95	SYMBOL	ENSEMBL
eqtl-a-ENSG 00000004939	ebi-a-GCST90018905	Prostate cancer || id:ebi-a-GCST90018905	|| id:eqtl-a-ENSG00000004939	Inverse variance weighted	56	-0.0862675	0.0225910626279179	0.000134180570998726	-0.13054599	-0.04198902	0.917348804786561	0.877616134134675	0.958880308727545	SLC4A1	ENSG00000004939
eqtl-a-ENSG 00000064393	ebi-a-GCST90018905	Prostate cancer || id:ebi-a-GCST90018905	|| id:eqtl-a-ENSG00000064393	Inverse variance weighted	24	0.114551071657029	0.0330413050676368	0.000526487579391663	0.0497901137244607	0.179312029589597	1.12136990982098	1.05105047215483	1.19639399625966	HIPK2	ENSG00000064393
eqtl-a-ENSG 00000071127	ebi-a-GCST90018905	Prostate cancer || id:ebi-a-GCST90018905	|| id:eqtl-a-ENSG00000071127	Inverse variance weighted	79	0.0593858404497922	0.0104363922851716	1.26842174221692e-08	0.0389305115708559	0.0798411693287284	1.06118460970665	1.03969823414286	1.08311502212624	WDR1	ENSG00000071127
eqtl-a-ENSG 00000072110	ebi-a-GCST90018905	Prostate cancer || id:ebi-a-GCST90018905	|| id:eqtl-a-ENSG00000072110	Inverse variance weighted	43	0.0491465356346644	0.0219565943499576	0.0251982616486359	0.00611161070874751	0.0921814605605812	1.05037425672174	1.00613032470627	1.09656378710764	ACTN1	ENSG00000072110
eqtl-a-ENSG 00000096968	ebi-a-GCST90018905	Prostate cancer || id:ebi-a-GCST90018905	|| id:eqtl-a-ENSG00000096968	Inverse variance weighted	49	-0.01772619	0.0086389619879774	0.0401804634102048	-0.03465856	-0.00079382	0.982429994707639	0.96593517322939	0.999206490508493	JAK2	ENSG00000096968
eqtl-a-ENSG 00000107485	ebi-a-GCST90018905	Prostate cancer || id:ebi-a-GCST90018905	|| id:eqtl-a-ENSG00000107485	Inverse variance weighted	4	0.24237628842888	0.0896452711357227	0.00685667152792146	0.0666715570028631	0.418081019854896	1.2742735970232	1.06894433323163	1.51904374212028	GATA3	ENSG00000107485
eqtl-a-ENSG 00000108465	ebi-a-GCST90018905	Prostate cancer || id:ebi-a-GCST90018905	|| id:eqtl-a-ENSG00000108465	Inverse variance weighted	107	-0.04377072	0.0077521170101473	1.63944291694687e-08	-0.05896487	-0.02857658	0.957173388759083	0.942739883441887	0.971827873457125	CDK5RAP3	ENSG00000108465
eqtl-a-ENSG 00000111843	ebi-a-GCST90018905	Prostate cancer || id:ebi-a-GCST90018905	|| id:eqtl-a-ENSG00000111843	Inverse variance weighted	45	-0.03765833	0.0106690629350972	0.000416069482528046	-0.05856969	-0.01674697	0.963041926095908	0.943112508334642	0.983392483104924	TMEM14C	ENSG00000111843
eqtl-a-ENSG 00000111859	ebi-a-GCST90018905	Prostate cancer || id:ebi-a-GCST90018905	|| id:eqtl-a-ENSG00000111859	Inverse variance weighted	35	0.0633158182927332	0.0227468498424664	0.00537761411855873	0.018731992601499	0.107899643983967	1.0653632473085	1.01890853699476	1.11393594960285	NEDD9	ENSG00000111859
eqtl-a-ENSG 00000113083	ebi-a-GCST90018905	Prostate cancer || id:ebi-a-GCST90018905	|| id:eqtl-a-ENSG00000113083	Inverse variance weighted	4	0.240902418694054	0.0955929986712215	0.0117326719113292	0.0535401412984594	0.428264696089648	1.27239686710211	1.05499933996101	1.53459222777437	LOX	ENSG00000113083
eqtl-a-ENSG 00000113580	ebi-a-GCST90018905	Prostate cancer || id:ebi-a-GCST90018905	|| id:eqtl-a-ENSG00000113580	Inverse variance weighted	15	-0.1087408	0.0407448763957071	0.00761179216284266	-0.18860075	-0.02888084	0.896962883154333	0.828117062810458	0.971532226405387	NR3C1	ENSG00000113580
eqtl-a-ENSG 00000115602	ebi-a-GCST90018905	Prostate cancer || id:ebi-a-GCST90018905	|| id:eqtl-a-ENSG00000115602	Inverse variance weighted	54	-0.07489375	0.00890487936063928	4.08556381257334e-17	-0.09234731	-0.05744018	0.927842067344744	0.911788421057661	0.944178366441578	IL1RL1	ENSG00000115602
eqtl-a-ENSG 00000117400	ebi-a-GCST90018905	Prostate cancer || id:ebi-a-GCST90018905	|| id:eqtl-a-ENSG00000117400	Inverse variance weighted	34	0.0404166152124666	0.019398826358663	0.0372096124372029	0.00239491554948718	0.0784383148754461	1.04124448213261	1.00239778565049	1.08159663468134	MPL	ENSG00000117400
eqtl-a-ENSG 00000123685	ebi-a-GCST90018905	Prostate cancer || id:ebi-a-GCST90018905	|| id:eqtl-a-ENSG00000123685	Inverse variance weighted	77	0.054284628038081	0.00830519795201091	6.30917135934331e-11	0.0380064400521396	0.0705628160240224	1.05578506542472	1.03873792238334	1.07311197594125	BATF3	ENSG00000123685
eqtl-a-ENSG 00000125845	ebi-a-GCST90018905	Prostate cancer || id:ebi-a-GCST90018905	|| id:eqtl-a-ENSG00000125845	Inverse variance weighted	7	0.0458902149113594	0.0217529790919432	0.0348923082947351	0.00325437589115062	0.0885260539315681	1.04695946410976	1.00325967712154	1.09256271779395	BMP2	ENSG00000125845
eqtl-a-ENSG 00000128606	ebi-a-GCST90018905	Prostate cancer || id:ebi-a-GCST90018905	|| id:eqtl-a-ENSG00000128606	Inverse variance weighted	32	-0.13228888	0.0352149624054393	0.000172230629152546	-0.2013102	-0.06326755	0.876087876277796	0.817658751189363	0.938692291673627	LRRC17	ENSG00000128606
eqtl-a-ENSG 00000132170	ebi-a-GCST90018905	Prostate cancer || id:ebi-a-GCST90018905	|| id:eqtl-a-ENSG00000132170	Inverse variance weighted	70	0.0477285863886345	0.00878376117471714	5.51880757828691e-08	0.0305124144861889	0.0649447582910801	1.04888593476824	1.03098268908969	1.06710007432426	PPARG	ENSG00000132170
eqtl-a-ENSG 00000134198	ebi-a-GCST90018905	Prostate cancer || id:ebi-a-GCST90018905	|| id:eqtl-a-ENSG00000134198	Inverse variance weighted	92	-0.03764182	0.0127798397254149	0.00322530836893276	-0.06269031	-0.01259333	0.963057826935287	0.939234303777758	0.987485629827228	TSPAN2	ENSG00000134198
eqtl-a-ENSG 00000137462	ebi-a-GCST90018905	Prostate cancer || id:ebi-a-GCST90018905	|| id:eqtl-a-ENSG00000137462	Inverse variance weighted	13	0.10271715170461	0.0379680843189267	0.00682314021173868	0.0282997064395134	0.177134596969706	1.10817791850772	1.02870394742231	1.19379176306782	TLR2	ENSG00000137462
eqtl-a-ENSG 00000138814	ebi-a-GCST90018905	Prostate cancer || id:ebi-a-GCST90018905	|| id:eqtl-a-ENSG00000138814	Inverse variance weighted	72	-0.10804319	0.0105997012886228	2.12995398123504e-24	-0.1288186	-0.08726777	0.897588829506295	0.879133422665484	0.916431665641544	PPP3CA	ENSG00000138814
eqtl-a-ENSG 00000141655	ebi-a-GCST90018905	Prostate cancer || id:ebi-a-GCST90018905	|| id:eqtl-a-ENSG00000141655	Inverse variance weighted	37	-0.16351257	0.0224265965716498	3.07637026634505e-13	-0.2074687	-0.11955644	0.849155826423771	0.812638678869106	0.887313927209194	TNFRSF11A	ENSG00000141655
eqtl-a-ENSG 00000155090	ebi-a-GCST90018905	Prostate cancer || id:ebi-a-GCST90018905	|| id:eqtl-a-ENSG00000155090	Inverse variance weighted	8	-0.11242	0.0379585066899162	0.00305990347011228	-0.18681868	-0.03802133	0.893668833746529	0.829594146751466	0.962692405120287	KLF10	ENSG00000155090
eqtl-a-ENSG 00000156535	ebi-a-GCST90018905	Prostate cancer || id:ebi-a-GCST90018905	|| id:eqtl-a-ENSG00000156535	Inverse variance weighted	41	-0.08060926	0.0203936839274167	7.72878087053537e-05	-0.12058088	-0.04063764	0.922554098558626	0.88640539032865	0.960176995823273	CD109	ENSG00000156535
eqtl-a-ENSG 00000157404	ebi-a-GCST90018905	Prostate cancer || id:ebi-a-GCST90018905	|| id:eqtl-a-ENSG00000157404	Inverse variance weighted	8	0.182018543500395	0.06482477641438	0.00498727464639933	0.0549619817282107	0.30907510527258	1.1996364391324	1.0565004475908	1.36216467241069	KIT	ENSG00000157404
eqtl-a-ENSG 00000158406	ebi-a-GCST90018905	Prostate cancer || id:ebi-a-GCST90018905	|| id:eqtl-a-ENSG00000158406	Inverse variance weighted	210	-0.0559846	0.0109965599228382	3.55989998452472e-07	-0.07753786	-0.03443134	0.945553698113044	0.92539199154423	0.966154671949654	H4C8	ENSG00000158406
eqtl-a-ENSG 00000161800	ebi-a-GCST90018905	Prostate cancer || id:ebi-a-GCST90018905	|| id:eqtl-a-ENSG00000161800	Inverse variance weighted	19	0.178052231452901	0.0267499420197511	2.81050162609673e-11	0.125622345094189	0.230482117811614	1.19488773032714	1.13385388193491	1.25920694970844	RACGAP1	ENSG00000161800
eqtl-a-ENSG 00000162769	ebi-a-GCST90018905	Prostate cancer || id:ebi-a-GCST90018905	|| id:eqtl-a-ENSG00000162769	Inverse variance weighted	69	0.0623425099599805	0.00642645238358816	2.98787806011948e-22	0.0497466632881477	0.0749383566318133	1.06432682484362	1.05100480454538	1.07781770852294	FLVCR1	ENSG00000162769
eqtl-a-ENSG 00000163565	ebi-a-GCST90018905	Prostate cancer || id:ebi-a-GCST90018905	|| id:eqtl-a-ENSG00000163565	Inverse variance weighted	41	-0.02952343	0.0133992842959388	0.0275694925208712	-0.05578602	-0.00326083	0.970908132284768	0.945741480377176	0.99674448133622	IFI16	ENSG00000163565
eqtl-a-ENSG 00000164038	ebi-a-GCST90018905	Prostate cancer || id:ebi-a-GCST90018905	|| id:eqtl-a-ENSG00000164038	Inverse variance weighted	52	-0.13185749	0.0228102387198681	7.44226335388354e-09	-0.17656555	-0.08714942	0.876465894074618	0.838143833033144	0.916540136907076	SLC9B2	ENSG00000164038
eqtl-a-ENSG 00000164342	ebi-a-GCST90018905	Prostate cancer || id:ebi-a-GCST90018905	|| id:eqtl-a-ENSG00000164342	Inverse variance weighted	23	-0.06937861	0.0245646123671737	0.00473793445997668	-0.11752525	-0.02123197	0.932973380213239	0.889118061079602	0.978991841791624	TLR3	ENSG00000164342
eqtl-a-ENSG 00000166147	ebi-a-GCST90018905	Prostate cancer || id:ebi-a-GCST90018905	|| id:eqtl-a-ENSG00000166147	Inverse variance weighted	28	0.0335648955225129	0.0095301804404938	0.000428371957392368	0.014885741859145	0.0522440491858807	1.03413455225085	1.01499708631006	1.05363284937784	FBN1	ENSG00000166147
eqtl-a-ENSG 00000168062	ebi-a-GCST90018905	Prostate cancer || id:ebi-a-GCST90018905	|| id:eqtl-a-ENSG00000168062	Inverse variance weighted	27	0.31042371852584	0.0902949917829467	0.000586278529946131	0.133445534631265	0.487401902420416	1.36400294502181	1.1427590236485	1.62808080752505	BATF2	ENSG00000168062
eqtl-a-ENSG 00000169071	ebi-a-GCST90018905	Prostate cancer || id:ebi-a-GCST90018905	|| id:eqtl-a-ENSG00000169071	Inverse variance weighted	31	-0.04619047	0.0101785342691725	5.67827480227254e-06	-0.0661404	-0.02624055	0.954860070243676	0.935999441016213	0.974100745996028	ROR2	ENSG00000169071
eqtl-a-ENSG 00000169710	ebi-a-GCST90018905	Prostate cancer || id:ebi-a-GCST90018905	|| id:eqtl-a-ENSG00000169710	Inverse variance weighted	36	0.0534674279546715	0.0106476673448896	5.12653322410322e-07	0.0325979999586878	0.0743368559506552	1.05492263022025	1.03313513538361	1.07716959537689	FASN	ENSG00000169710
eqtl-a-ENSG 00000170271	ebi-a-GCST90018905	Prostate cancer || id:ebi-a-GCST90018905	|| id:eqtl-a-ENSG00000170271	Inverse variance weighted	126	0.0153732230518441	0.00534740283895475	0.00404161551542964	0.00489231348749284	0.0258541326161955	1.01549199892077	1.00490430039305	1.02619124972274	FAXDC2	ENSG00000170271
eqtl-a-ENSG 00000173757	ebi-a-GCST90018905	Prostate cancer || id:ebi-a-GCST90018905	|| id:eqtl-a-ENSG00000173757	Inverse variance weighted	26	0.0610771741022094	0.0304128217978681	0.044614550495404	0.00146804337838798	0.120686304826031	1.06298094562195	1.00146912148157	1.12827092370428	STAT5B	ENSG00000173757
eqtl-a-ENSG 00000178467	ebi-a-GCST90018905	Prostate cancer || id:ebi-a-GCST90018905	|| id:eqtl-a-ENSG00000178467	Inverse variance weighted	132	-0.03584894	0.00496000630554148	4.9160298788783e-13	-0.04557056	-0.02612733	0.964786020014617	0.955452187779586	0.974211034650301	P4HTM	ENSG00000178467
eqtl-a-ENSG 00000178732	ebi-a-GCST90018905	Prostate cancer || id:ebi-a-GCST90018905	|| id:eqtl-a-ENSG00000178732	Inverse variance weighted	12	-0.08868522	0.0405309171285067	0.0286631834831755	-0.16812582	-0.00924462	0.915133595267459	0.84524748218542	0.990797979098186	GP5	ENSG00000178732
eqtl-a-ENSG 00000179295	ebi-a-GCST90018905	Prostate cancer || id:ebi-a-GCST90018905	|| id:eqtl-a-ENSG00000179295	Inverse variance weighted	83	0.0627228479547001	0.0125942271571552	6.34905112119879e-07	0.0380381627266759	0.0874075331827243	1.06473170576482	1.03877087445104	1.09134134691635	PTPN11	ENSG00000179295
eqtl-a-ENSG 00000180354	ebi-a-GCST90018905	Prostate cancer || id:ebi-a-GCST90018905	|| id:eqtl-a-ENSG00000180354	Inverse variance weighted	74	-0.02552713	0.0125056240523145	0.0412258253942992	-0.05003815	-0.0010161	0.974795937188781	0.951193137480542	0.998984414118728	MTURN	ENSG00000180354
eqtl-a-ENSG 00000187098	ebi-a-GCST90018905	Prostate cancer || id:ebi-a-GCST90018905	|| id:eqtl-a-ENSG00000187098	Inverse variance weighted	12	0.104124515850197	0.0420436810623215	0.013264810420383	0.021718900968047	0.186530130732347	1.10973862636169	1.02195647311557	1.20506093090706	MITF	ENSG00000187098
eqtl-a-ENSG 00000187608	ebi-a-GCST90018905	Prostate cancer || id:ebi-a-GCST90018905	|| id:eqtl-a-ENSG00000187608	Inverse variance weighted	21	-0.0846937	0.0298482583414865	0.004547184794765	-0.14319629	-0.02619111	0.918793668402591	0.866583954162597	0.974148899297871	ISG15	ENSG00000187608
eqtl-a-ENSG 00000196924	ebi-a-GCST90018905	Prostate cancer || id:ebi-a-GCST90018905	|| id:eqtl-a-ENSG00000196924	Inverse variance weighted	10	-0.9905431	0.0733174240714497	1.3589808726124e-41	-1.13424525	-0.84684095	0.371374942078478	0.321664805498035	0.428767292058116	FLNA	ENSG00000196924
eqtl-a-ENSG 00000204388	ebi-a-GCST90018905	Prostate cancer || id:ebi-a-GCST90018905	|| id:eqtl-a-ENSG00000204388	Inverse variance weighted	185	0.153064681220343	0.0121280804092711	1.62402672376596e-36	0.129293643618172	0.176835718822514	1.16540035602219	1.13802424866451	1.19343501811183	HSPA1B	ENSG00000204388
eqtl-a-ENSG 00000204389	ebi-a-GCST90018905	Prostate cancer || id:ebi-a-GCST90018905	|| id:eqtl-a-ENSG00000204389	Inverse variance weighted	10	-0.45537219	0.0909913432375831	5.598799413757e-07	-0.63371523	-0.27702916	0.634211874764211	0.530616773319071	0.758032392334627	HSPA1A	ENSG00000204389
eqtl-a-ENSG 00000204577	ebi-a-GCST90018905	Prostate cancer || id:ebi-a-GCST90018905	|| id:eqtl-a-ENSG00000204577	Inverse variance weighted	13	0.0946901017039308	0.04673632445415	0.0427597235054622	0.00308690577379674	0.186293297634065	1.09931812551913	1.00309167517372	1.20477556638639	LILRB3	ENSG00000204577

### 3.3 The value of prognostic genes in risk models

**Figure 2:**
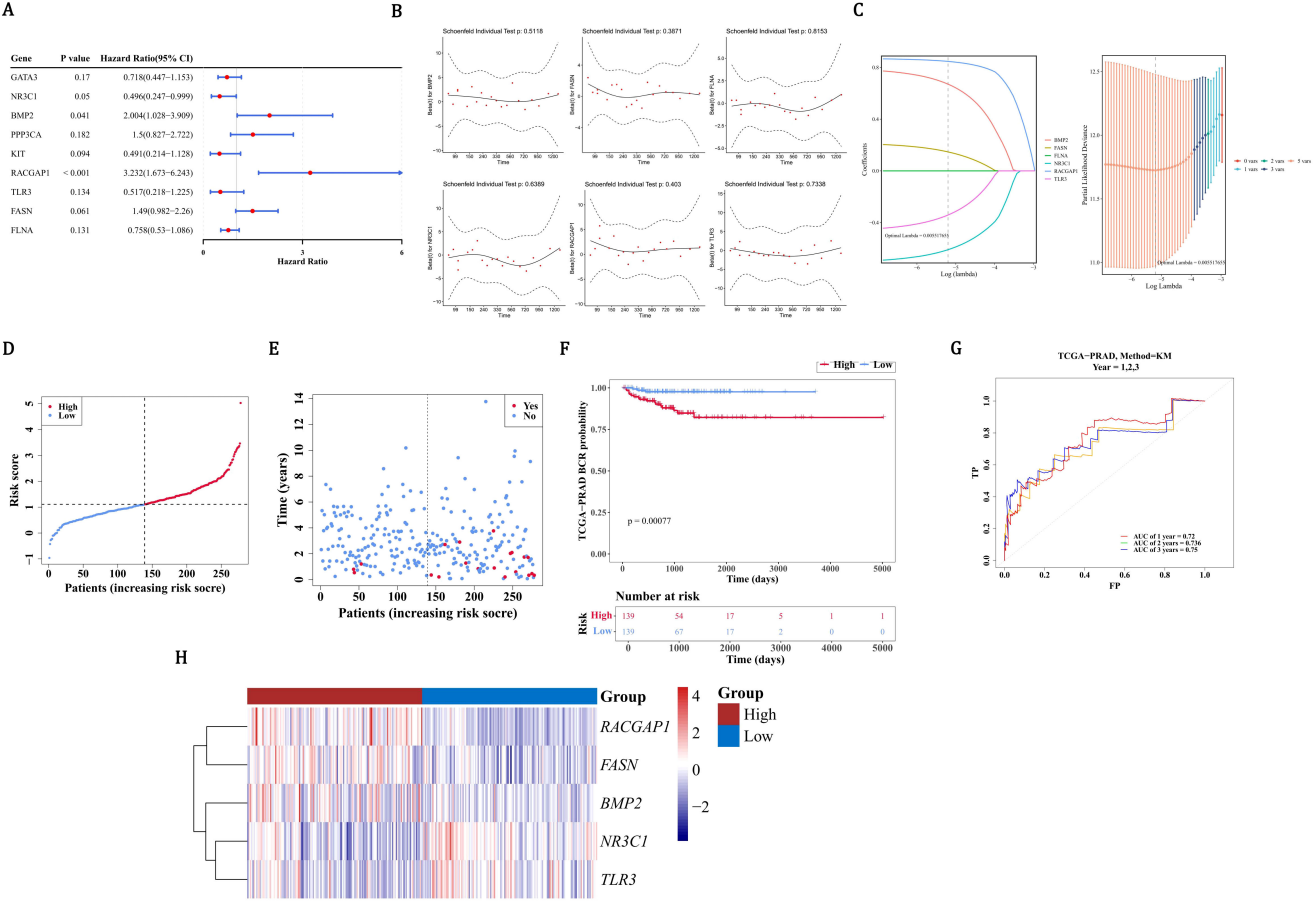
Development and validation of a prognostic gene signature for PCa recurrence. **(A)** Univariate Cox regression analysis identified 9 candidate genes. **(B)** MR analysis and PH assumption testing confirmed consistency in 6 candidate genes. **(C)** LASSO regression selected 5 prognostic genes (NR3C1, BMP2, RACGAP1, TLR3, FASN) for model construction. **(D)** Risk score distribution (median=1.11) in TCGA-PRAD cohort (n=278) divided into high- (HRG) and low-risk (LRG) groups. **(E)** Survival status distribution between risk groups. **(F)** Significant survival difference by Kaplan-Meier analysis. **(G)** ROC validation showing predictive accuracy (AUC>0.7). **(H)** Differential expression patterns of signature genes across risk groups, red indicates HRG, blue indicates LRG.

In order to further screen for genes related to prognosis among the 23 candidate prognostic genes, univariate Cox regression analysis was conducted. The 9 candidate prognostic genes associated with PCa recurrence were gained utilizing univariate Cox regression analysis (*P* < 0.2), including 4 risk factors (HR > 1) and 5 protective factors (HR < 1) (**Figure 2A**). Notably, compared with the results of the MR analysis, only the OR/HR of 6 candidate prognostic genes was consistent. Besides, these 6 candidate prognostic genes were also identified by PH assumption test (*P* > 0.05) (**Figure 2B**). Among them, 5 candidate prognostic genes were identified by LASSO analysis (lambda.min=0.005517655) (**Figure 2C**). So NR3C1, BMP2, RACGAP1, TLR3, and FASN were considered as prognostic genes to build the risk model in TCGA-PRAD-train. On the basis of the median of the risk scores (1.111923), PCa patients were divided into a HRG (139 PCa patients) and a LRG (139 PCa patients). Patients’ risk score distribution (**Figure 2D**) and survival status (**Figure 2E**) were displayed. The survival curves indicated that the survival rate of the HRG was substantially lower than that of the LRG (P=0.00077) (**Figure 2F**). The AUC values of the ROC curves were all greater than 0.7, indicating that the risk model had good performance (**Figure 2G**). In addition, BMP2, RACGAP1, and FASN were prominently expressed in the HRG, while TLR3 and NR3C1 were prominently expressed in the LRG (**Figure 2H**).

### 3.4 Validation of risk models

**Figure 3:**
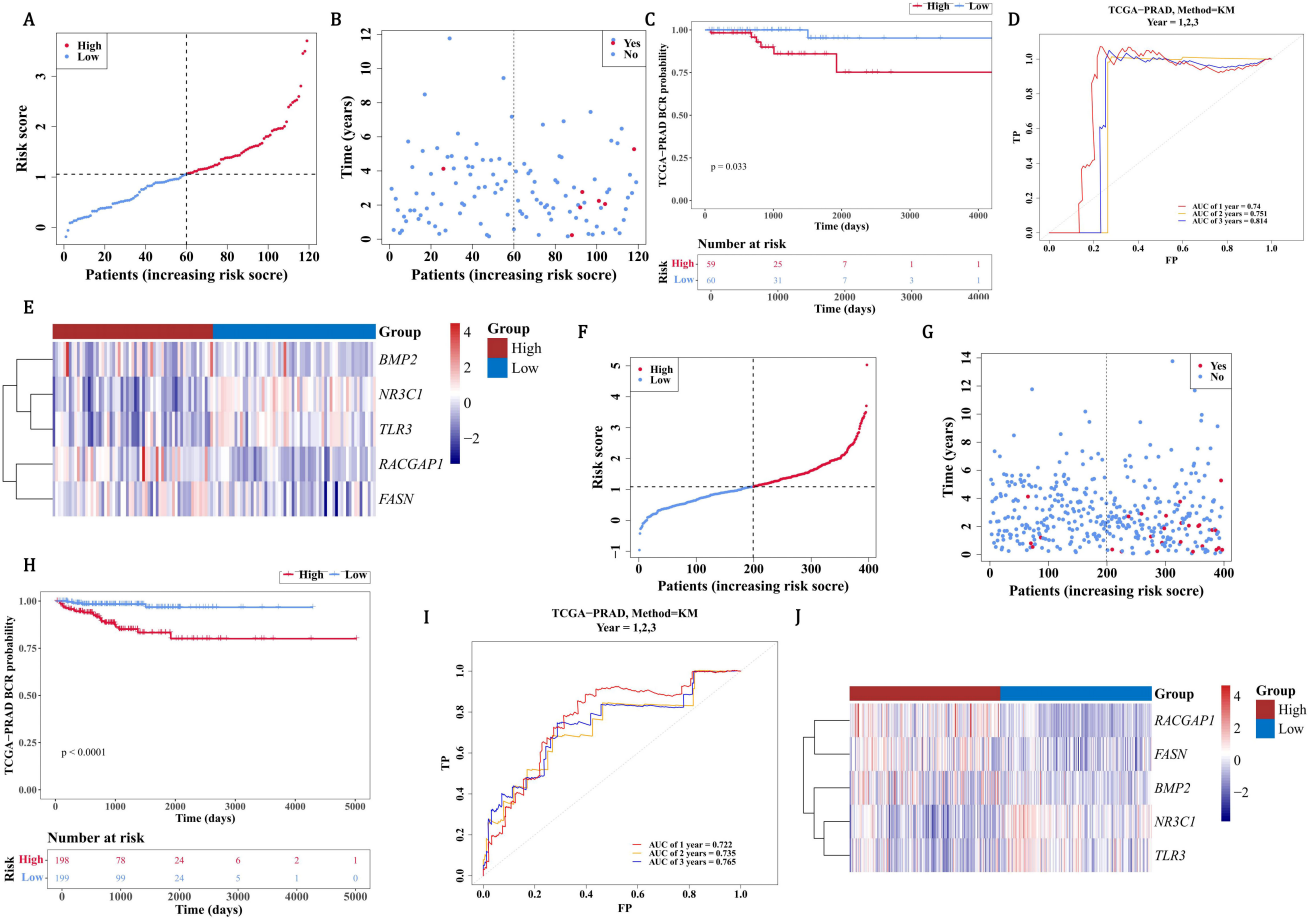
Validation of PCa recurrence risk model in TCGA-PRAD cohorts. **(A)** Risk score distribution stratified by median cutoff (1.057) in validation cohort (HRG: 59 patients, red; LRG: 60 patients, blue). **(B)** Survival status mapping across ordered risk scores. **(C)** Significant survival divergence between groups by Kaplan-Meier analysis (P=0.033). **(D)** ROC validation demonstrating robust 1-/3-/5-year recurrence prediction (AUC>0.7). **(E)** Heatmap contrasting elevated expression of BMP2/RACGAP1/FASN in HRG (red) versus TLR3/NR3C1 in LRG (blue). **(F-H)** Relapse cohort stratification (median=1.086) with risk distribution (198 HRG vs 199 LRG) and survival status. **(I)** Superior prognostic accuracy in relapse-specific ROC analysis (AUC>0.7). **(J)** Conserved expression dichotomy of signature genes across relapse subgroups.

In order to assess the accuracy of risk prediction, the ROC curve was used to calculate the AUC value based on the TCGA-PRAD training set and the GSE116918 dataset to evaluate the model’s effectiveness. In the TCGA-PRAD-validation, on the basis of the median of the risk scores (1.056659), PCa patients were separated into a HRG (59 PCa patients) and a LRG (60 PCa patients). Patients’ risk score distribution (**Figure 3A**) and survival status (**Figure 3B**) were also displayed. The survival curves indicated that HRG had a lower survival rate than LRG (P=0.033) (**Figure 3C**). As the AUC values were all above 0.7, it demonstrated that the risk model had excellent performance (**Figure 3D**). BMP2, RACGAP1, and FASN exhibited high expression in the HRG, with TLR3 and NR3C1 showing high expression in the LRG (**Figure 3E**). Similarly, in all samples with relapse information of TCGA-PRAD, on the basis of the median of the risk scores (1.085715), PCa patients were categorized into a HRG (198 PCa patients) and a LRG (199 PCa patients). The risk score distribution (**Figure 3F**) among patients and their survival status (**Figure 3G**) were likewise shown. According to the survival curves, the survival rate in the HRG was markedly lower than that in the LRG (*P* < 0.0001) (**Figure 3H**). Given that the AUC values all exceeded 0.7, it was evident that the risk model performed well (**Figure 3I**). High expression of BMP2, RACGAP1, and FASN was observed in the HRG, while high expression of TLR3 and NR3C1 was found in the LRG (**Figure 3J**). Based on the results of 3.4, the risk model was accurate. In the GSE116918 dataset, there was a significant survival difference between the high and low-risk groups (p=0.014), but the ROC curves for recurrence at 1, 2, and 3 years showed that the AUC values were all less than 0.6. This suggests that although the risk score was significantly associated with survival differences, its accuracy in predicting recurrence remains limited. Further validation and optimization may be needed, possibly by integrating other clinical indicators or more comprehensive models (**Supplementary Figure 5**).

### 3.5 The predictive ability of prognostic genes for PCa patients

**Figure 4:**
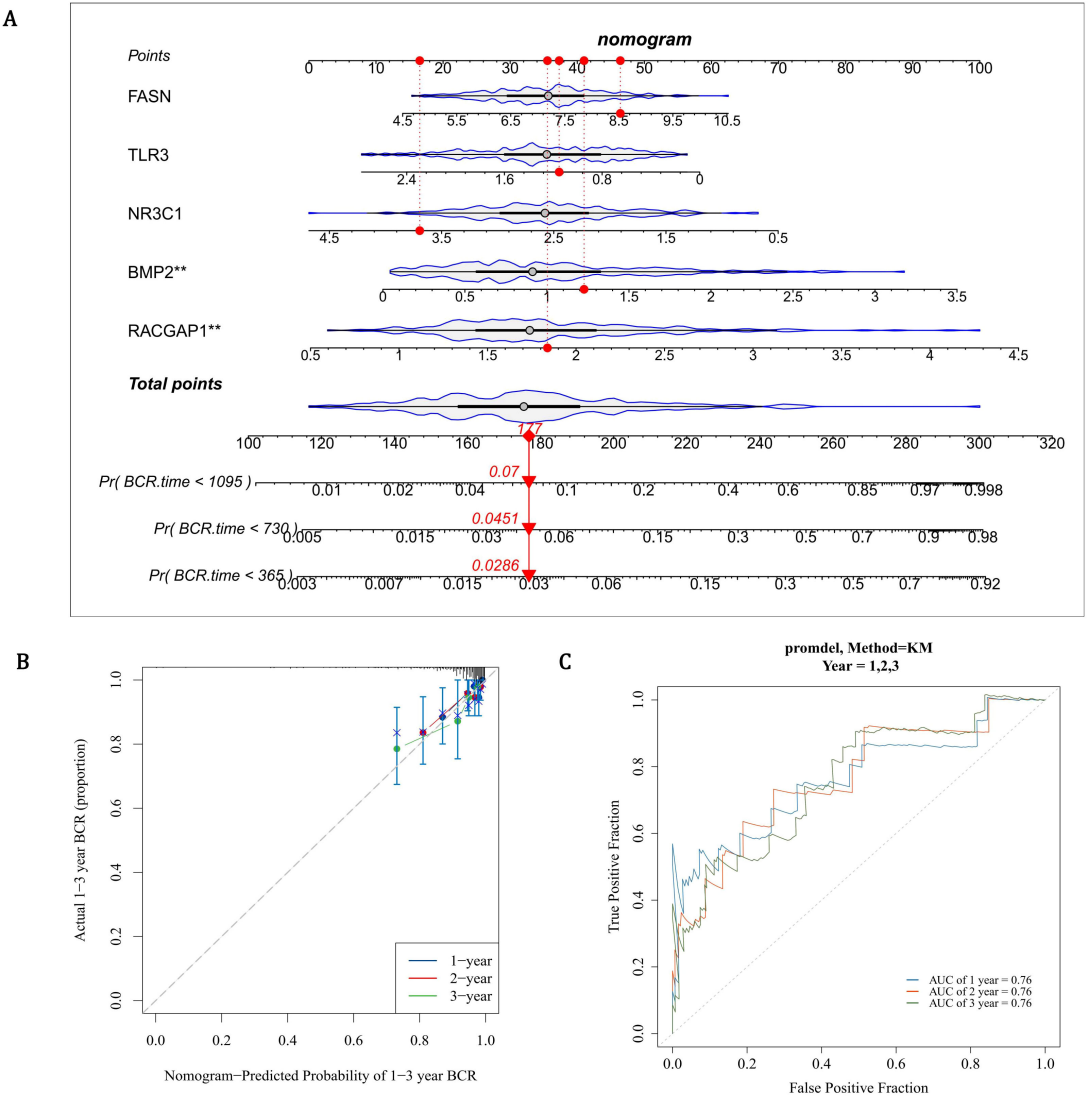
Development and validation of a gene-based nomogram for PCa recurrence prediction. **(A)** Prognostic nomogram integrating FASN, TLR3, NR3C1, BMP2, and RACGAP1 expression scores to estimate biochemical recurrence (BCR) probabilities at 1-, 3-, and 5-year intervals. Cumulative risk scores correlate with escalating recurrence rates (Pr[BCR]). **(B)** Calibration curves demonstrate high concordance between predicted and observed outcomes (slope ≈1) for 1- and 3-year BCR predictions, with narrow 95% confidence intervals. **(C)** ROC curves validate robust discriminative capacity, with AUC values exceeding 0.7 across all time points.

The nomogram indicated that prognostic genes could predict the return rate of PCa patients quite well, and the recurrence rate increased as the total score of the nomogram rose (**Figure 4A**). The slope of all calibration curves was close to 1, indicating the accuracy of the nomogram model (**Figure 4B**). The AUC values of ROC curves all exceeded 0.7, which further verified the accuracy of the nomogram model (**Figure 4C**). In conclusion, prognostic genes could be utilized to predict the recurrence rate of PCa patients.

### 3.6 Tumor microenvironment and immune cells in PCa

**Figure 5:**
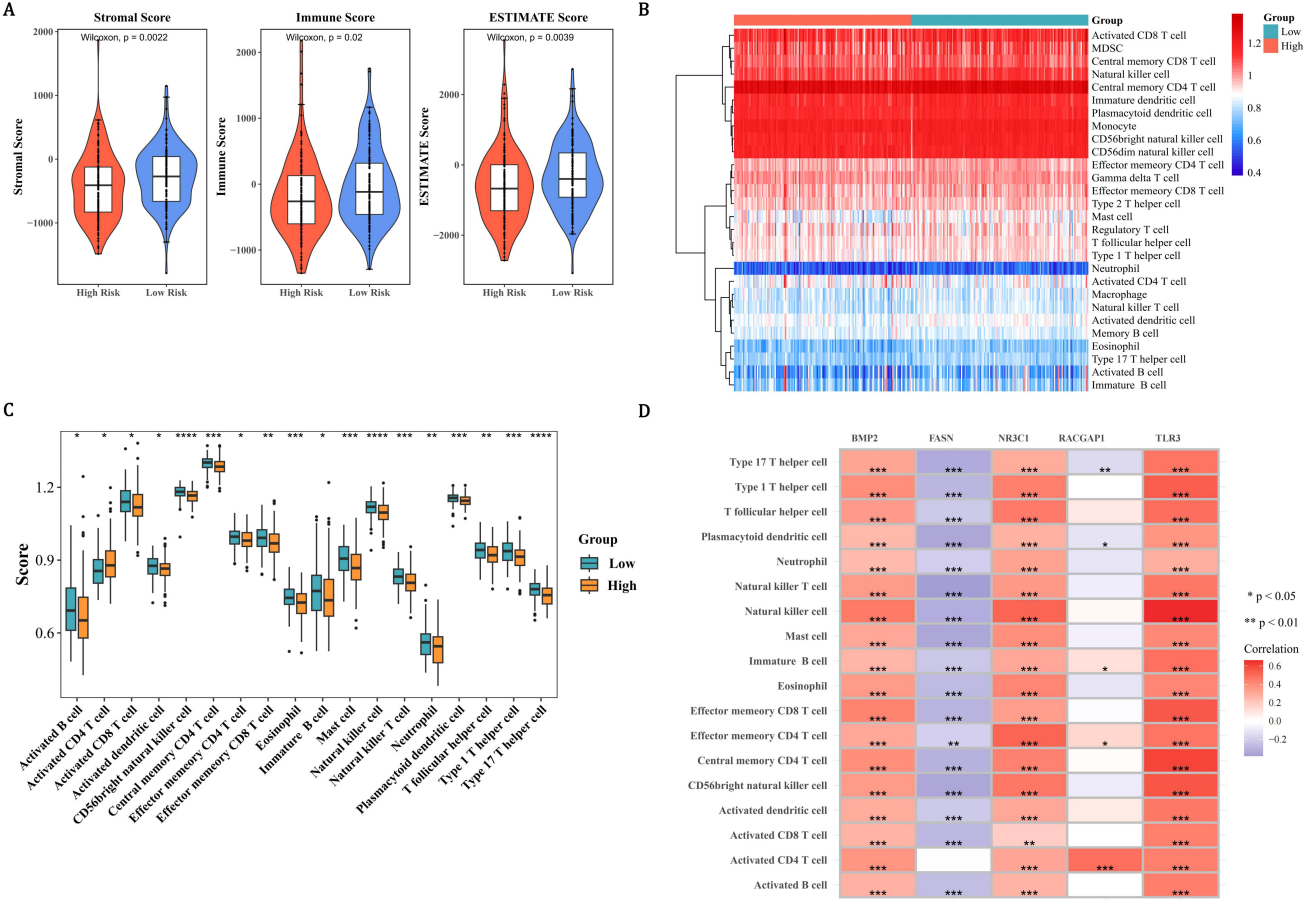
Tumor microenvironment and immune landscape stratification in PCa risk groups. **(A)** Violin plots demonstrating elevated stromal, immune, and ESTIMATE scores in the LRG compared to the HRG. **(B)** Heatmap of 28 immune cell infiltration profiles (blue: low; red: high) revealing distinct immune microenvironments between risk groups. **(C)** Box plots identifying 18 differentially infiltrated immune cells (P < 0.05), with LRG showing higher infiltration in all cell types except activated CD4 T cells. **(D)** Correlation matrix highlighting TLR3-NK cell synergy (cor=0.66, P < 0.0001) and FASN-NKT cell antagonism (cor=−0.38, P < 0.0001).

In order to assess tumor purity, immune infiltration, and stromal infiltration in the malignant tumor tissue within the patient’s tumor microenvironment, immune infiltration analysis was conducted. Within the microenvironment specific to PCa tumors, immune scores, ESTIMATE scores, and the stromal scores in the LRG were all strikingly higher than those in the HRG (*P* < 0.05) (**Figure 5A**). Subsequently, the infiltration levels of 28 immune cells in the HRG and LRG were analyzed (**Figure 5B**). There were 18 immune cells that showed notable distinctions between the HRG and the LRG, and they were defined as differentially expressed immune cells (*P* < 0.05) (**Figure 5C**, **Supplementary Table 8**). Except for the activated CD4 T cells, the extents of infiltration of the remaining cells in the LRG were all remarkably higher than those in the HRG (*P* < 0.05). TLR3 had the largest notable positive link with natural killer cells (cor=0.66, *P* < 0.0001) and FASN had the largest notable negative correlation with natural killer (NK) T cells (cor=-0.38, *P* < 0.0001) (**Figure 5D**, **Supplementary Table 9**). In summary, the occurrence and development of PCa might be related to changes in the tumor microenvironment or some immune cells.

### 3.7 Enrichment pathways and drug sensitivity in HRG and LRG

**Figure 6:**
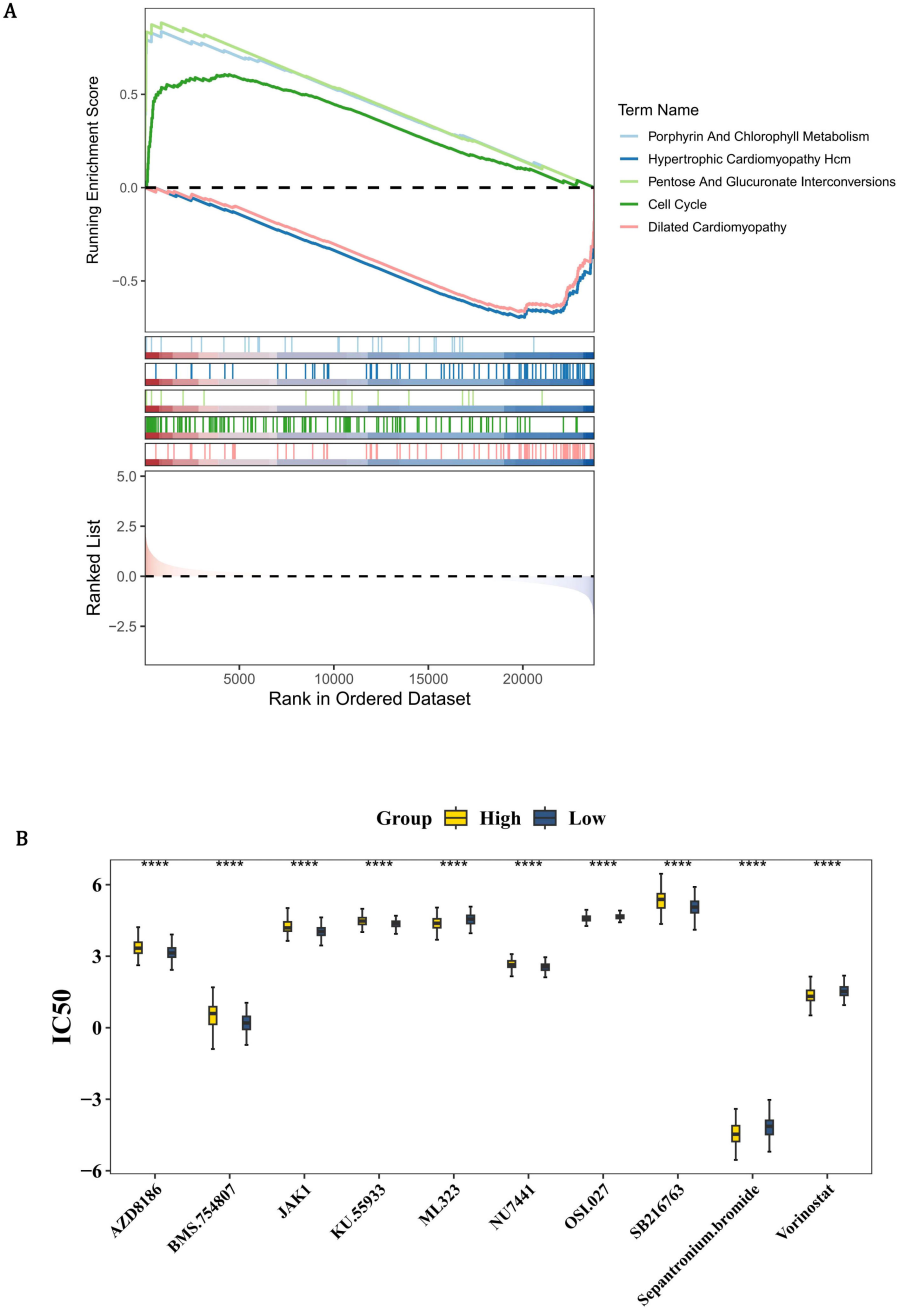
Pathway enrichment and therapeutic vulnerability profiling in PCa risk groups. **(A)** GSEA enrichment plot showing HRG-specific activation of 41 pathways (FDR <0.25), including hypertrophic cardiomyopathy (HCM), porphyrin metabolism, and cell cycle regulation. Colored traces represent pathway-specific enrichment scores, with dashed lines indicating significance thresholds. **(B)** Box plots comparing IC50 values of 86 clinically actionable drugs (Wilcoxon test, P <0.05). Yellow indicates HRG; blue indicates LRG. LRG exhibits heightened AZD8186 sensitivity (HRG median IC50: 6.3 μM vs. LRG: 3.1 μM, P=0.002), while HRG shows preferential ML323 response (HRG: 0.8 μM vs. LRG: 2.4 μM, P=0.0001).

In order to determine the biological pathways involved in the development of PCa between the high and low-risk groups, GSEA enrichment analysis was conducted. By GSEA analysis, the HRG and LRG were strikingly enriched 41 pathways such as hcm, porphyrin and chlorophyll metabolism, pentose and glucuronate interconversions, and cell cycle (**Figure 6A**, **Supplementary Table 10**). These pathways suggested that the risk of PCa might be related to some biological role. In order to obtain molecular targeted drugs corresponding to the genes in the high and low-risk groups, drug sensitivity analysis was conducted. Among 198 drugs, the HRG and LRG suggested notable disparities in sensitivity to 86 drugs (*P* < 0.05) (**Figure 6B**, **Supplementary Table 11**). The IC_50_ value of AZD8186 in the HRG was strikingly higher than that in the LRG, indicating that the LRG was more sensitive to this drug. On the contrary, the IC_50_ value of ML323 in the HRG was remarkably lower than that in the LRG, indicating that the HRG was more responsive to this drug.

### 3.8 The functions and related regulatory factors of prognostic genes

**Figure 7:**
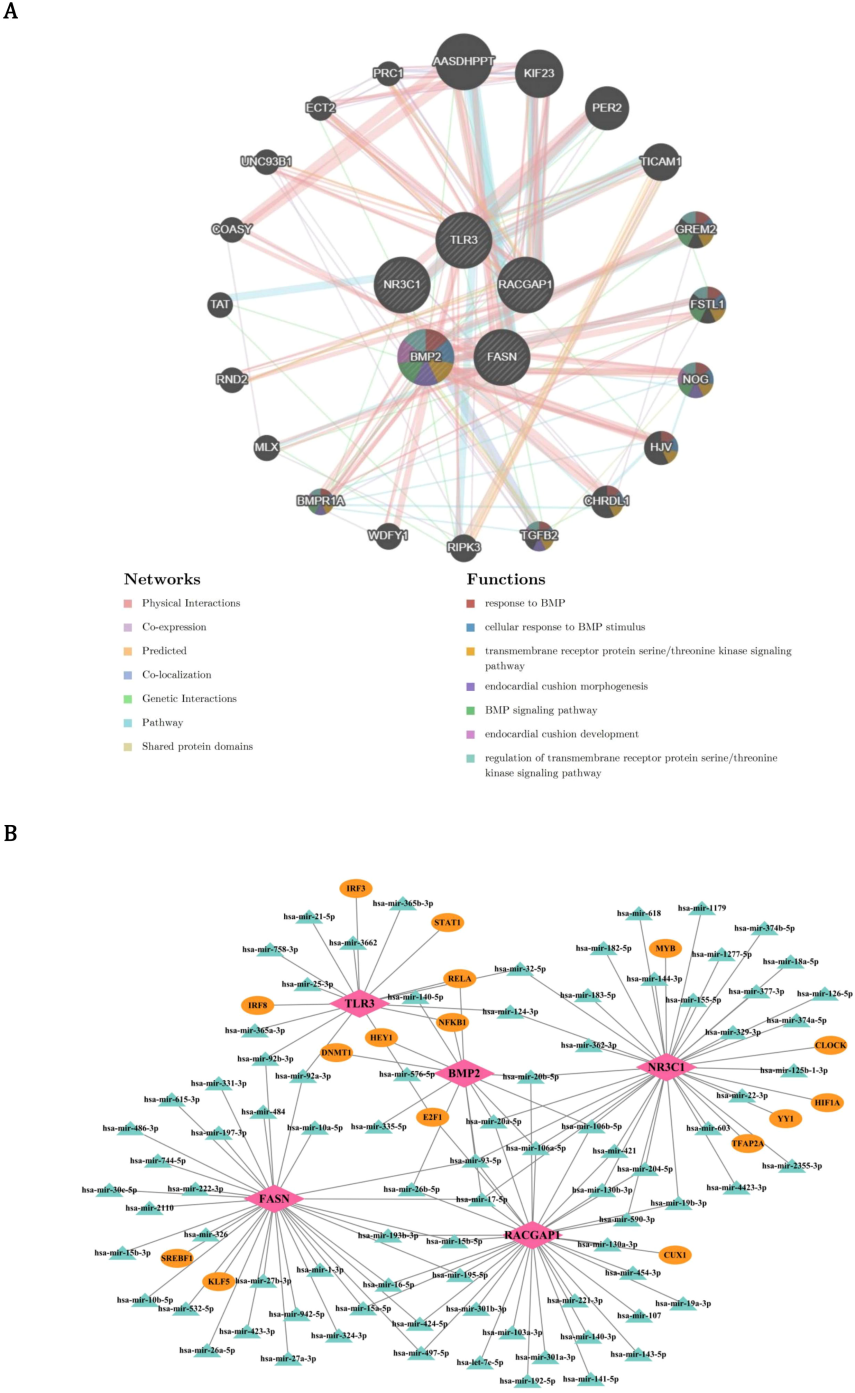
Functional interactome and regulatory networks of PCa prognostic genes. **(A)** GeneMANIA-derived functional interaction network of 20 co-expressed genes (e.g., BMPR1A) enriched in 7 pathways including BMP response (red edges) and cell adhesion (blue edges). Node size reflects interaction degree, with physical interactions (gray edges) and genetic linkages (gold edges) highlighting modular biology. **(B)** Multi-layer regulatory network mapping transcription factors (TFs, pink nodes) and miRNAs (green nodes) targeting core prognostic genes. Circular layout emphasizes combinatorial regulation, with edge thickness proportional to prediction confidence.

In order to identify other genes related to the function of prognostic genes, the GeneMANIA database was used to predict genes associated with the function of prognostic genes and the functions they are involved in. By GeneMANIA analysis, 20 genes related to the functions of prognostic genes were acquired such as BMPR1A and these genes were related to 7 functions such as response to BMP (**Figure 7A**). Furthermore, in order to explore the upstream regulatory factors and their interactions for the prognostic genes, a TF-mRNA-miRNA regulatory network was constructed. Two TFs and 32 miRNAs were predicted for FASN; 5 TFs and 32 miRNAs were predicted for NR3C1; 2 TFs and 32 miRNAs were predicted for RACGAP1; 4 TFs and 10 miRNAs were predicted for BMP2; 6 TFs and 10 miRNAs were predicted for TLR3 (**Figure 7B**). Then, the network was constructed to demonstrate the complex relationships between prognostic genes and regulatory molecules.

### 3.9 Key cells in PCa

**Figure 8:**
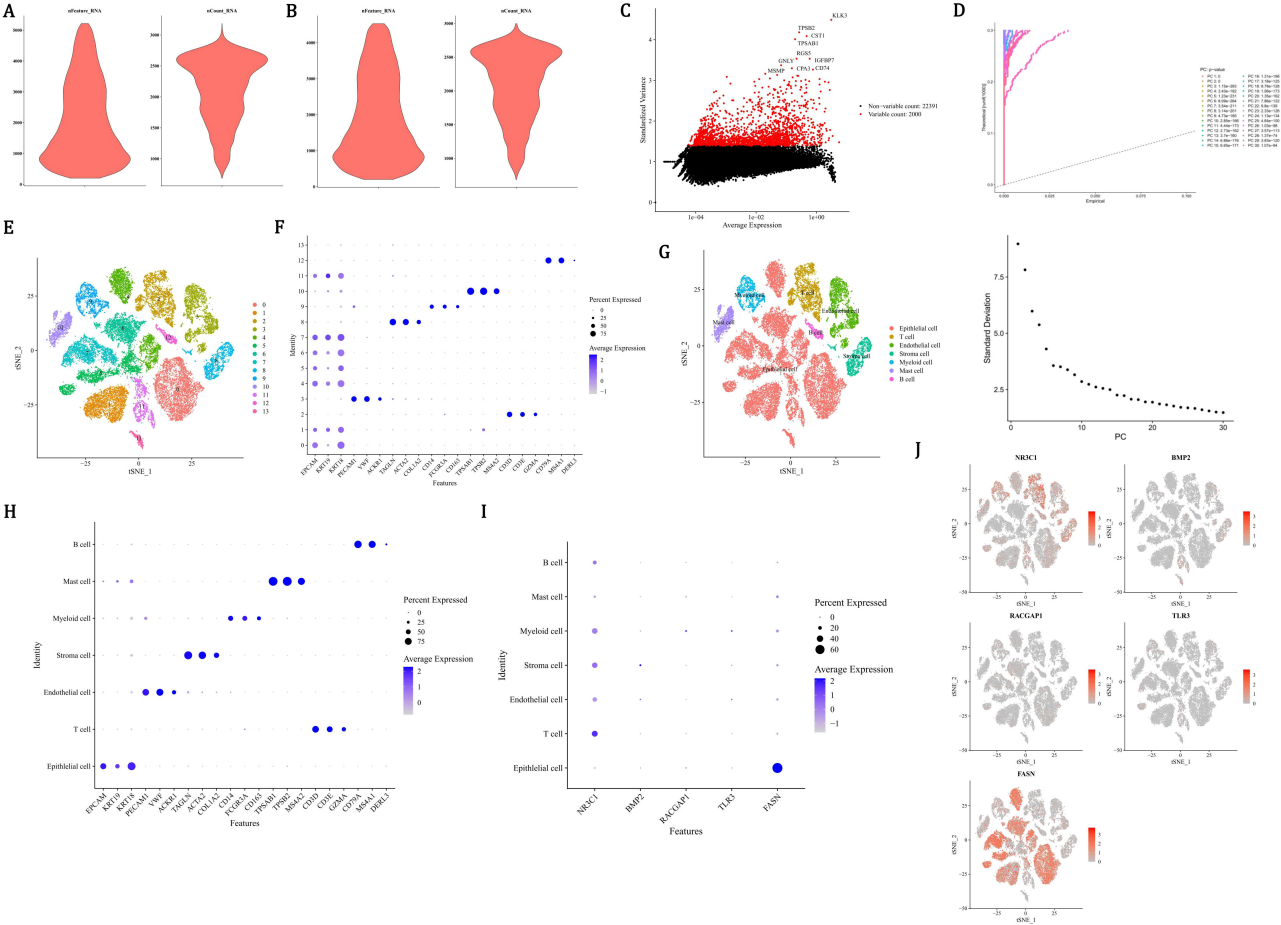
Single-cell transcriptomic profiling identifies epithelial cells as key mediators in PCa progression. **(A, B)** Quality control metrics showing 36,424 cells (24,391 genes) pre-filtering **(A)** and 34,571 cells post-filtering **(B)** with mitochondrial/ribosomal thresholds. **(C)** Top 10 highly variable genes (HVGs) among 2,000 identified, ranked by dispersion. **(D)** Principal component analysis using first 30 principal components (PCs) for dimensionality reduction. **(E)** t-SNE visualization of 14 unsupervised cell clusters. **(F-H)** Cell type annotation using lineage-specific markers: mast (TPSB2), stroma (COL1A2), endothelial (PECAM1), T cells (CD3E), epithelial (KRT18), myeloid (CD14), B cells (MS4A1). **(I)** Violin plots revealing FASN overexpression (red gradient) in epithelial clusters versus other cell types. **(J)** Spatial expression mapping showing NR3C1 enrichment (blue) in T cells and FASN dominance (red) in epithelial compartments.

In order to ensure the accuracy, reliability, and interpretability of the single-cell data, quality control was performed on all scRNA-seq data. There were 24,391 genes and 36,424 cells before quality control (**Figure 8A**), and 24,391 genes and 34,571 cells after quality control (**Figure 8B**). Then, the 2,000 HVGs and the top 10 most varied gene names were displayed (**Figure 8C**). After dimensionality reduction, the first 30 PCs were used for clustering analysis (**Figure 8D**). Finally, the 14 cell clusters were acquired (**Figure 8E**). After annotating cell clusters with markers genes, there were 7 cells acquired, which included mast cells (TPSB2, MS4A2, TPSAB1), stroma cells (COL1A2, TAGLN, ACTA2), endothelial cells (PECAM1, VWF, ACKR1), T cells (GZMA, CD3E, CD3D), epithelial cells (KRT18, EPCAM, KRT19), myeloid cells (CD14, FCGR3A, CD163), and B cells (MS4A1, CD79A, DERL3) (**Figures 8F-H**). Among all the prognostic genes, FASN was prominently expressed in epithelial cells, so epithelial cells were considered as key cells (**Figure 8I**). NR3C1 was more abundantly distributed in T cells, and FASN was more abundantly distributed in epithelial cells (**Figure 8J**).

### 3.10 Communication networks, differentiation of key cells, and expression of prognostic genes in key cells

**Figure 9:**
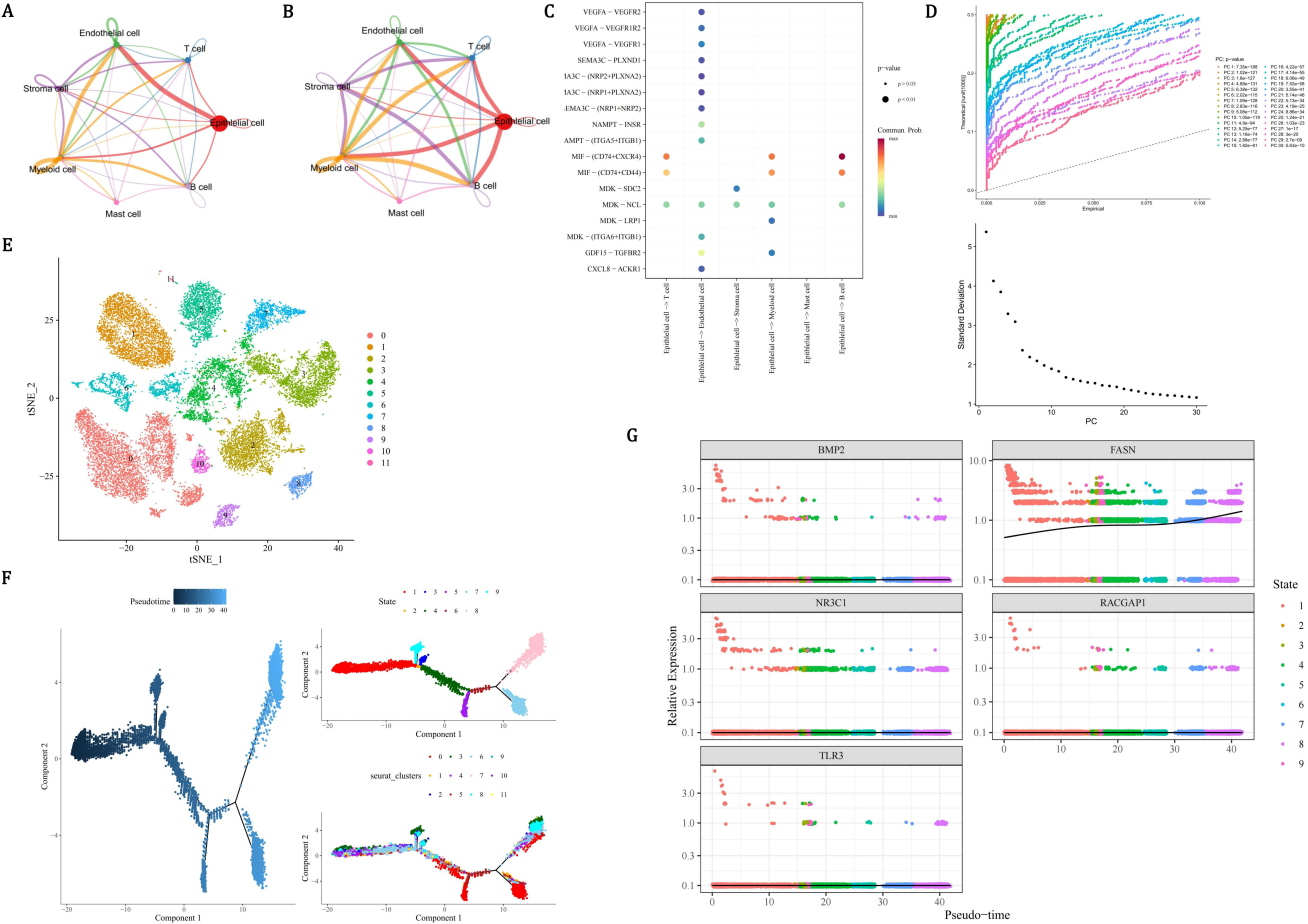
Epithelial cell communication dynamics and FASN-driven progression in PCa. **(A)** Cell-cell interaction network showing dominant endothelial-epithelial crosstalk (edge thickness proportional to interaction frequency). **(B)** Force-directed graph quantifying epithelial cells as central interactors with myeloid, T, and B cells. **(C)** Ligand-receptor validation of epithelial-B cell communication via MIF-(CD74+CXCR4) axis (red: ligand expression; blue: receptor activity). **(D-E)** UMAP visualization of 12 epithelial subclusters **(D)** and pseudo-temporal ordering **(E)** from quiescent (dark blue) to advanced malignant states (light blue). **(F)** Pseudotime trajectory map resolving 9 differentiation stages across 12 subclusters. **(G)** Lineage-specific expression kinetics identifying FASN as the sole prognostic gene with monotonic upregulation, contrasting static patterns of NR3C1/RACGAP1/TLR3/BMP2.

In order to identify the cell types of the samples in the dataset GSE141445 and describe the cellular states of the clustering results, annotations were made for the seven different cell clustering results. Among the annotated cells, the engagements between endothelial cells and epithelial cells were the most frequent (**Figure 9A**, **Supplementary Figure 6A**). Epithelial cells and myeloid cells had the strongest interactions with other cells (**Figure 9B**). Epithelial cells had the strongest interaction with endothelial cells, T cells, myeloid cells, and B cells (**Supplementary Figure 6B**). Interaction between epithelial cells and B cells was carried out by MIF−(CD74+CXCR4) (**Figure 9C**). Through reduction and clustering, epithelial cells were eventually categorized into 12 clusters (**Figures 9D, E**). Further investigation into the developmental trajectory of the key cell, epithelial cell, revealed that epithelial cells varied from dark blue to light blue during differentiation and were categorized into 9 stages and 12 clusters (**Figure 9F**). During the differentiation of epithelial cells, only the expression level of FASN continuously increased, and it remained relatively active throughout the entire cell development stage (**Figure 9G**). In conclusion, the development of PCa might be related to epithelial cells and FASN. Furthermore, the expression patterns of prognostic genes during the pseudotime process of myeloid cells differentiation were demonstrated. As shown in the figure, the FASN and TLR3 genes exhibited higher expression levels at the early stages of myeloid cell differentiation, while RACGAP1 and NR3C1 genes had higher expression at the late stages, and BMP2 gene showed higher expression at the mid-stage of differentiation. These results suggested that these five prognostic genes had elevated expression at specific stages of myeloid cell differentiation, which might have led to their overall expression being less prominent (**Supplementary Figure 7**).

### 3.11 Validation of prognostic gene expression

**Figure 10:**
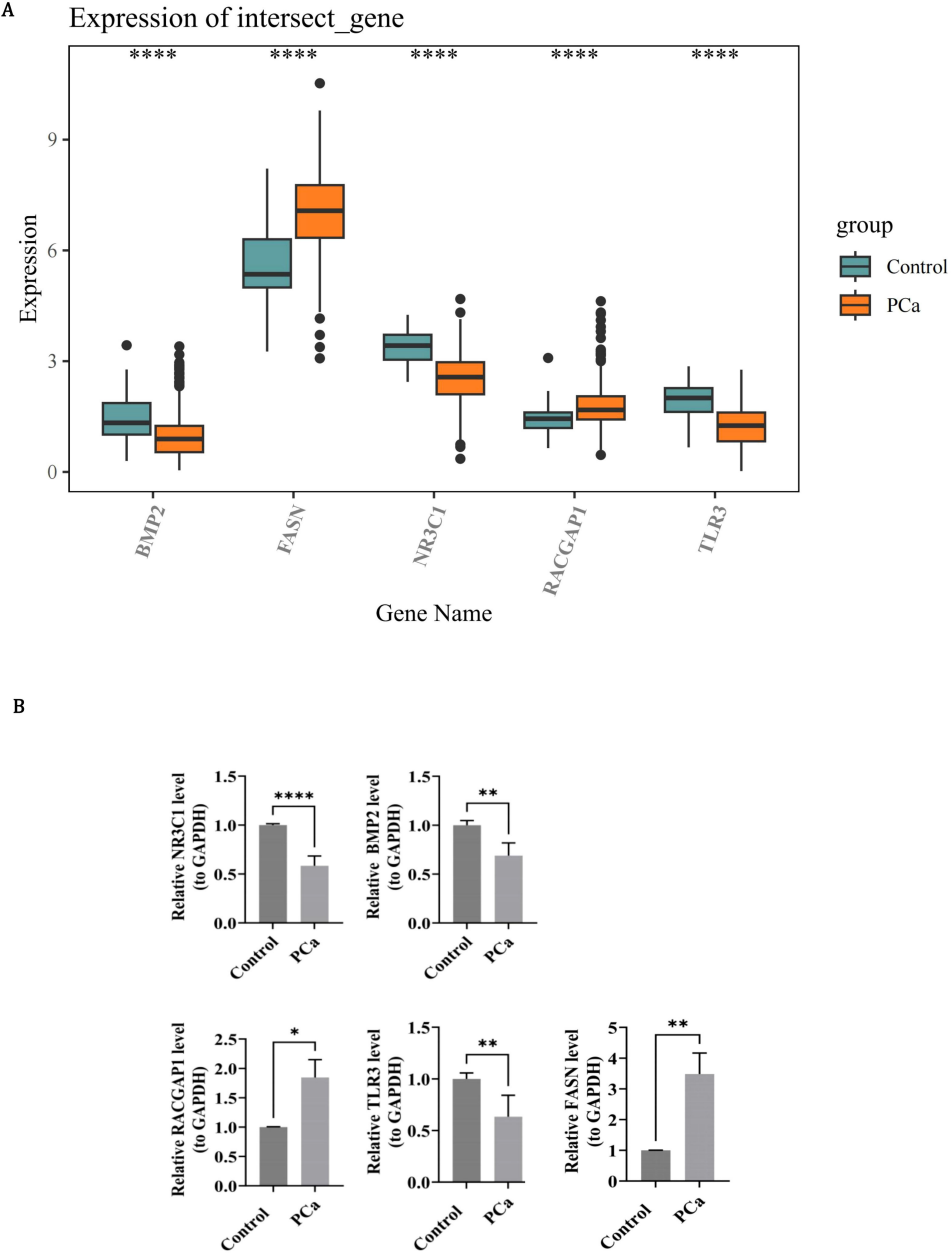
Experimental validation of prognostic gene expression patterns in PCa. **(A)** Box plots of RNA-seq expression levels in control (teal) and PCa (orange) groups. Whiskers extend to 1.5×IQR; dots indicate outliers. **(B)** RT-qPCR confirmation using 15 paired PCa/control specimens. Bar graphs quantify concordant expression trends.

In order to determine the expression patterns of prognostic genes in PCa samples and control samples, expression analysis was conducted using the dataset and RT-qPCR experiments. The expression levels of BMP2, NR3C1 and TLR3 were all remarkably lower in PCa samples than in the control samples (*P* < 0.0001), while the expression levels of RACGAP1 and FASN were remarkably higher in PCa samples than in the control samples (*P* < 0.0001) (**Figure 10A**). In RT-qPCR, the expression levels of NR3C1 (*P* < 0.0001), BMP2 (*P* < 0.01) and TLR3 (*P* < 0.01) in PCa were significantly lower than those in control group, while the expression levels of RACGAP1 (*P* < 0.05) and FASN (*P* < 0.01) in PCa were significantly higher than those in control group (**Figure 10B**). The expression level and trend of prognostic genes *in vitro* samples were consistent with that of bioinformatics analysis, indicating the reliability of bioinformatics analysis results. Previous studies have highlighted the significance of BMP2 and FASN in PCa progression. Horvath et al. demonstrated that reduced BMP2 expression is associated with PCa progression, linking its loss to more aggressive phenotypes (52). Similarly, Tae et al. reported that decreased BMP2 expression correlates with a higher incidence of biochemical recurrence (BCR) and elevated Gleason scores (GS) (53). However, the precise mechanisms underlying BMP2’ s role as a tumor outcome determinant remain elusive. In contrast, the biological role of FASN as a key regulator of lipid metabolism is well-established. By driving lipid synthesis, FASN provides energy to fuel tumor proliferation and progression. Despite this, its specific regulatory effects within the PCa microenvironment are not fully understood. Chianese et al. observed FASN overexpression in PCa and proposed that FASN inhibition disrupts the metabolic axis, leading to lipid accumulation and subsequent lipotoxicity (54, 55). This metabolic dysregulation impairs replication mechanisms and arrests cells in the G0/G1 phase, thereby inhibiting proliferation (56, 57). These findings underscore the multifaceted roles of BMP2 and FASN in PCa biology and warrant further investigation into their underlying mechanisms and therapeutic potential.

### 3.12 Overexpression of BMP2 or silencing of FASN suppresses malignant behaviors of PCa cells

**Figure 11:**
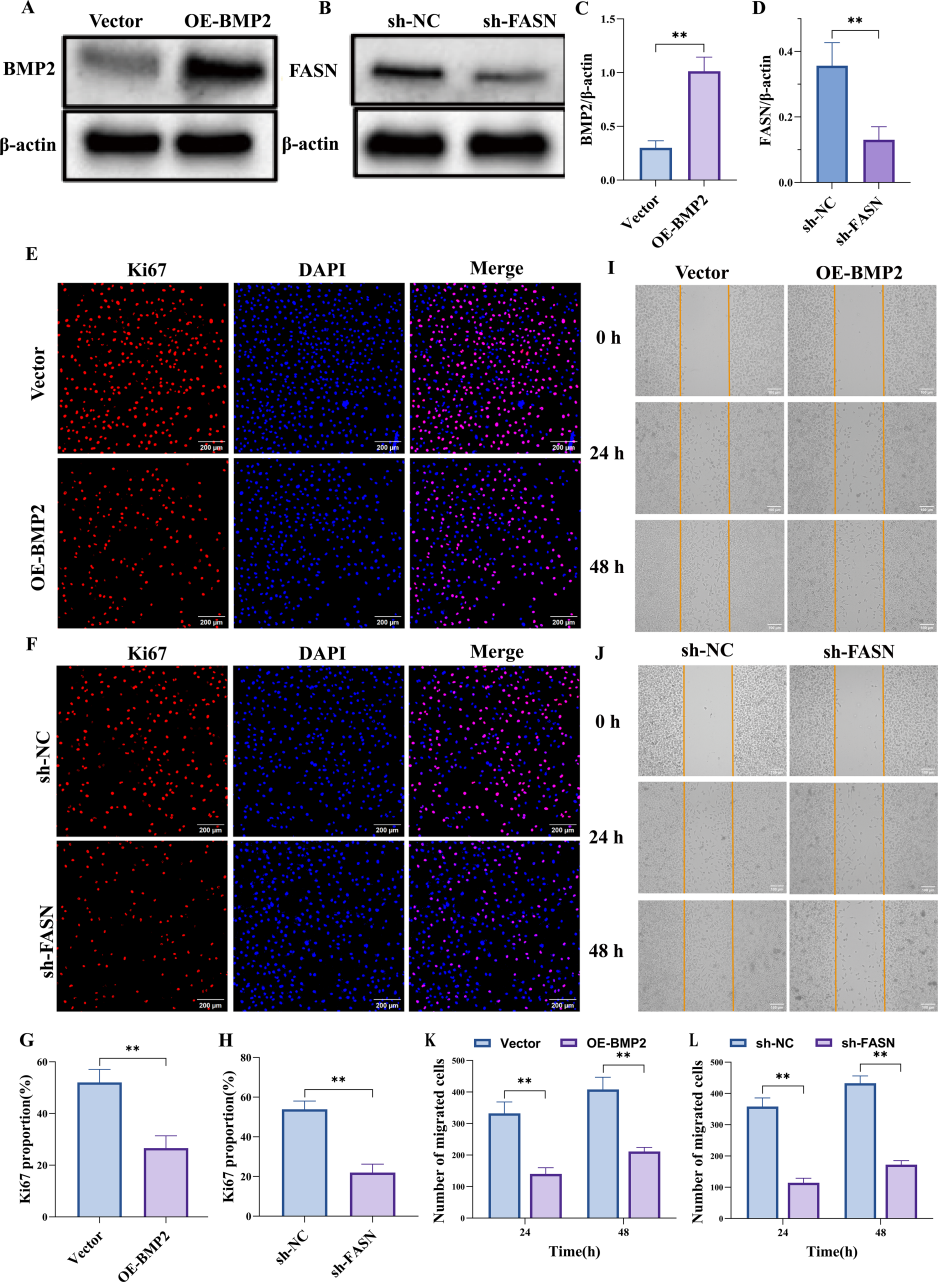
Overexpression of BMP2 or silencing of FASN suppresses malignant behaviors of PC3 cells. **(A–D)** Western blot results confirm that BMP2 overexpression effectively increases BMP2 protein levels in PC3 cells, whereas FASN knockdown effectively reduces FASN protein levels. **(E–H)** Ki67 immunostaining shows that BMP2 overexpression or FASN knockdown both suppress PC3 cell proliferative activity. **(I–L)** Scratch-wound assay indicate that BMP2 overexpression or FASN knockdown significantly reduces the migratory capacity of PC3 cells. Data are presented as mean ± SEM, n=3. **p < 0.01.

Western blot analysis revealed that compared with the Vector group, the protein level of BMP2 was significantly upregulated in the OE-BMP2 group, while the FASN protein level showed no obvious change; conversely, in the sh-FASN group, the FASN protein level was notably downregulated compared with the sh-NC group, with no significant difference in BMP2 level (**Figures 11A-D**). Immunofluorescence staining demonstrated that the proportion of Ki67-positive cells was significantly higher in the OE-BMP2 group than in the Vector group (**Figures 11E, G**), and lower in the sh-FASN group than in the sh-NC group (**Figures 11F, H**), as quantified by statistical analysis. In the cell migration assay, the number of migrated cells in the OE-BMP2 group was significantly greater than that in the Vector group at both 24 h and 48 h time points, whereas the sh-FASN group showed a significantly reduced number of migrated cells compared with the sh-NC group at the same time points, as shown by the quantitative results (**Figures 11I-L**).

## 4 Discussion

PCa remains a leading contributor to global cancer-related morbidity and mortality among males, with rising incidence rates documented in recent epidemiological studies (58). The clinical management of PCa faces significant challenges due to the lack of discernible clinical manifestations in early-stage disease, resulting in delayed diagnosis for the majority of patients until intermediate/advanced phases or metastatic progression, which is the critical factor driving elevated mortality (59). These clinical realities underscore the urgent need to develop novel therapeutic strategies and personalized treatment paradigms to improve patient outcomes. In this study, we integrated transcriptomic profiling and scRNA-seq data to systematically identify MCDRGs with causal associations in PCa pathogenesis. A predictive risk model was developed and rigorously validated to assess the clinical utility of these biomarkers. Through comprehensive bioinformatic interrogation, we elucidated the mechanistic contributions of candidate genes to PCa progression, complemented by single-cell resolution analysis of their expression dynamics across tumor-associated cellular subpopulations. The computational findings were further substantiated through *in vitro* functional validation, confirming the biological relevance of identified molecular networks.

NR3C1 (Nuclear Receptor Subfamily 3 Group C Member 1), located at chromosome 5q31.3-q32, encodes the glucocorticoid receptor (GR), the sole mediator of glucocorticoid signaling through ligand-activated transcriptional regulation within the nuclear receptor superfamily (60). This receptor-ligand complex translocates to the nucleus, binding specific DNA response elements to orchestrate diverse physiological processes including glucose/lipid metabolism, inflammatory responses, and cellular differentiation (60, 61). Emerging evidence positions NR3C1 as a pivotal oncogenic regulator across malignancies: it drives progression in triple-negative breast cancer, ovarian carcinoma, urothelial cancer, and clear cell renal carcinoma (61–65), while mediating platinum and targeted therapy resistance in lung and ovarian cancers (66, 67). In PCa, a dynamic AR-NR3C1 axis governs therapeutic resistance. Androgen receptor (AR) suppresses NR3C1 expression in treatment-naïve states, whereas androgen deprivation therapy induces compensatory GR upregulation—a critical mechanism enabling treatment evasion through AR-GR crosstalk (68, 69). Mechanistically, Qian et al. delineated the role of NR3C1 in PCa lineage plasticity, demonstrating that the ONECUT2 (OC2) transcription factor activates NR3C1 and neuroendocrine splicing factor SRRM4 to drive adenocarcinoma progression and therapy-resistant stem-like/neuroendocrine variants (70).

BMP2 (Bone Morphogenetic Protein 2), a key TGF-β superfamily member located at chromosome 20p12, encodes a multifunctional regulator of cellular processes including proliferation, differentiation, migration, and apoptosis, though it remains best characterized for its osteoinductive role in skeletal development (71). During embryogenesis, BMP2 drives osteogenic differentiation of mesenchymal stem cells by stimulating extracellular matrix production (collagen, osteocalcin) and subsequent bone mineralization (72, 73). Beyond developmental biology, BMP2 exhibits remarkable therapeutic potential in fracture repair through site-specific upregulation that recruits progenitor cells and accelerates osteogenesis (74). The pleiotropic nature of BMP2 signaling manifests through context-dependent tumor modulation. While suppressing metastasis in breast cancer (75, 76) and inhibiting proliferation/biochemical recurrence in PCa (53, 77), its aberrant activation paradoxically enhances hepatocellular carcinoma progression via proliferative and invasive mechanisms (78, 79). Moreover, recent evidence extends the functional repertoire of BMP2 to fibroblast biology, demonstrating anti-inflammatory properties that mitigate atrial fibrosis (80).

RACGAP1 (Rac GTPase-Activating Protein 1), a critical regulator of cellular dynamics, encodes a member of the GTPase-activating protein (GAP) family that modulates Rac GTPase activity to control cytoskeletal reorganization and mitotic fidelity (81). During cell division, RACGAP1 ensures genomic stability through its indispensable role in spindle assembly and chromosome segregation (82, 83). Emerging oncogenic roles of RACGAP1 span multiple malignancies. Its dysregulated expression correlates with aggressive phenotypes in lung adenocarcinoma and bladder cancer, where it drives tumor progression via enhanced proliferation, invasion, and metastatic dissemination. In hepatocellular carcinoma, RACGAP1 emerges as both an independent prognostic biomarker and a modulator of tumor immune microenvironment (84–86). Notably, RACGAP1 intersects with therapeutic resistance pathways in PCa. Mechanistic studies reveal its capacity to activate downstream effectors of the PI3K/AKT axis, a compensatory signaling network implicated in ADT resistance and neuroendocrine differentiation (87). Clinical validation through qPCR analysis confirms RACGAP1 overexpression in castration-resistant PCa (CRPC), underscoring its functional relevance in treatment-refractory disease (88).

TLR3 (Toll-like Receptor 3), located at chromosome 4q35.1, encodes a pattern recognition receptor predominantly expressed on immune cells including dendritic cells, NK cells, and macrophages (89). This receptor initiates antiviral immunity through specific recognition of viral double-stranded RNA (dsRNA), triggering signal transduction cascades that activate NF-κB and induce interferon/cytokine production (90). Emerging evidence reveals context-dependent prognostic implications of TLR3 dysregulation across malignancies, low TLR3 expression correlates with favorable outcomes in gastric, prostate, and breast cancers, yet paradoxically associates with poor prognosis in clear cell renal carcinoma and hepatocellular carcinoma (91–93). Muresan et al. analyzed hormone-naïve and hormone-resistant PCa specimens, demonstrating TLR3 upregulation in therapy-refractory tumors alongside its functional role in promoting migratory and invasive capacities (94). These findings align with prior clinical observations documenting TLR3’s association with aggressive PCa behavior (95–97). Intriguingly, comparative studies reveal tissue-specific expression patterns, While González-Reyes et al. reported elevated TLR3 levels in PCa versus benign prostate tissue (97), our data paradoxically demonstrate significant TLR3 downregulation in tumor versus adjacent paracancerous tissues, which correlates with changes in the tumor microenvironment and immune evasion mechanisms, suggests that TLR3 suppression may play a critical role in the malignant transformation of prostate epithelium. As a critical bridge between innate and adaptive immunity, TLR3 plays a pivotal role in anti-tumor immunity (98). Extensive research has demonstrated that TLR3 can directly activate tumor-specific NK cells or mediate the release of interferon to enhance cytotoxic lymphocyte (CTL) infiltration and response, establish type 1 T helper cells (Th1) immunity, and upregulate genes involved in the recruitment and functionality of immune cells within the tumor microenvironment (99, 100). Notably, TLR3 executes its functions through diverse immune pathways. However, its expression at the tissue or organismal level may exhibit ectopic distribution, such as variations in subcellular localization (e.g., cytoplasmic versus membrane-bound) or differential expression between cell types (101, 102). Importantly, the functional impact of TLR3 cannot be comprehensively inferred from its overall expression levels alone, as shifts in cellular composition or differential expression across specific cell types may obscure its true immunological significance under various conditions. These complexities underscore the need for a nuanced exploration of the role of TLR3 in tumor immunity.

FASN (Fatty Acid Synthase), the rate-limiting enzyme catalyzing the final step of *de novo* fatty acid synthesis, is ubiquitously expressed with elevated activity in lipid-metabolizing organs including liver, adipose tissue, and mammary glands (103, 104). This multienzyme complex mediates the NADPH-dependent condensation of acetyl-CoA and malonyl-CoA to generate palmitate, the primary substrate for membrane phospholipid synthesis, energy storage, and bioactive lipid precursors (104). Under physiological conditions, FASN activity is tightly regulated, with endogenous synthesis suppressed under nutrient-replete conditions via insulin-mediated regulation to prioritize dietary fatty acid utilization. In oncogenic contexts, FASN undergoes pathological upregulation to fuel tumorigenic demands. Elevated FASN expression drives *de novo* lipogenesis, fulfilling the biosynthetic requirements of rapidly proliferating cancer cells for membrane remodeling and signaling lipid generation (105, 106). Beyond its role in lipid synthesis, FASN promotes lipid accumulation within tumor cells, which may disrupt antigen presentation or alter surface molecule profiles, thereby impairing the immune system’ s ability to recognize these cells (107, 108). Moreover, FASN-mediated metabolic reprogramming reshapes the tumor microenvironment through lipid-driven immunosuppression, where increased FASN activity inversely correlates with antitumor immune cell infiltration across multiple malignancies (109, 110). These findings position FASN as a compelling therapeutic target, with pharmacological inhibition strategies showing promise for disrupting cancer-specific lipogenic dependencies.

Our investigation revealed 18 differentially abundant immune cell populations between LRG and HRG in the PCa microenvironment, with NK cells demonstrating the most significant correlations with prognostic gene signatures. Unlike T cells requiring antigen-specific MHC recognition, NK cells execute innate immunosurveillance through non-antigen-directed cytotoxicity against malignant cells. Notably, NK cells constitute 2-9% of tumor-infiltrating lymphocytes in prostate carcinoma, underscoring their microenvironmental relevance (111). Gannon et al. reported reduced biochemical recurrence rates in treatment-naïve patients exhibiting elevated intraprostatic NK cell infiltration (112). Complementary studies by Pasero et al. linked high surface NKG2D expression on tumor-associated NK cells with attenuated disease progression (113). Mechanistically, Lundholm et al. identified tumor-derived exosomal NKG2D ligands as immunosuppressive agents that downregulate NK cell activation via receptor internalization—a plausible immune evasion mechanism (114). Multidimensional profiling by Zorko et al. further delineated NK cell-mediated clinical benefits, enhanced NK infiltration inversely correlated with driver mutations (e.g., AR-V7 variants) in primary tumors while positively associating with immunosuppressive checkpoints, suggesting dual roles in tumor editing and microenvironment modulation (111). These findings collectively nominate NK cell potentiation strategies—including endogenous activity enhancement and adoptive cell therapies—as promising therapeutic frontiers for CRPC. Additionally, in tumor immunotherapy, mitochondrial function in NK cells directly impacts their activity, survival, and antitumor capacity (115, 116). Thus, optimizing mitochondrial health in NK cells may emerge as a potential strategy to enhance therapeutic efficacy.

Our analysis identified clinically divergent drug sensitivities between HRG and LRG, including AZD8186 and JAK1. AZD8186, a potent selective PI3Kβ inhibitor, targets the oncogenic PI3K signaling axis implicated in tumor cell proliferation, metabolic adaptation, and angiogenesis (117–119). The first-in-human trial (NCT01884285) established its manageable safety profile and preliminary efficacy (120). Preclinically, Ruiz et al. demonstrated synergistic antitumor activity of AZD8186 combined with selumetinib in docetaxel-resistant murine models without additive toxicity, highlighting its therapeutic potential for taxane-refractory prostate cancer (121). Mechanistic insights into chemoresistance emerged from single-cell profiling of docetaxel-resistant tumors by Cheng et al. revealing IL-11 overexpression that activates the JAK1/STAT4 axis. This cascade facilitates STAT4-CBP complex formation, driving c-MYC transcription—a well-characterized oncogene promoting tumorigenesis and therapy resistance (122–124). IL-11 further orchestrates a chemoresistant niche via autocrine tumor cell signaling and paracrine stromal interactions involving extracellular matrix remodeling (125, 126). Therapeutic opportunities​lie in disrupting this axis through JAK1 inhibition, IL-11 neutralization, or STAT4-CBP interface targeting. Future work should employ advanced humanized models recapitulating tumor-immune-stroma crosstalk to validate these strategies and delineate microenvironmental influences on drug response.

GeneMANIA analysis identified 20 functionally interconnected genes associated with prognostic signatures, including BMPR1A—a pivotal receptor mediating BMP signaling through ligand binding and pathway activation (127). Yang et al. mechanistically demonstrated that GALNT12 enhances BMPR1A O-glycosylation to suppress metastatic PCa cell proliferation, migration, and invasion, nominating GALNT12 as a therapeutic target (128). Notably, several network components like GREM2 and FSTL1 remain underexplored in PCa contexts, warranting further investigation of their therapeutic potential. The reconstructed regulatory network revealed transcription factors with established oncogenic roles. Yin Yang 1 (YY1), a C2H2 zinc finger transcriptional regulator implicated in tumor-associated immune suppression, was shown to drive IL-6 production in M2-polarized macrophages while inhibiting anti-tumor T-cell activity—a mechanism suggesting YY1-targeted immunomodulation strategies (14, 129, 130). Furthermore, CUX1, a homeodomain transcription factor governing development and cell cycle progression (131), emerged as a network hub differentially regulated during ADT. Sharma et al. identified CUX1 as a key transcriptional regulator through whole-transcriptome profiling of pre-/post-ADT specimens (132), while Dorris et al. reported paradoxical effects: CUX1 knockdown enhanced migration in androgen-sensitive cells but increased invasion in castration-resistant lineages (133). These context-dependent phenotypes underscore the need for mechanistic elucidation of CUX1’s dual roles in PCa progression.

Epithelial cells, integral components of innate immune surveillance and tissue barrier defense (134, 135), emerge as pivotal mediators of PCa progression through multifaceted mechanisms. In this study, the results form scRNA-seq analyze revealed three interconnected oncogenic roles: (1) Prognostic gene FASN exhibits sustained overexpression in malignant epithelia, directly driving tumorigenic behaviors; (2) Epithelial cells orchestrate a pro-tumorigenic niche via crosstalk with endothelial and immune compartments; (3) Persistent FASN activation during epithelial differentiation suggests dynamic involvement in malignant transformation. These findings align with growing interest in PCa immunotherapy, though its unique immunosuppressive microenvironment poses translational challenges (136–138). Zhu et al. pioneered an epithelial cell marker gene prognostic signature (ECMGPS) derived from scRNA-seq data (GSE176031), demonstrating robust predictive accuracy for immunotherapy responses despite lacking clinical cohort validation (139). Mechanistic insights from Jiang et al. established its capacity to induce epithelial-mesenchymal transition (EMT) via transcriptional repression of E-cadherin and N-cadherin activation, facilitating peritoneal metastasis in ovarian cancer (140). To date, no studies have mechanistically delineated FASN-epithelial cell interactions in PCa. Our study addresses this critical knowledge gap by providing the first functional evidence linking FASN activity to epithelial cell-mediated oncogenic progression in PCa pathogenesis.

Experimental validation using RT-qPCR confirmed the consistency between bioinformatic predictions and experimental findings, with dysregulated expression of BMP2, NR3C1, TLR3, RACGAP1, and FASN closely associated with PCa progression. Specifically, BMP2 was found to suppress tumor cell proliferation and migration, as evidenced by downregulation of Ki67 expression and reduced migratory activity in PC3 cells. Conversely, FASN promoted both proliferation and migration, underscoring its role as a driver of tumor aggressiveness. These findings highlight the dual regulatory roles of BMP2 and FASN in PCa pathobiology, suggesting their potential as both biomarkers and therapeutic targets. The observed concordance between bioinformatic analysis and experimental validation strengthens the reliability of the identified prognostic genes. Furthermore, the mechanistic insights into BMP2 and FASN’s opposing effects on proliferation and migration provide a foundation for the development of targeted therapeutic strategies. Future studies should prioritize mechanistic investigation of these biomarkers, including development of targeted agents, analysis of stage-specific expression dynamics, and optimization of combinatorial therapeutic strategies. Such efforts will advance precision oncology frameworks to address unmet clinical needs in PCa management.

## 5 Conclusions

This study innovatively integrated MR with transcriptomic and scRNA-seq analyses to identify five prognostic genes associated with PCa progression, subsequently constructing a risk stratification model and evaluating its clinical utility. Mechanistic exploration revealed functional roles of these genes in tumorigenic pathways, complemented by scRNA-seq analysis of their expression patterns across PCa-associated cellular subpopulations. While these findings advance our understanding of PCa biology, several limitations warrant consideration. First, the prognostic model relied solely on a single dataset without external validation cohorts, potentially limiting its generalizability. Second, experimental validation focused primarily on preliminary functional assays, necessitating further exploration of the molecular mechanisms underlying BMP2 and FASN-mediated effects. Despite these constraints, our study establishes a robust theoretical foundation for the development of personalized therapeutic strategies in PCa management. By identifying and characterizing key prognostic genes, we provide a comprehensive framework for advancing precision oncology and addressing unmet clinical needs in PCa prognosis and treatment.

## Data Availability

The original contributions presented in the study are included in the article/**Supplementary Material**. Further inquiries can be directed to the corresponding author.
