# Supplementary material for: Mendelian randomization combined with single-cell sequencing analysis revealed prognostic genes related to myeloid cell differentiation in prostate cancer and experimental verification
Source: Front Immunol. 2025 Sep 23;16:1619194. doi: 10.3389/fimmu.2025.1619194 (PMC12500568; doi:10.3389/fimmu.2025.1619194)
Supplement: Supplementary file 1 [file DataSheet1.zip › Supplementary Figure 1/FLNA.pdf]

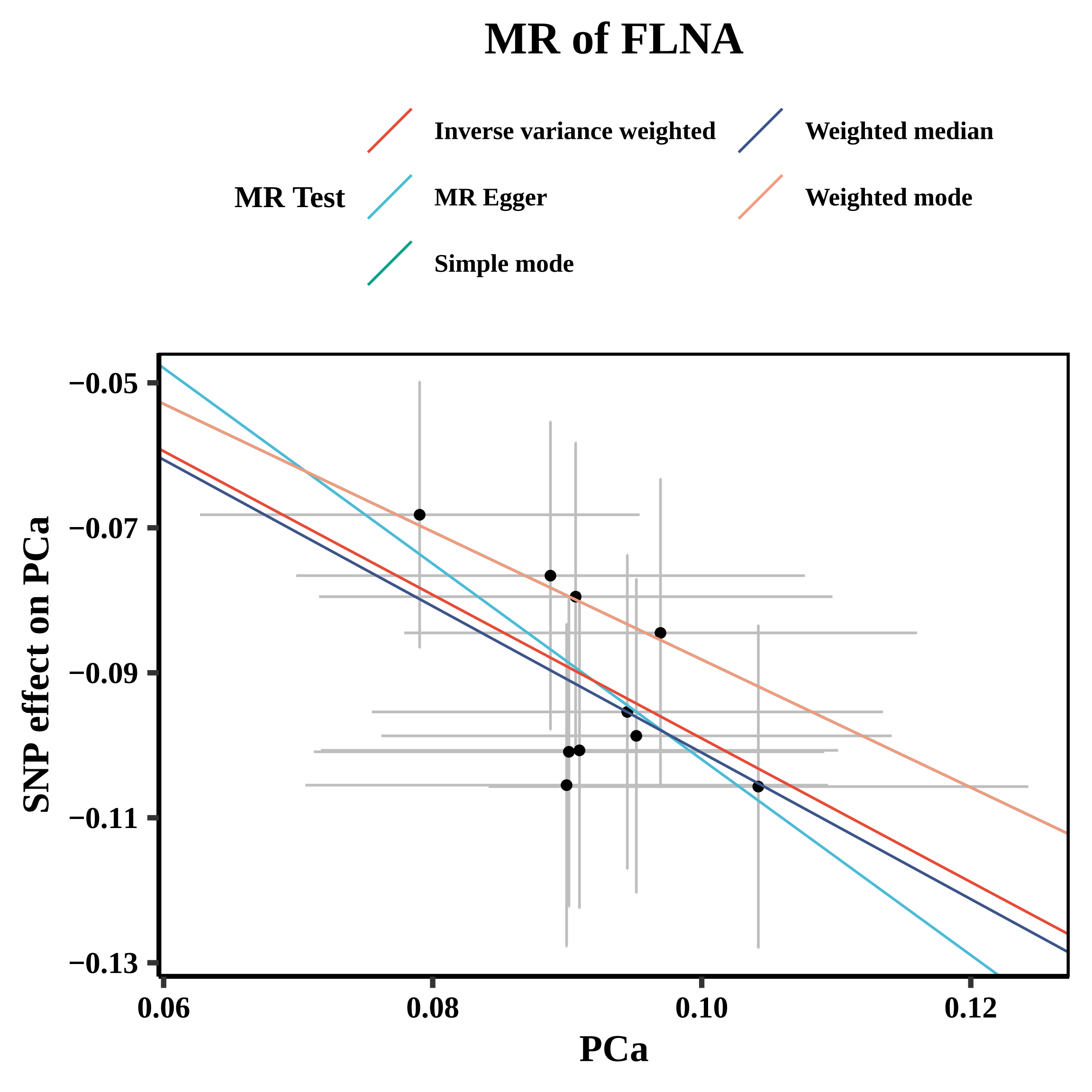
MR of FLNA
MR Test
Inverse variance weighted
MR Egger
Simple mode
Weighted median
Weighted mode
SNP effect on PCa
−0.05
−0.07
−0.09
−0.11
−0.13
0.06
0.08
0.10
0.12
PCa
