# Supplementary material for: Mendelian randomization combined with single-cell sequencing analysis revealed prognostic genes related to myeloid cell differentiation in prostate cancer and experimental verification
Source: Front Immunol. 2025 Sep 23;16:1619194. doi: 10.3389/fimmu.2025.1619194 (PMC12500568; doi:10.3389/fimmu.2025.1619194)
Supplement: Supplementary file 1 [file DataSheet1.zip › Supplementary Figure 1/GATA3.pdf]

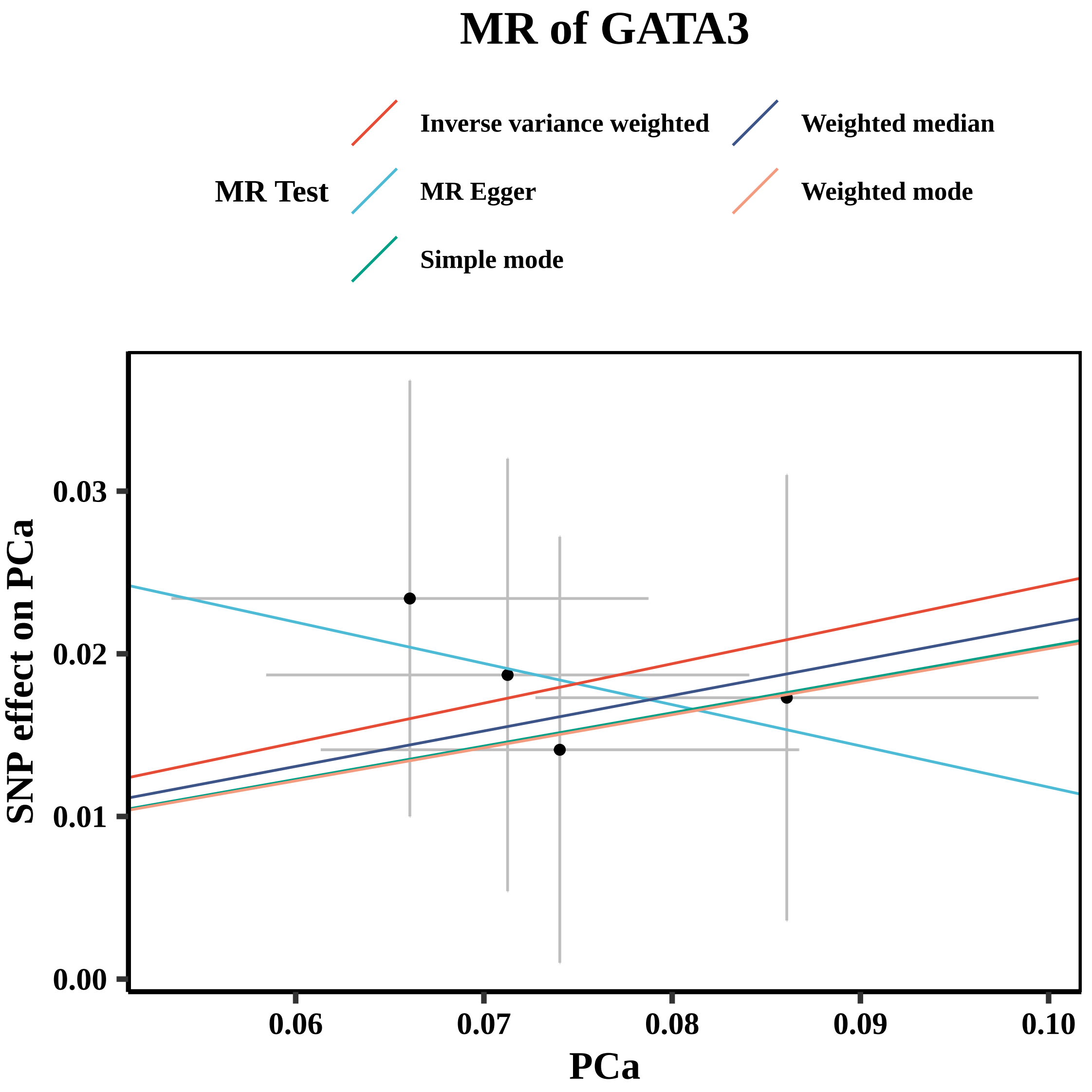
MR of GATA3
MR Test
Inverse variance weighted
Weighted median
MR Egger
Weighted mode
Simple mode
SNP effect on PCa
0.00
0.01
0.02
0.03
0.06
0.07
0.08
0.09
0.10
PCa
