# Supplementary material for: Mendelian randomization combined with single-cell sequencing analysis revealed prognostic genes related to myeloid cell differentiation in prostate cancer and experimental verification
Source: Front Immunol. 2025 Sep 23;16:1619194. doi: 10.3389/fimmu.2025.1619194 (PMC12500568; doi:10.3389/fimmu.2025.1619194)
Supplement: Supplementary file 1 [file DataSheet1.zip › Supplementary Figure 1/MITF.pdf]

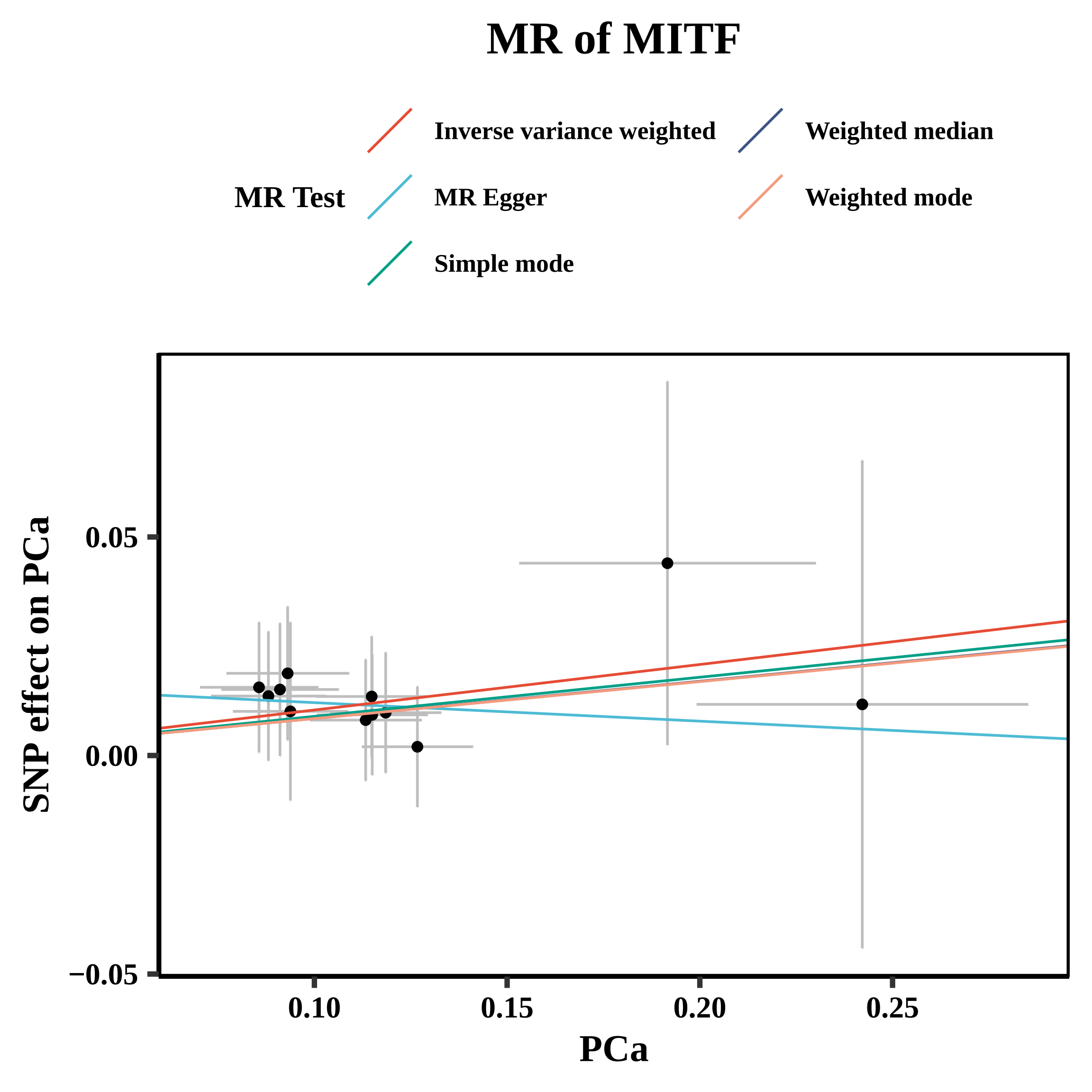
MR of MITF
MR Test
Inverse variance weighted
MR Egger
Simple mode
Weighted median
Weighted mode
SNP effect on PCa
0.05
0.00
−0.05
0.10
0.15
0.20
0.25
PCa
