# Supplementary figures and images for: Mendelian randomization combined with single-cell sequencing analysis revealed prognostic genes related to myeloid cell differentiation in prostate cancer and experimental verification
Source: Front Immunol. 2025 Sep 23;16:1619194. doi: 10.3389/fimmu.2025.1619194 (PMC12500568; doi:10.3389/fimmu.2025.1619194)

# MR of ACTN1

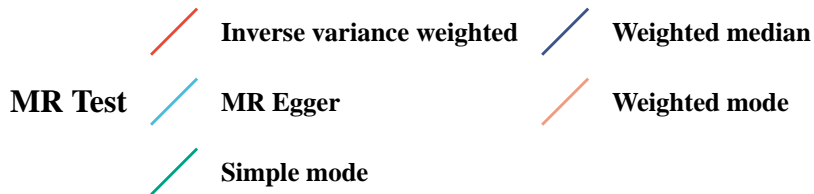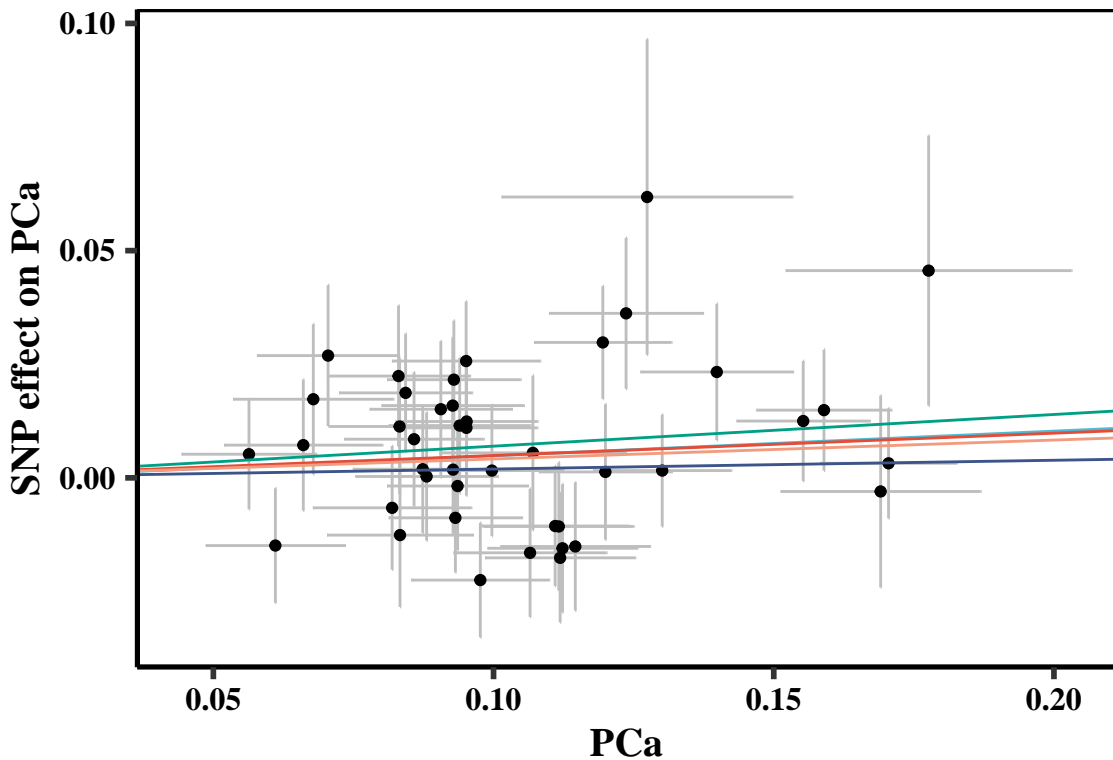

Supplement: Supplementary file 1 [file DataSheet1.zip › Supplementary Figure 1/ACTN1.pdf]

# MR of BATF3

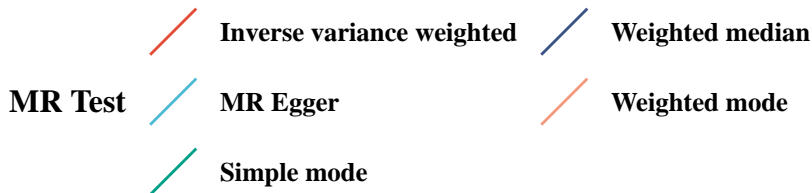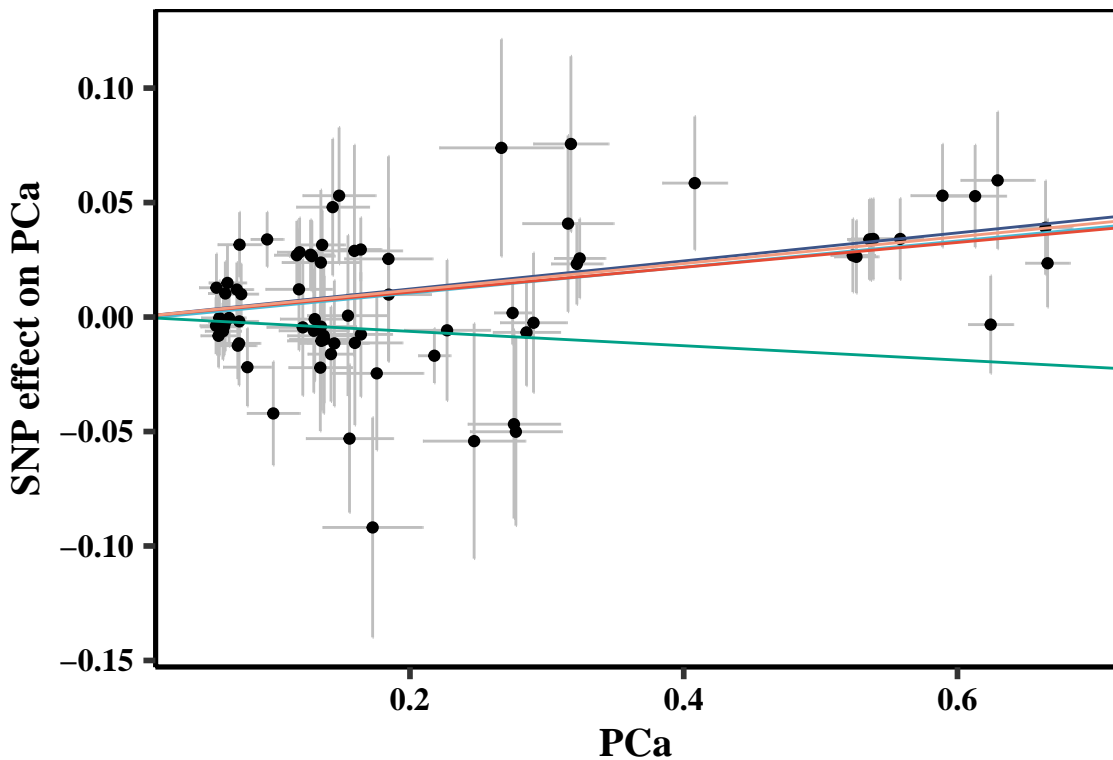

Supplement: Supplementary file 1 [file DataSheet1.zip › Supplementary Figure 1/BATF3.pdf]

# MR of BMP2

MR Test

Inverse variance weighted

Weighted median

MR Egger

Weighted mode

Simple mode

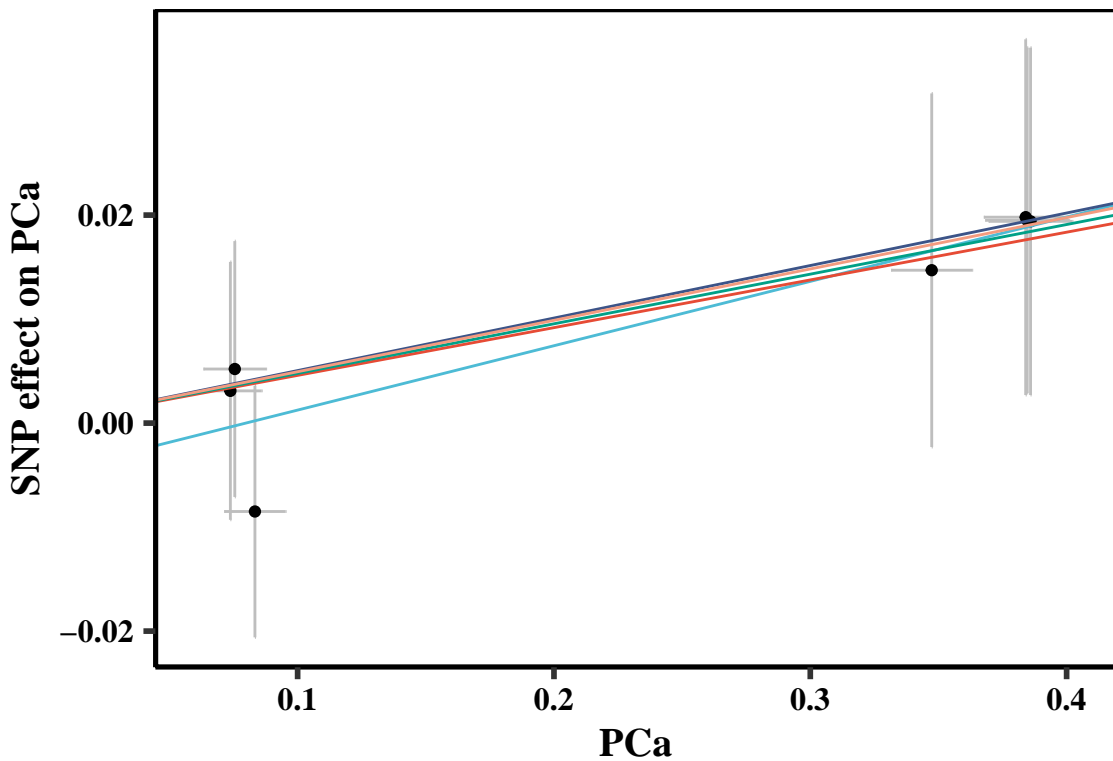

Supplement: Supplementary file 1 [file DataSheet1.zip › Supplementary Figure 1/BMP2.pdf]

# MR of CD109

MR Test

- Inverse variance weighted
- Weighted median
- MR Egger
- Weighted mode
- Simple mode

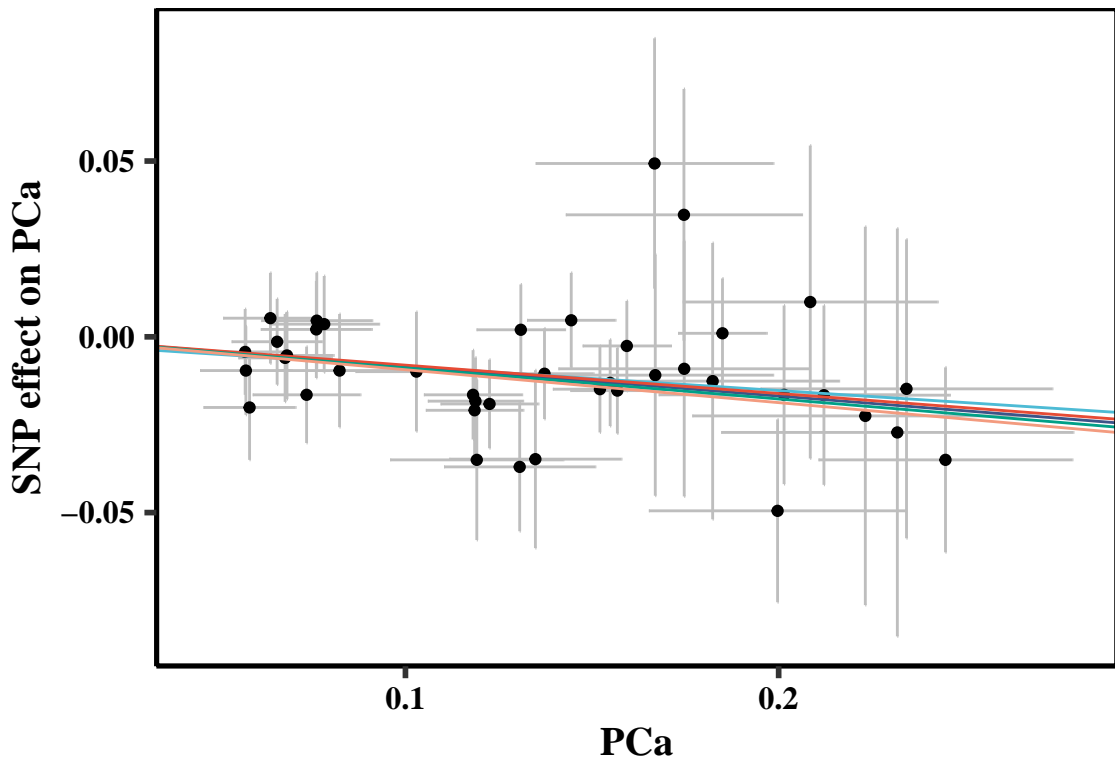

Supplement: Supplementary file 1 [file DataSheet1.zip › Supplementary Figure 1/CD109.pdf]

# MR of FASN

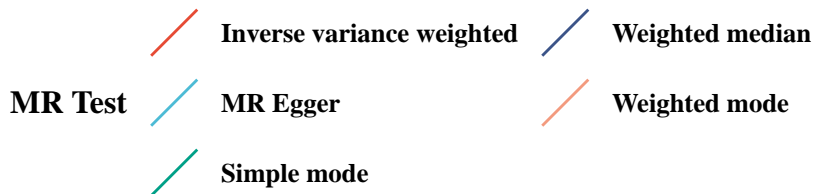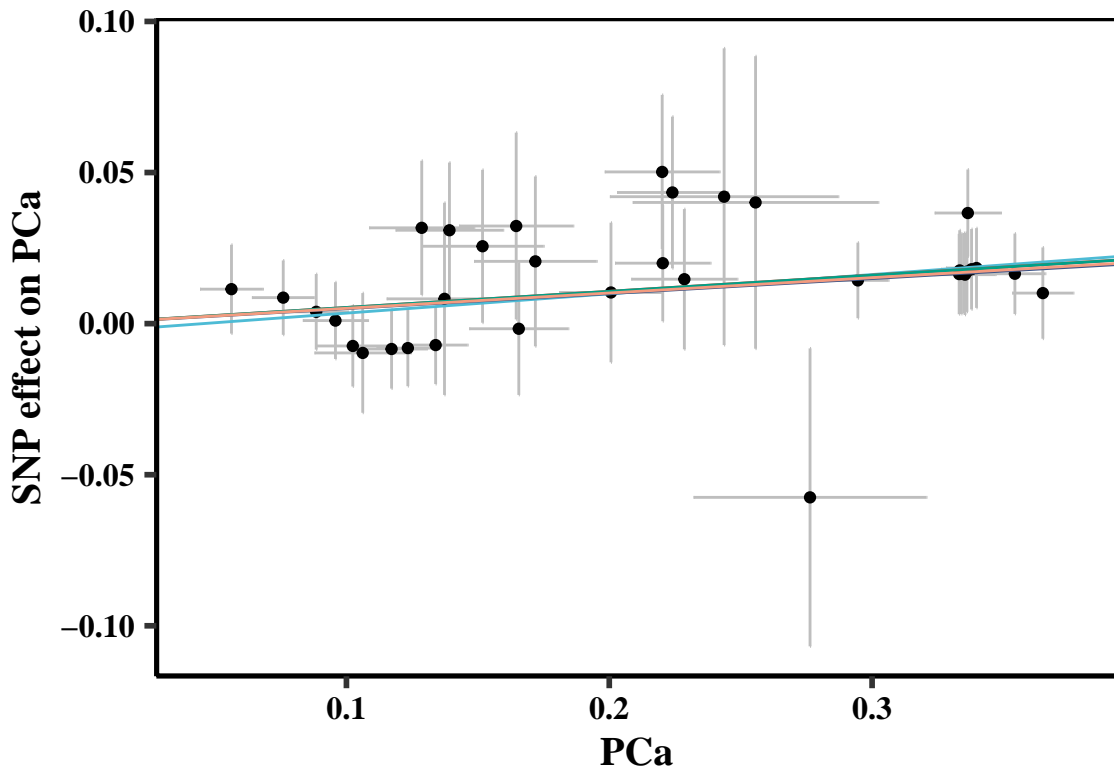

Supplement: Supplementary file 1 [file DataSheet1.zip › Supplementary Figure 1/FASN.pdf]

# MR of FBN1

MR Test

|                           |                 |
|---------------------------|-----------------|
| Inverse variance weighted | Weighted median |
| MR Egger                  | Weighted mode   |
| Simple mode               |                 |

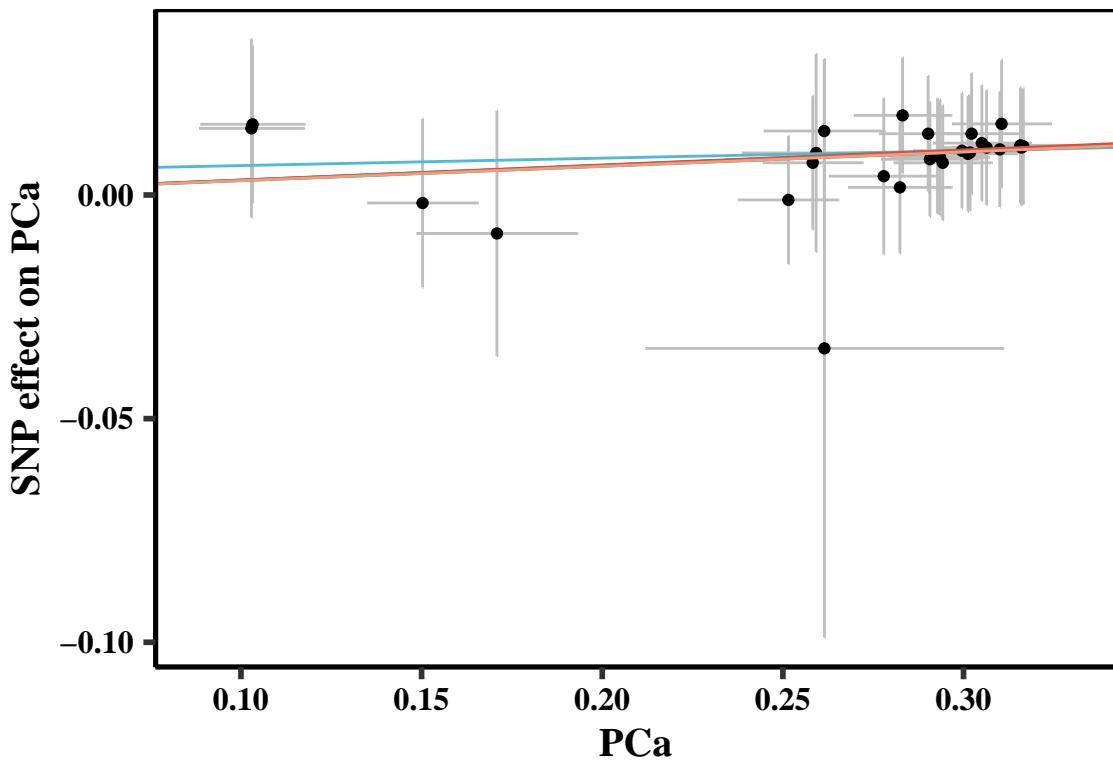

Supplement: Supplementary file 1 [file DataSheet1.zip › Supplementary Figure 1/FBN1.pdf]

# MR of GP5

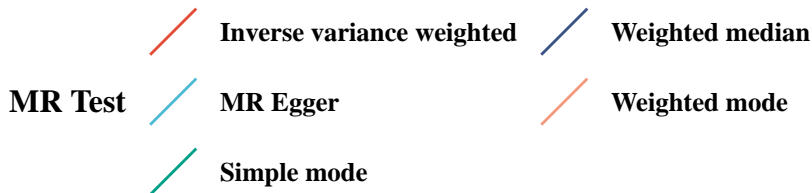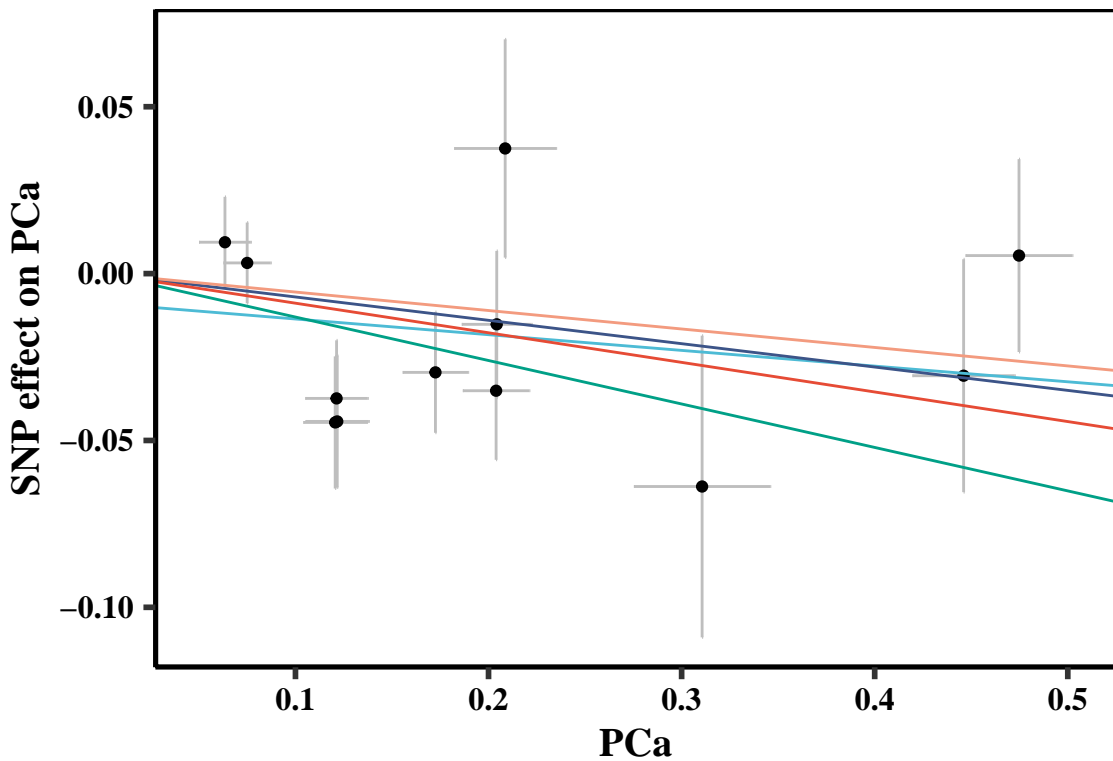

Supplement: Supplementary file 1 [file DataSheet1.zip › Supplementary Figure 1/GP5.pdf]

# MR of IFI16

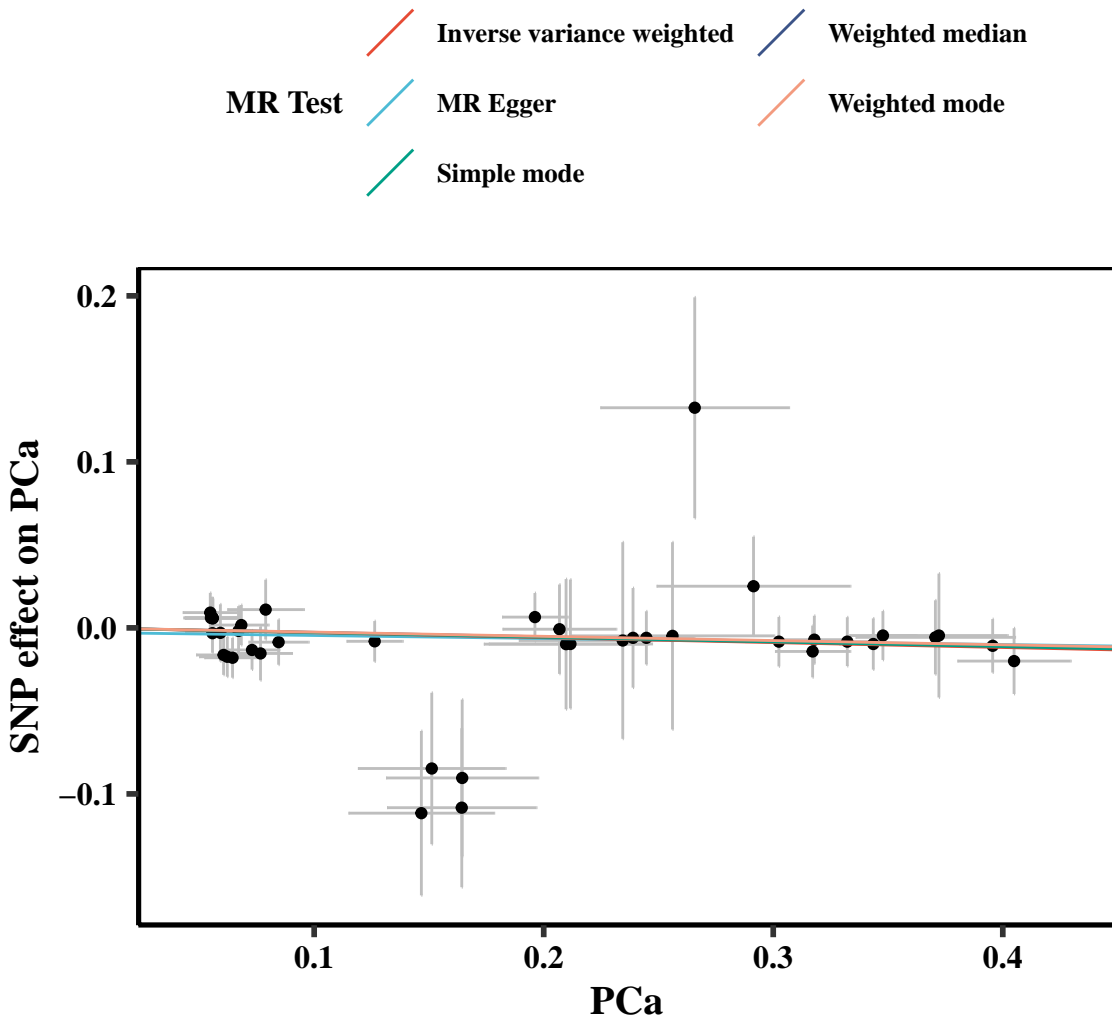

Supplement: Supplementary file 1 [file DataSheet1.zip › Supplementary Figure 1/IFI16.pdf]

# MR of IL1RL1

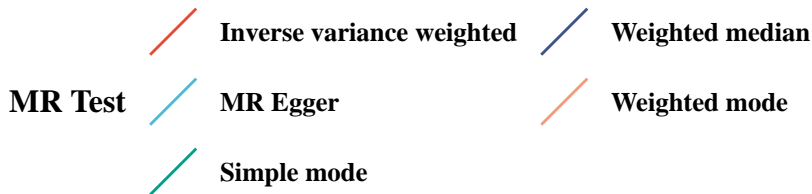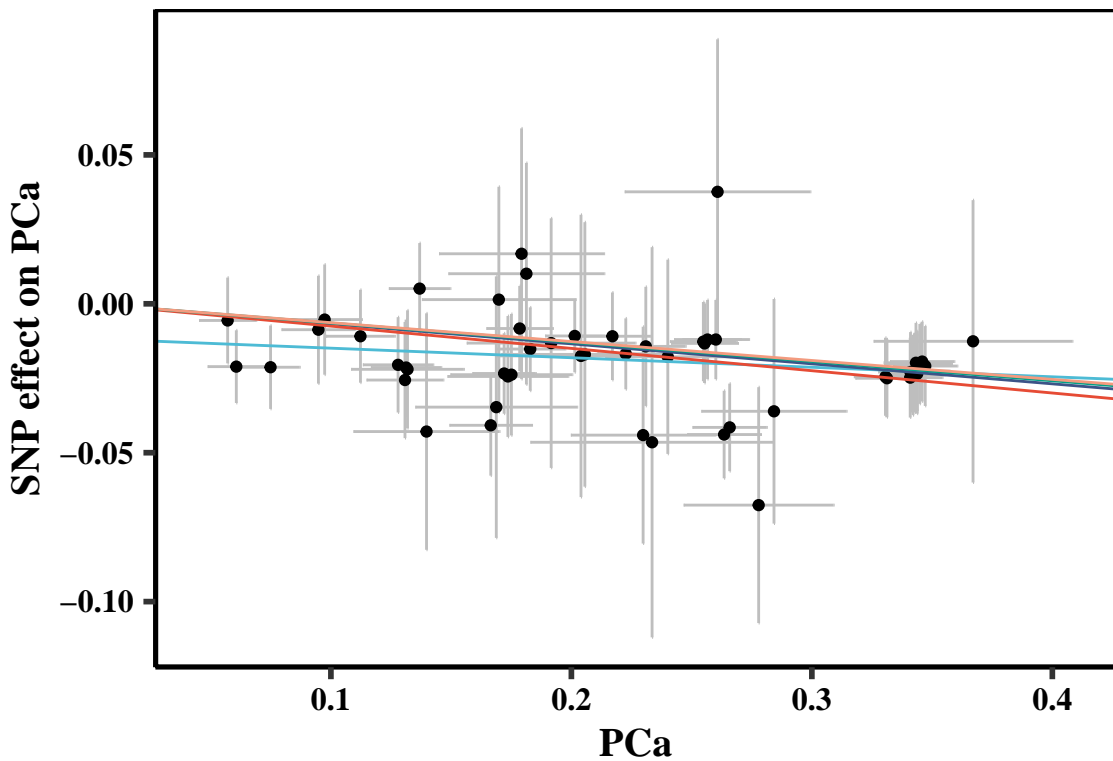

Supplement: Supplementary file 1 [file DataSheet1.zip › Supplementary Figure 1/IL1RL1.pdf]

## MR of ISG15

## MR Test

### Inverse variance weighted

### Weighted median

### MR Egger

### Weighted mode

## Simple mode

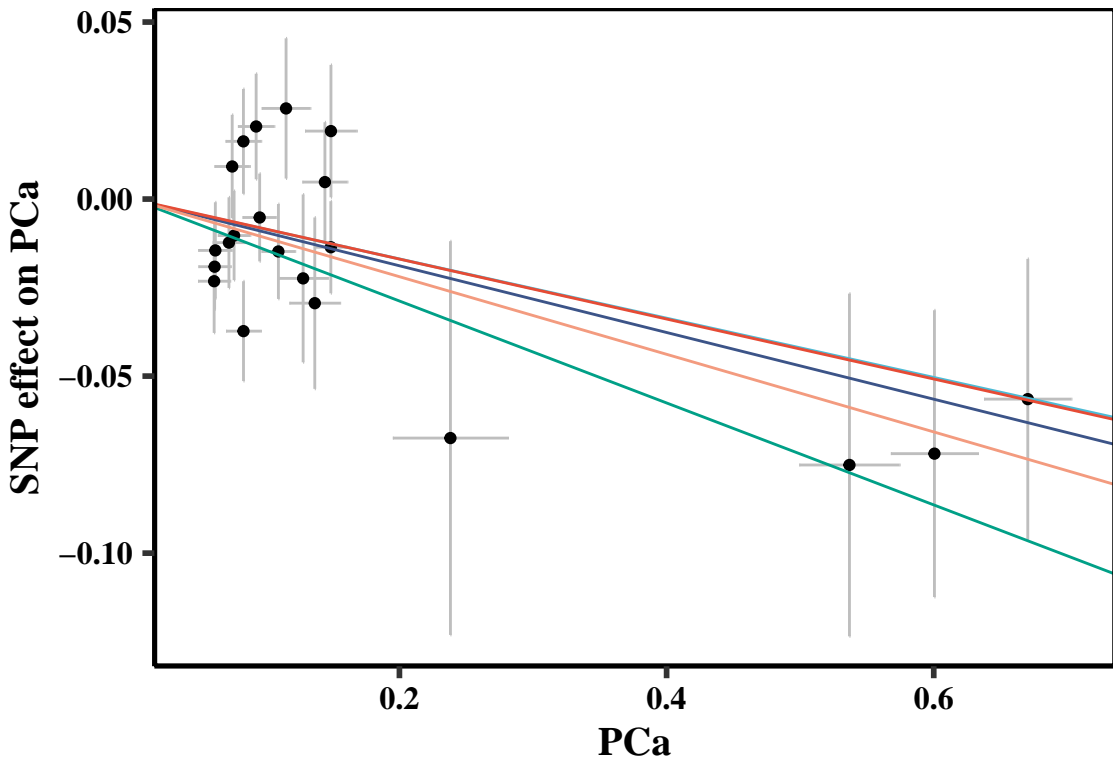

Supplement: Supplementary file 1 [file DataSheet1.zip › Supplementary Figure 1/ISG15.pdf]

# MR of KIT

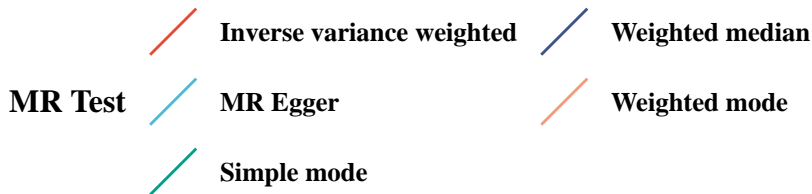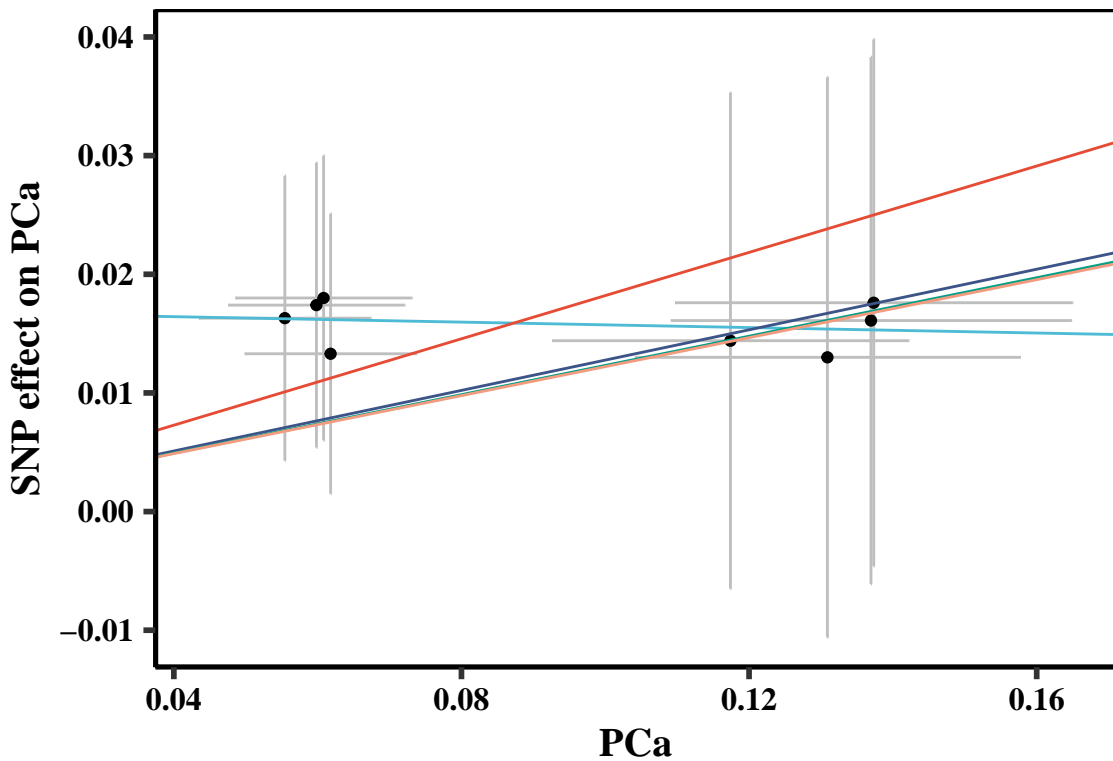

Supplement: Supplementary file 1 [file DataSheet1.zip › Supplementary Figure 1/KIT.pdf]

# MR of KLF10

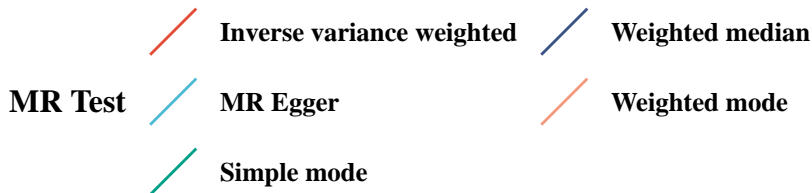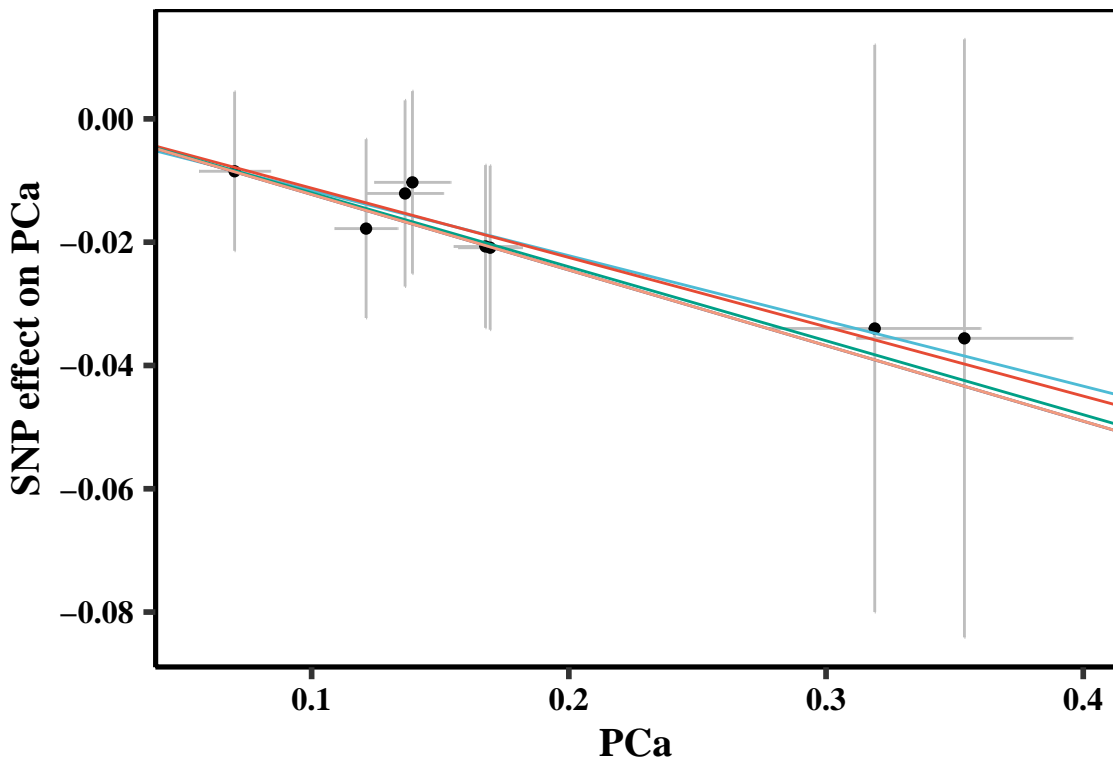

Supplement: Supplementary file 1 [file DataSheet1.zip › Supplementary Figure 1/KLF10.pdf]

# MR of LOX

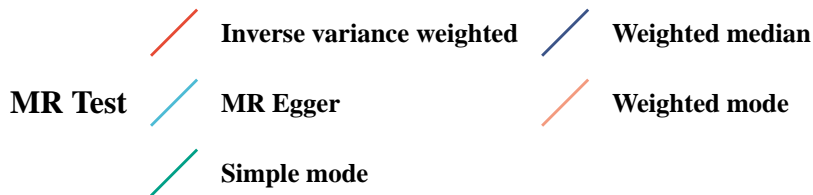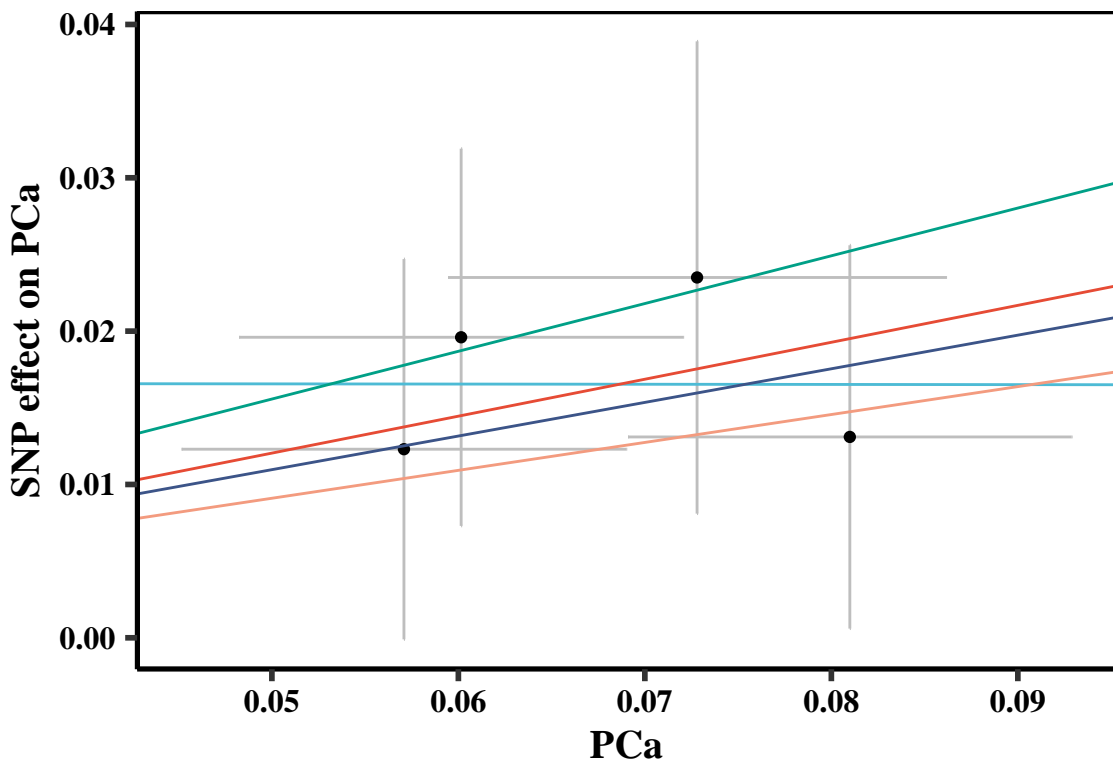

Supplement: Supplementary file 1 [file DataSheet1.zip › Supplementary Figure 1/LOX.pdf]

# MR of MTURN

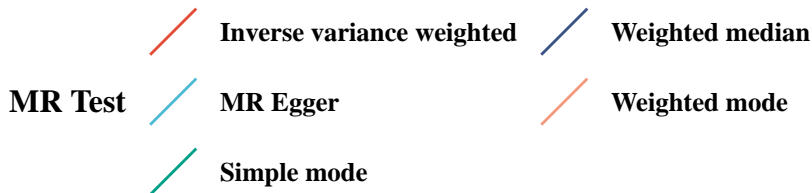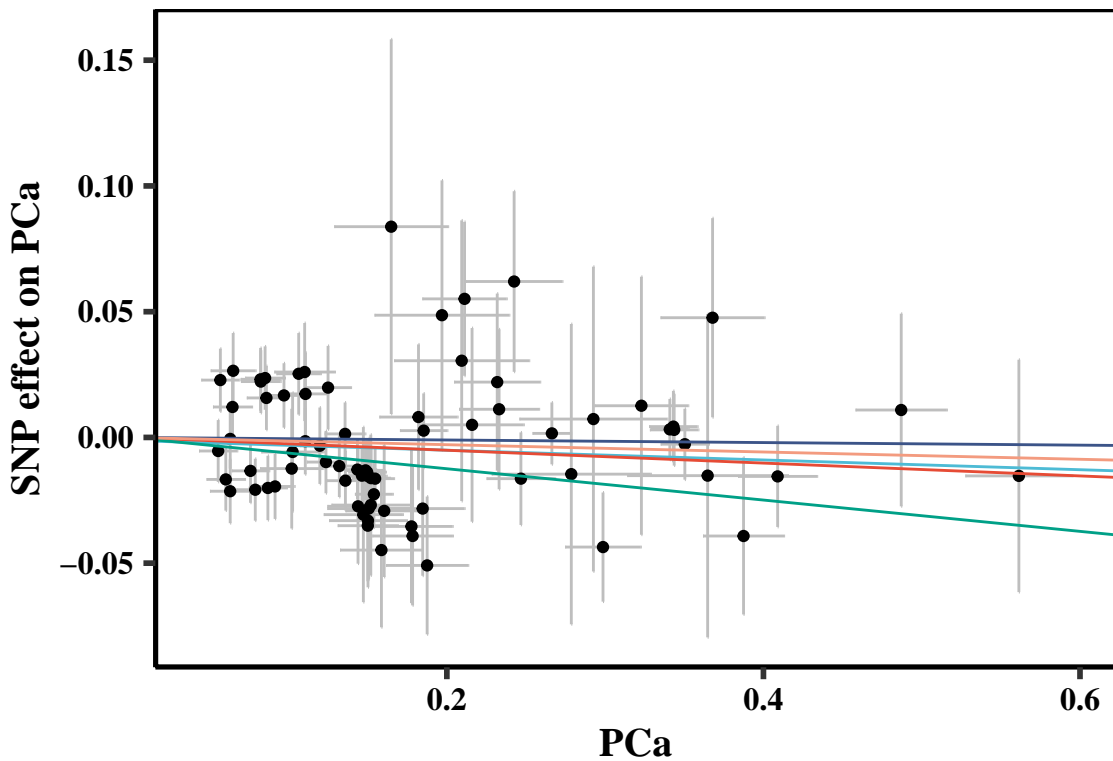

Supplement: Supplementary file 1 [file DataSheet1.zip › Supplementary Figure 1/MTURN.pdf]

# MR of NR3C1

MR Test

- Inverse variance weighted
- MR Egger
- Simple mode
- Weighted median
- Weighted mode

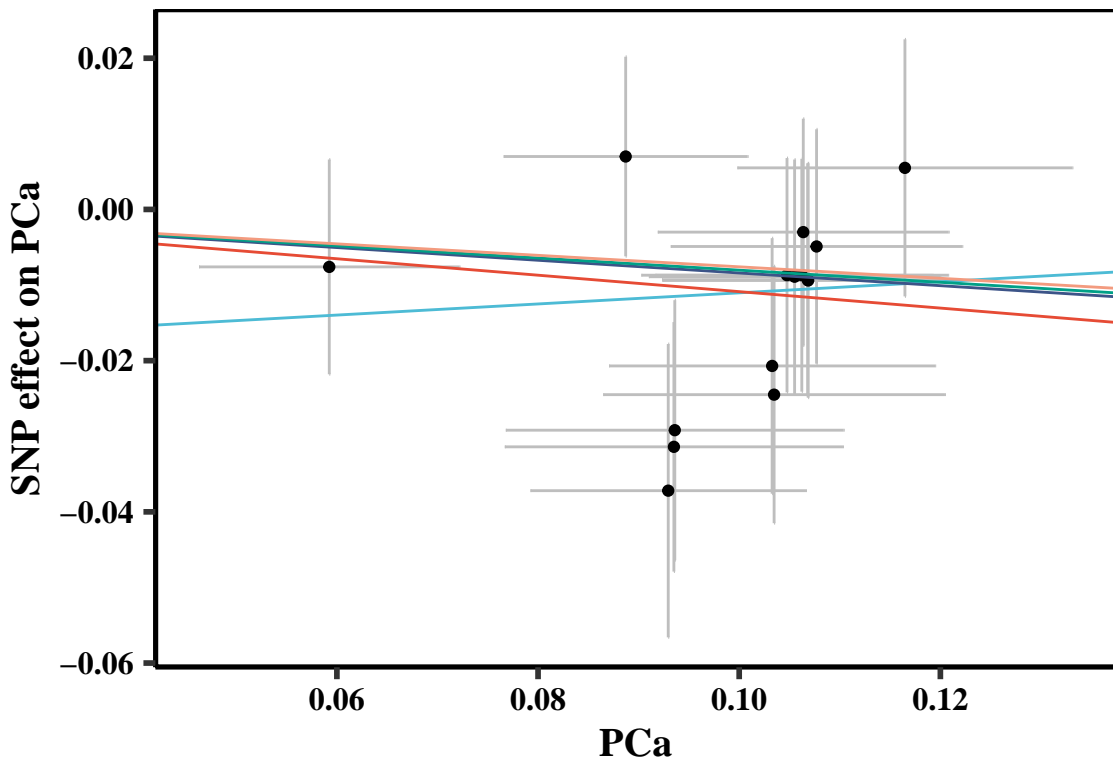

Supplement: Supplementary file 1 [file DataSheet1.zip › Supplementary Figure 1/NR3C1.pdf]

# MR of PPP3CA

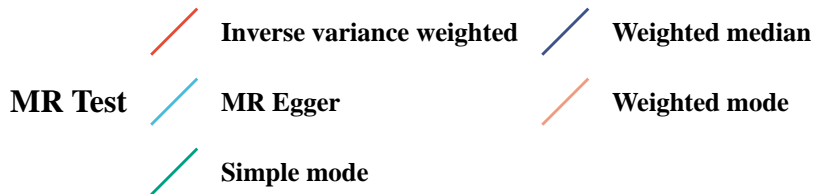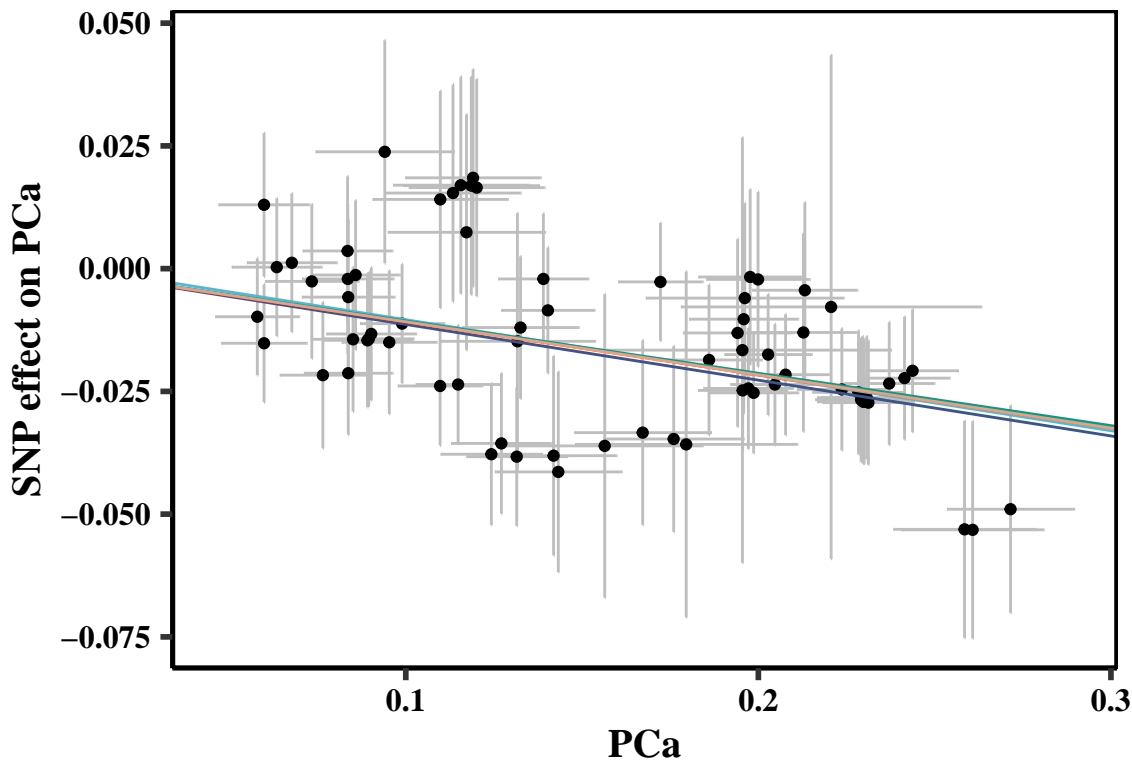

Supplement: Supplementary file 1 [file DataSheet1.zip › Supplementary Figure 1/PPP3CA.pdf]

# MR of RACGAP1

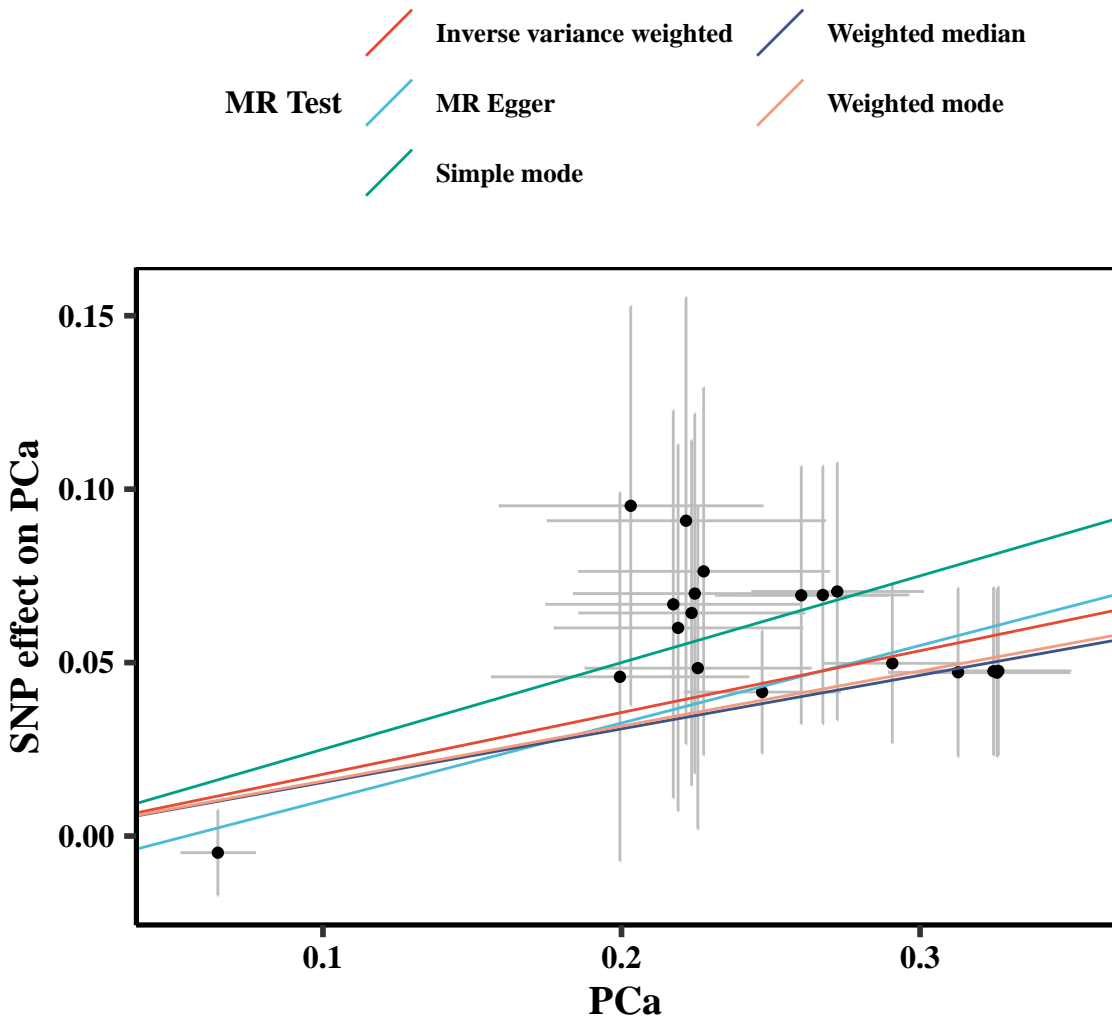

Supplement: Supplementary file 1 [file DataSheet1.zip › Supplementary Figure 1/RACGAP1.pdf]

# MR of STAT5B

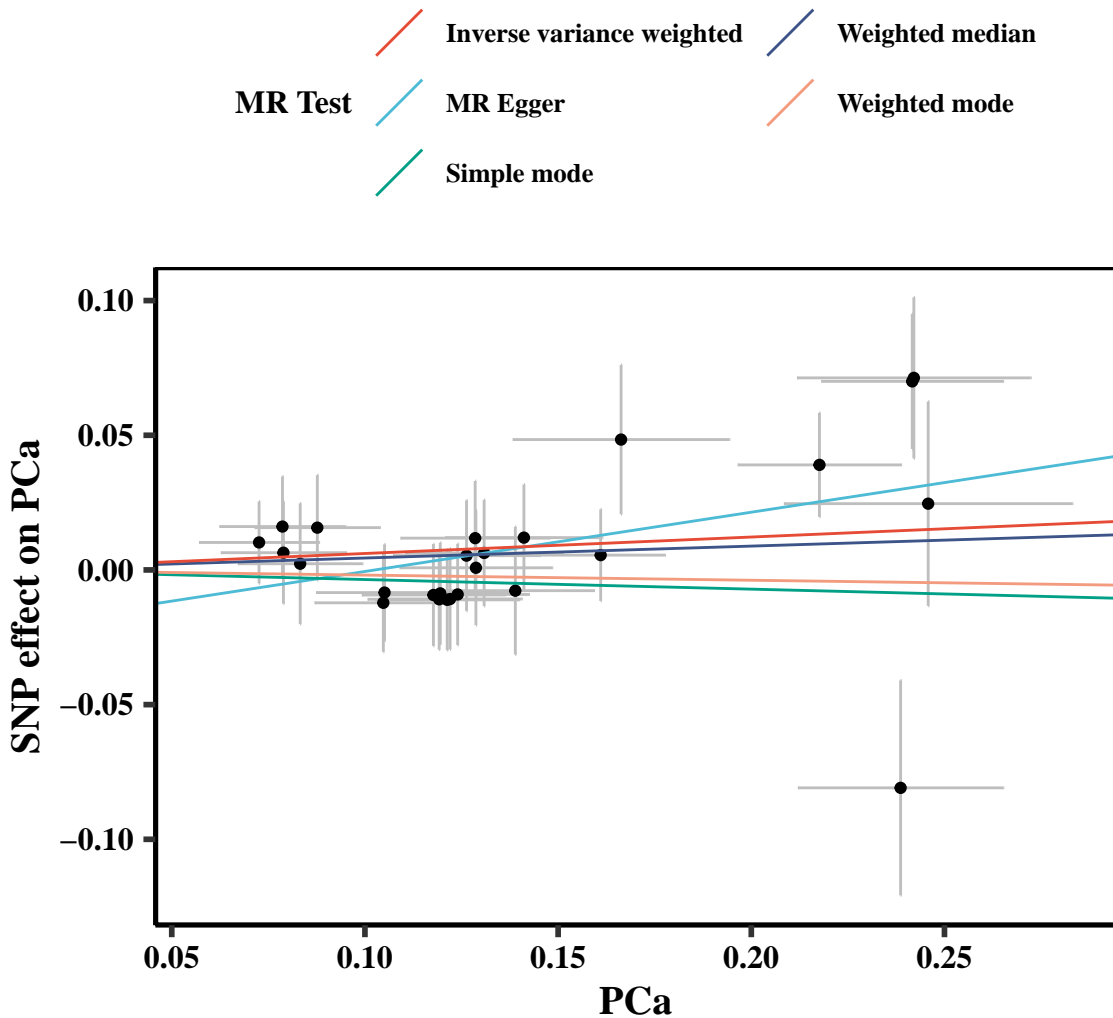

Supplement: Supplementary file 1 [file DataSheet1.zip › Supplementary Figure 1/STAT5B.pdf]

# MR of TLR2

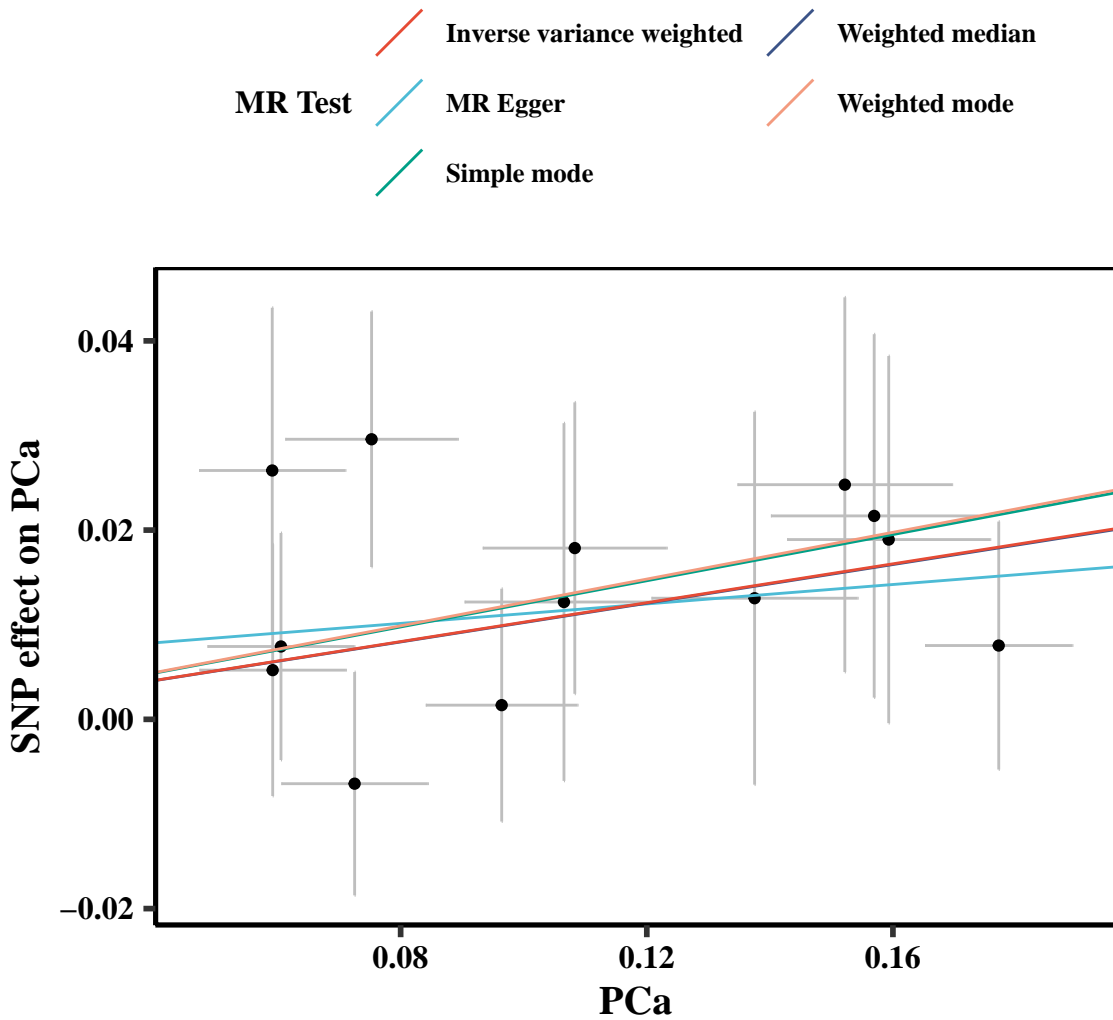

Supplement: Supplementary file 1 [file DataSheet1.zip › Supplementary Figure 1/TLR2.pdf]

# MR of TLR3

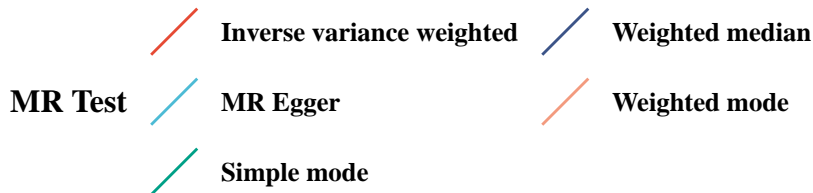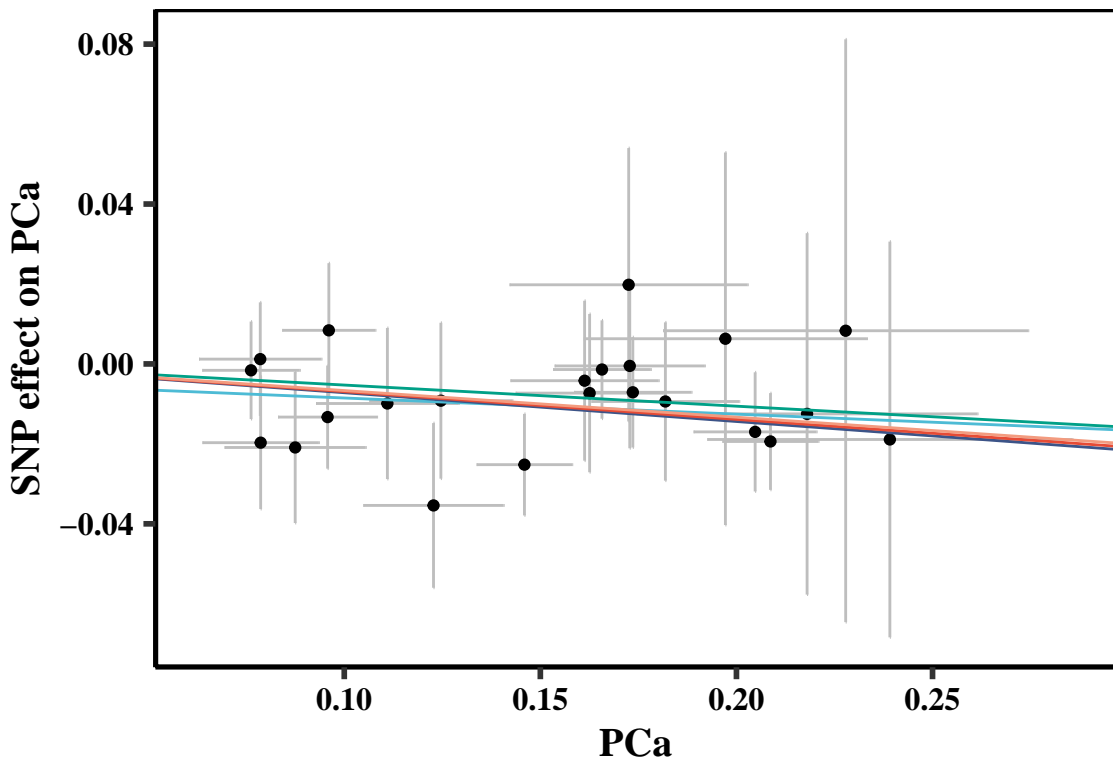

Supplement: Supplementary file 1 [file DataSheet1.zip › Supplementary Figure 1/TLR3.pdf]

# MR effect size for ACTN1 on PCa

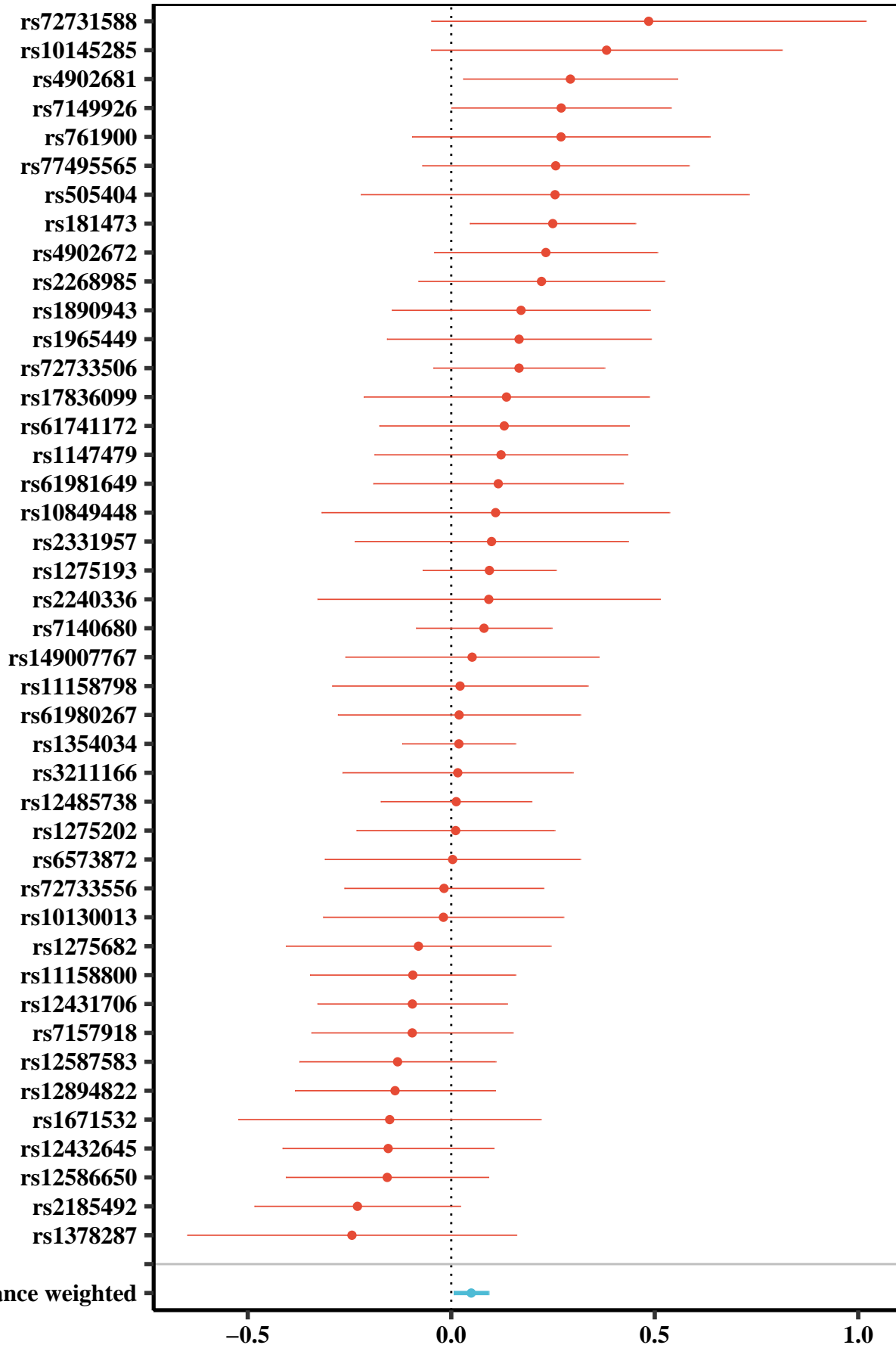

Supplement: Supplementary file 2 [file DataSheet2.zip › Supplementary Figure 2/ACTN1.pdf]

# MR effect size for BATF3 on PCa

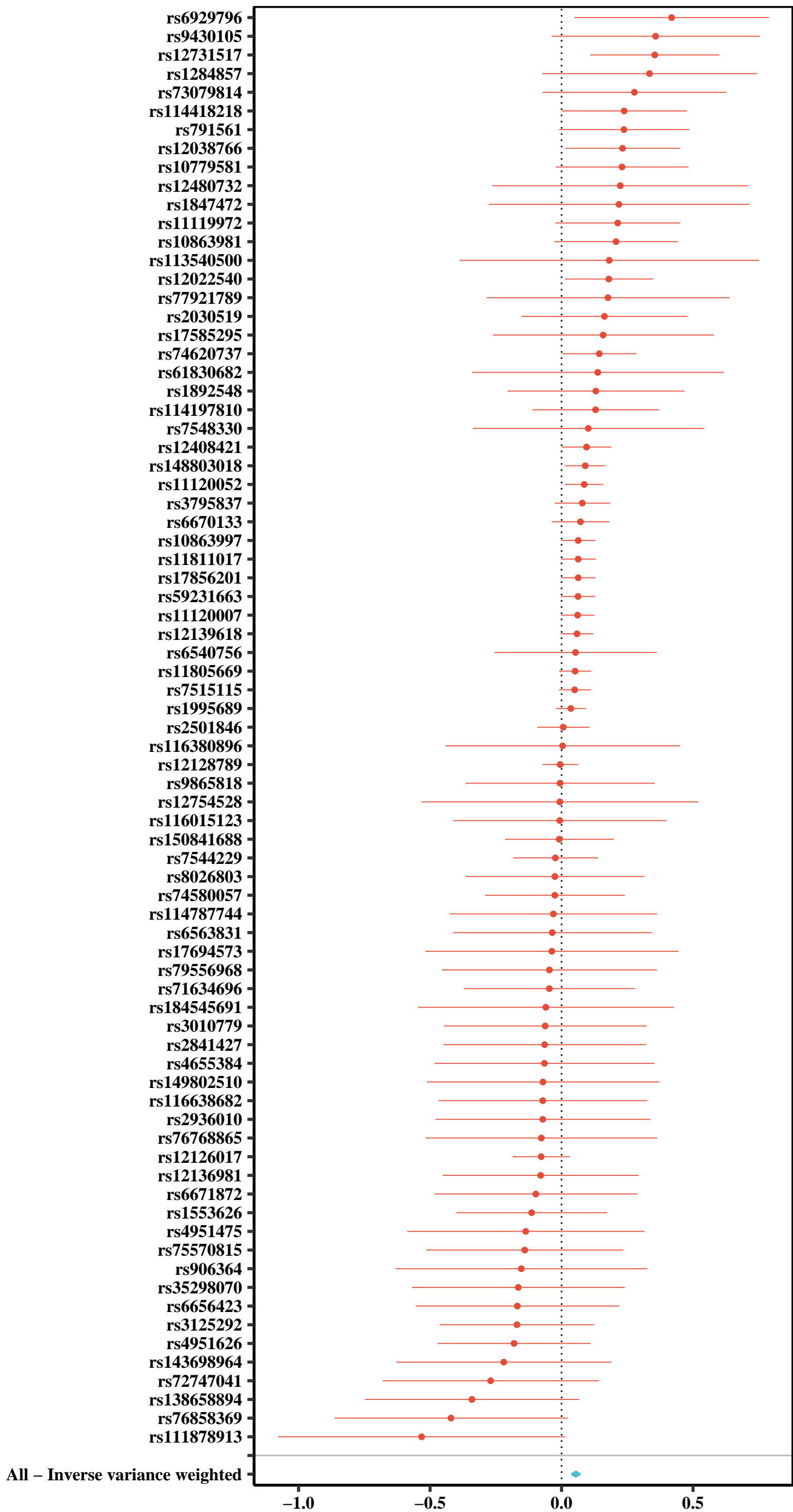

Supplement: Supplementary file 2 [file DataSheet2.zip › Supplementary Figure 2/BATF3.pdf]

# MR effect size for BMP2 on PCa

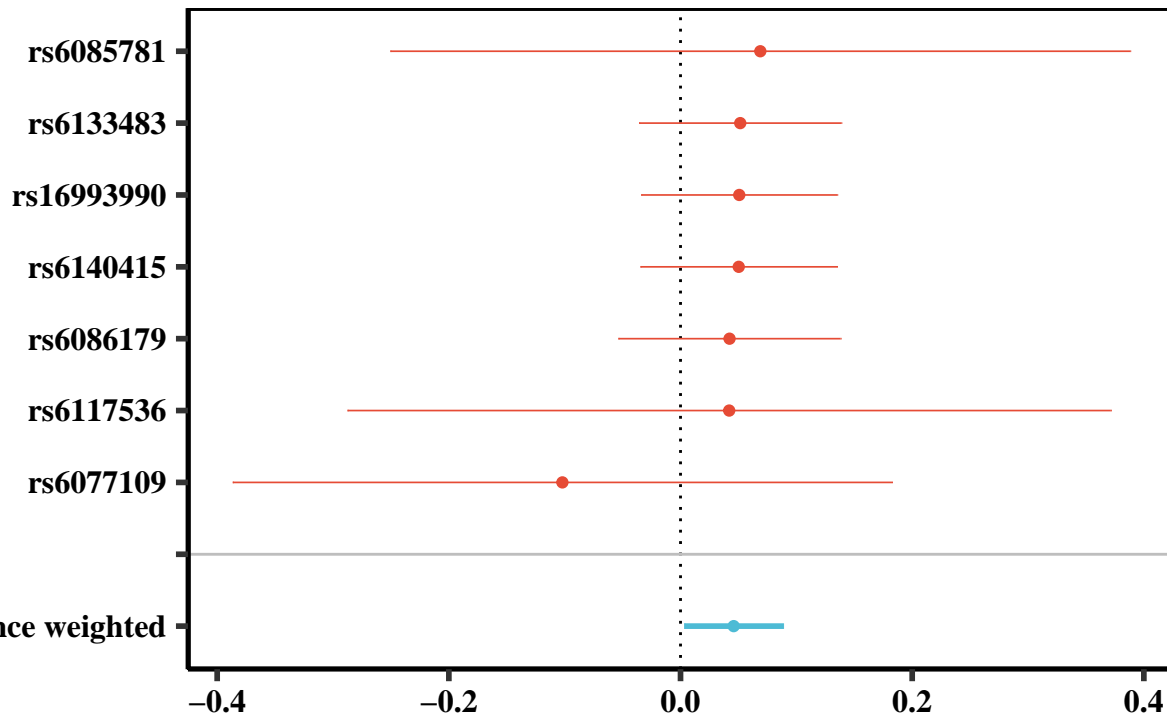

Supplement: Supplementary file 2 [file DataSheet2.zip › Supplementary Figure 2/BMP2.pdf]

# MR effect size for CD109 on PCa

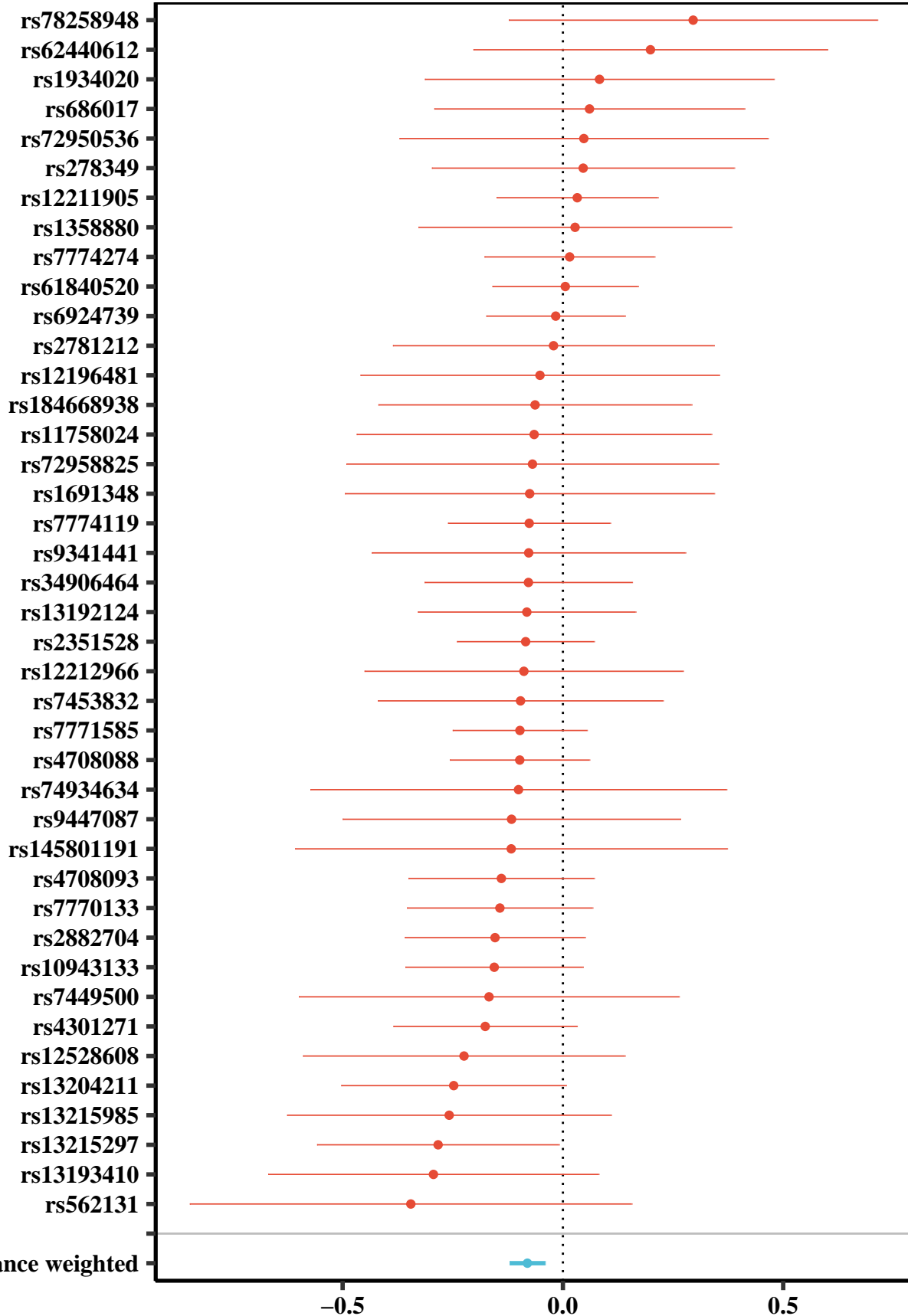

All - Inverse variance weighted

Supplement: Supplementary file 2 [file DataSheet2.zip › Supplementary Figure 2/CD109.pdf]

# MR effect size for FASN on PCa

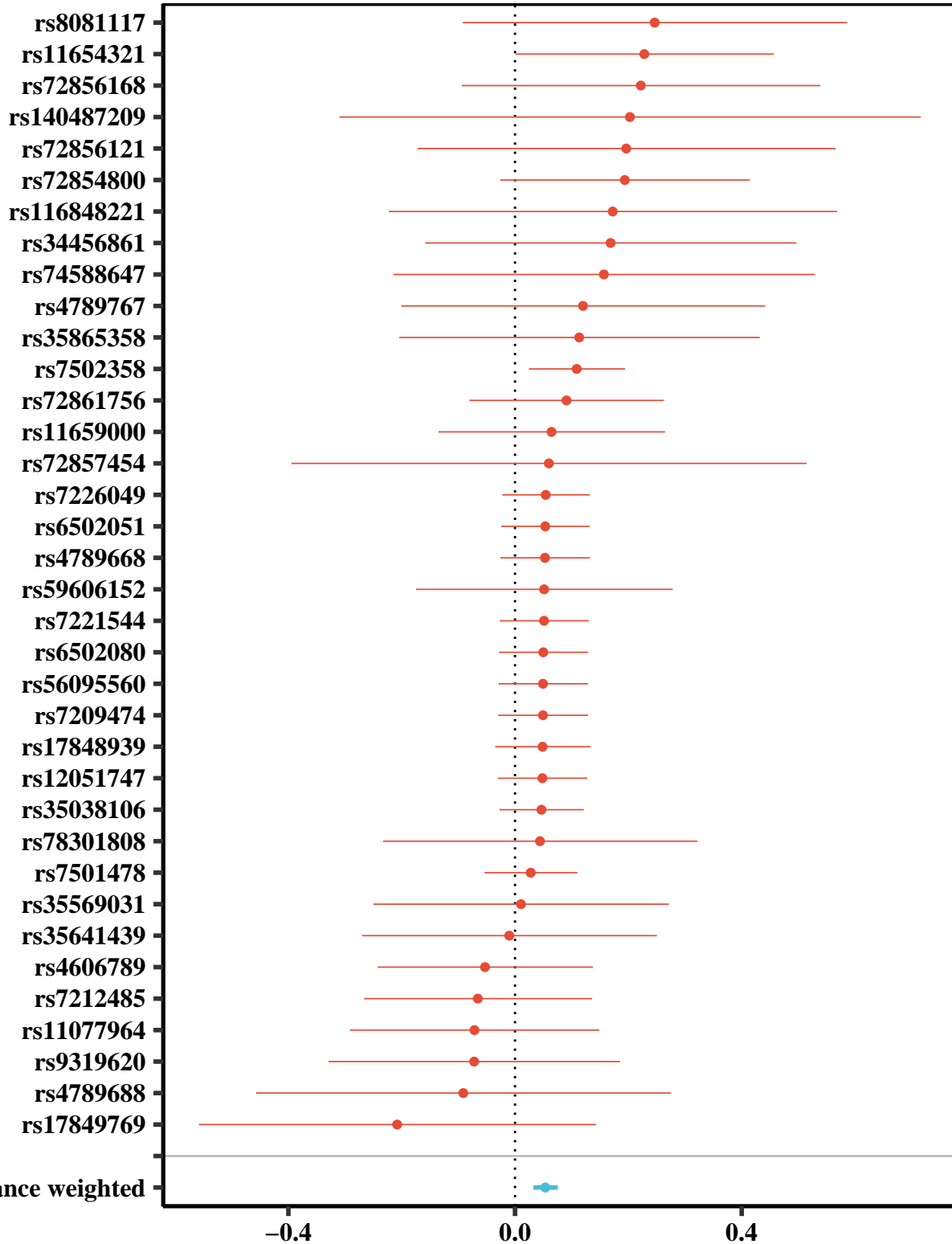

Supplement: Supplementary file 2 [file DataSheet2.zip › Supplementary Figure 2/FASN.pdf]

# MR effect size for FBN1 on PCa

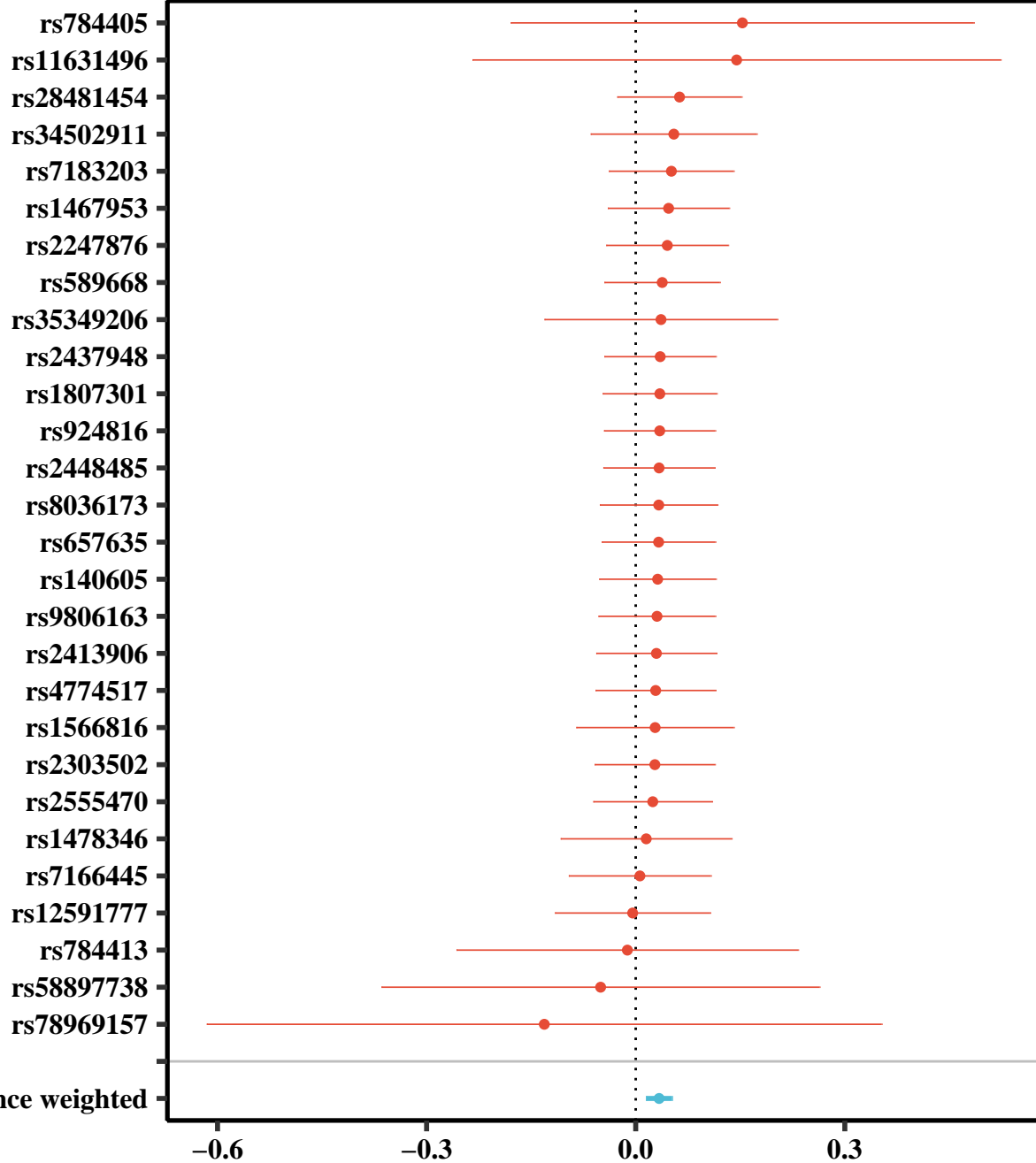

Supplement: Supplementary file 2 [file DataSheet2.zip › Supplementary Figure 2/FBN1.pdf]

# MR effect size for FLNA on PCa

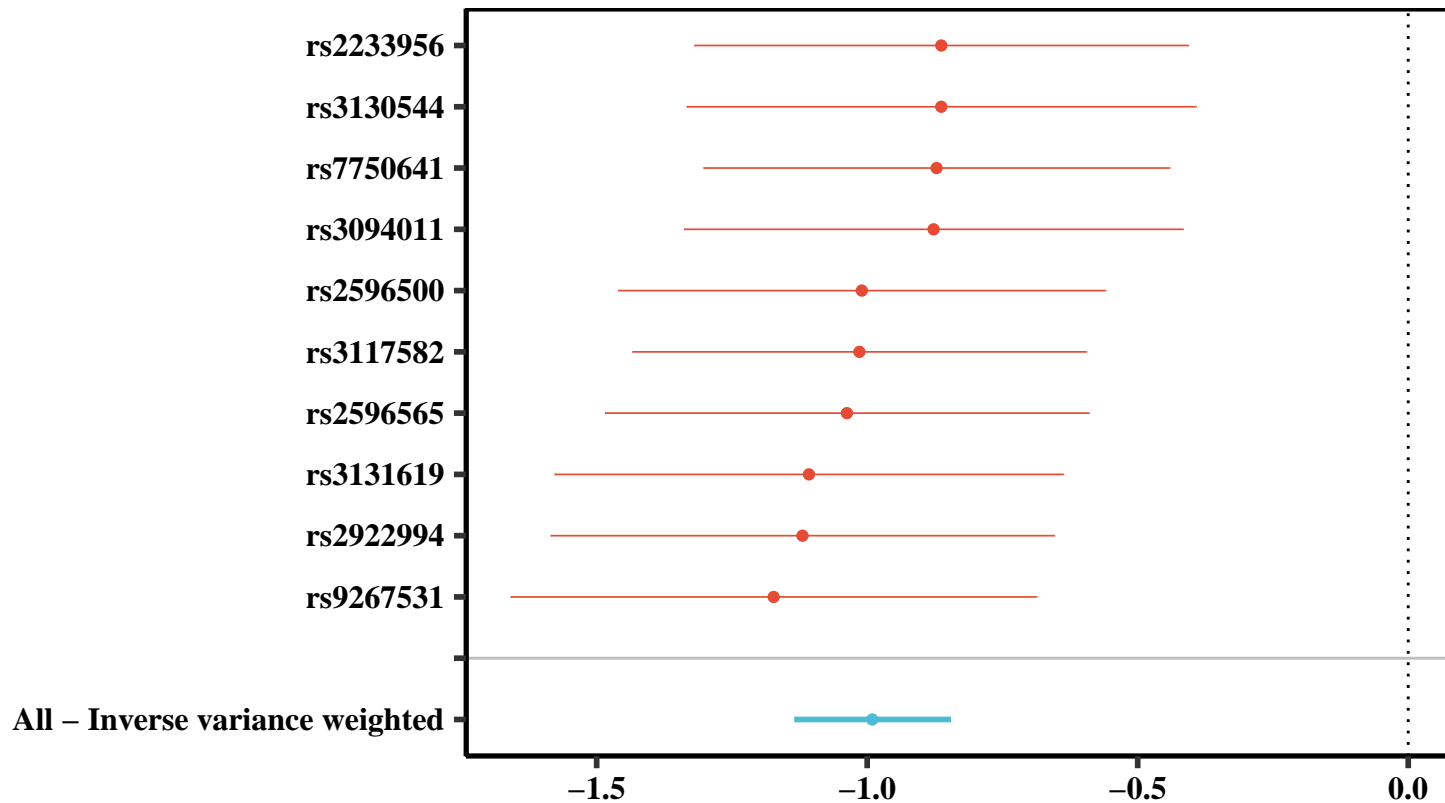

Supplement: Supplementary file 2 [file DataSheet2.zip › Supplementary Figure 2/FLNA.pdf]

# MR effect size for GATA3 on PCa

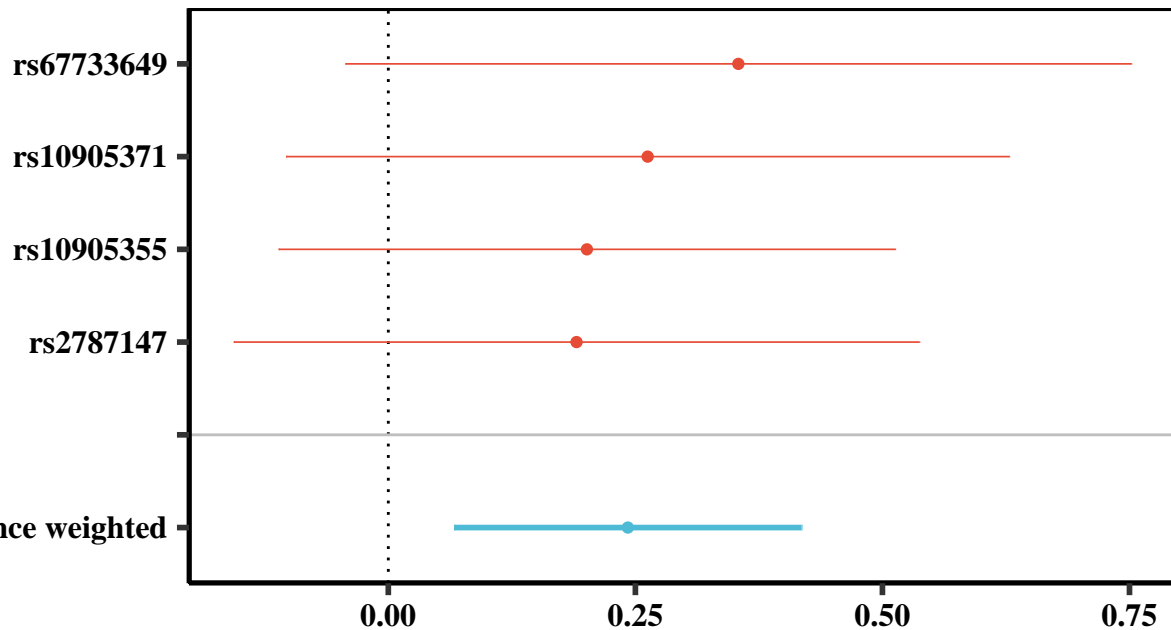

Supplement: Supplementary file 2 [file DataSheet2.zip › Supplementary Figure 2/GATA3.pdf]

# MR effect size for GP5 on PCa

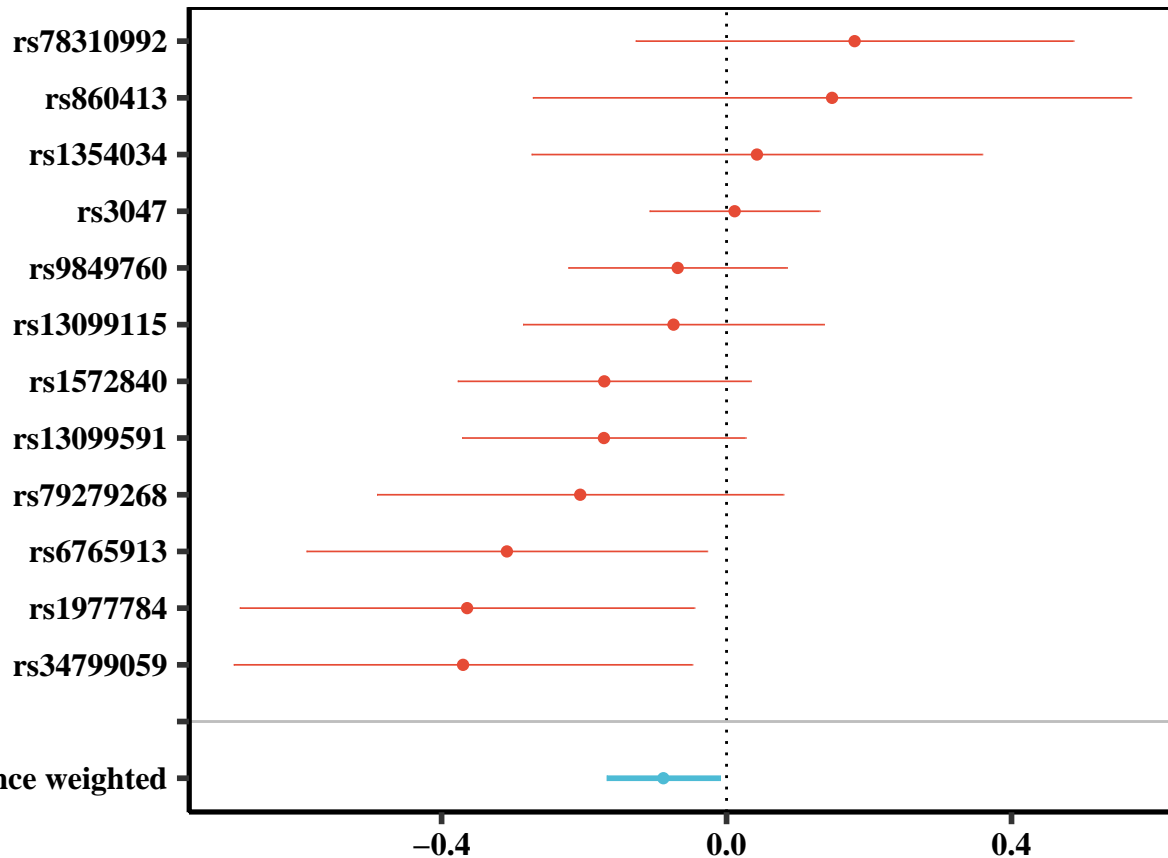

Supplement: Supplementary file 2 [file DataSheet2.zip › Supplementary Figure 2/GP5.pdf]

# MR effect size for IFI16 on PCa

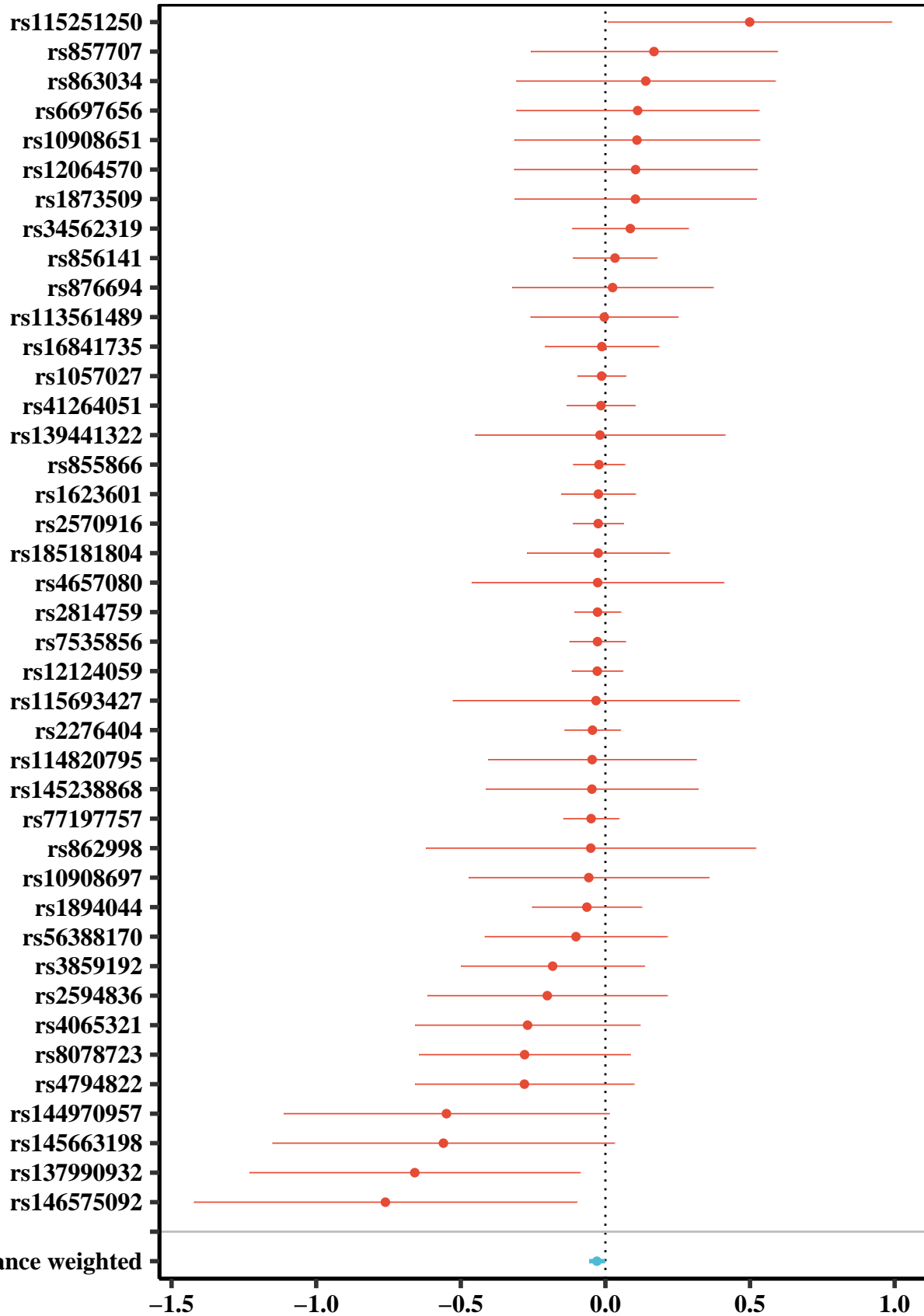

Supplement: Supplementary file 2 [file DataSheet2.zip › Supplementary Figure 2/IFI16.pdf]

MR effect size for IL1RL1 on PCa

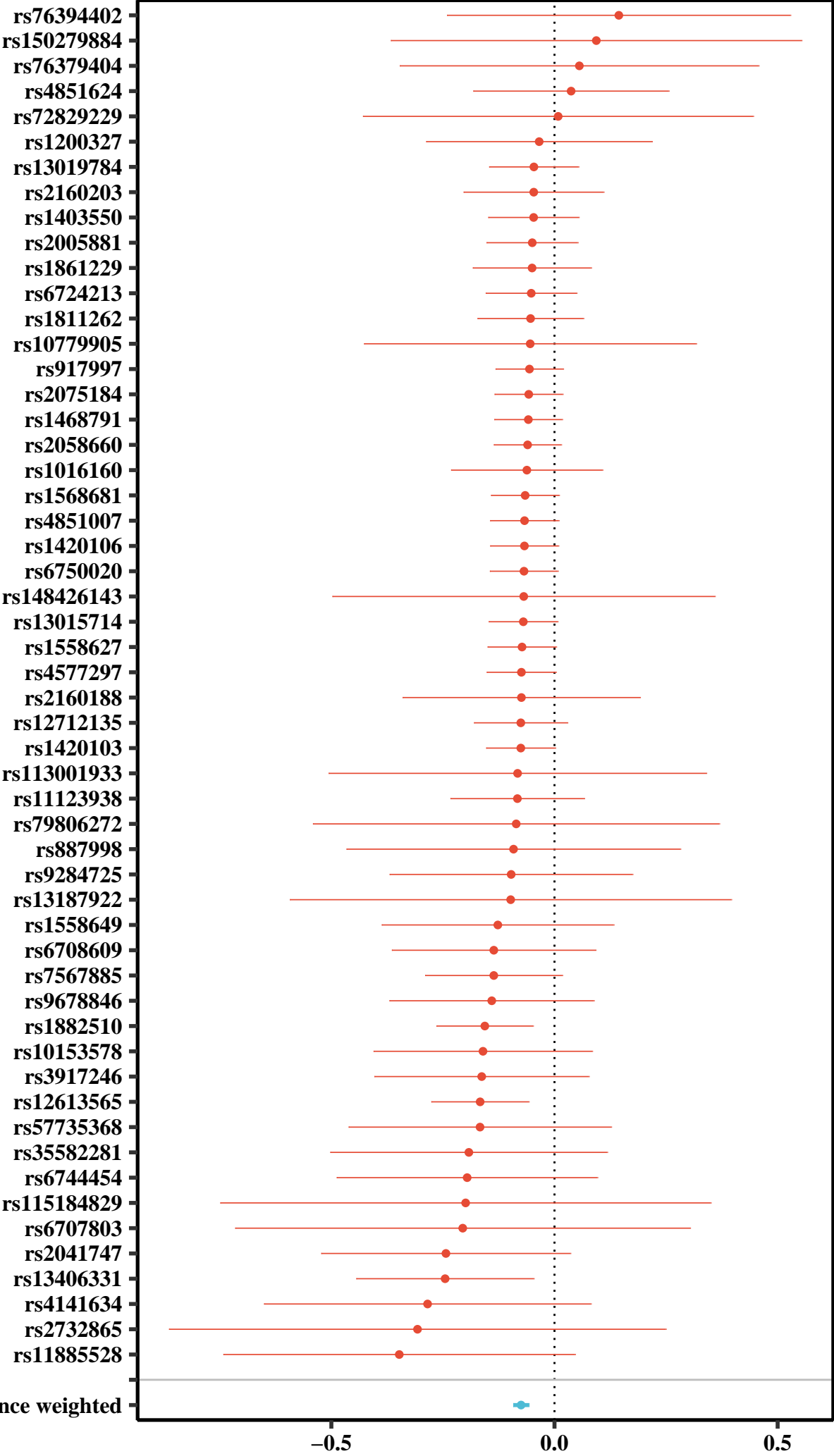

Supplement: Supplementary file 2 [file DataSheet2.zip › Supplementary Figure 2/IL1RL1.pdf]

# MR effect size for ISG15 on PCa

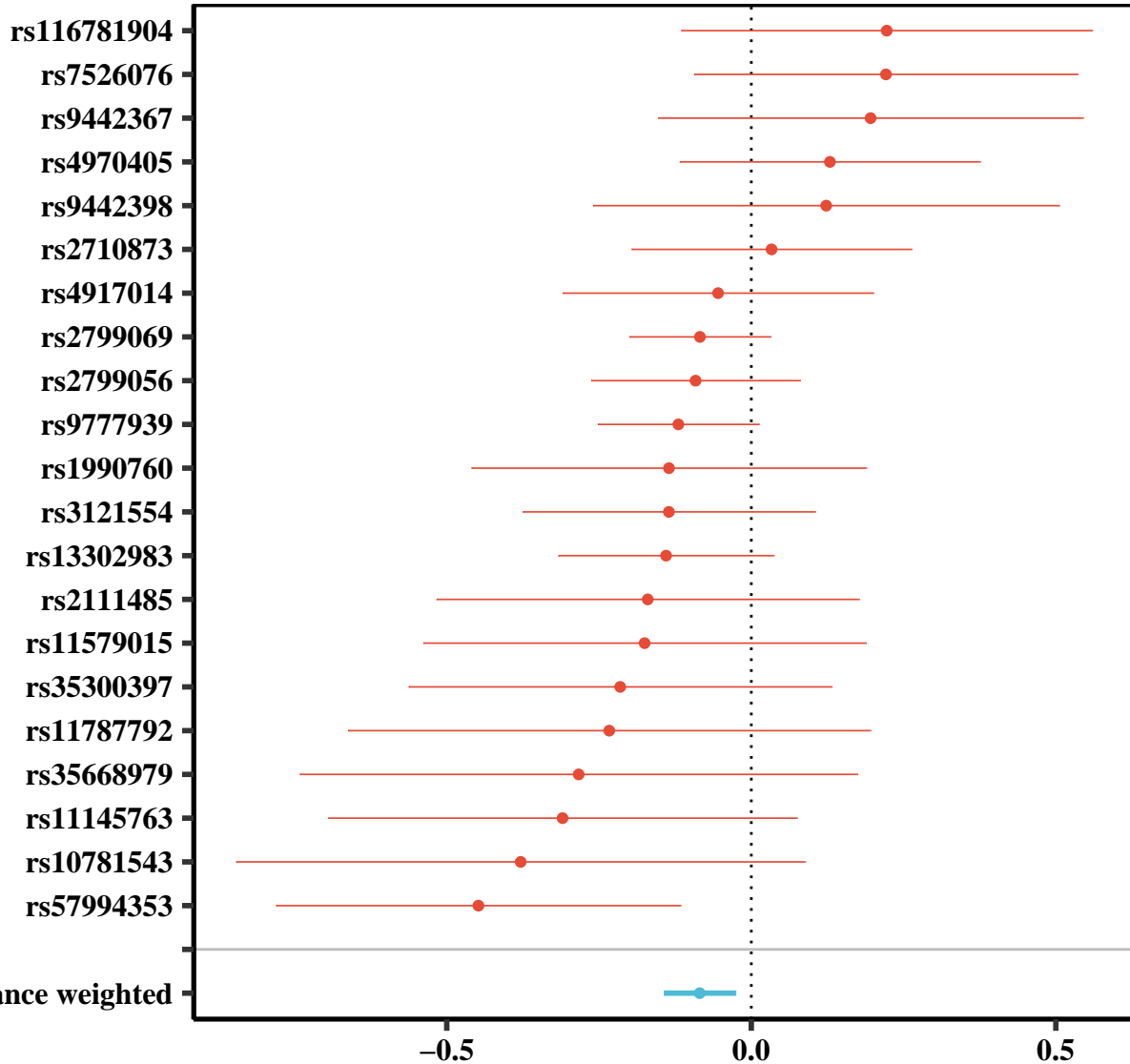

Supplement: Supplementary file 2 [file DataSheet2.zip › Supplementary Figure 2/ISG15.pdf]

# MR effect size for KIT on PCa

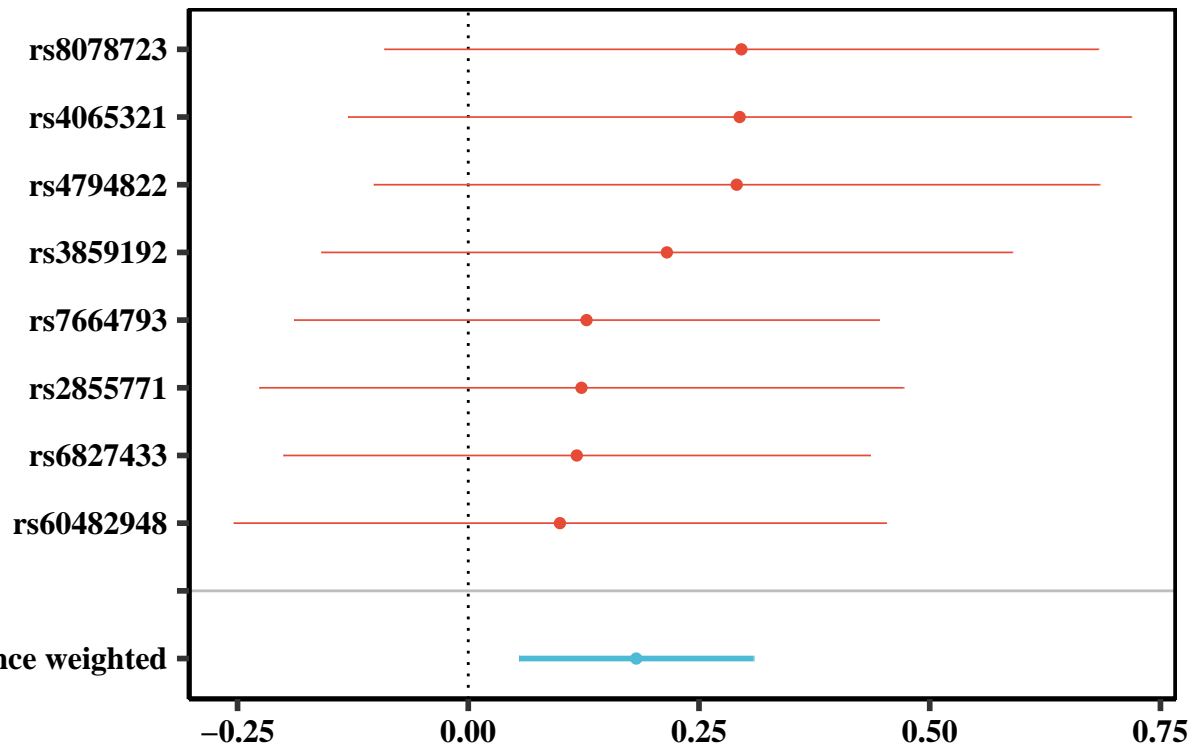

Supplement: Supplementary file 2 [file DataSheet2.zip › Supplementary Figure 2/KIT.pdf]

# MR effect size for KLF10 on PCa

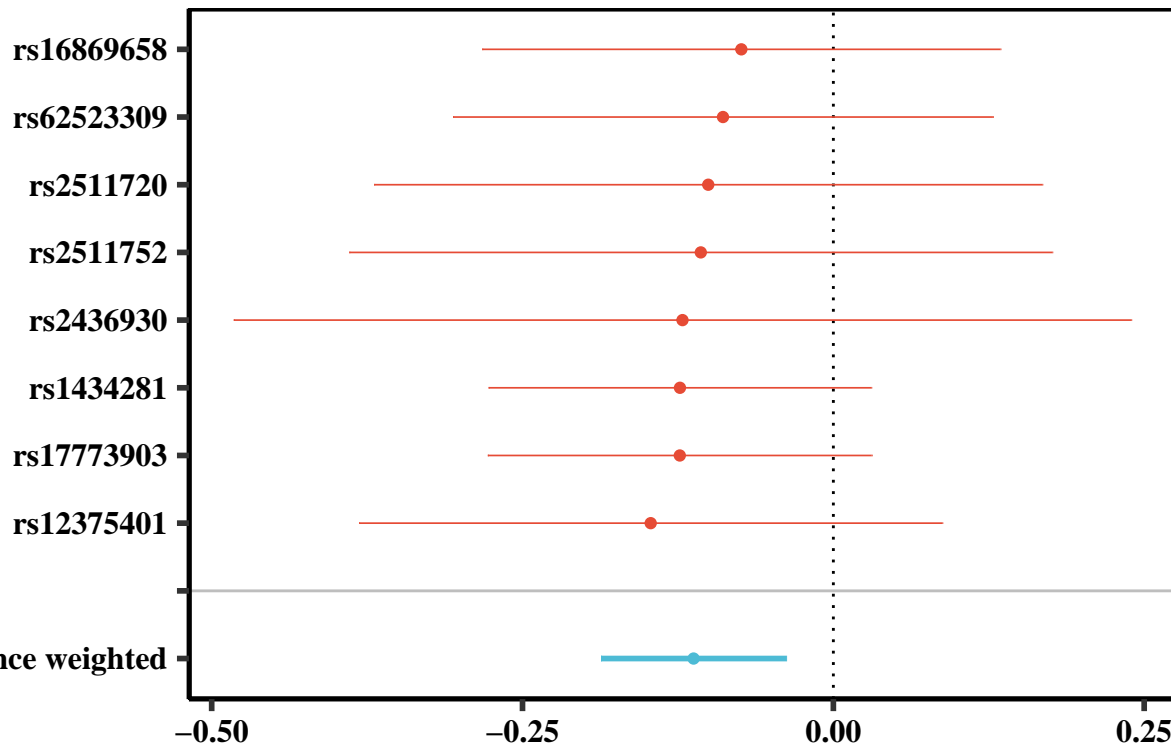

Supplement: Supplementary file 2 [file DataSheet2.zip › Supplementary Figure 2/KLF10.pdf]

# MR effect size for LOX on PCa

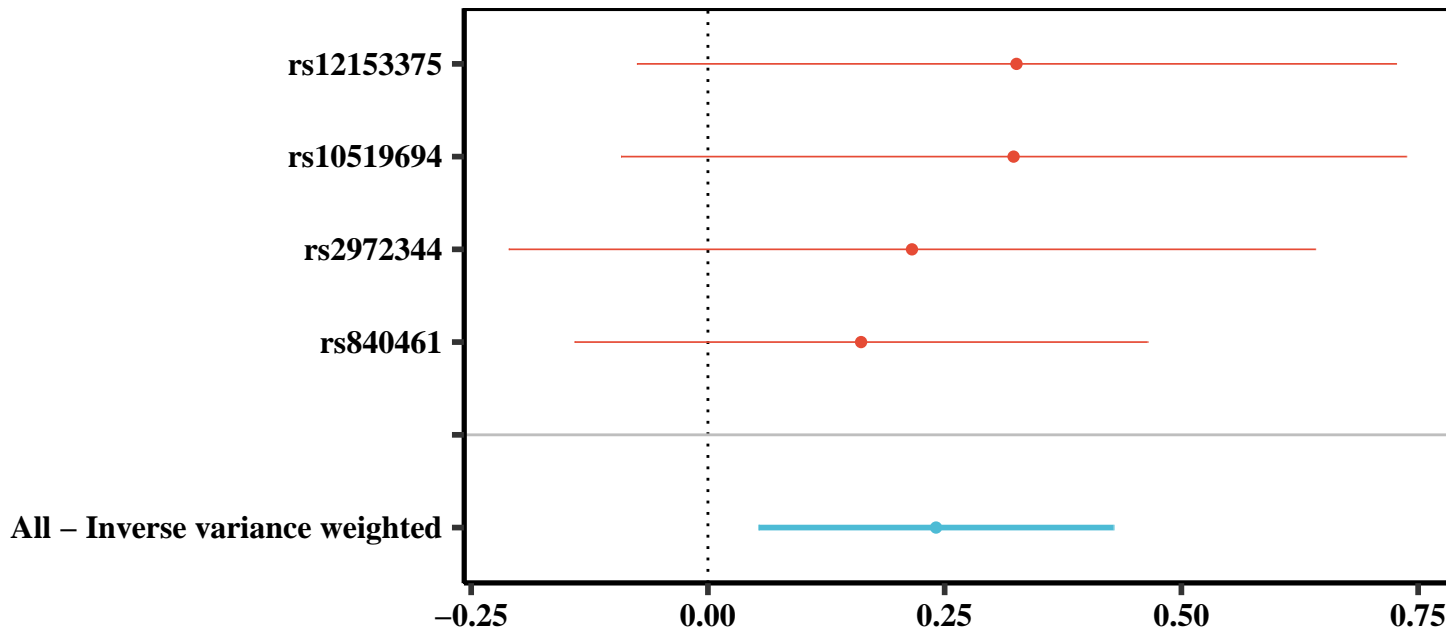

Supplement: Supplementary file 2 [file DataSheet2.zip › Supplementary Figure 2/LOX.pdf]

# MR effect size for MITF on PCa

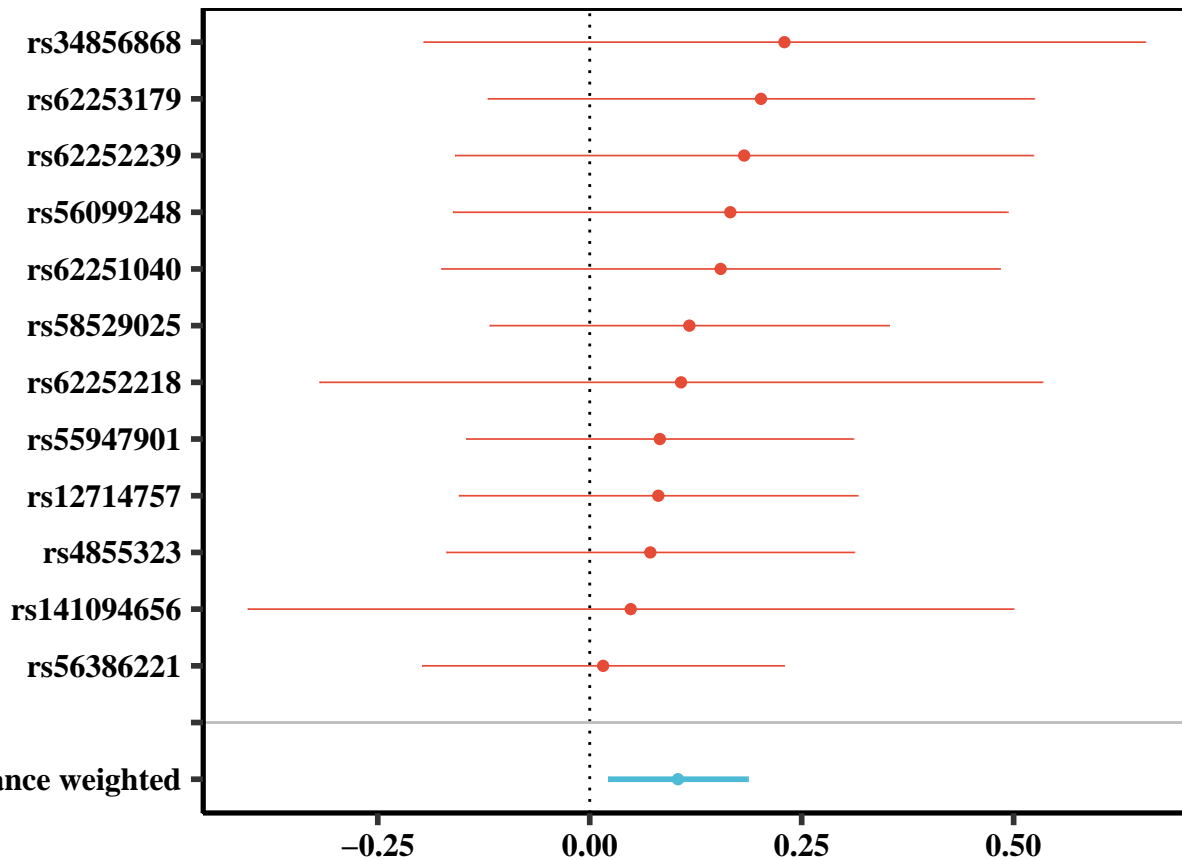

Supplement: Supplementary file 2 [file DataSheet2.zip › Supplementary Figure 2/MITF.pdf]

MR effect size for MTURN on PCa

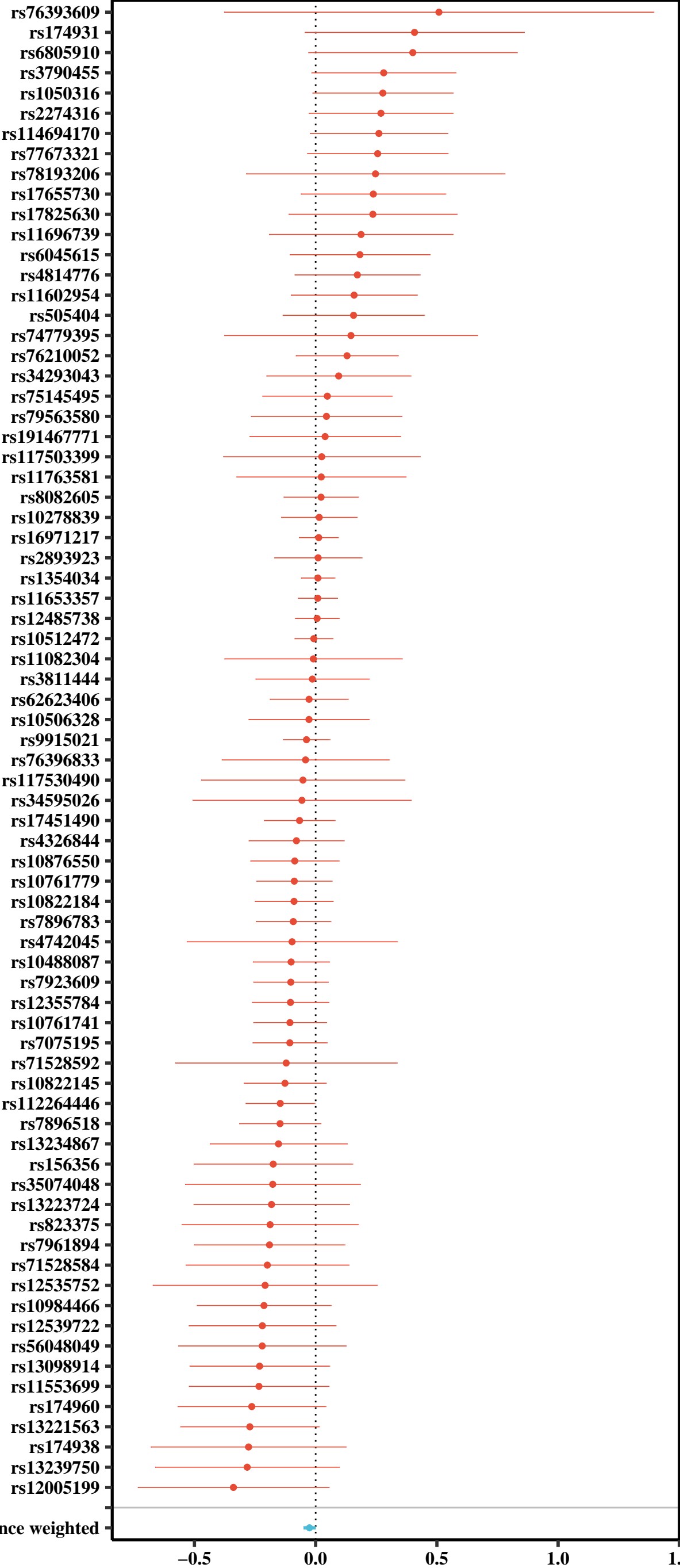

Supplement: Supplementary file 2 [file DataSheet2.zip › Supplementary Figure 2/MTURN.pdf]

# MR effect size for NR3C1 on PCa

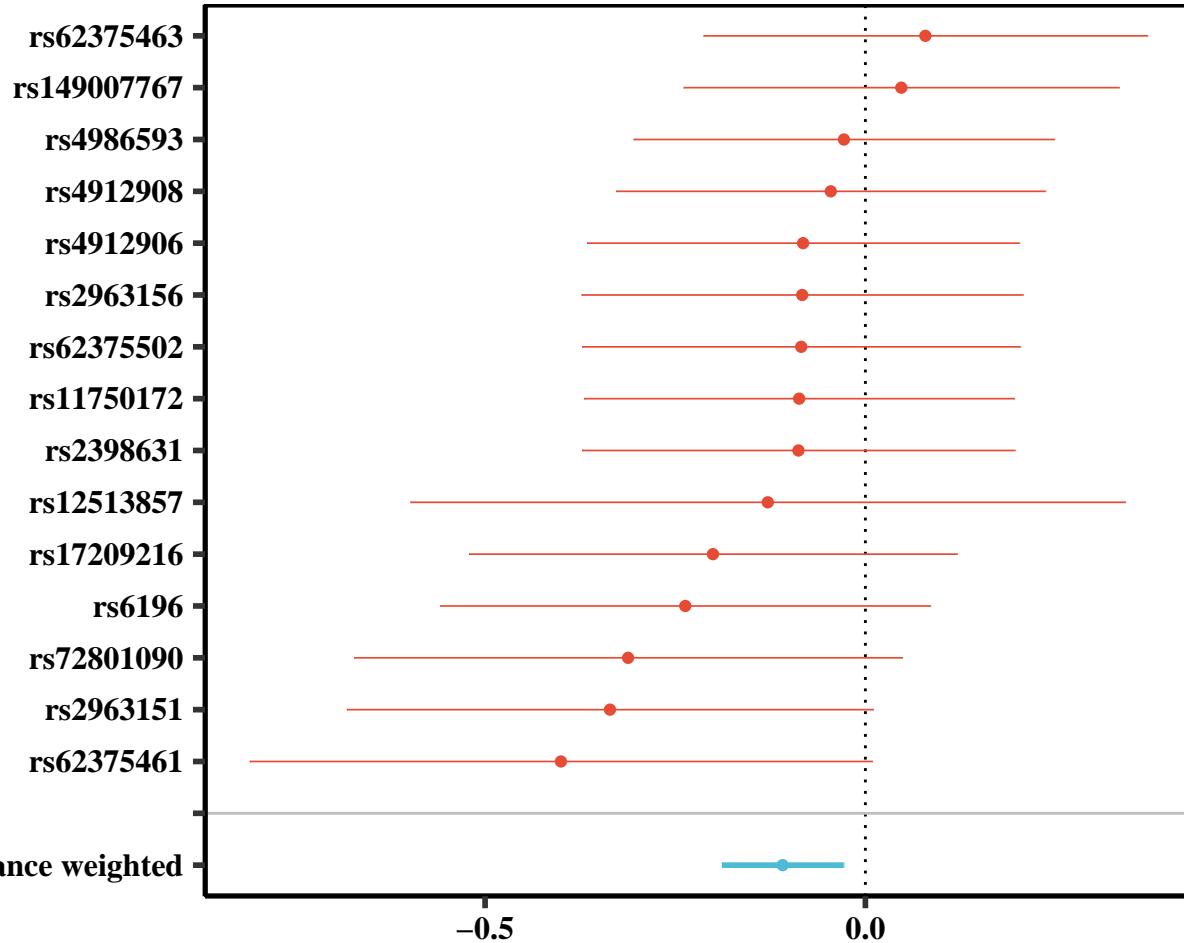

Supplement: Supplementary file 2 [file DataSheet2.zip › Supplementary Figure 2/NR3C1.pdf]

MR effect size for PPP3CA on PCa

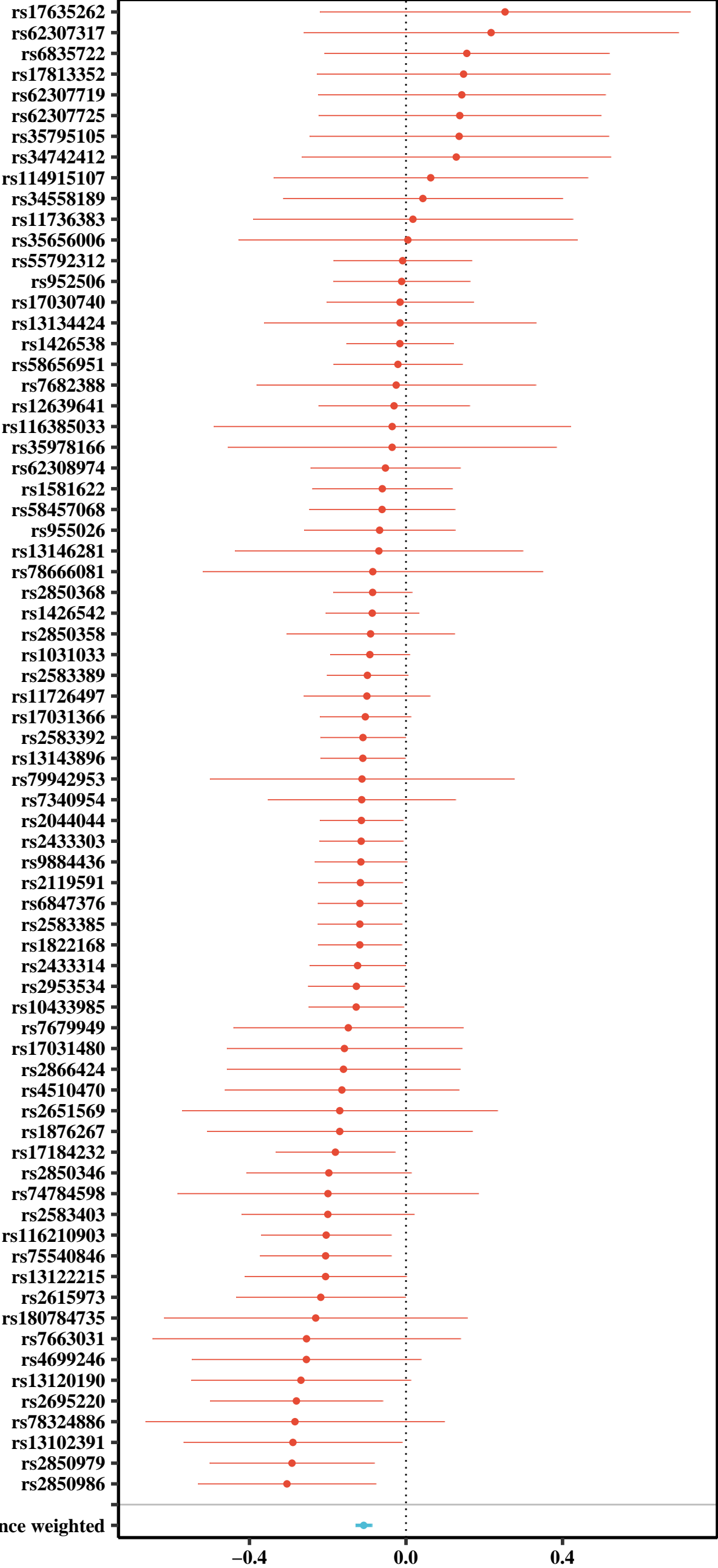

All – Inverse variance weighted

Supplement: Supplementary file 2 [file DataSheet2.zip › Supplementary Figure 2/PPP3CA.pdf]

# MR effect size for RACGAP1 on PCa

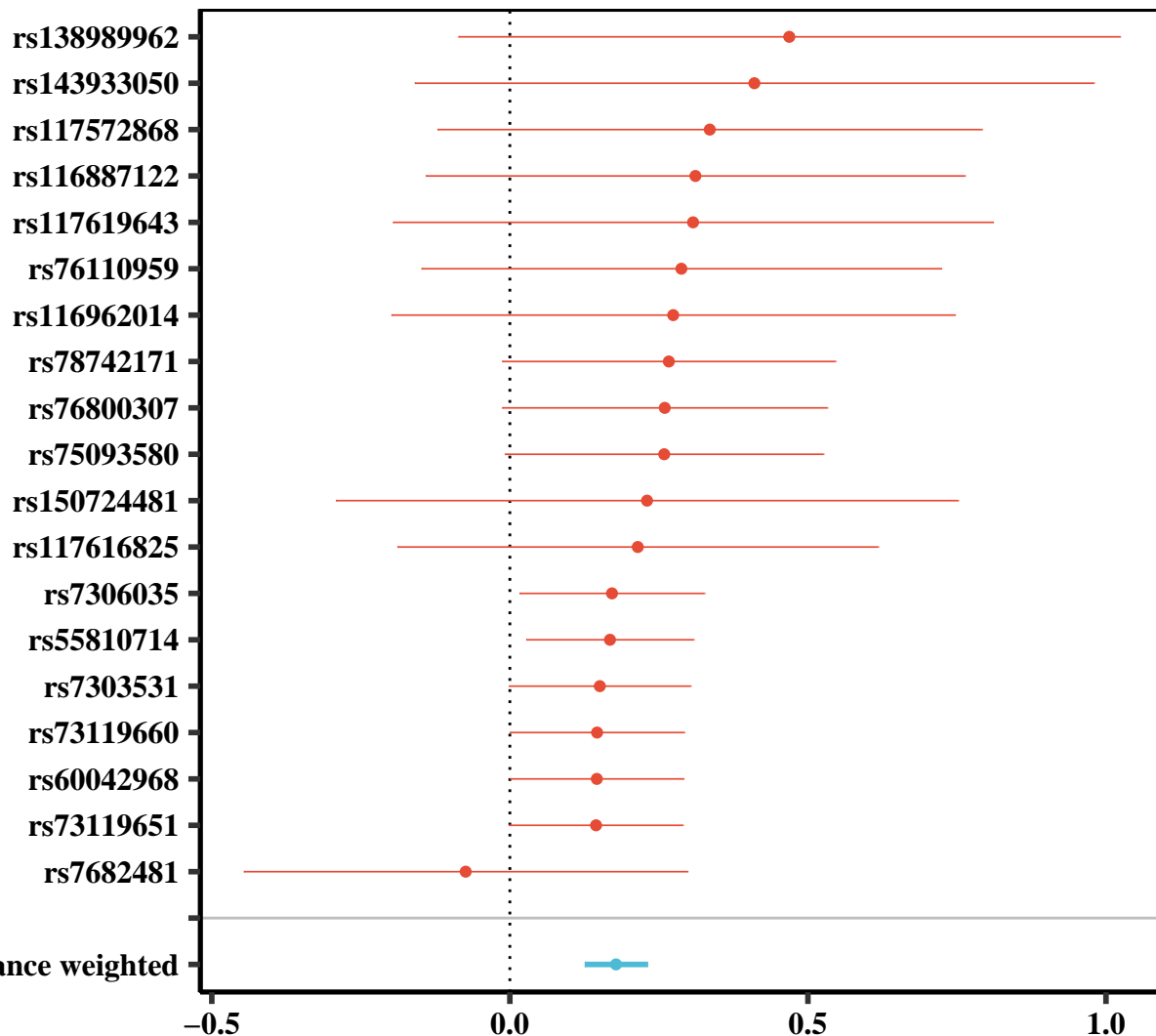

Supplement: Supplementary file 2 [file DataSheet2.zip › Supplementary Figure 2/RACGAP1.pdf]

# MR effect size for STAT5B on PCa

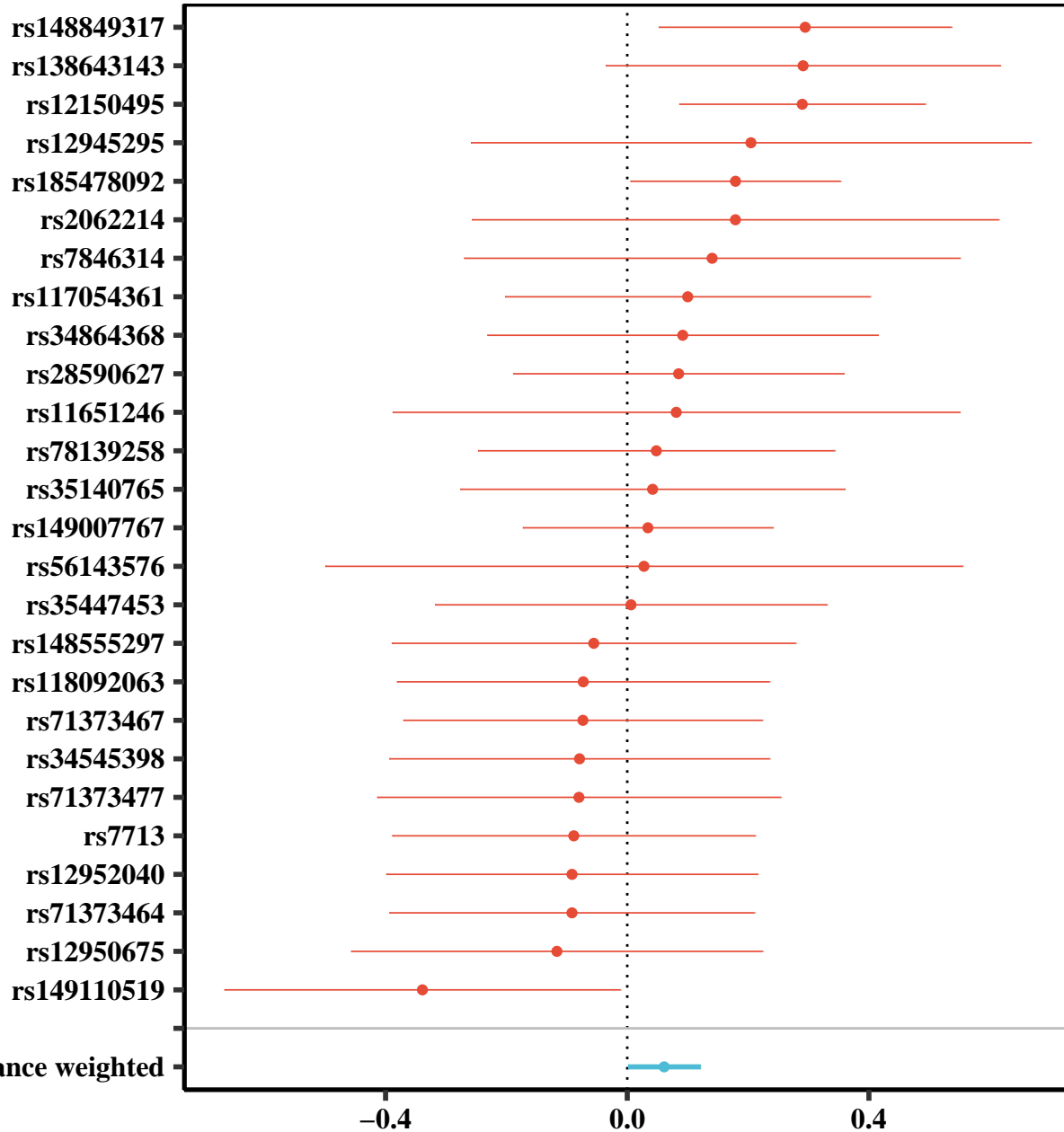

Supplement: Supplementary file 2 [file DataSheet2.zip › Supplementary Figure 2/STAT5B.pdf]

# MR effect size for TLR2 on PCa

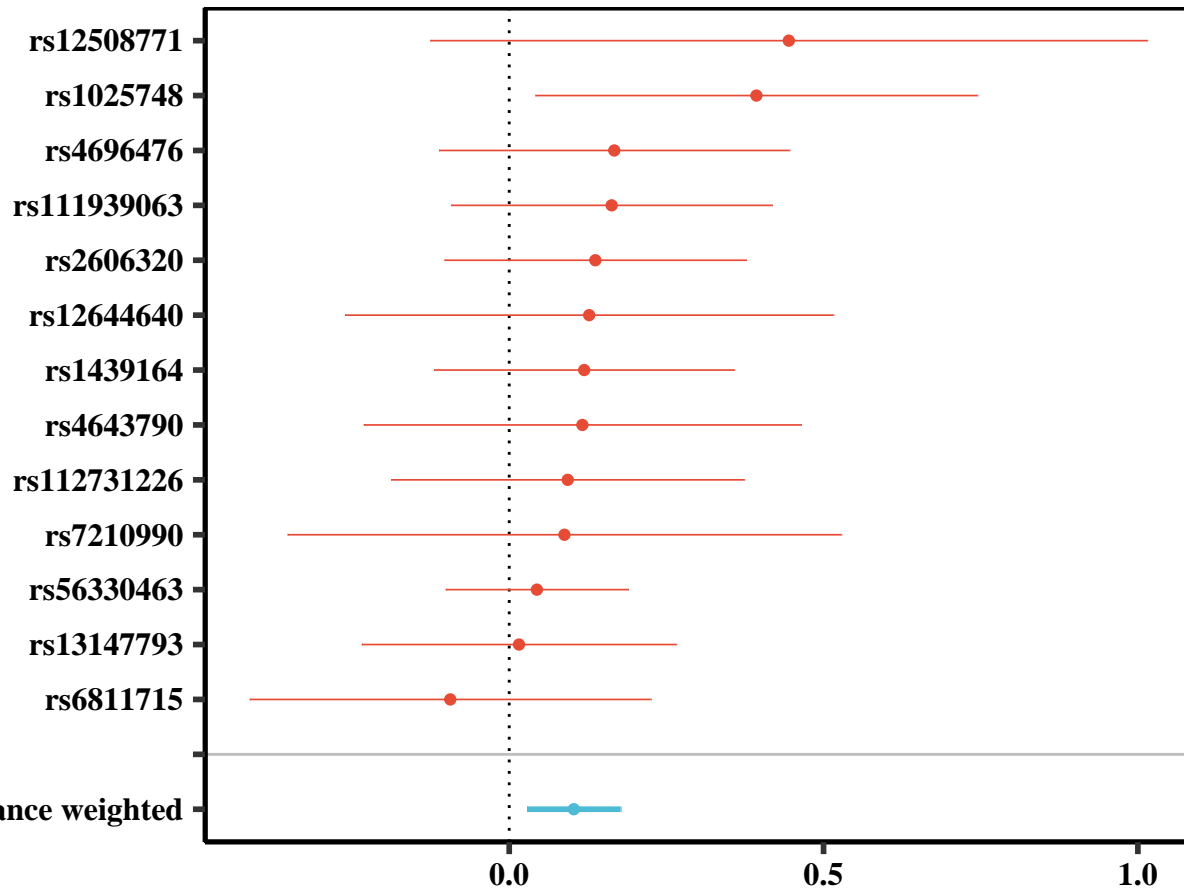

Supplement: Supplementary file 2 [file DataSheet2.zip › Supplementary Figure 2/TLR2.pdf]

# MR effect size for TLR3 on PCa

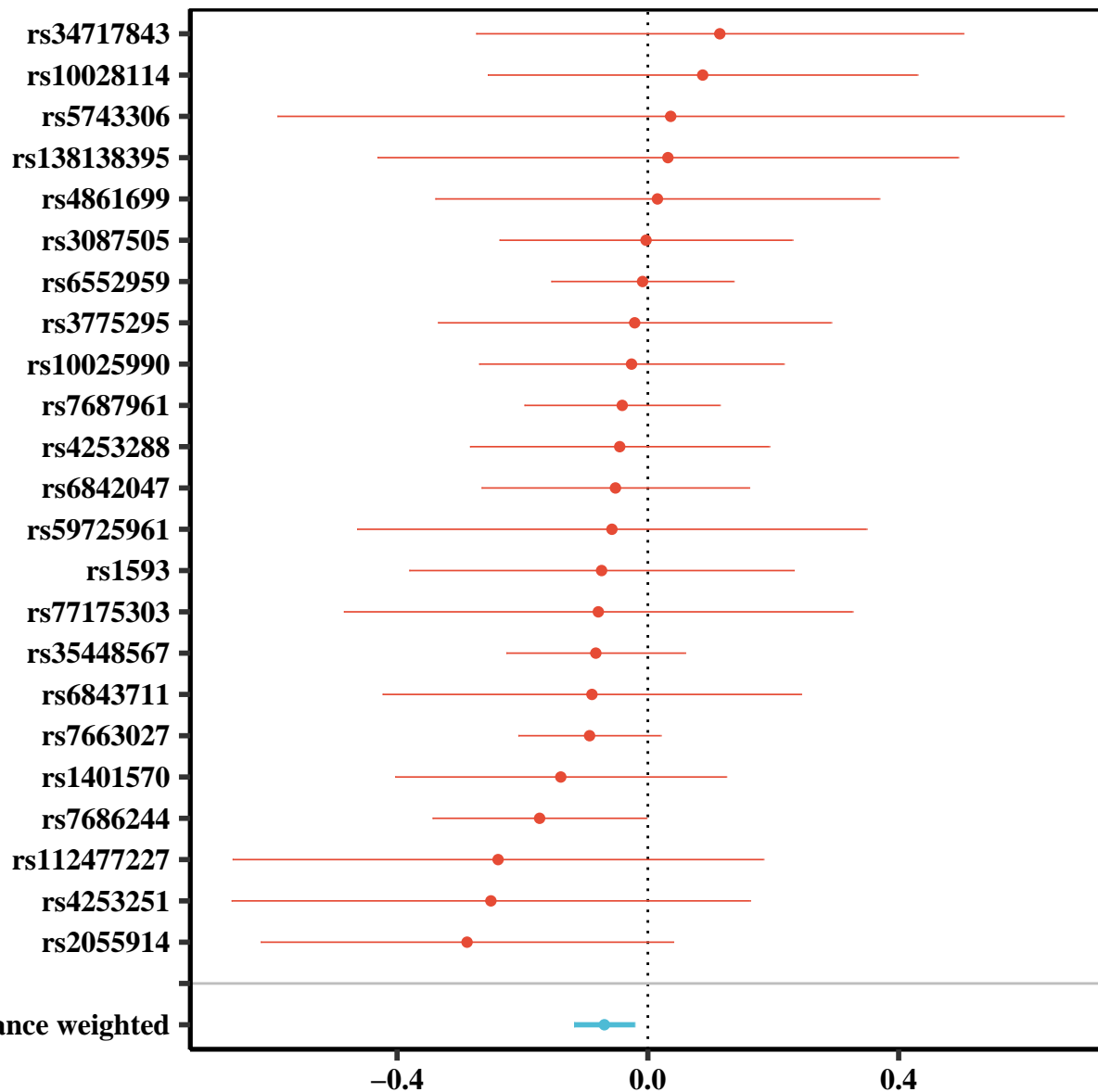

Supplement: Supplementary file 2 [file DataSheet2.zip › Supplementary Figure 2/TLR3.pdf]

# MR of ACTN1

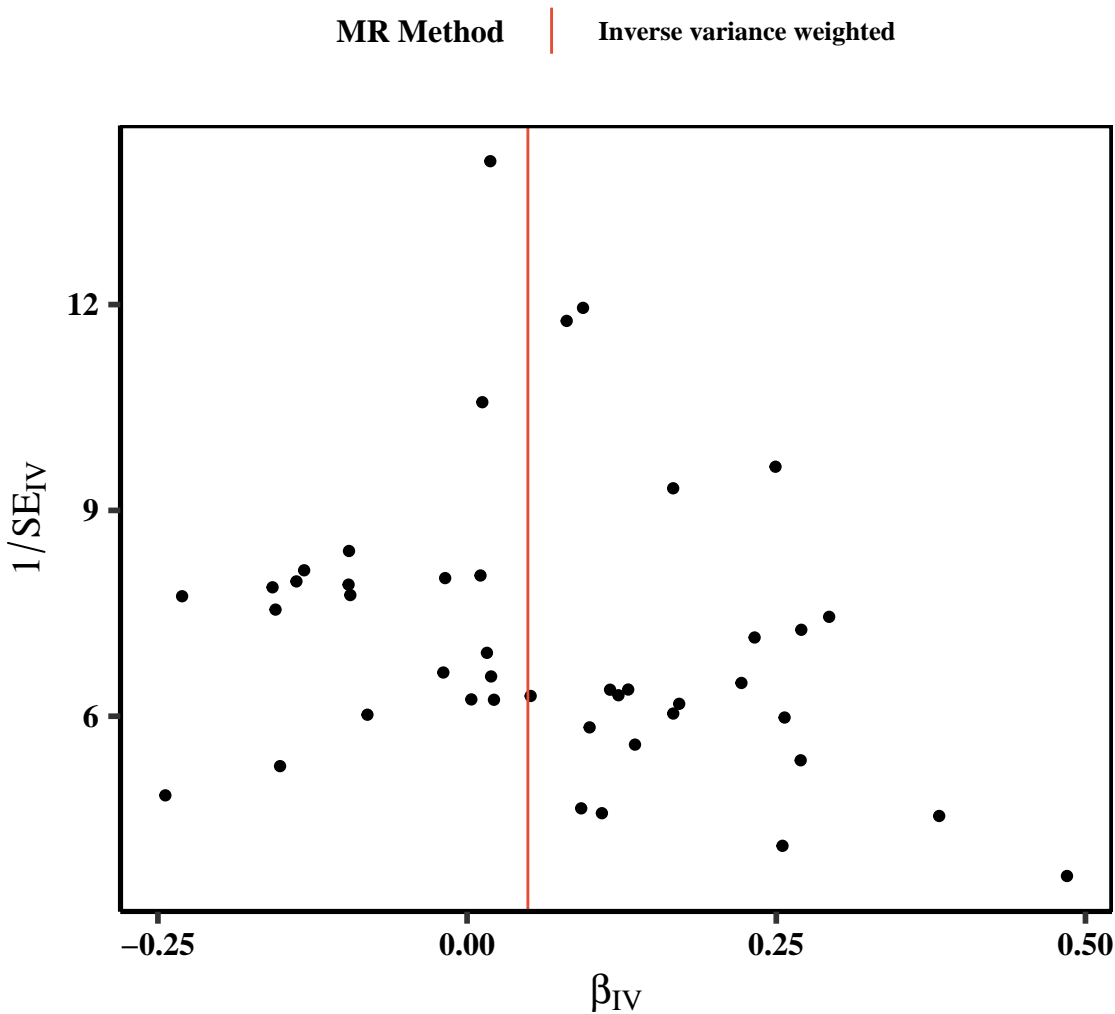

Supplement: Supplementary file 3 [file DataSheet3.zip › Supplementary Figure 3/ACTN1.pdf]

# MR of BATF3

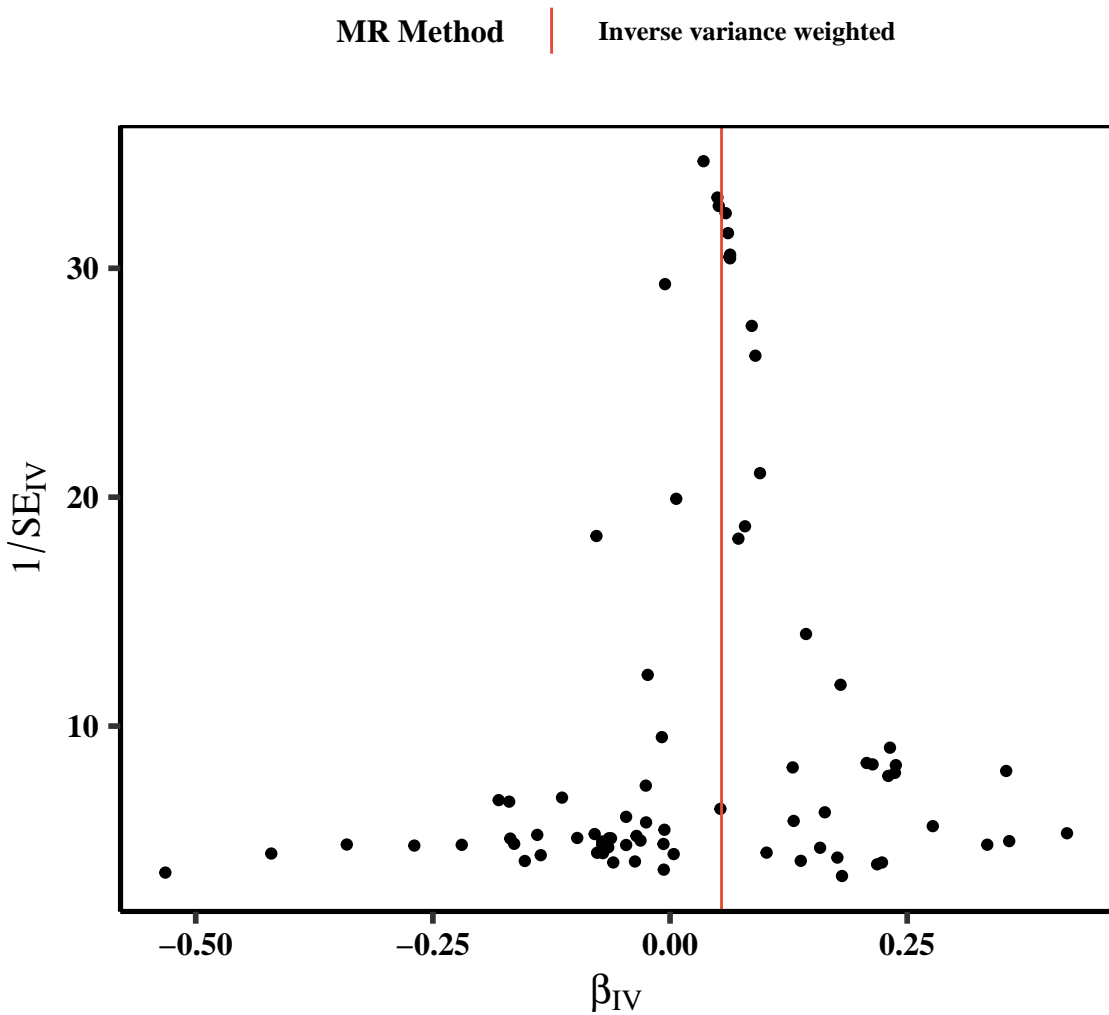

Supplement: Supplementary file 3 [file DataSheet3.zip › Supplementary Figure 3/BATF3.pdf]

# MR of BMP2

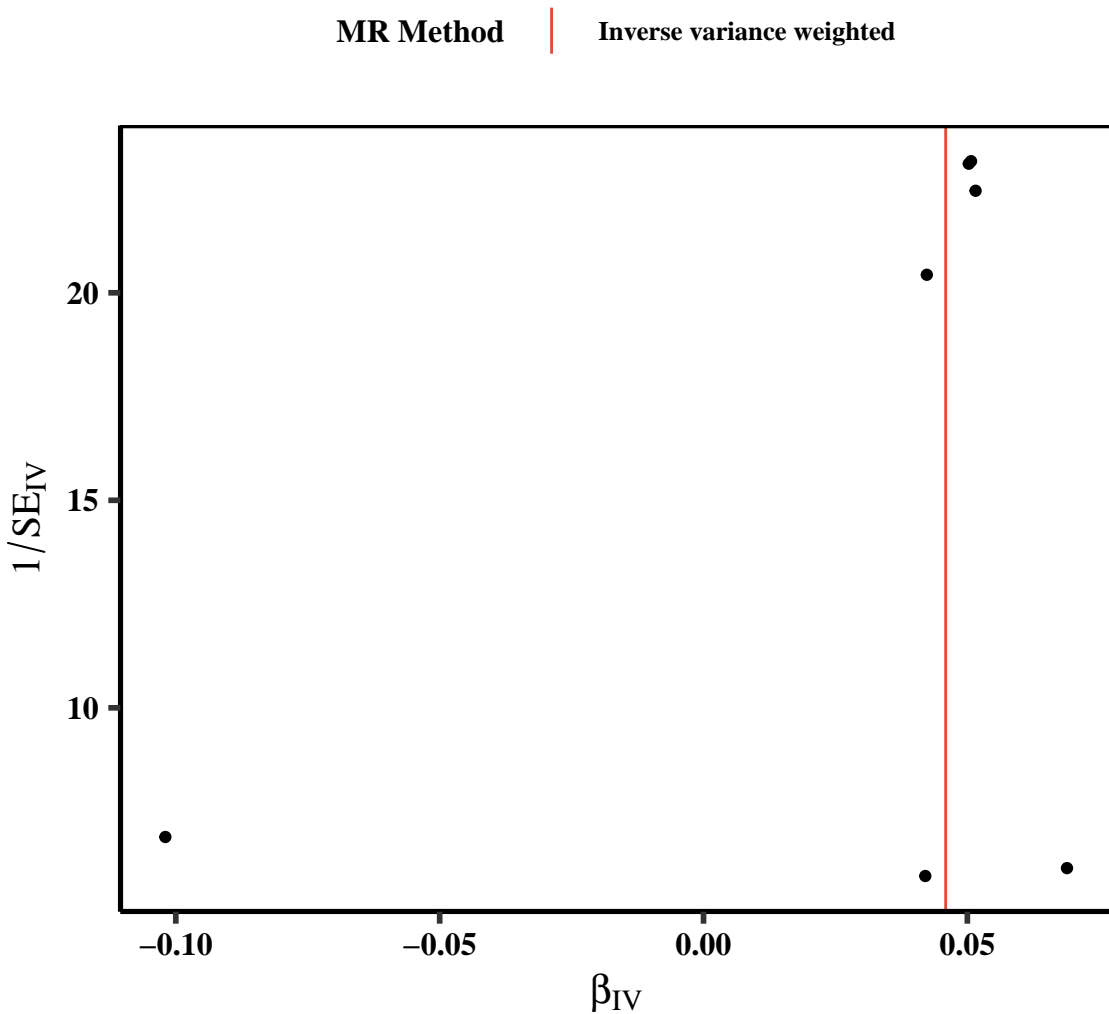

Supplement: Supplementary file 3 [file DataSheet3.zip › Supplementary Figure 3/BMP2.pdf]

# MR of CD109

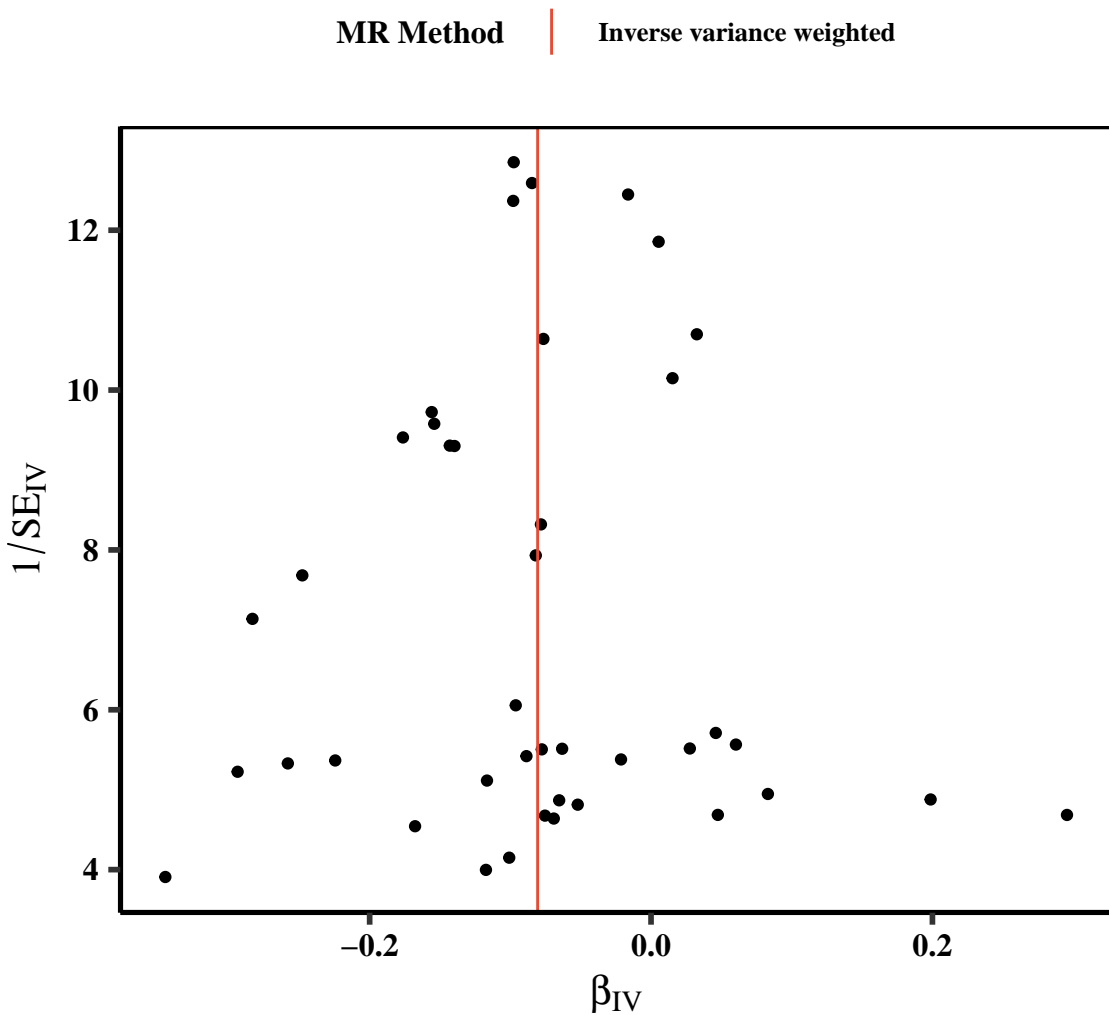

Supplement: Supplementary file 3 [file DataSheet3.zip › Supplementary Figure 3/CD109.pdf]

# MR of FASN

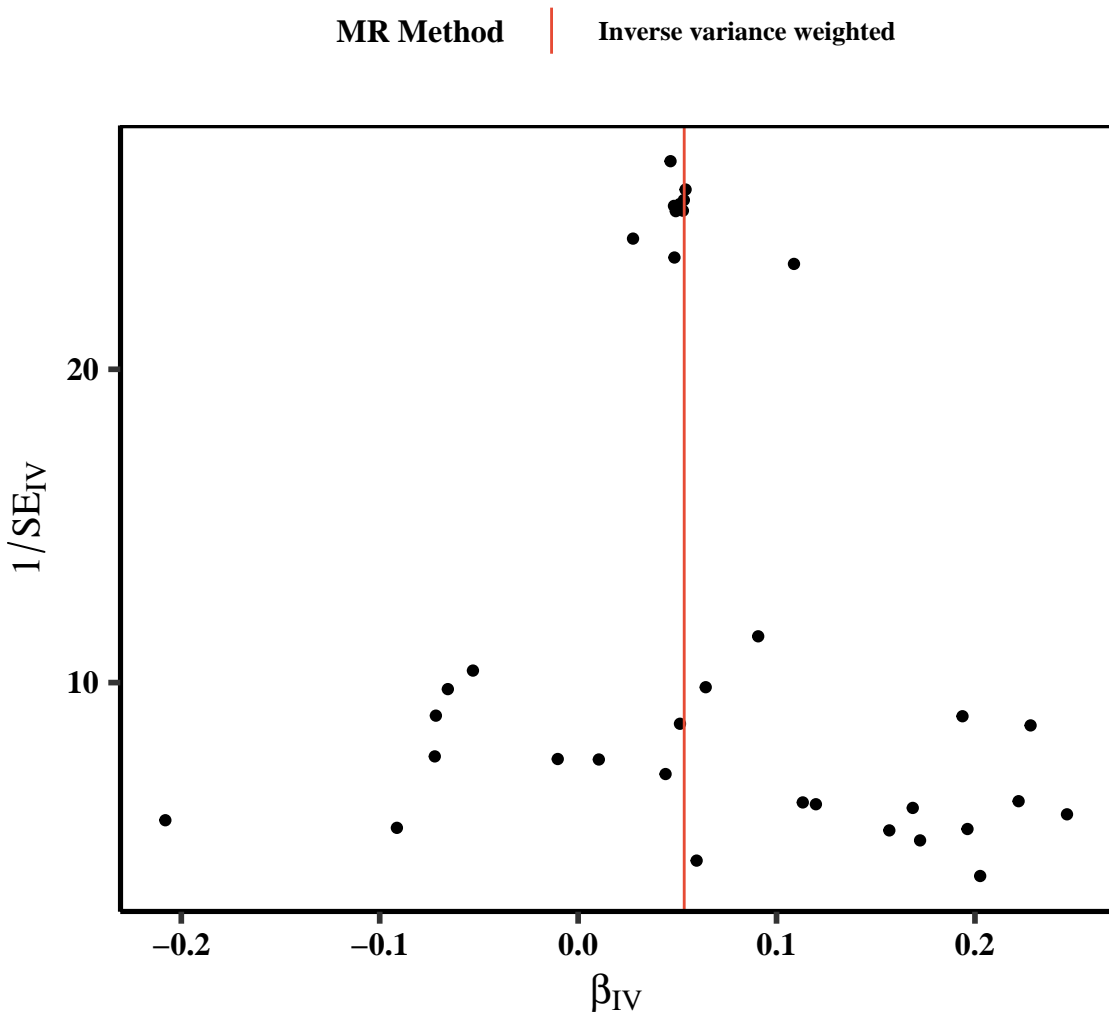

Supplement: Supplementary file 3 [file DataSheet3.zip › Supplementary Figure 3/FASN.pdf]

# MR of FBN1

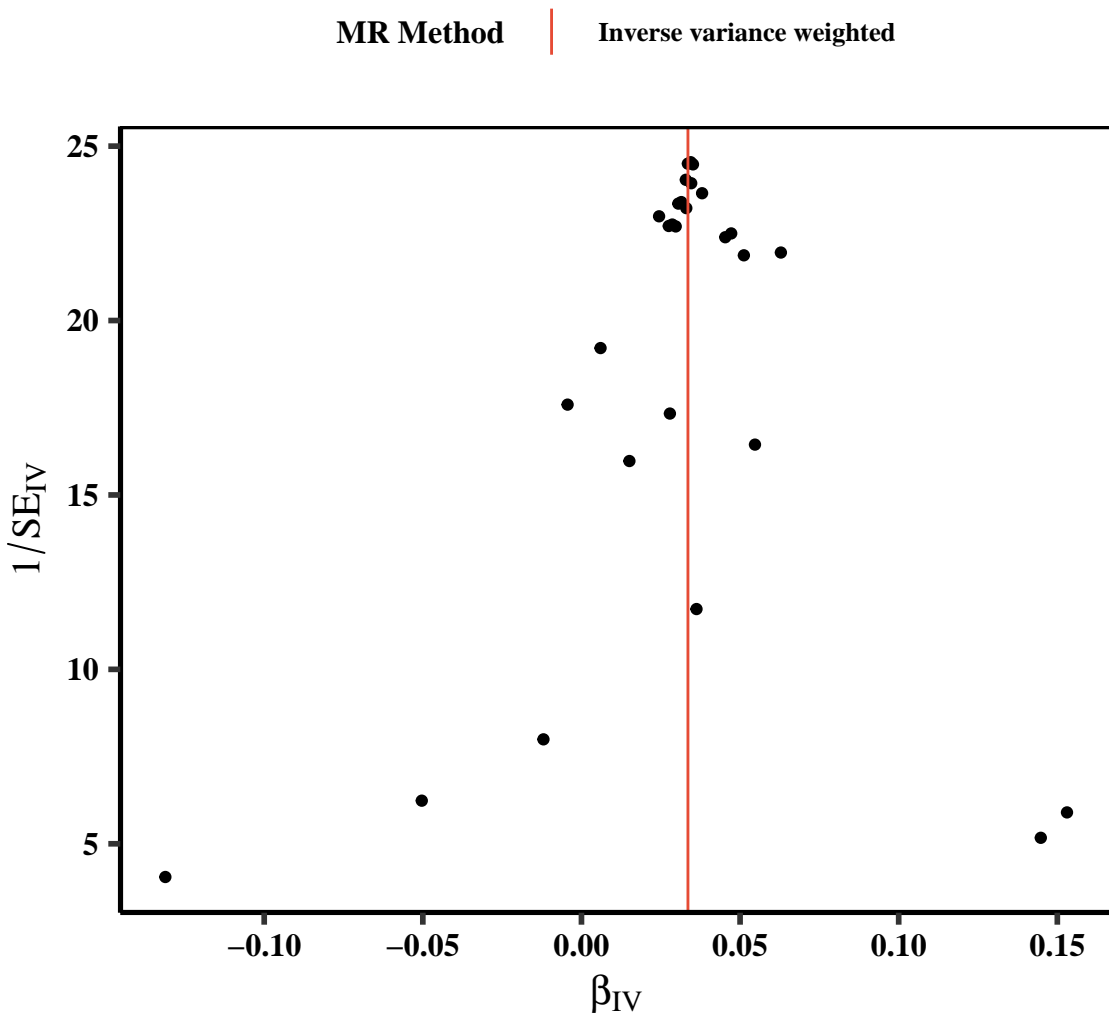

Supplement: Supplementary file 3 [file DataSheet3.zip › Supplementary Figure 3/FBN1.pdf]

# MR of FLNA

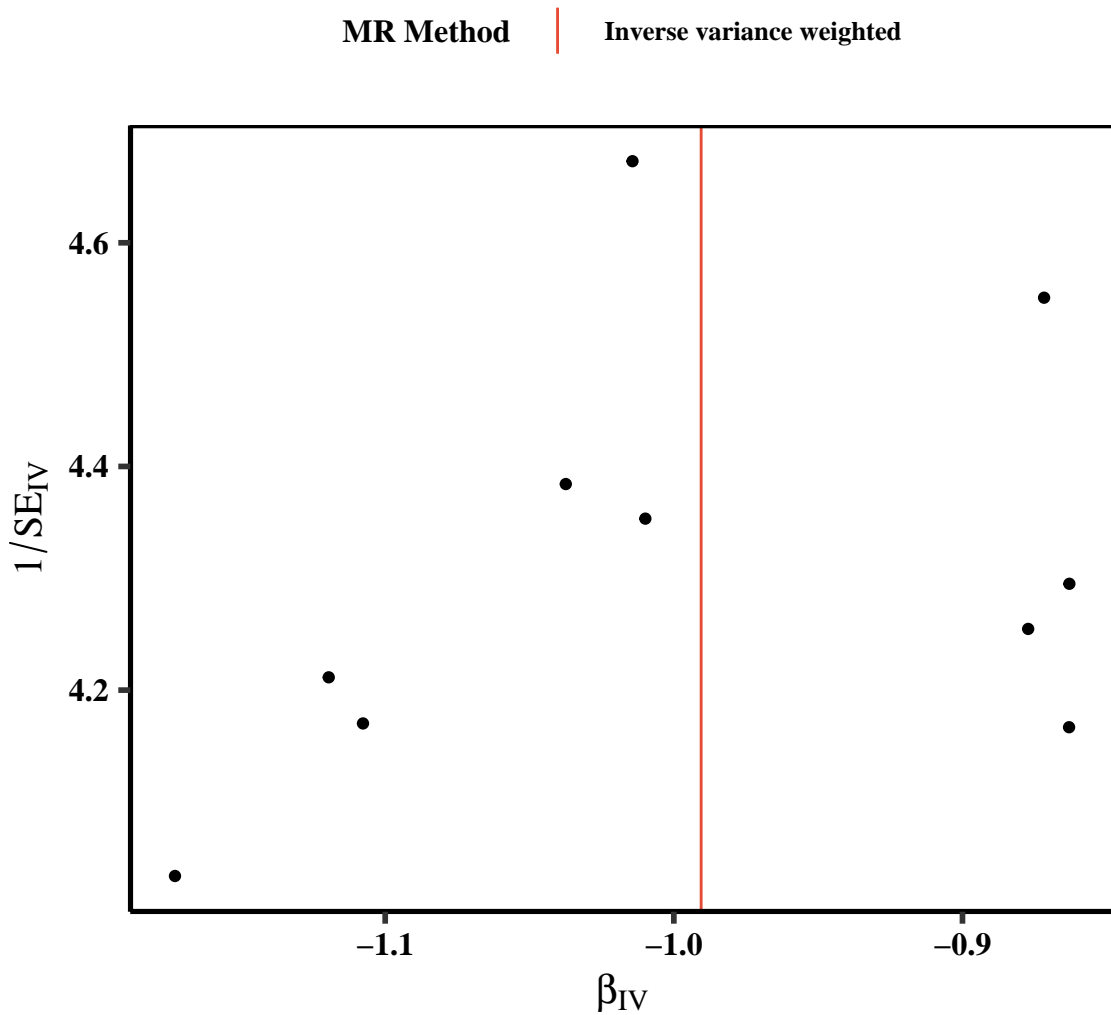

Supplement: Supplementary file 3 [file DataSheet3.zip › Supplementary Figure 3/FLNA.pdf]

# MR of GATA3

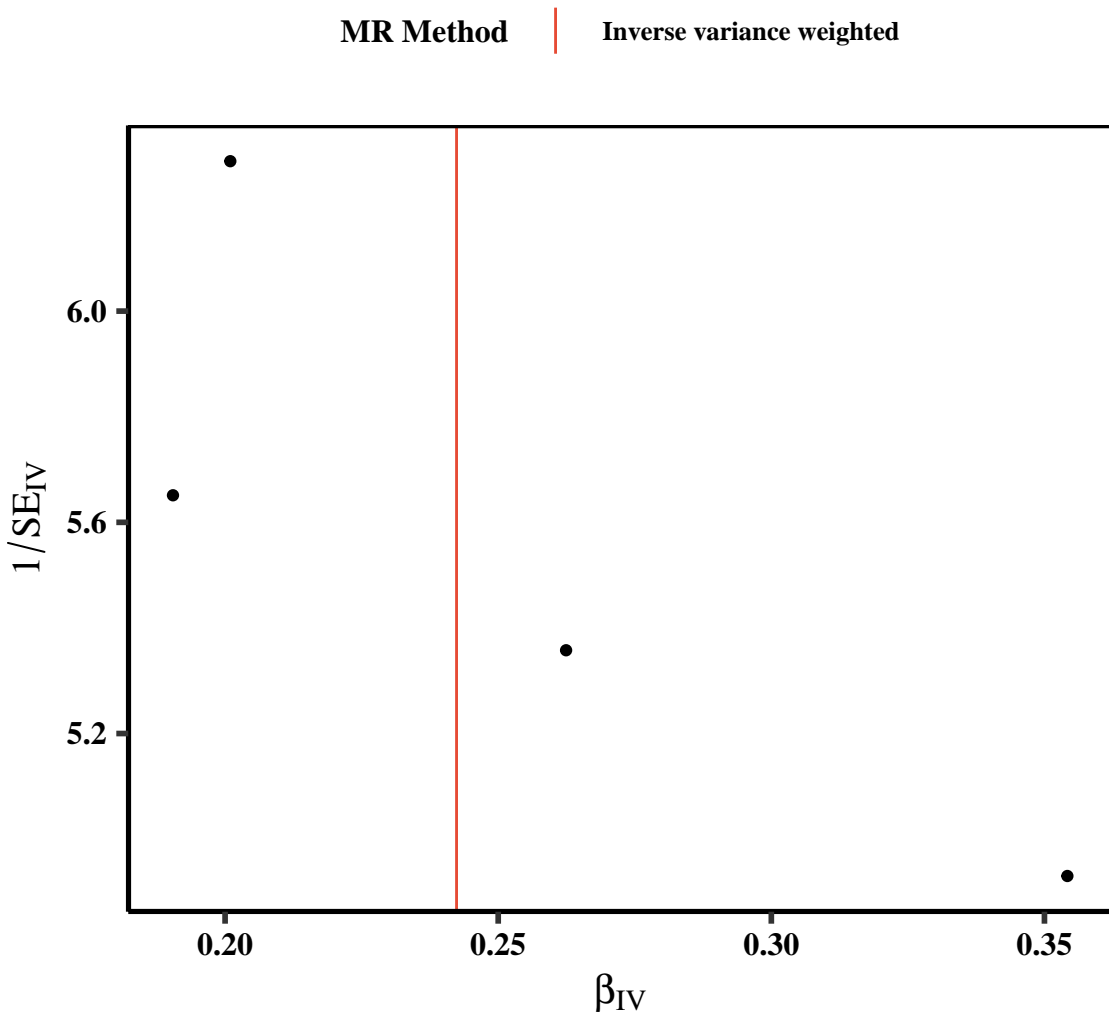

Supplement: Supplementary file 3 [file DataSheet3.zip › Supplementary Figure 3/GATA3.pdf]

# MR of GP5

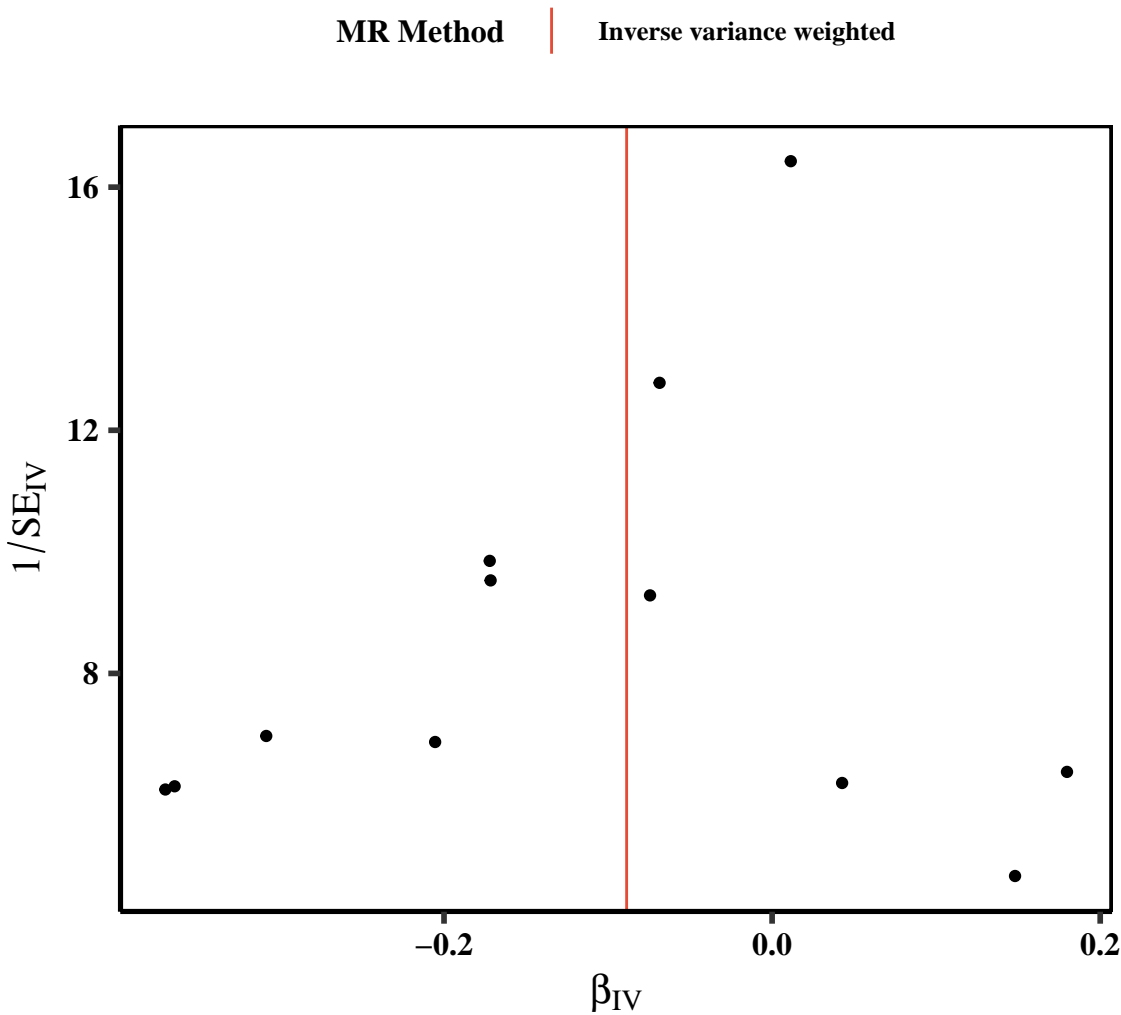

Supplement: Supplementary file 3 [file DataSheet3.zip › Supplementary Figure 3/GP5.pdf]

# MR of IFI16

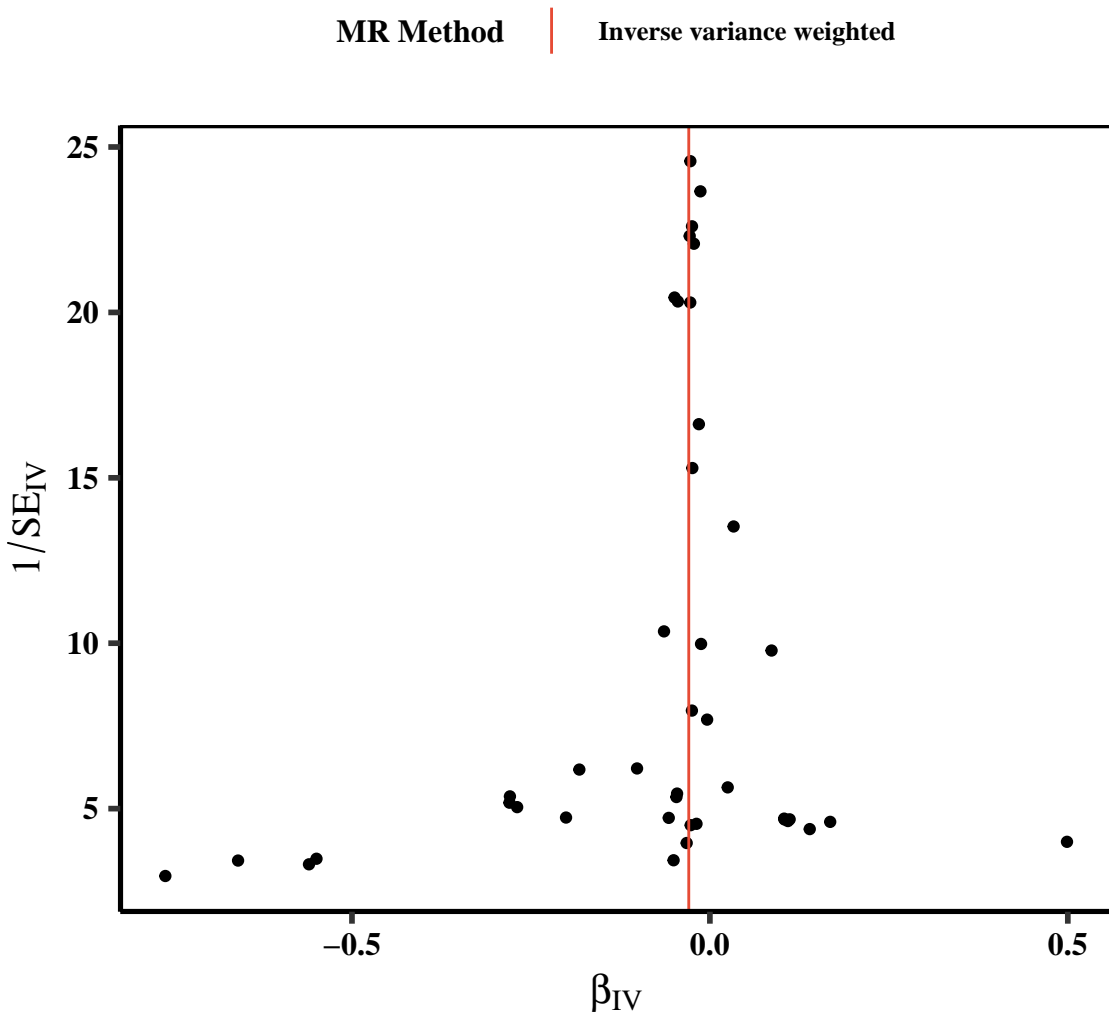

Supplement: Supplementary file 3 [file DataSheet3.zip › Supplementary Figure 3/IFI16.pdf]

# MR of IL1RL1

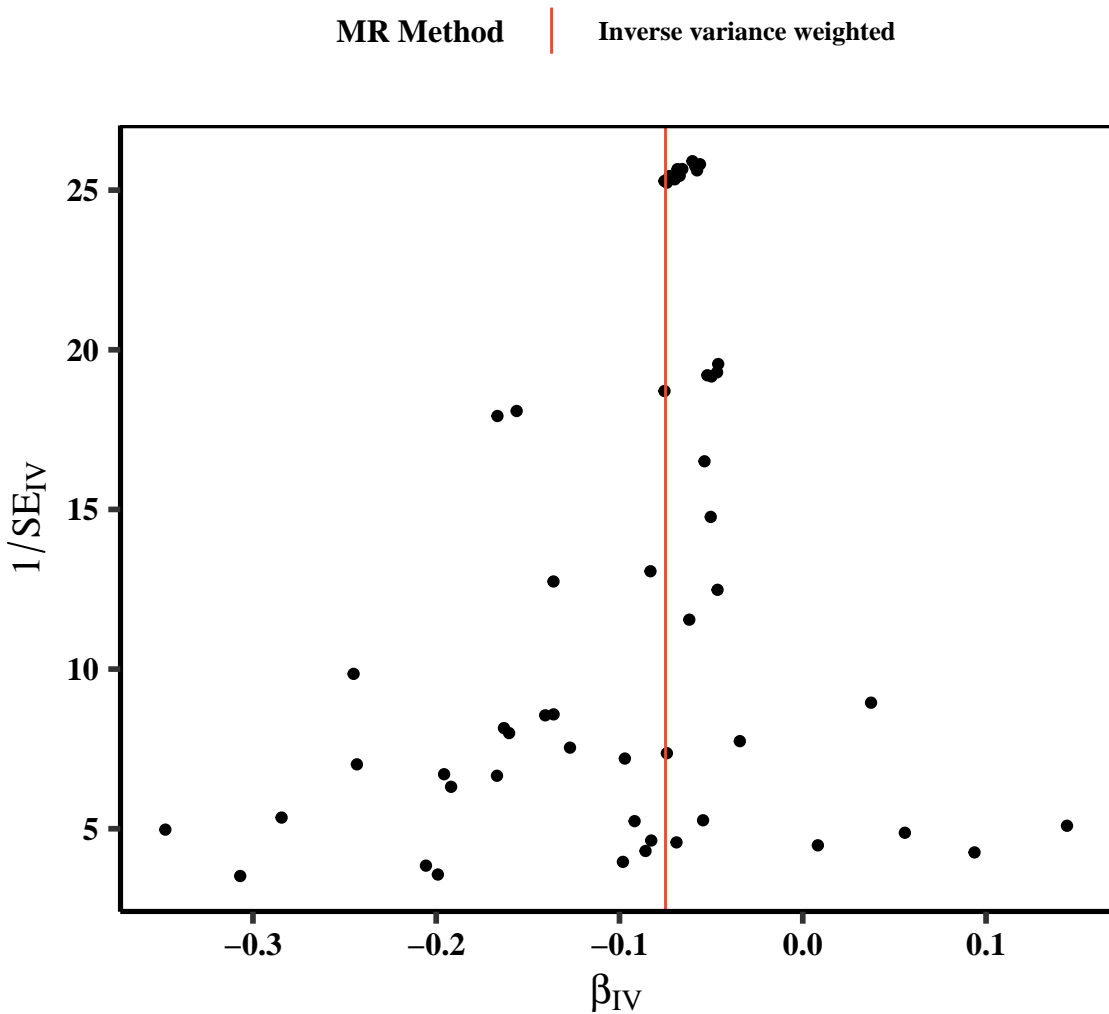

Supplement: Supplementary file 3 [file DataSheet3.zip › Supplementary Figure 3/IL1RL1.pdf]

# MR of ISG15

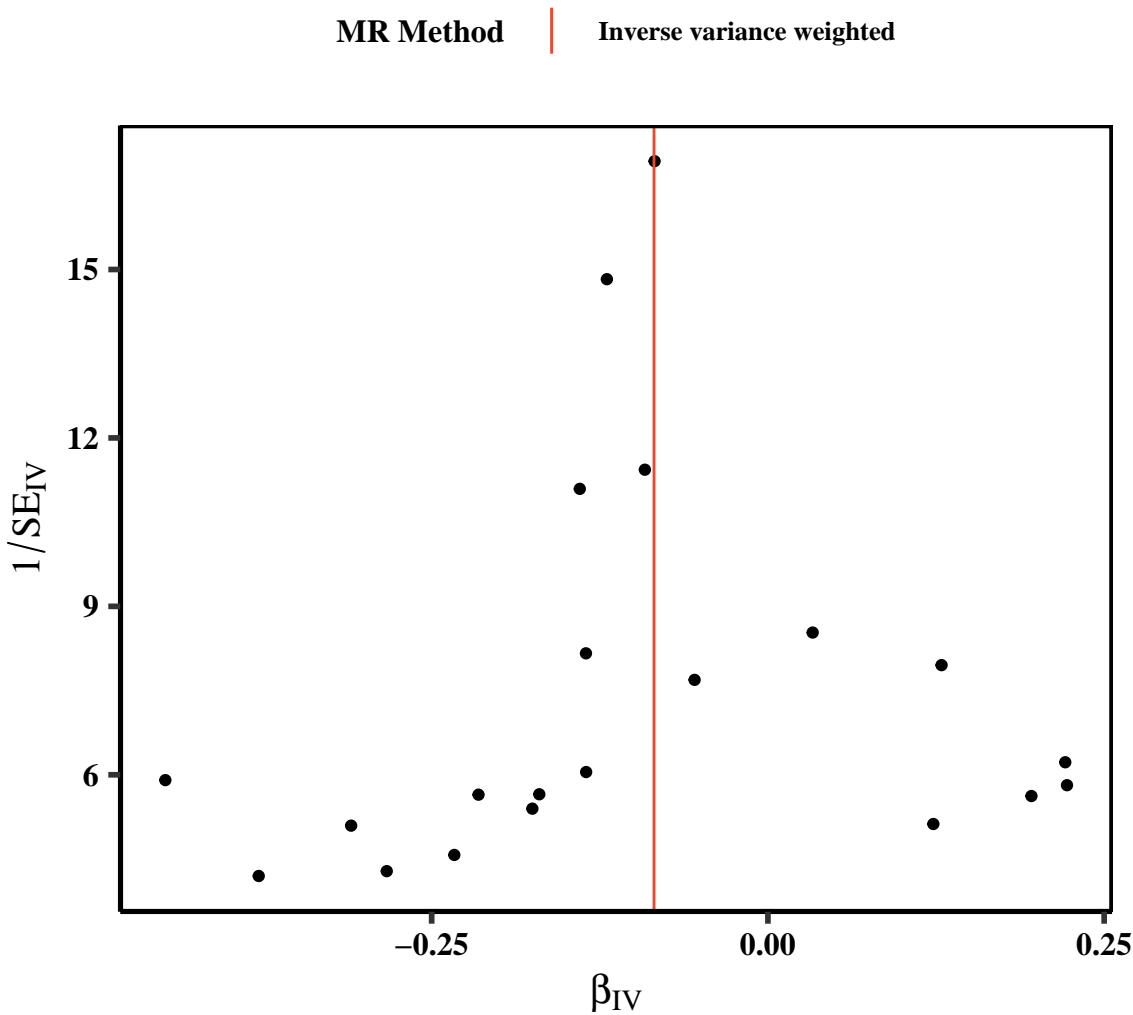

Supplement: Supplementary file 3 [file DataSheet3.zip › Supplementary Figure 3/ISG15.pdf]

# MR of KIT

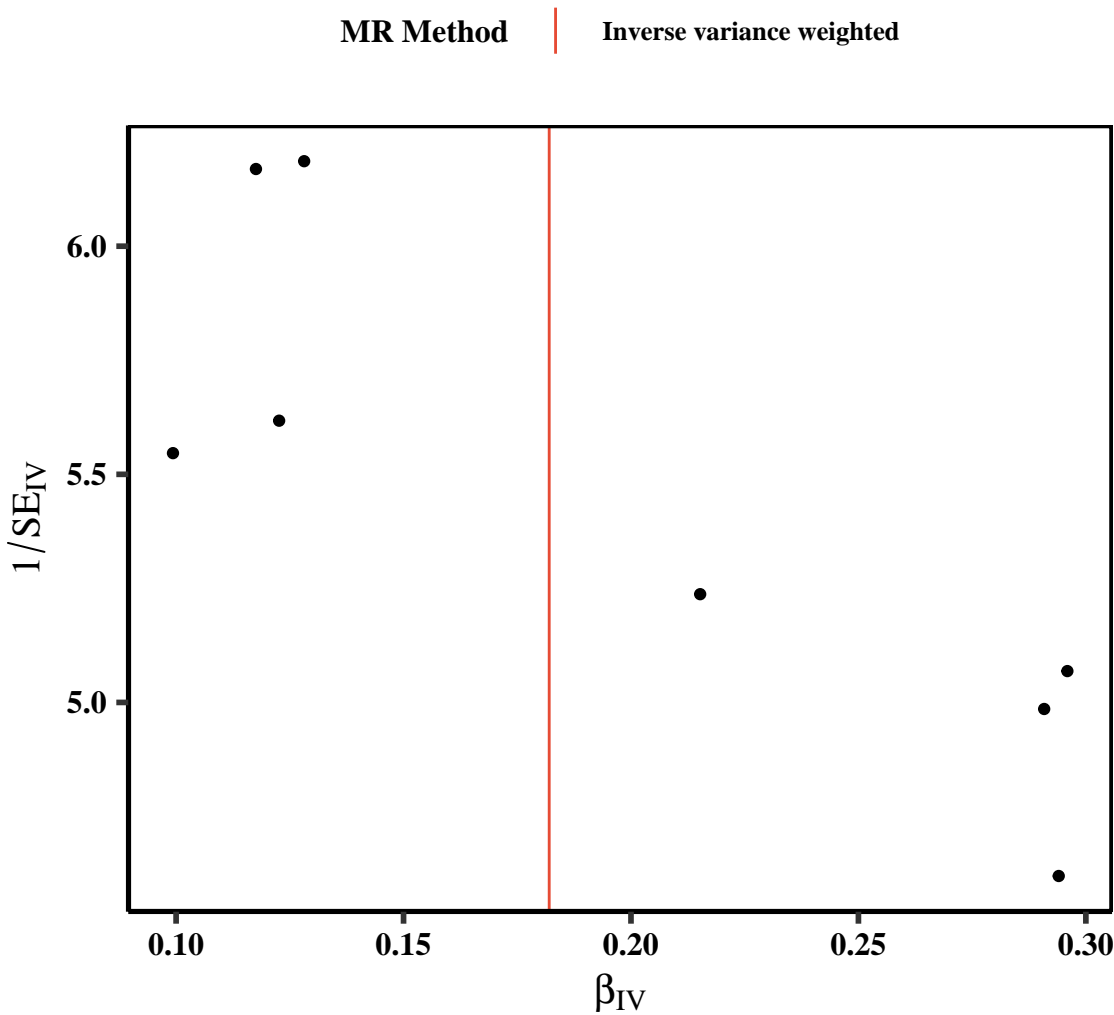

Supplement: Supplementary file 3 [file DataSheet3.zip › Supplementary Figure 3/KIT.pdf]

# MR of KLF10

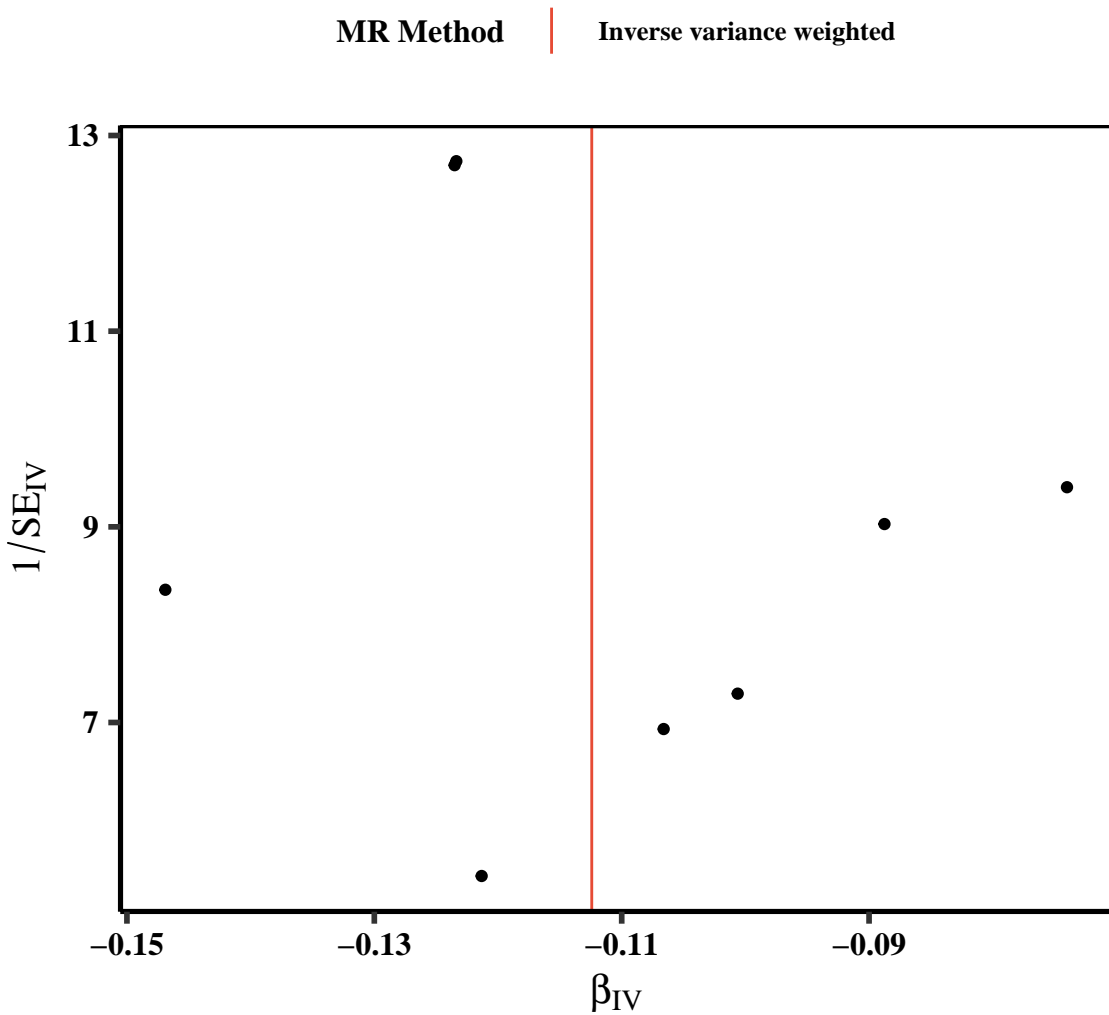

Supplement: Supplementary file 3 [file DataSheet3.zip › Supplementary Figure 3/KLF10.pdf]

# MR of LOX

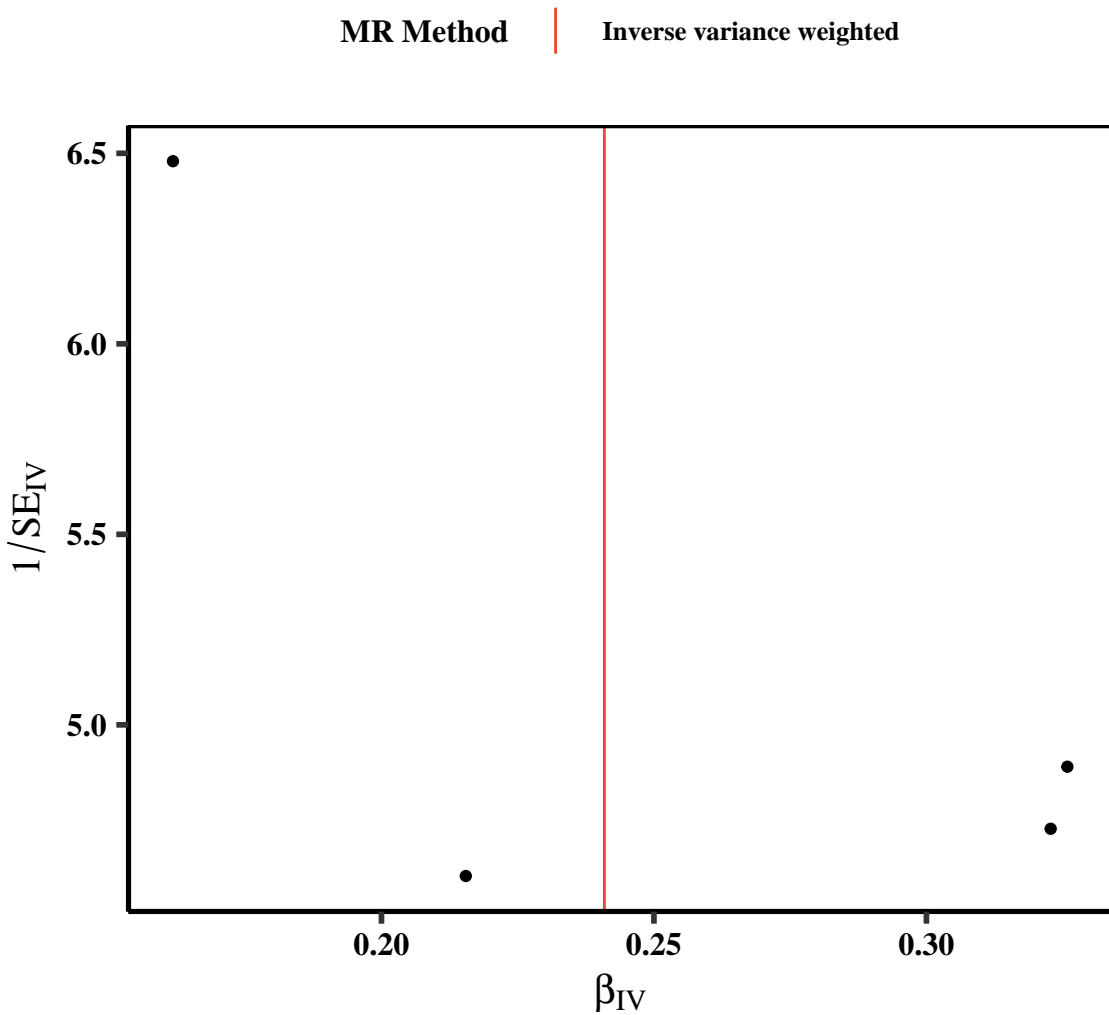

Supplement: Supplementary file 3 [file DataSheet3.zip › Supplementary Figure 3/LOX.pdf]

# MR of MITF

MR Method

Inverse variance weighted

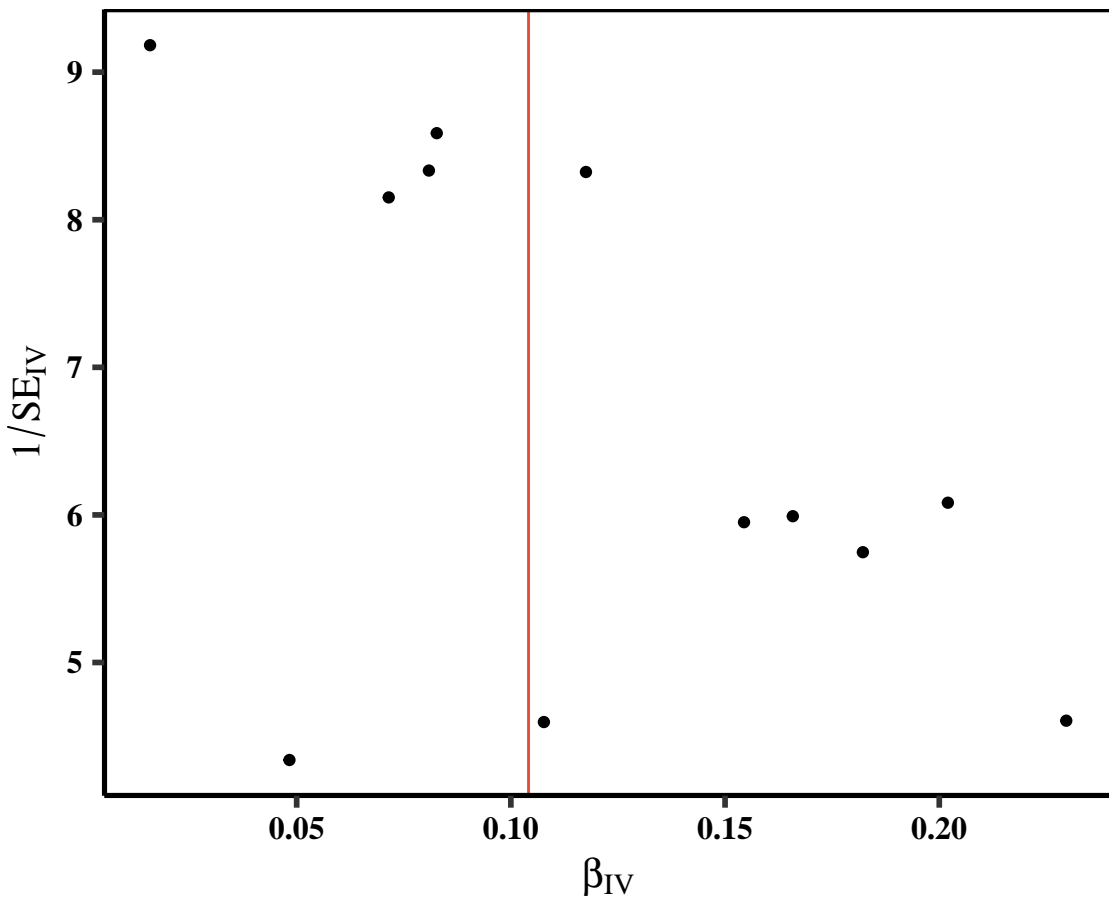

Supplement: Supplementary file 3 [file DataSheet3.zip › Supplementary Figure 3/MITF.pdf]

# MR of MTURN

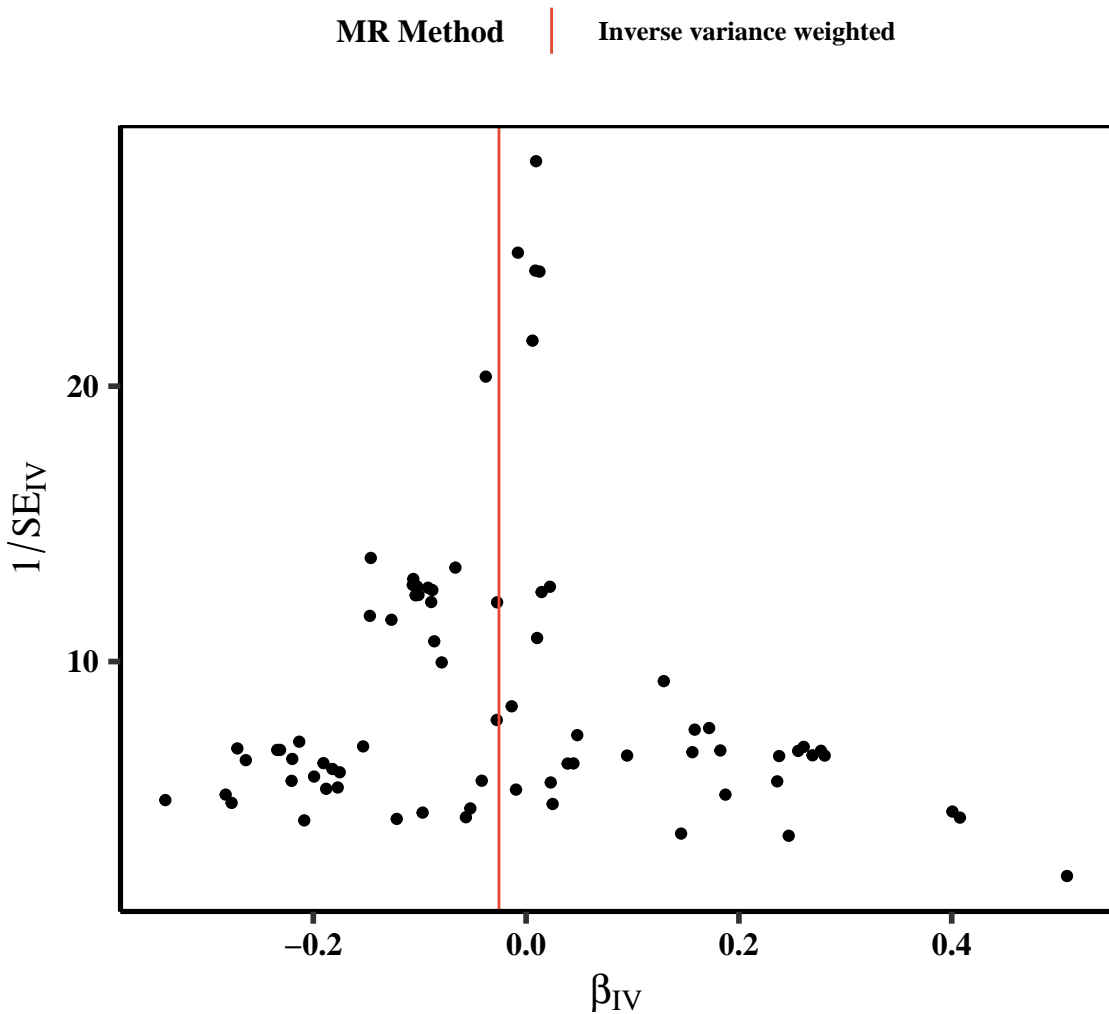

Supplement: Supplementary file 3 [file DataSheet3.zip › Supplementary Figure 3/MTURN.pdf]

# MR of NR3C1

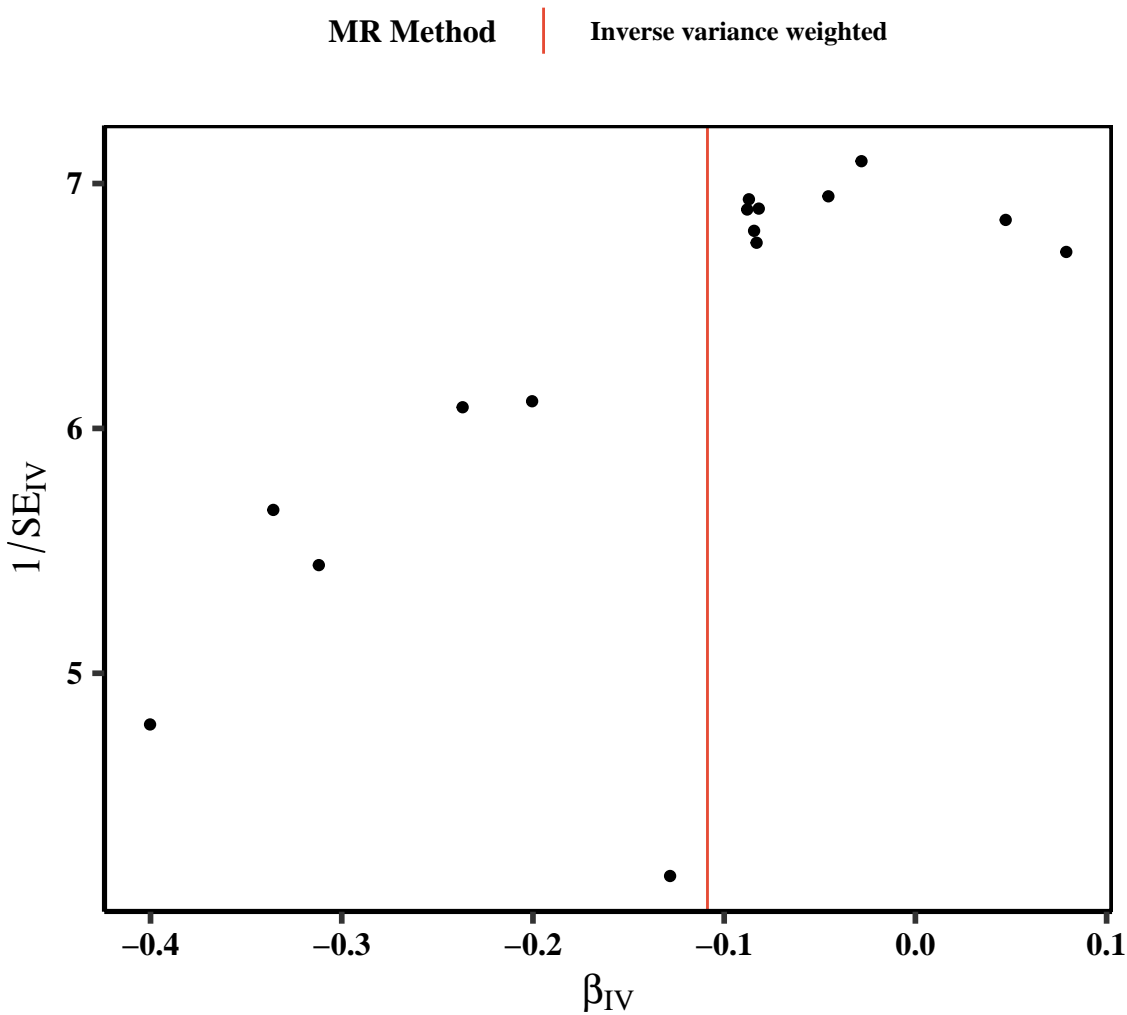

Supplement: Supplementary file 3 [file DataSheet3.zip › Supplementary Figure 3/NR3C1.pdf]

# MR of PPP3CA

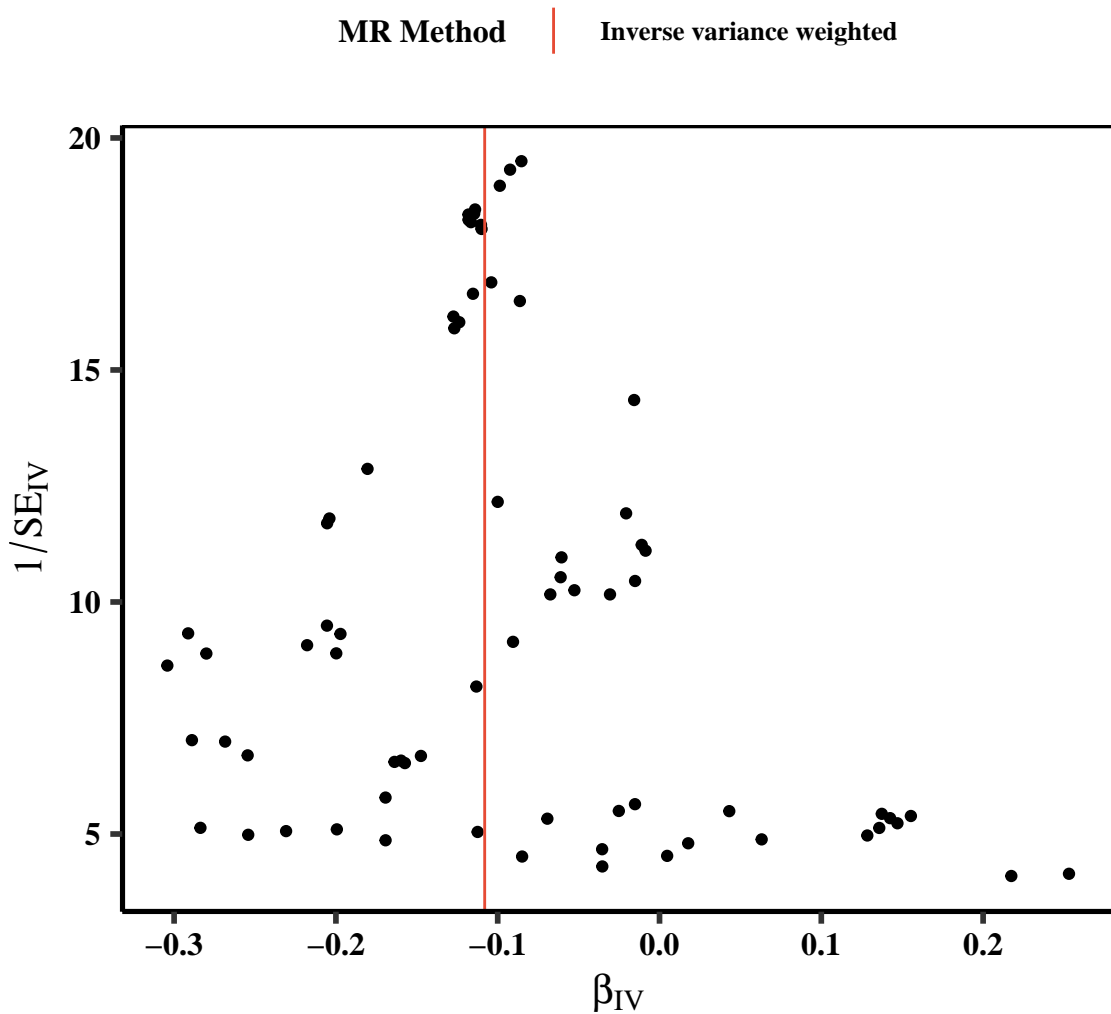

Supplement: Supplementary file 3 [file DataSheet3.zip › Supplementary Figure 3/PPP3CA.pdf]

# MR of RACGAP1

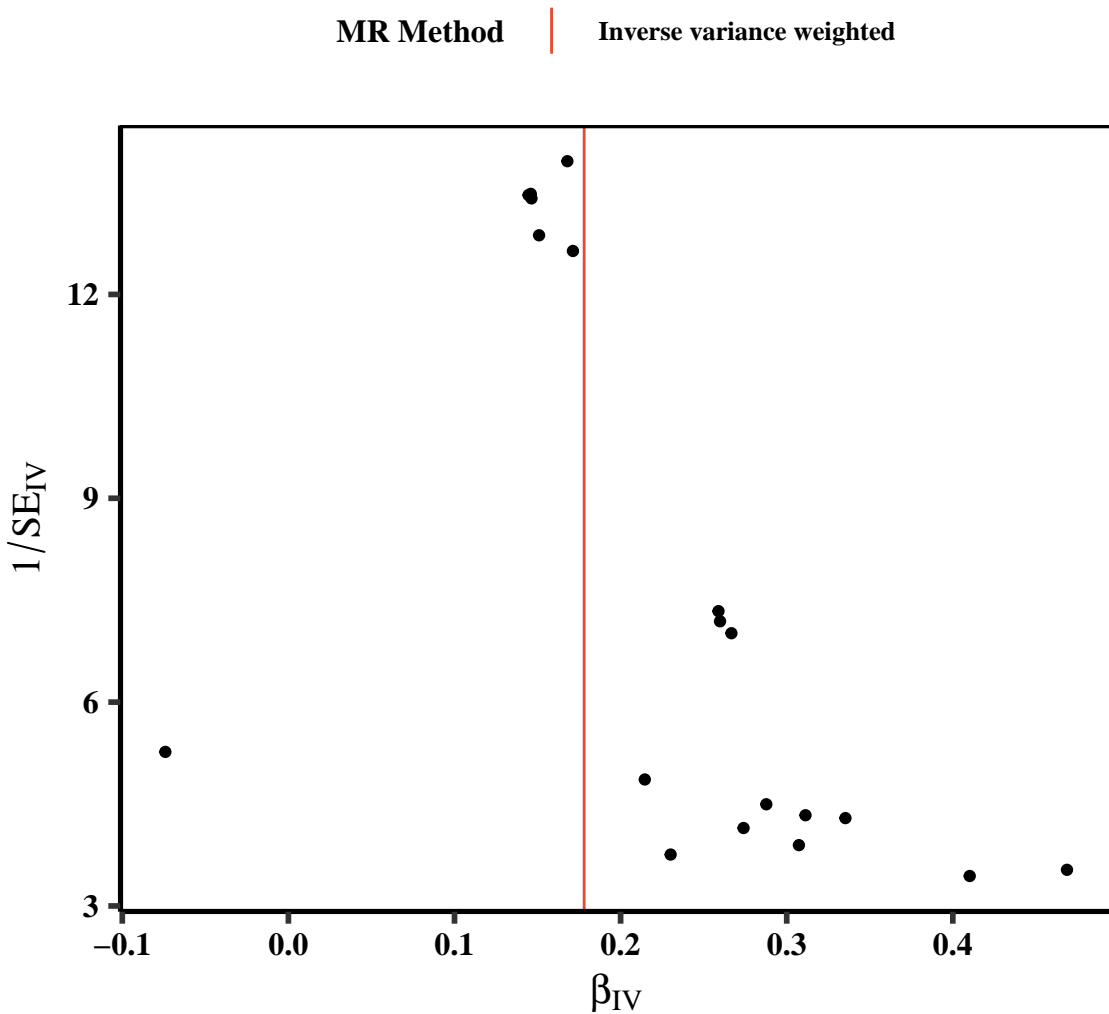

Supplement: Supplementary file 3 [file DataSheet3.zip › Supplementary Figure 3/RACGAP1.pdf]

# MR of STAT5B

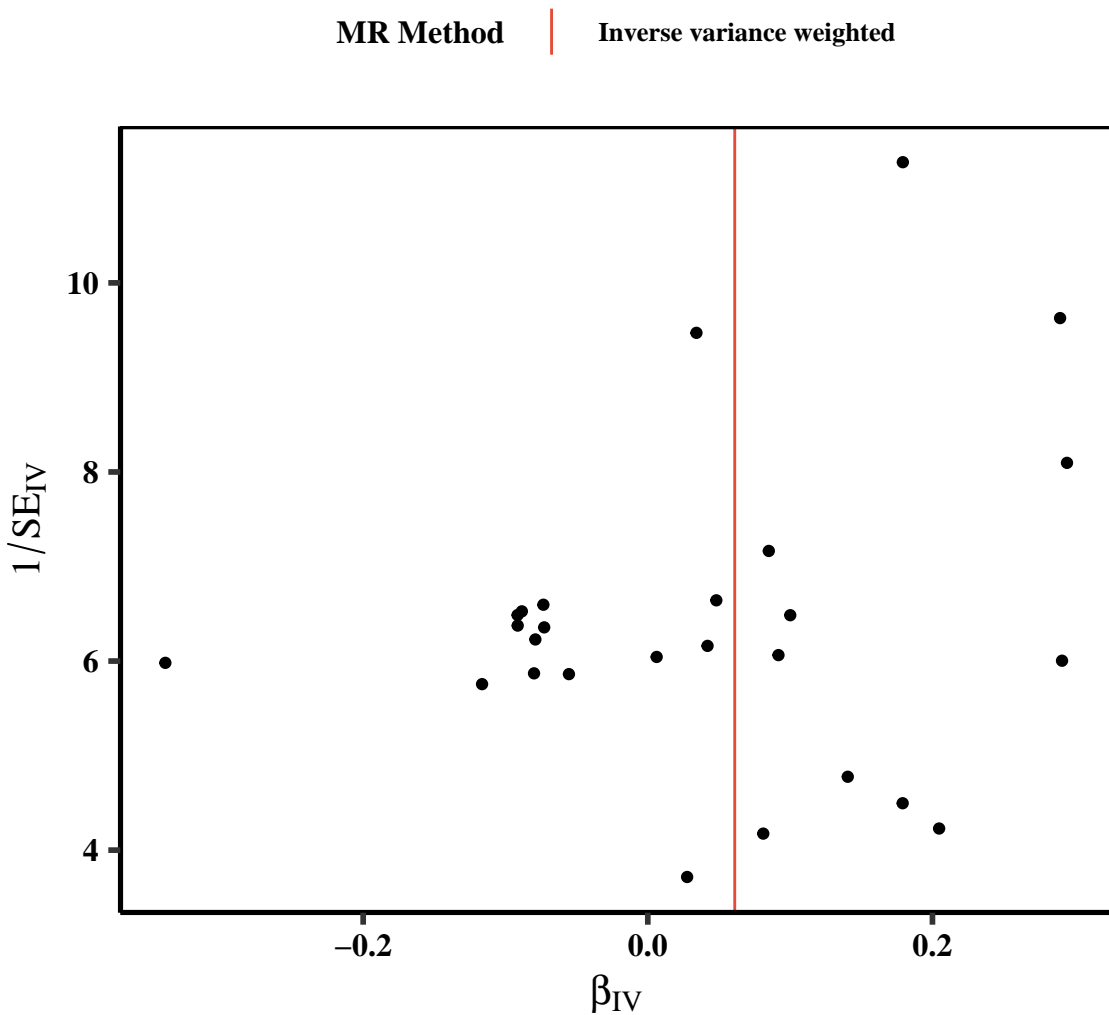

Supplement: Supplementary file 3 [file DataSheet3.zip › Supplementary Figure 3/STAT5B.pdf]

# MR of TLR2

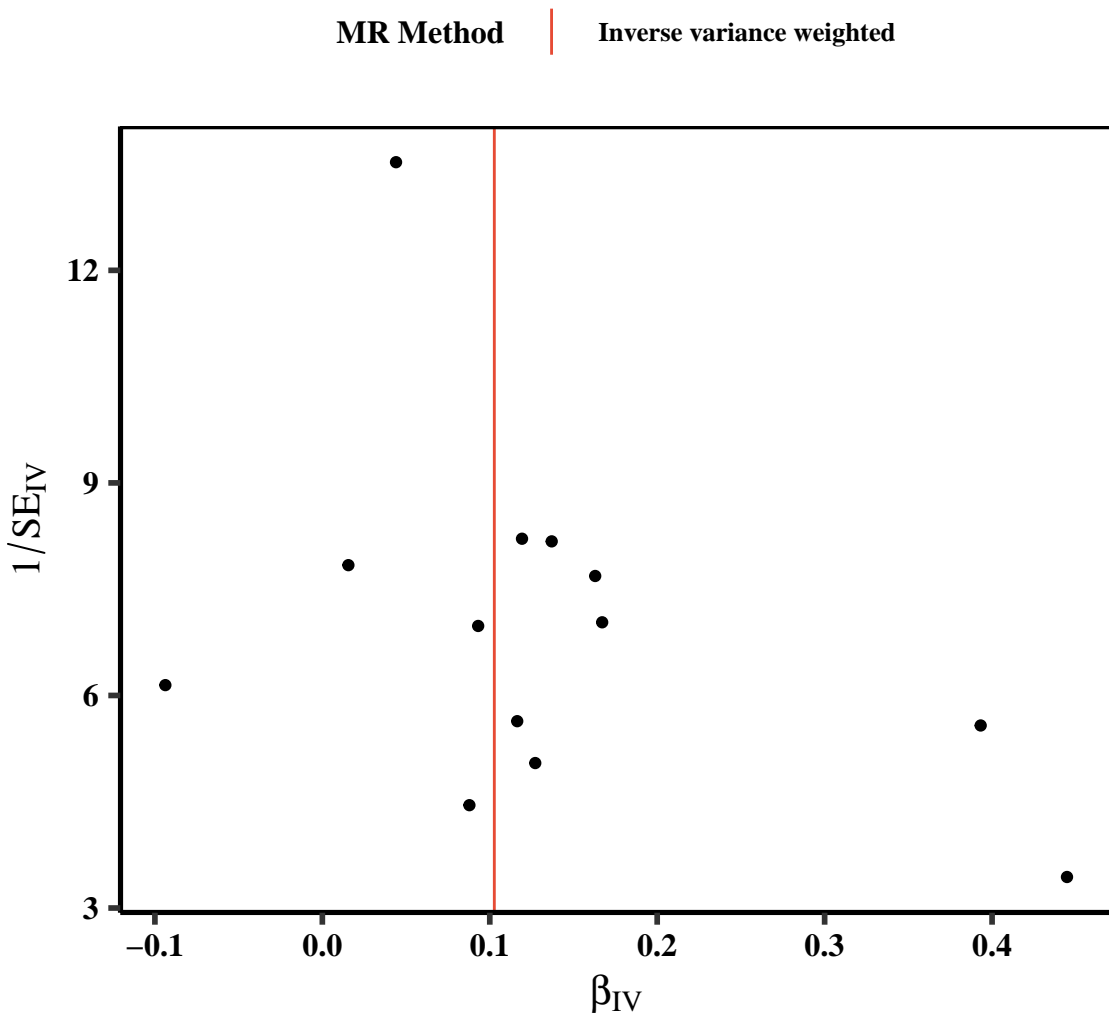

Supplement: Supplementary file 3 [file DataSheet3.zip › Supplementary Figure 3/TLR2.pdf]

# MR of TLR3

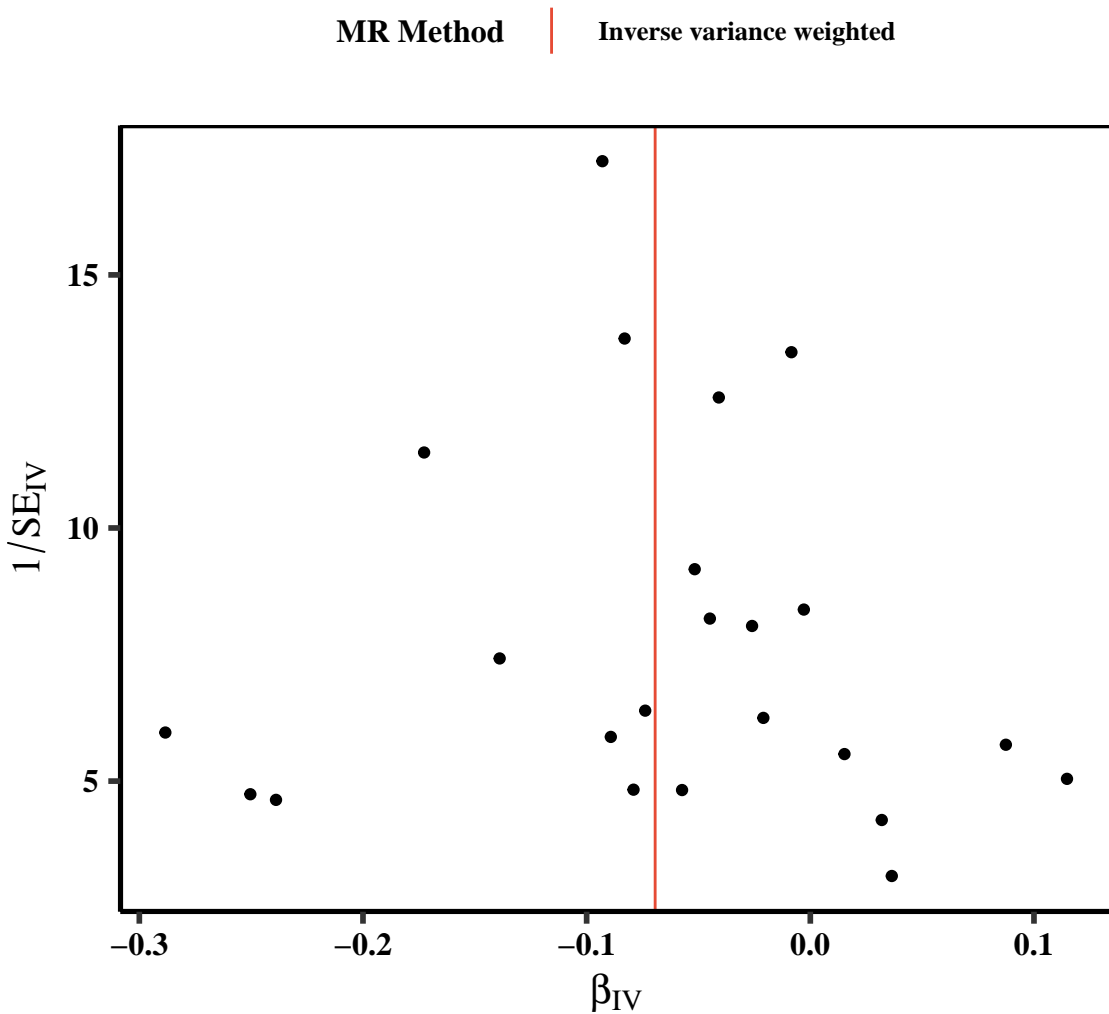

Supplement: Supplementary file 3 [file DataSheet3.zip › Supplementary Figure 3/TLR3.pdf]

# MR leave-one-out sensitivity analysis for ACTN1 on PCa

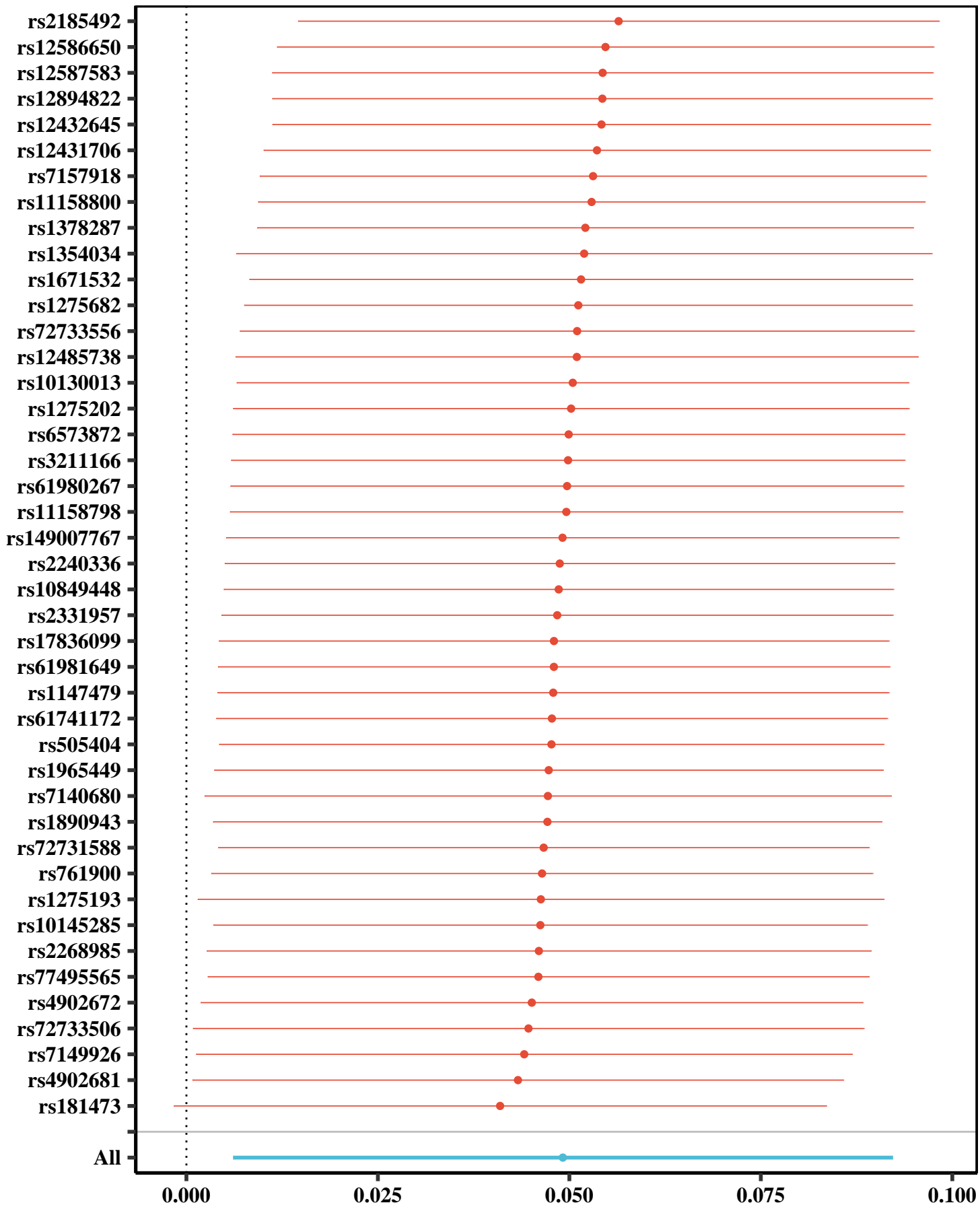

Supplement: Supplementary file 4 [file DataSheet4.zip › Supplementary Figure 4/ACTN1.pdf]

MR leave-one-out sensitivity analysis for BATF3 on PCa

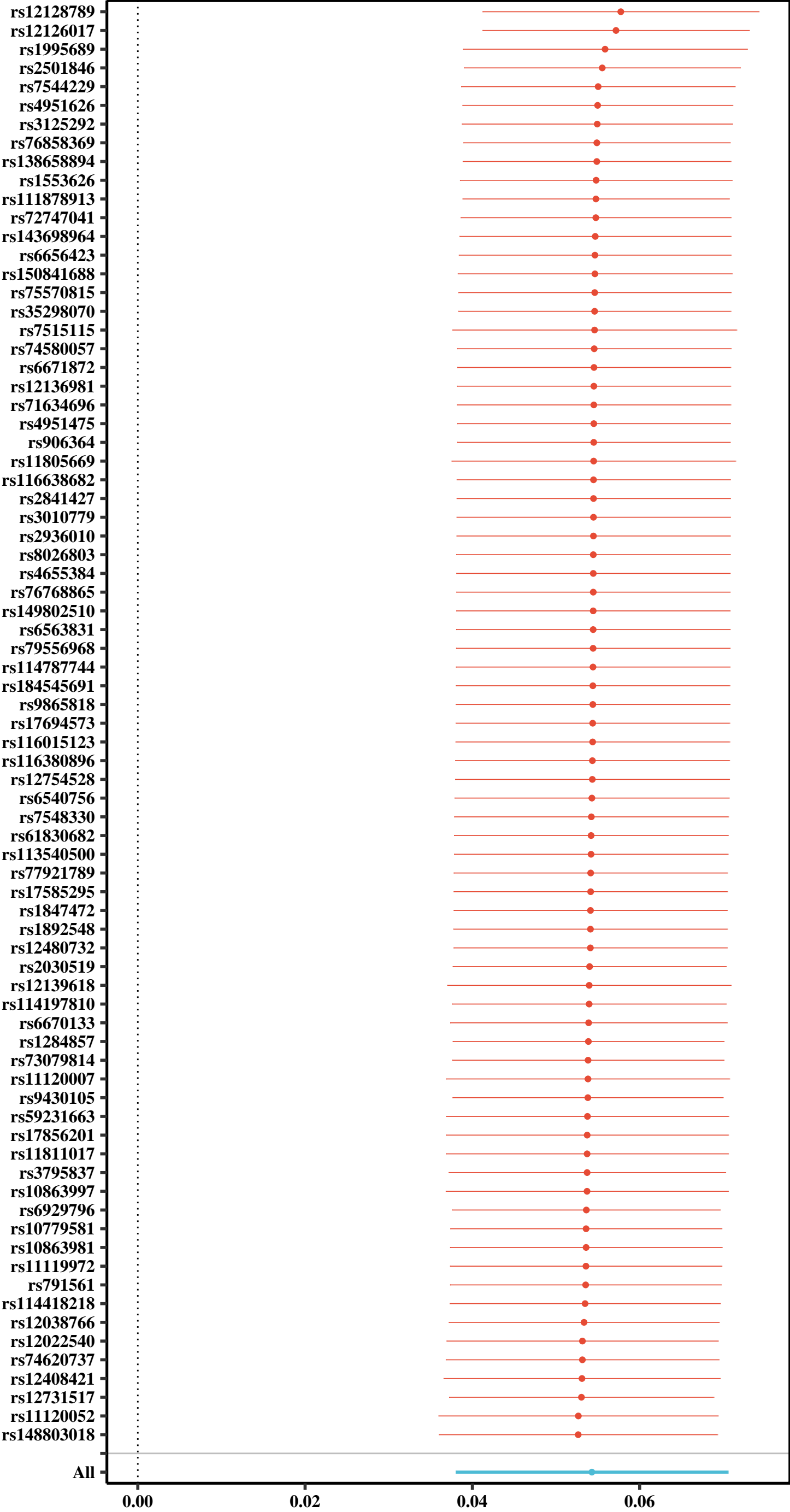

Supplement: Supplementary file 4 [file DataSheet4.zip › Supplementary Figure 4/BATF3.pdf]

# MR leave-one-out sensitivity analysis for BMP2 on PCa

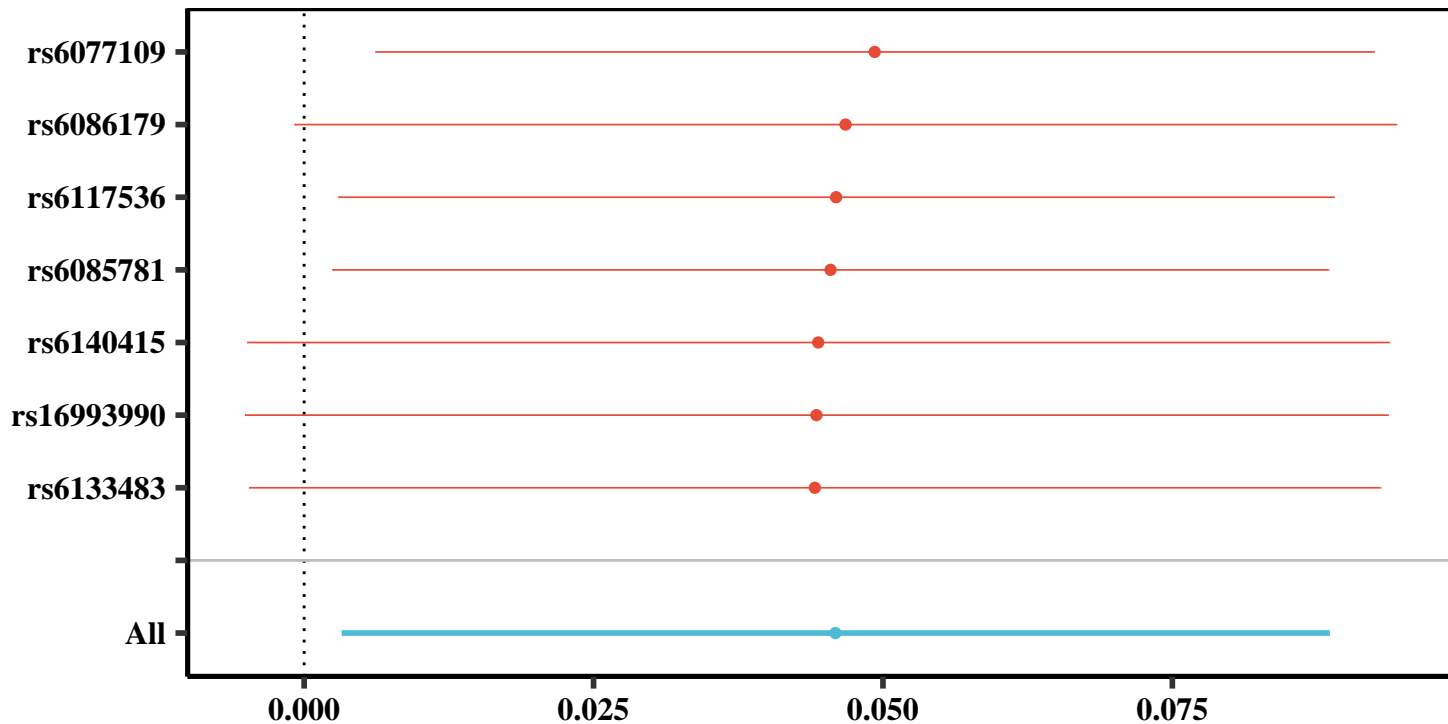

Supplement: Supplementary file 4 [file DataSheet4.zip › Supplementary Figure 4/BMP2.pdf]

# MR leave-one-out sensitivity analysis for CD109 on PCa

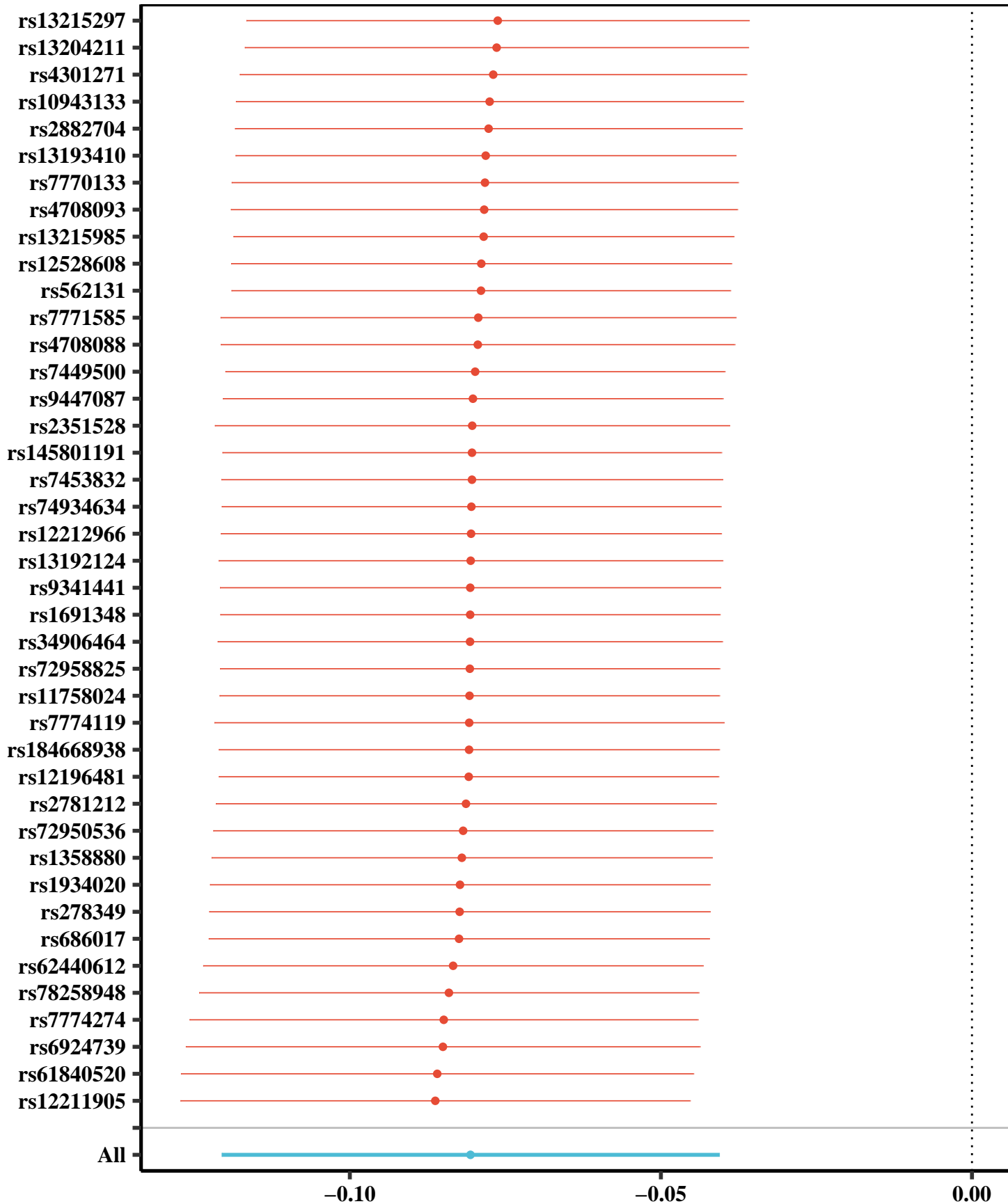

Supplement: Supplementary file 4 [file DataSheet4.zip › Supplementary Figure 4/CD109.pdf]

# MR leave-one-out sensitivity analysis for FASN on PCa

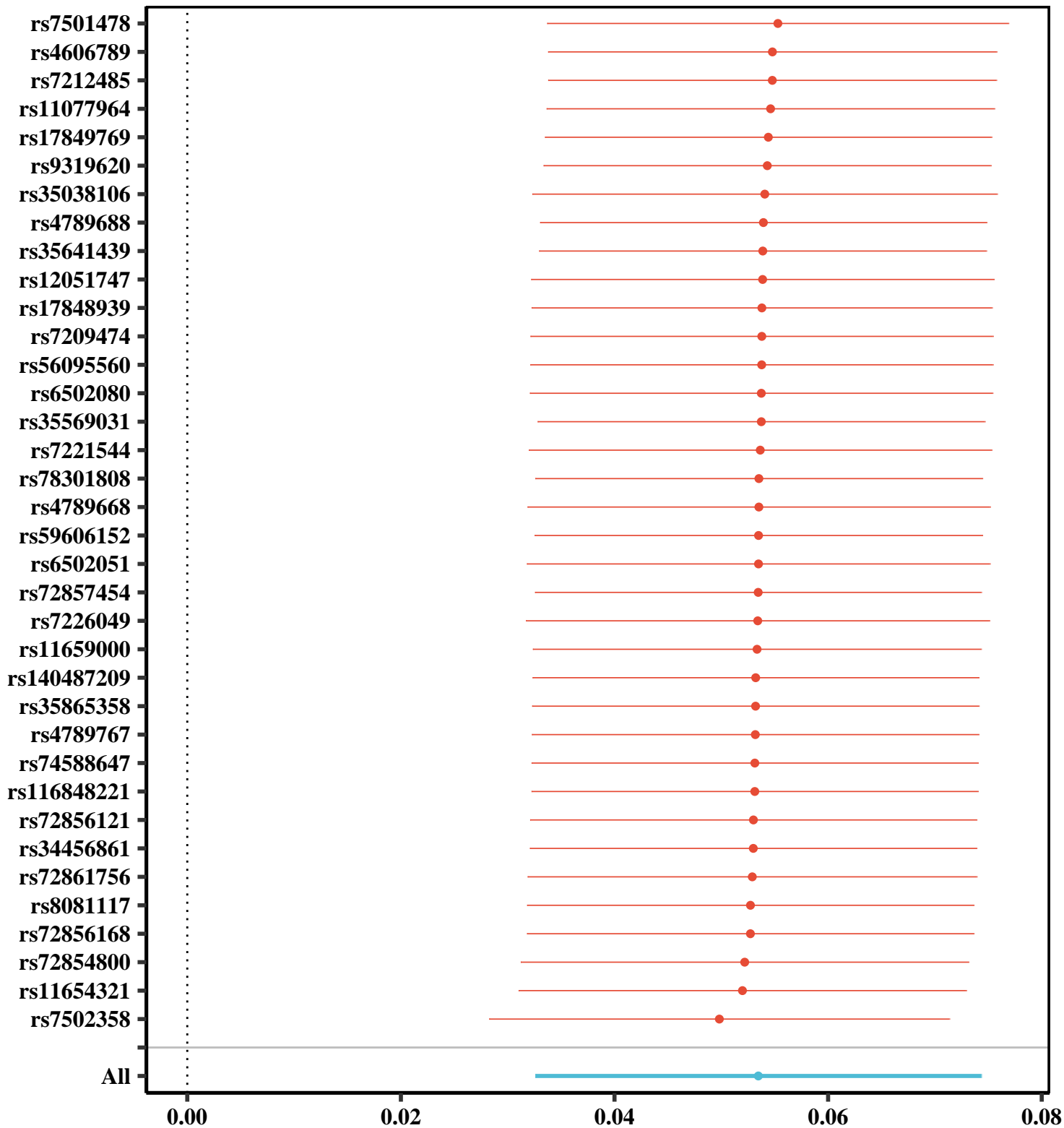

Supplement: Supplementary file 4 [file DataSheet4.zip › Supplementary Figure 4/FASN.pdf]

# MR leave-one-out sensitivity analysis for FBN1 on PCa

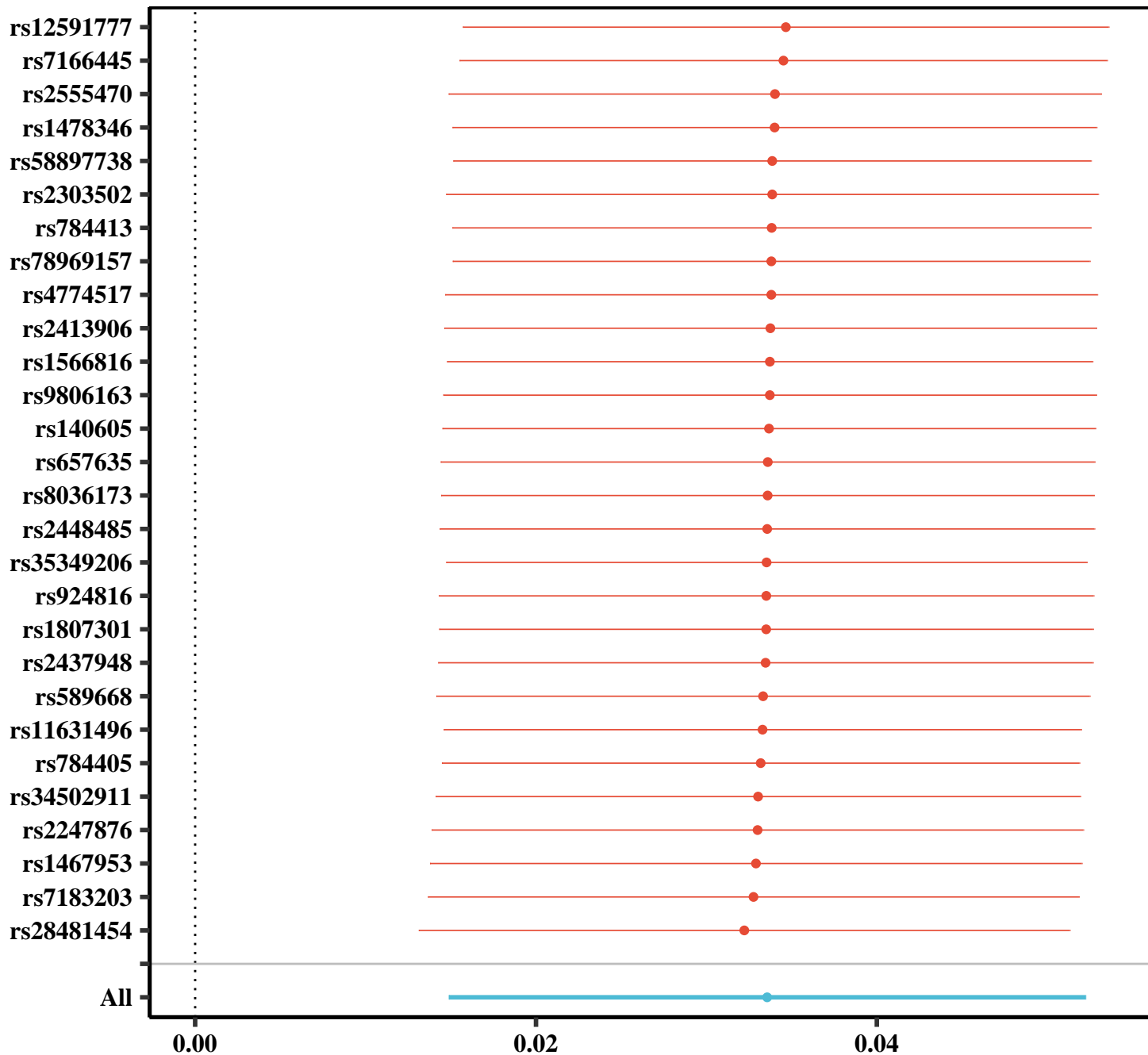

Supplement: Supplementary file 4 [file DataSheet4.zip › Supplementary Figure 4/FBN1.pdf]

# MR leave-one-out sensitivity analysis for FLNA on PCa

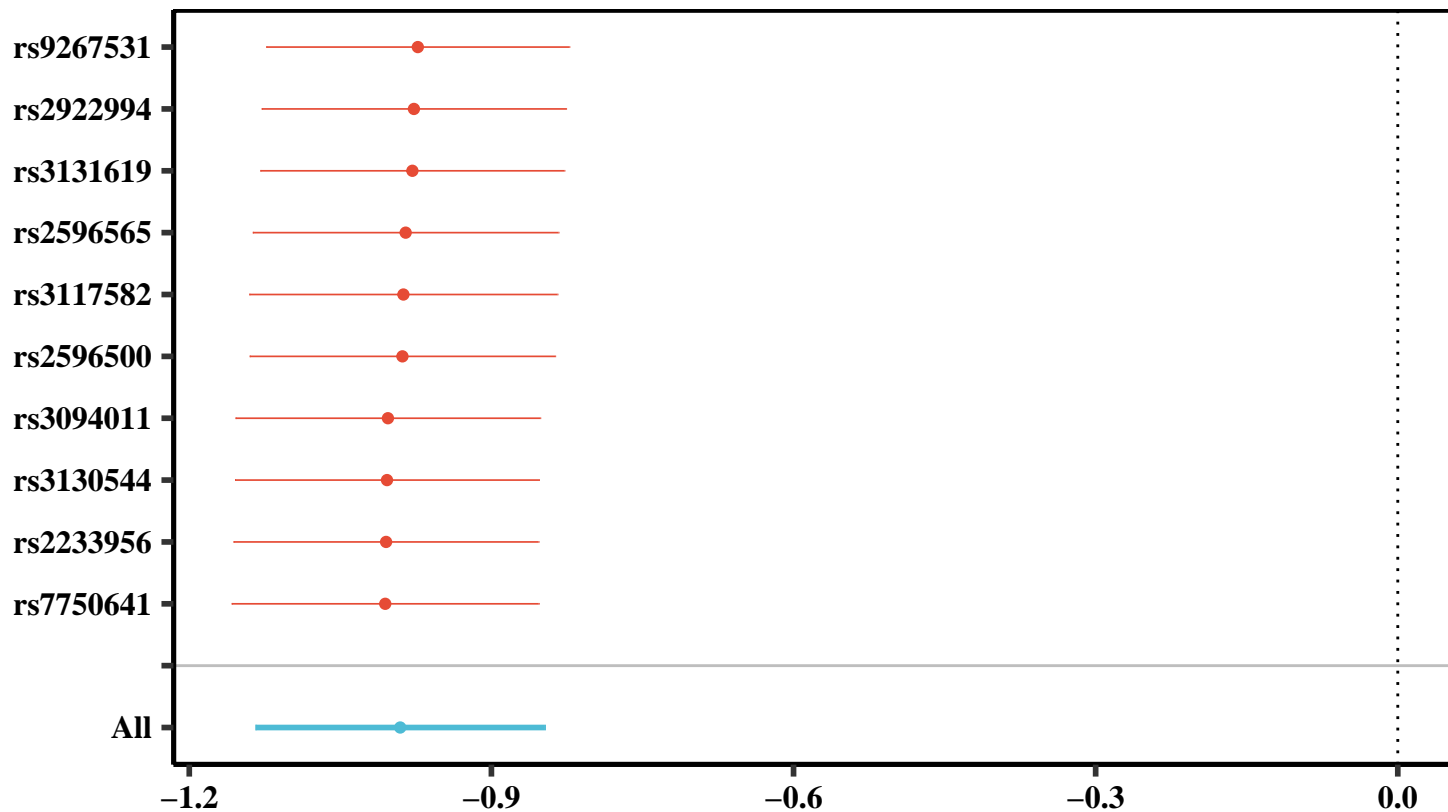

Supplement: Supplementary file 4 [file DataSheet4.zip › Supplementary Figure 4/FLNA.pdf]

# MR leave-one-out sensitivity analysis for GATA3 on PCa

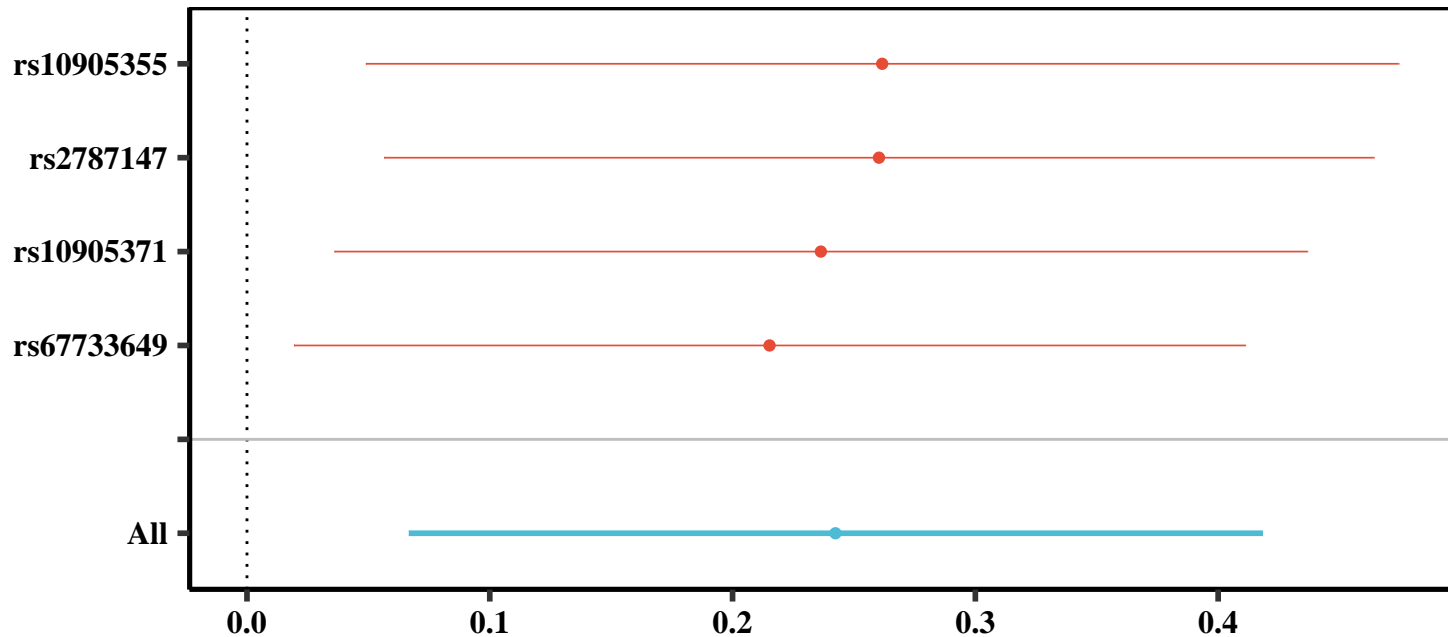

Supplement: Supplementary file 4 [file DataSheet4.zip › Supplementary Figure 4/GATA3.pdf]

# MR leave-one-out sensitivity analysis for GP5 on PCa

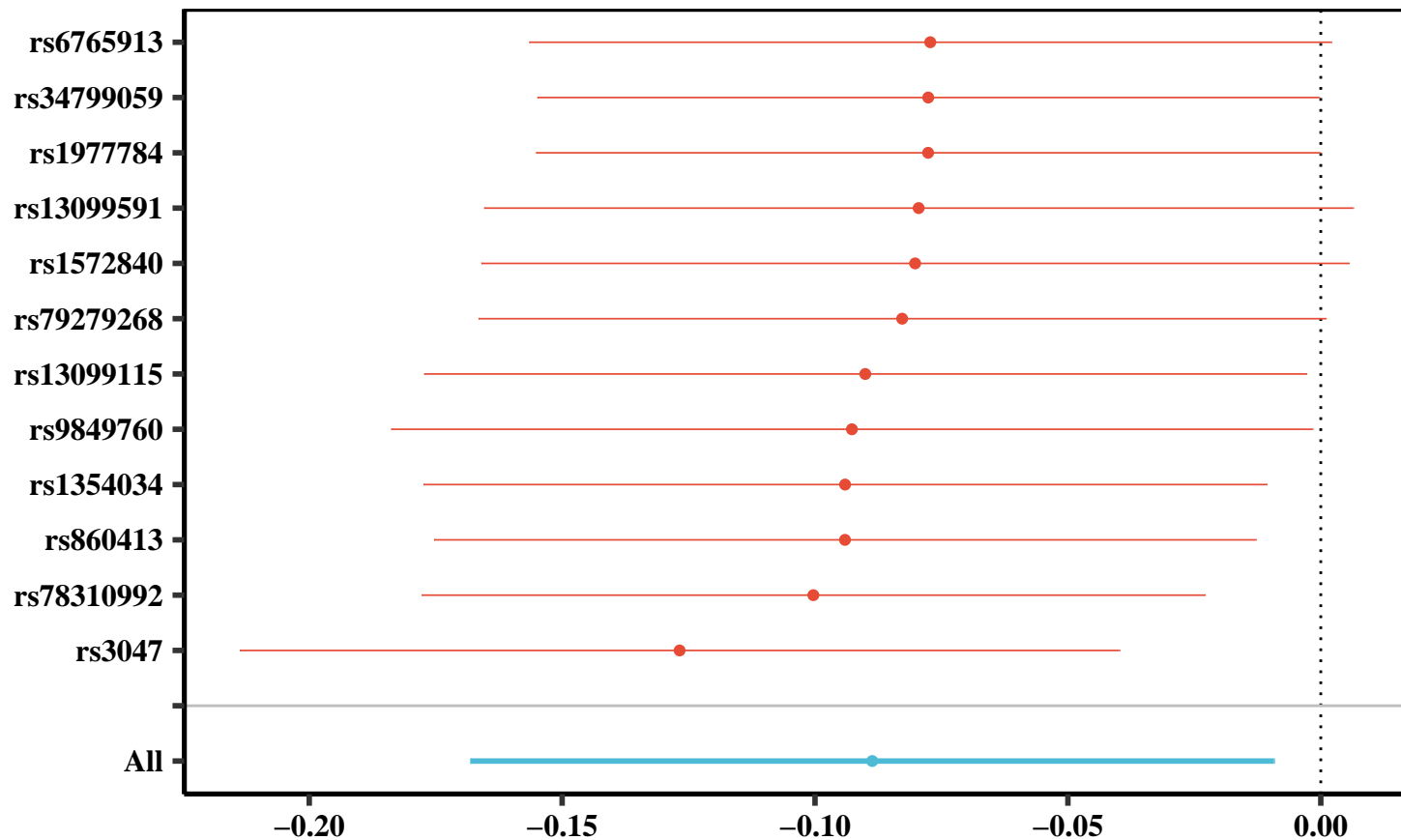

Supplement: Supplementary file 4 [file DataSheet4.zip › Supplementary Figure 4/GP5.pdf]

# MR leave-one-out sensitivity analysis for IFI16 on PCa

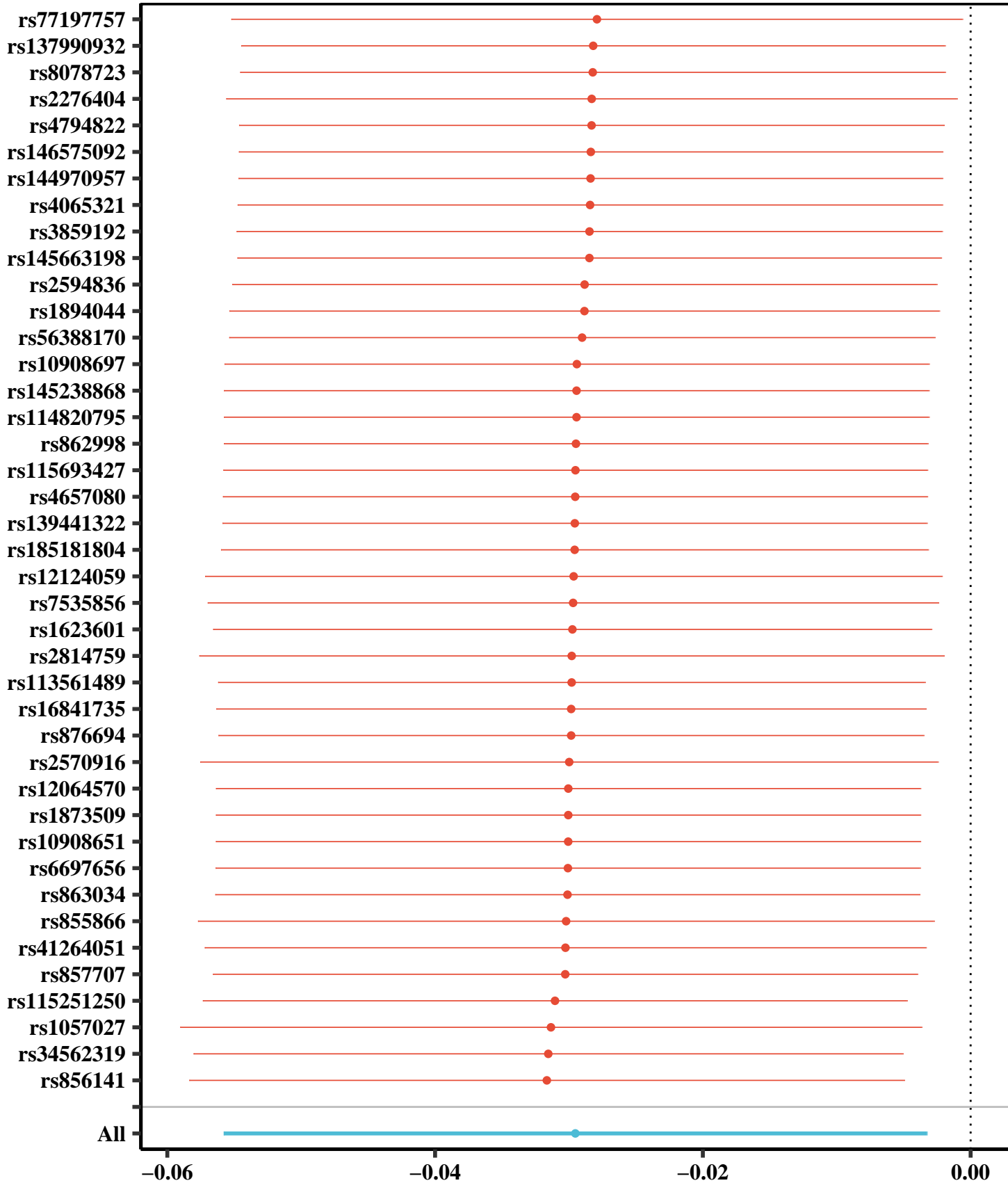

Supplement: Supplementary file 4 [file DataSheet4.zip › Supplementary Figure 4/IFI16.pdf]

MR leave-one-out sensitivity analysis for IL1RL1 on PCa

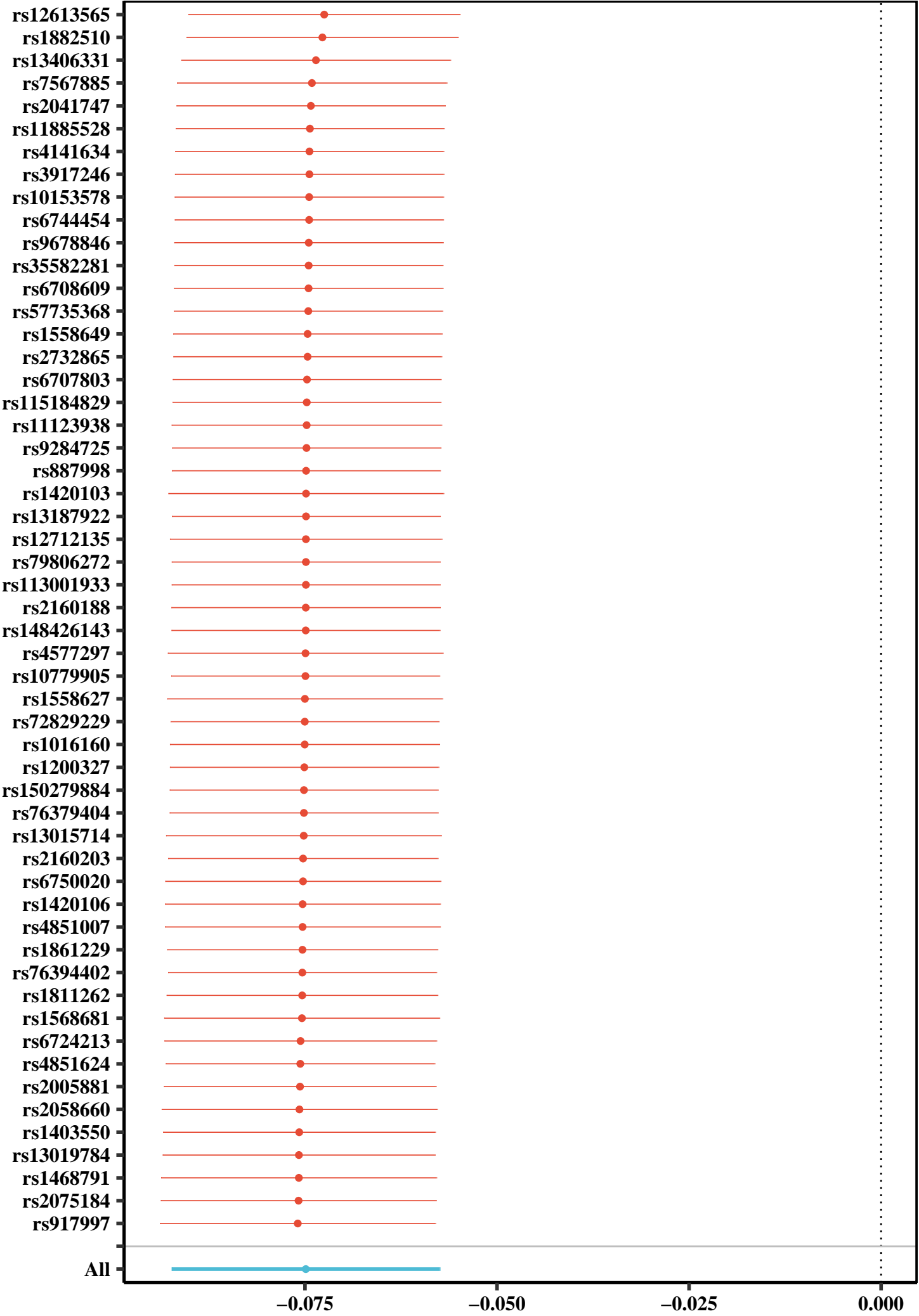

Supplement: Supplementary file 4 [file DataSheet4.zip › Supplementary Figure 4/IL1RL1.pdf]

# MR leave-one-out sensitivity analysis for ISG15 on PCa

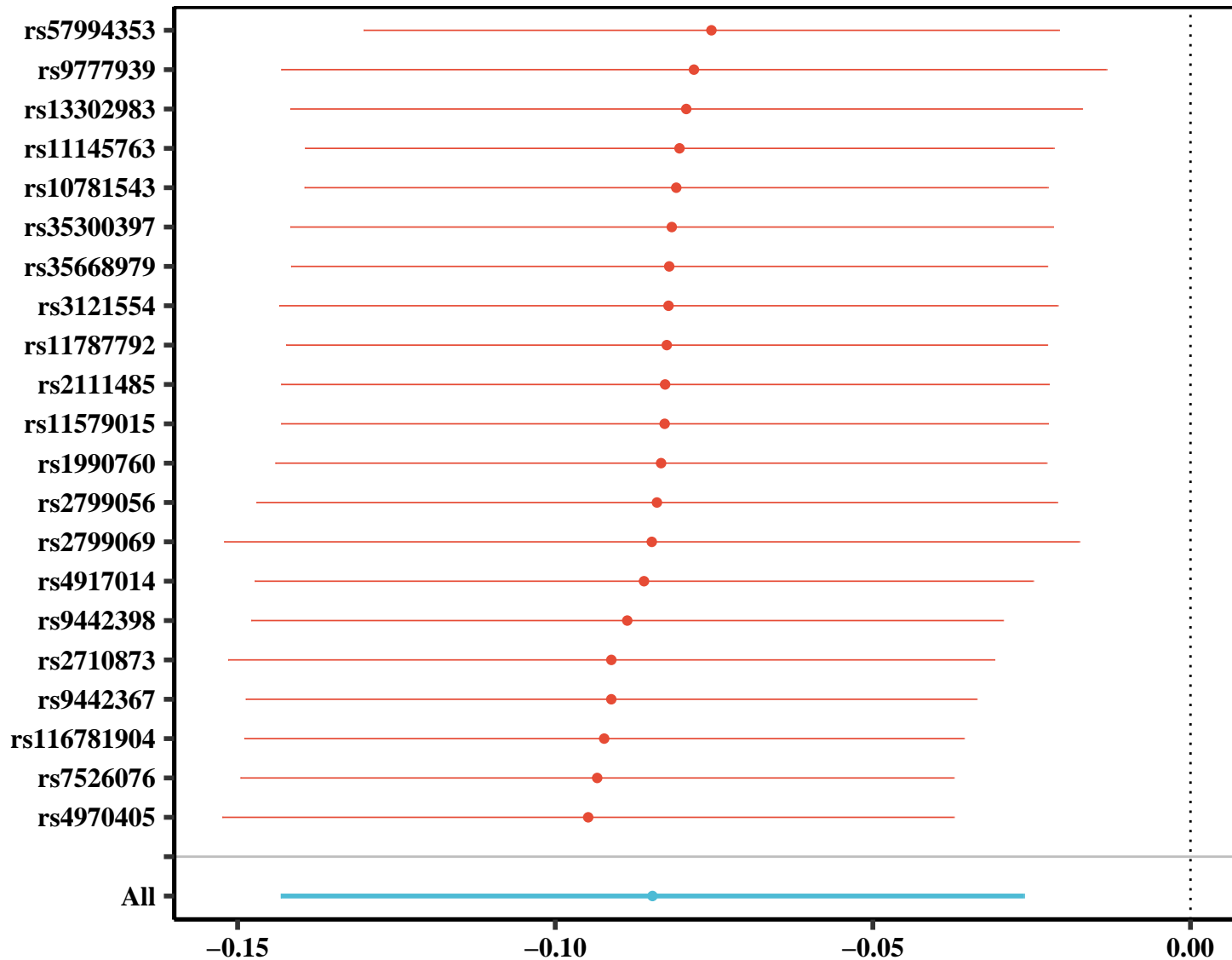

Supplement: Supplementary file 4 [file DataSheet4.zip › Supplementary Figure 4/ISG15.pdf]

# MR leave-one-out sensitivity analysis for KIT on PCa

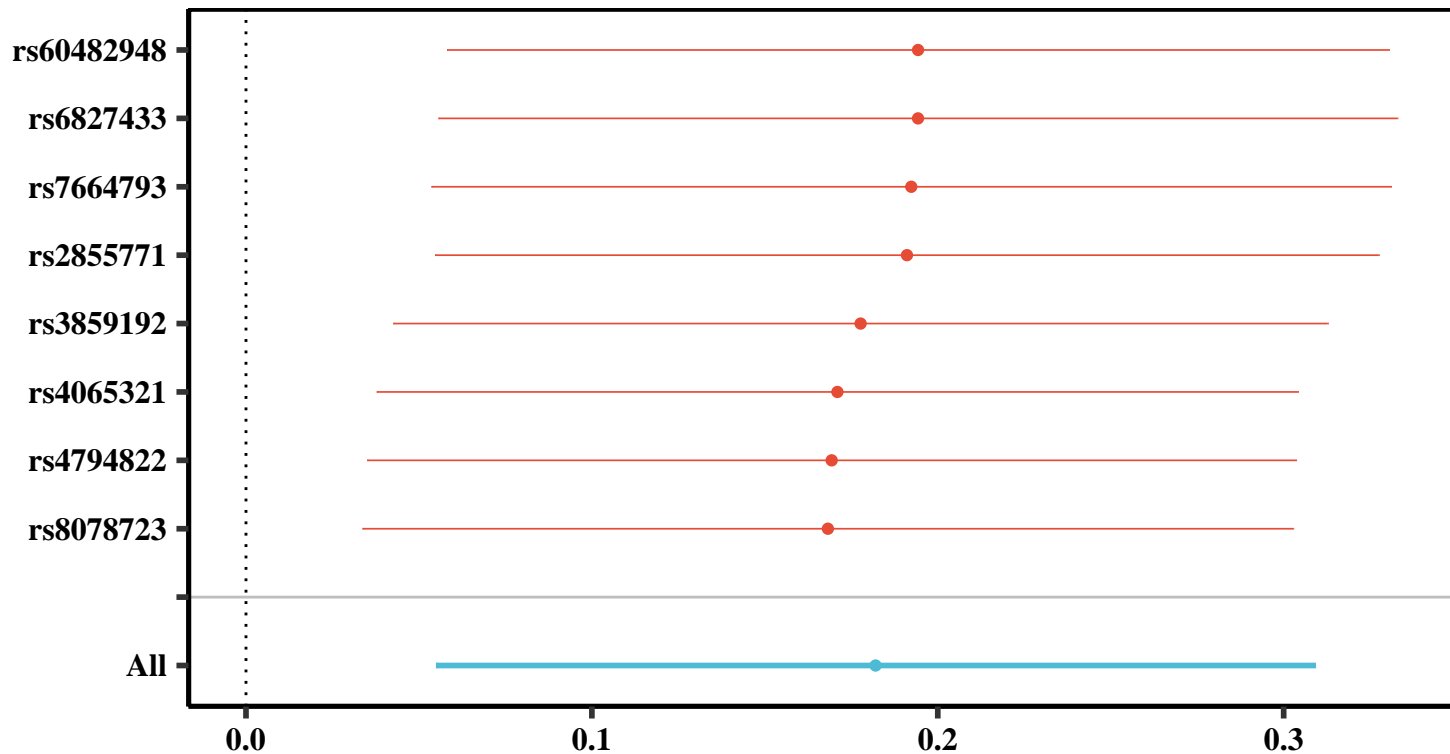

Supplement: Supplementary file 4 [file DataSheet4.zip › Supplementary Figure 4/KIT.pdf]

# MR leave-one-out sensitivity analysis for KLF10 on PCa

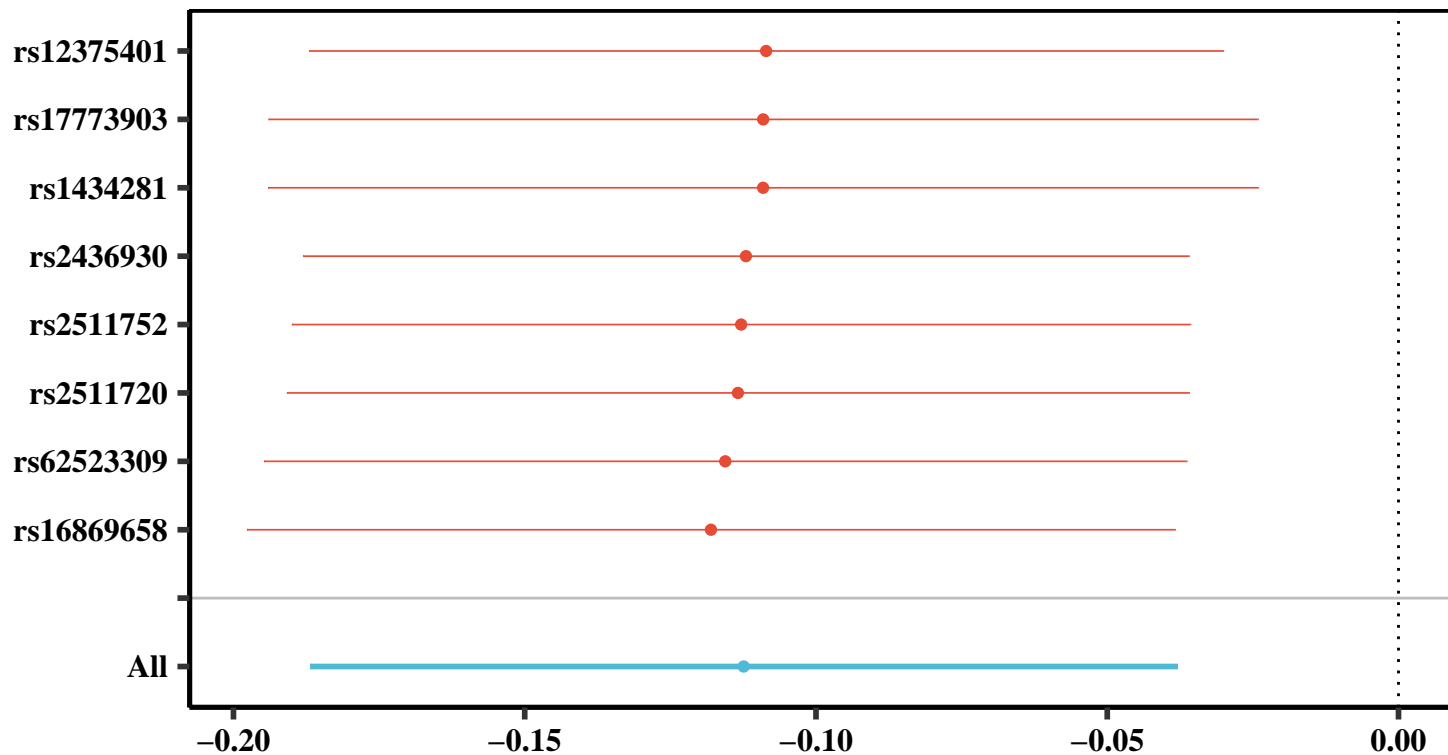

Supplement: Supplementary file 4 [file DataSheet4.zip › Supplementary Figure 4/KLF10.pdf]

# MR leave-one-out sensitivity analysis for LOX on PCa

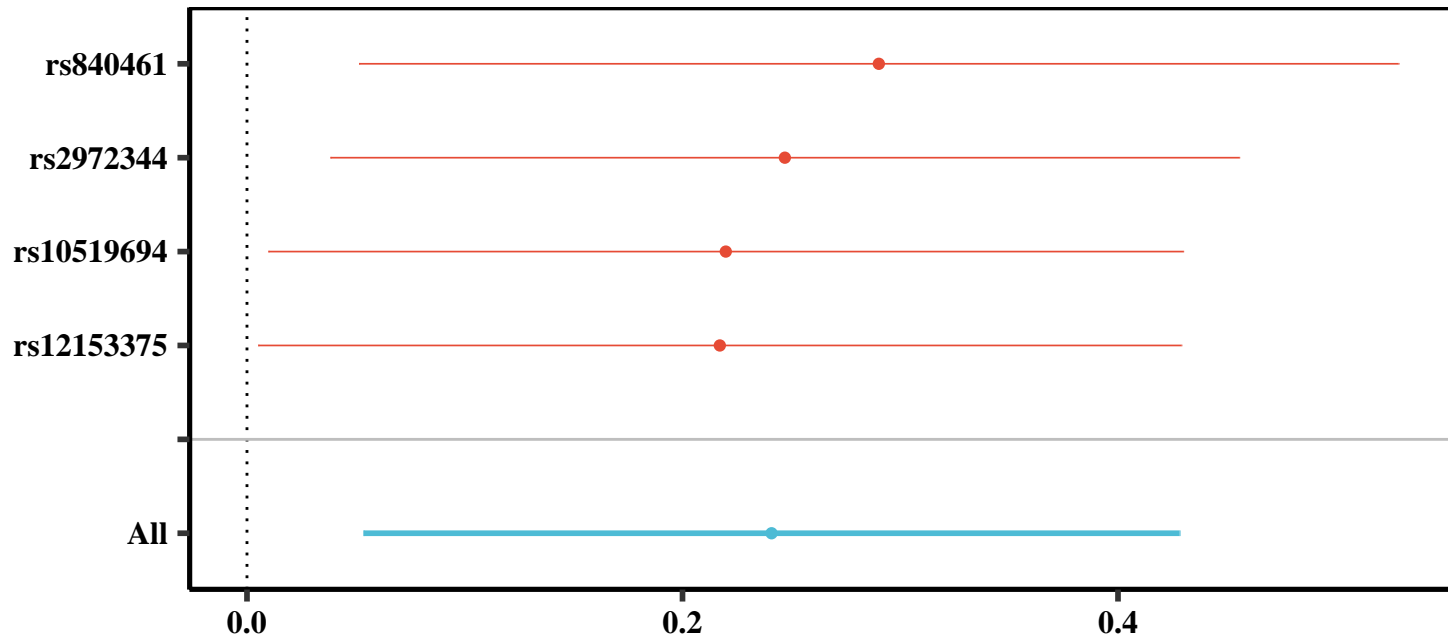

Supplement: Supplementary file 4 [file DataSheet4.zip › Supplementary Figure 4/LOX.pdf]

# MR leave-one-out sensitivity analysis for MITF on PCa

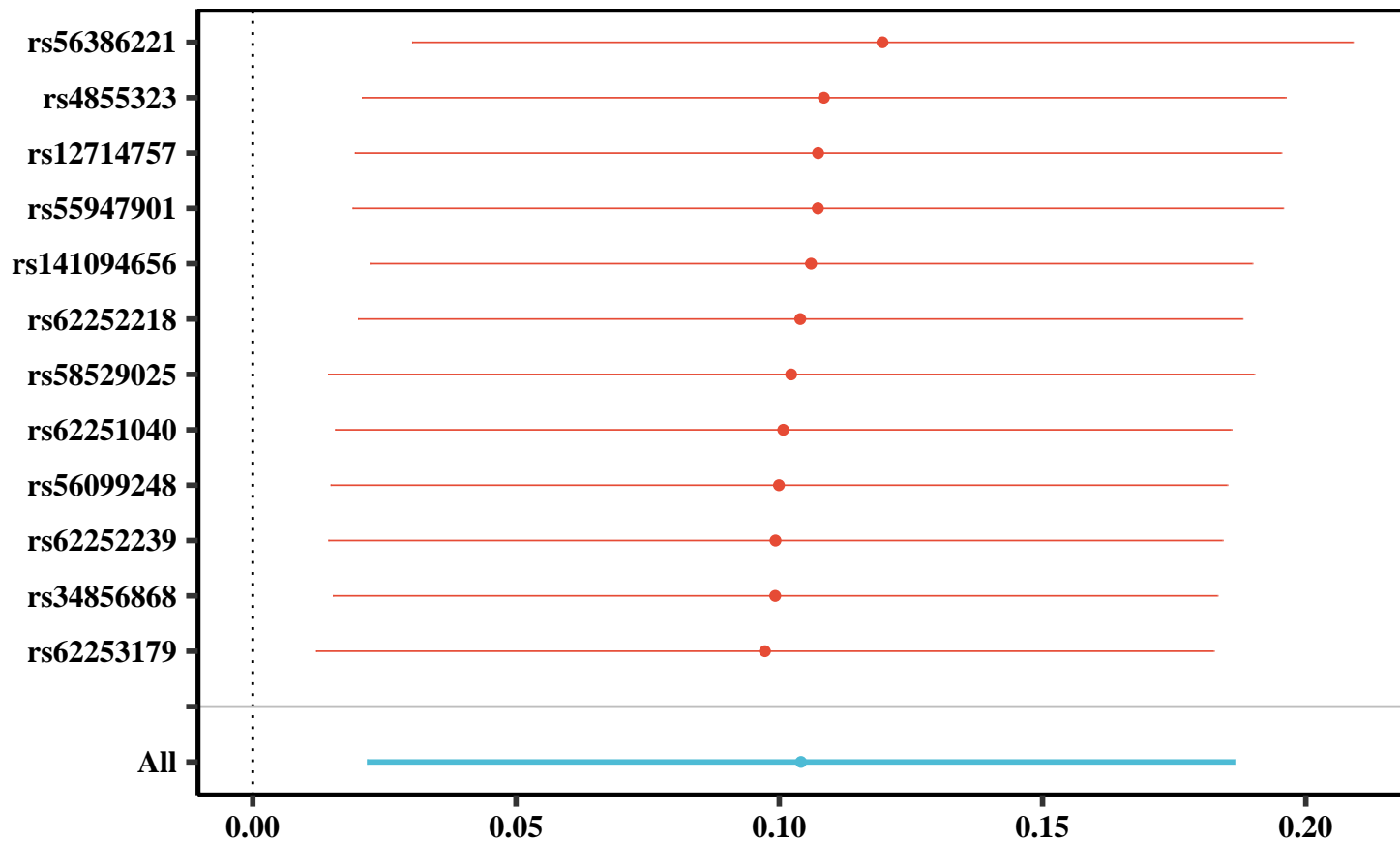

Supplement: Supplementary file 4 [file DataSheet4.zip › Supplementary Figure 4/MITF.pdf]

MR leave-one-out sensitivity analysis for MTURN on PCa

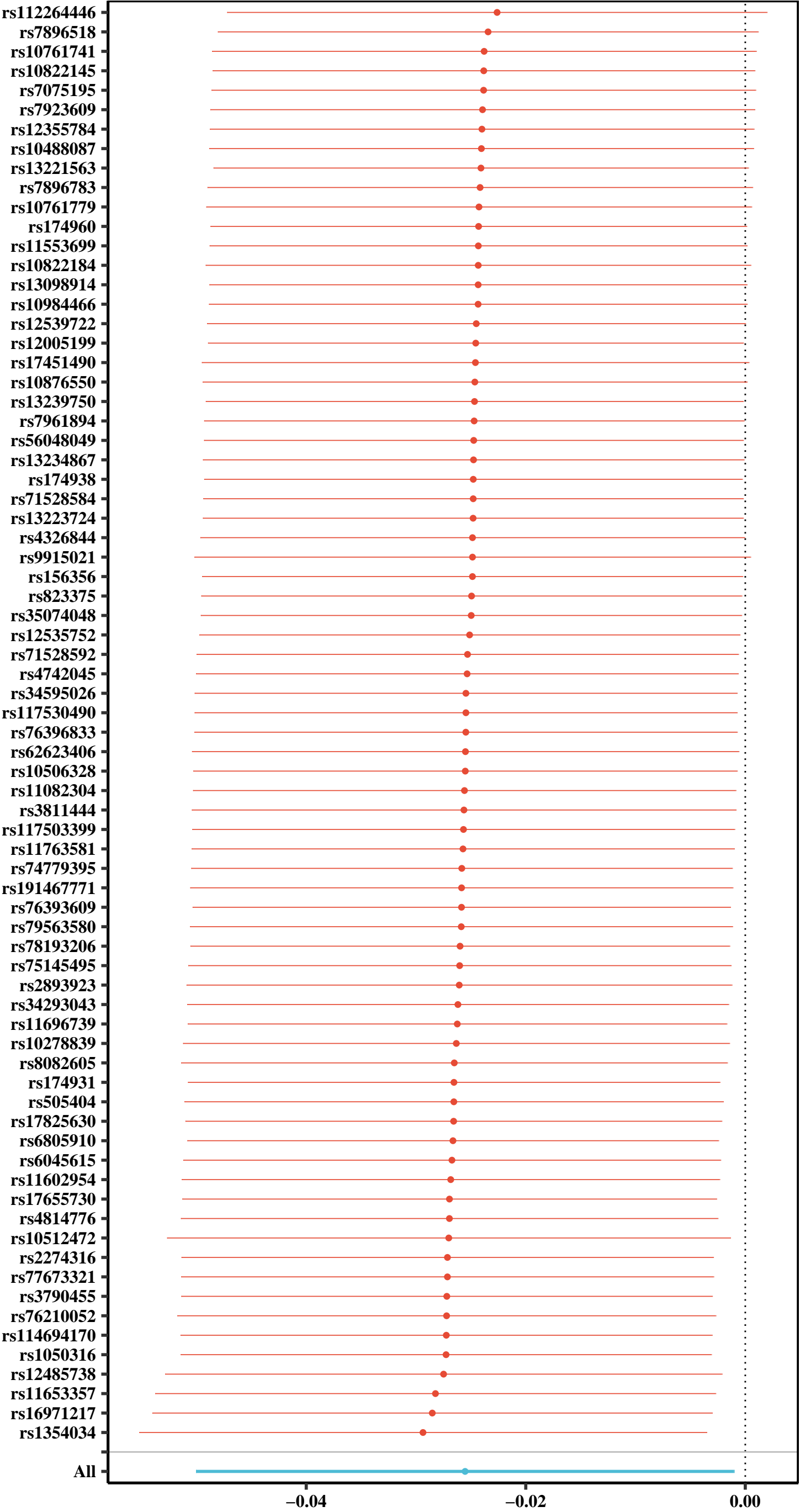

Supplement: Supplementary file 4 [file DataSheet4.zip › Supplementary Figure 4/MTURN.pdf]

# MR leave-one-out sensitivity analysis for NR3C1 on PCa

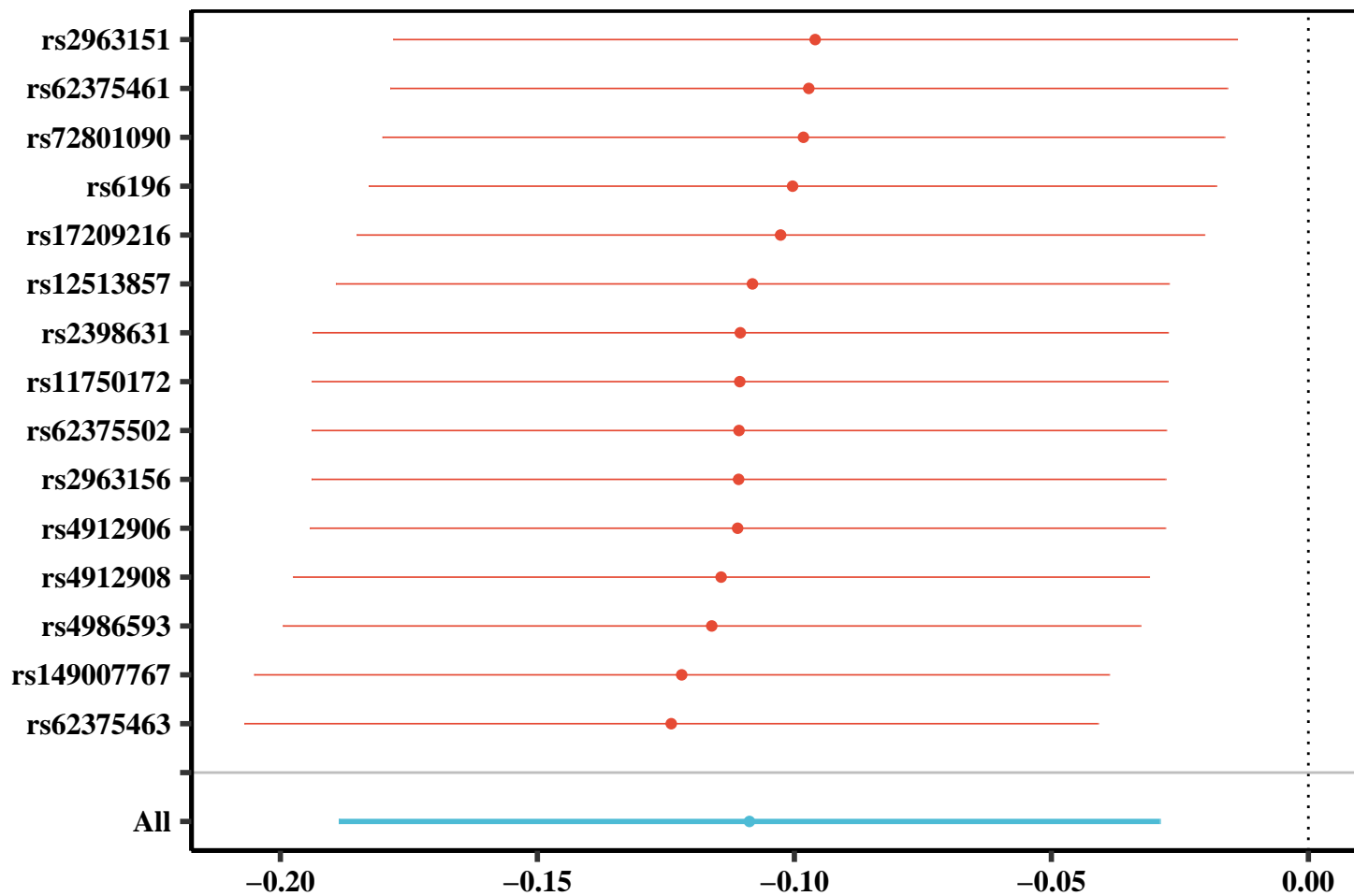

Supplement: Supplementary file 4 [file DataSheet4.zip › Supplementary Figure 4/NR3C1.pdf]

MR leave-one-out sensitivity analysis for PPP3CA on PCa

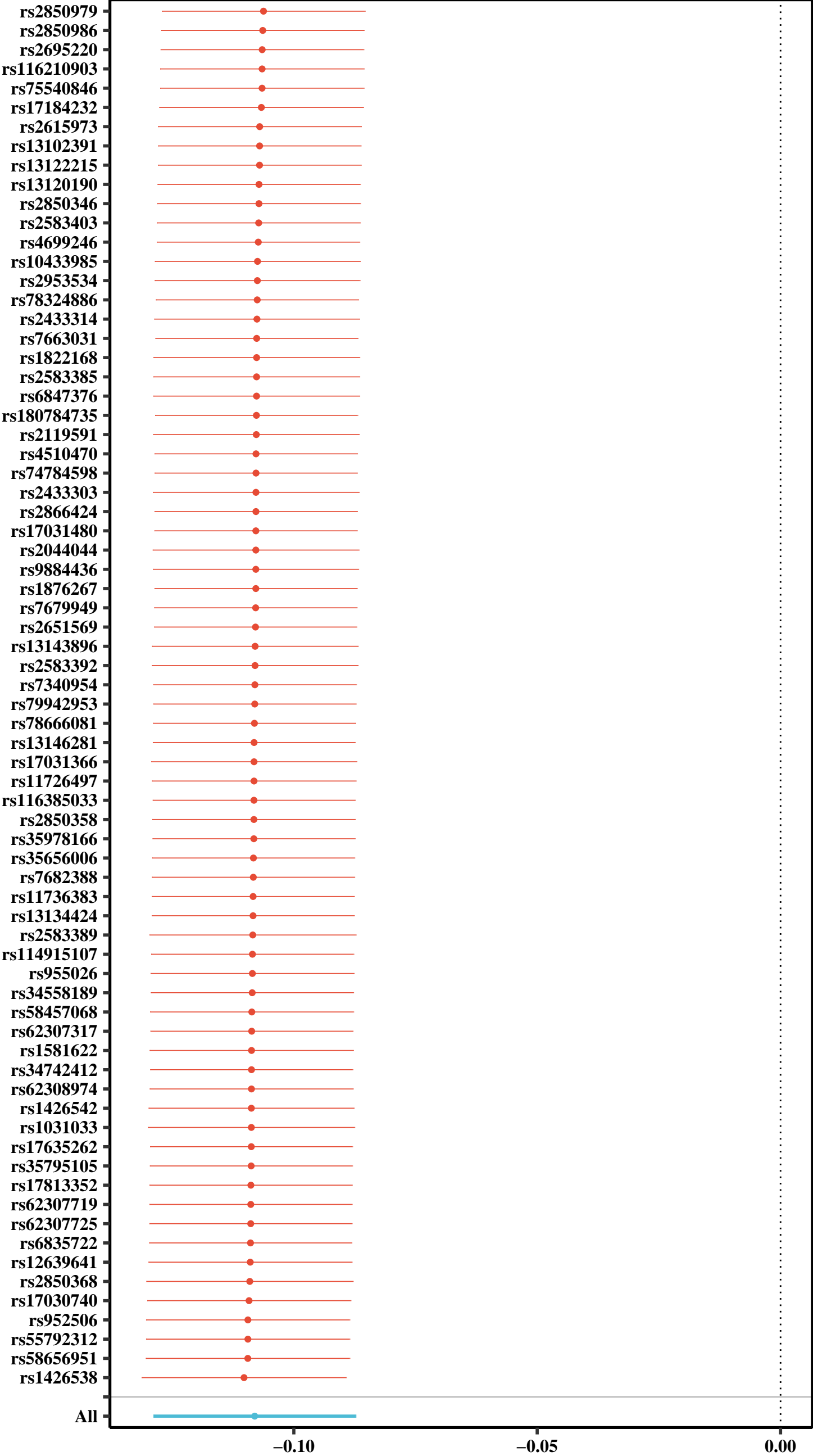

Supplement: Supplementary file 4 [file DataSheet4.zip › Supplementary Figure 4/PPP3CA.pdf]

# MR leave-one-out sensitivity analysis for RACGAP1 on PCa

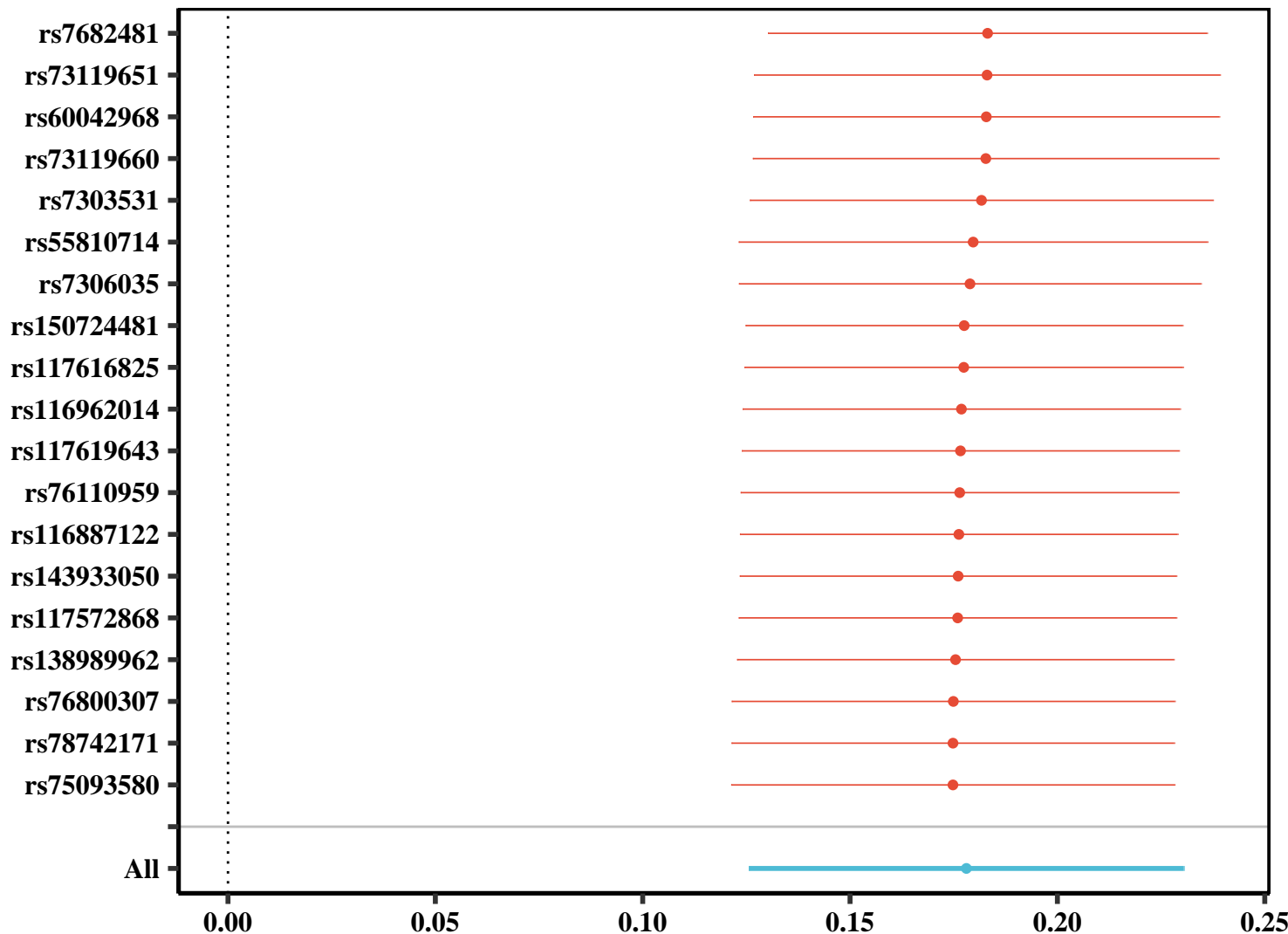

Supplement: Supplementary file 4 [file DataSheet4.zip › Supplementary Figure 4/RACGAP1.pdf]

# MR leave-one-out sensitivity analysis for STAT5B on PCa

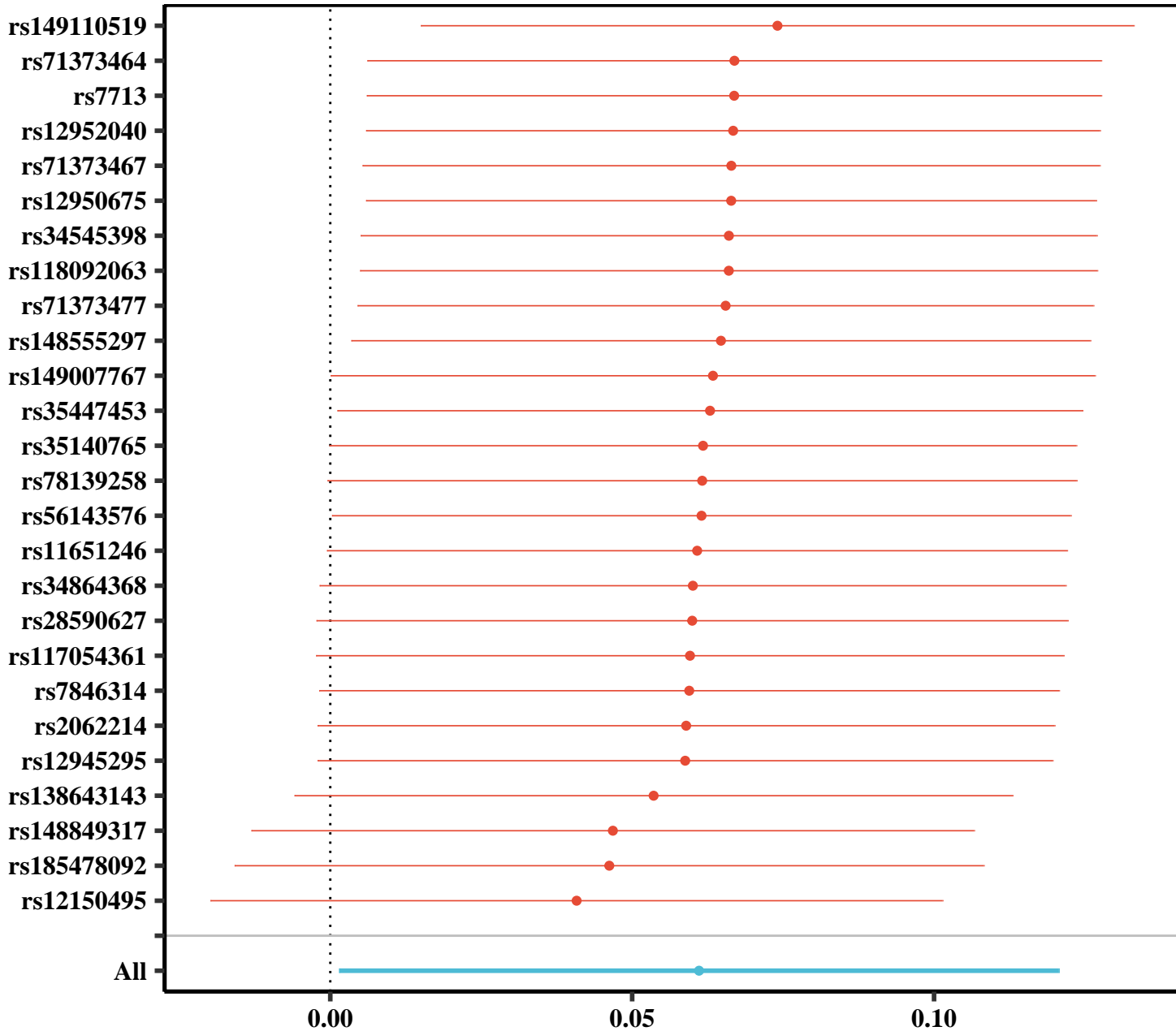

Supplement: Supplementary file 4 [file DataSheet4.zip › Supplementary Figure 4/STAT5B.pdf]

# MR leave-one-out sensitivity analysis for TLR2 on PCa

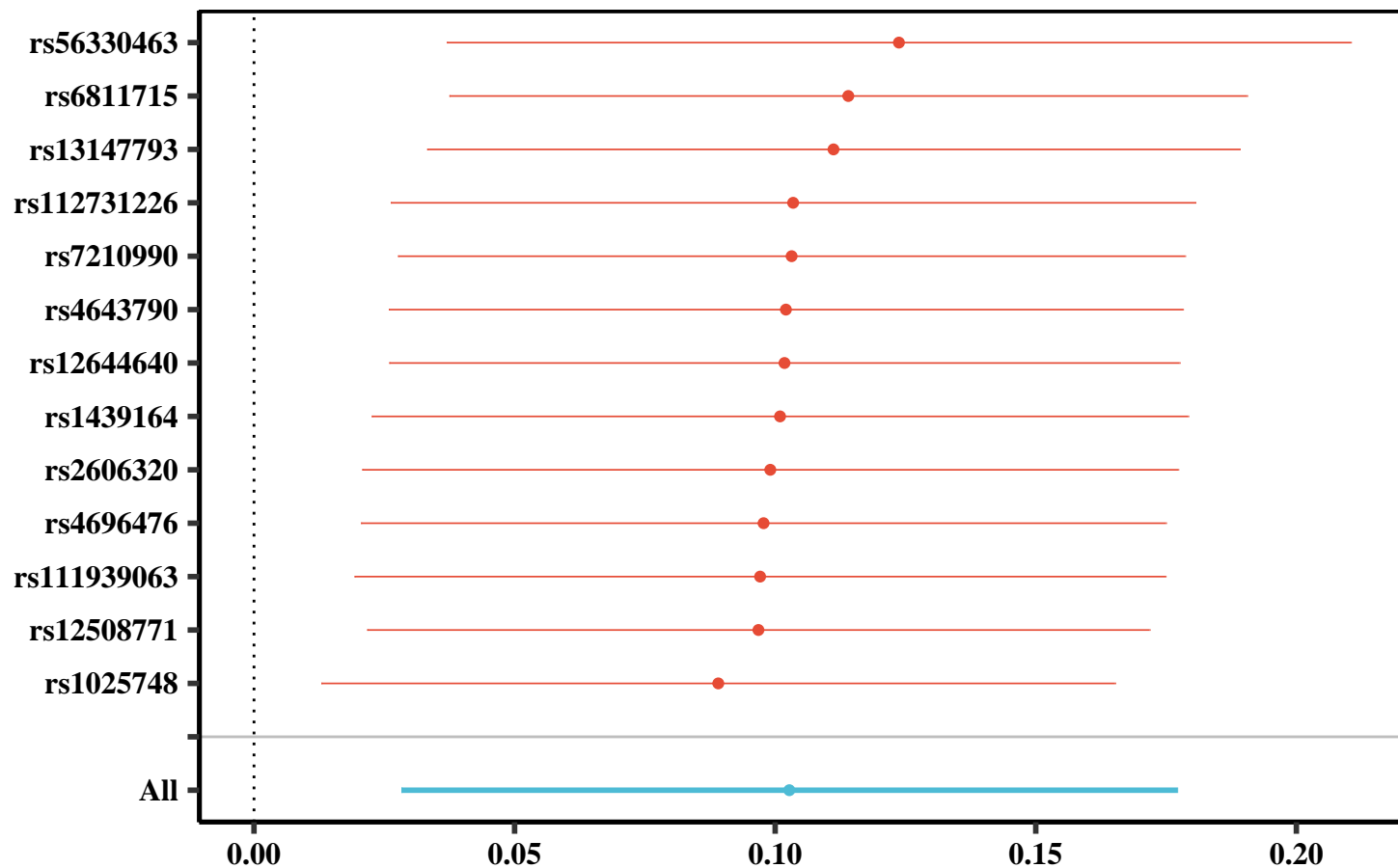

Supplement: Supplementary file 4 [file DataSheet4.zip › Supplementary Figure 4/TLR2.pdf]

# MR leave-one-out sensitivity analysis for TLR3 on PCa

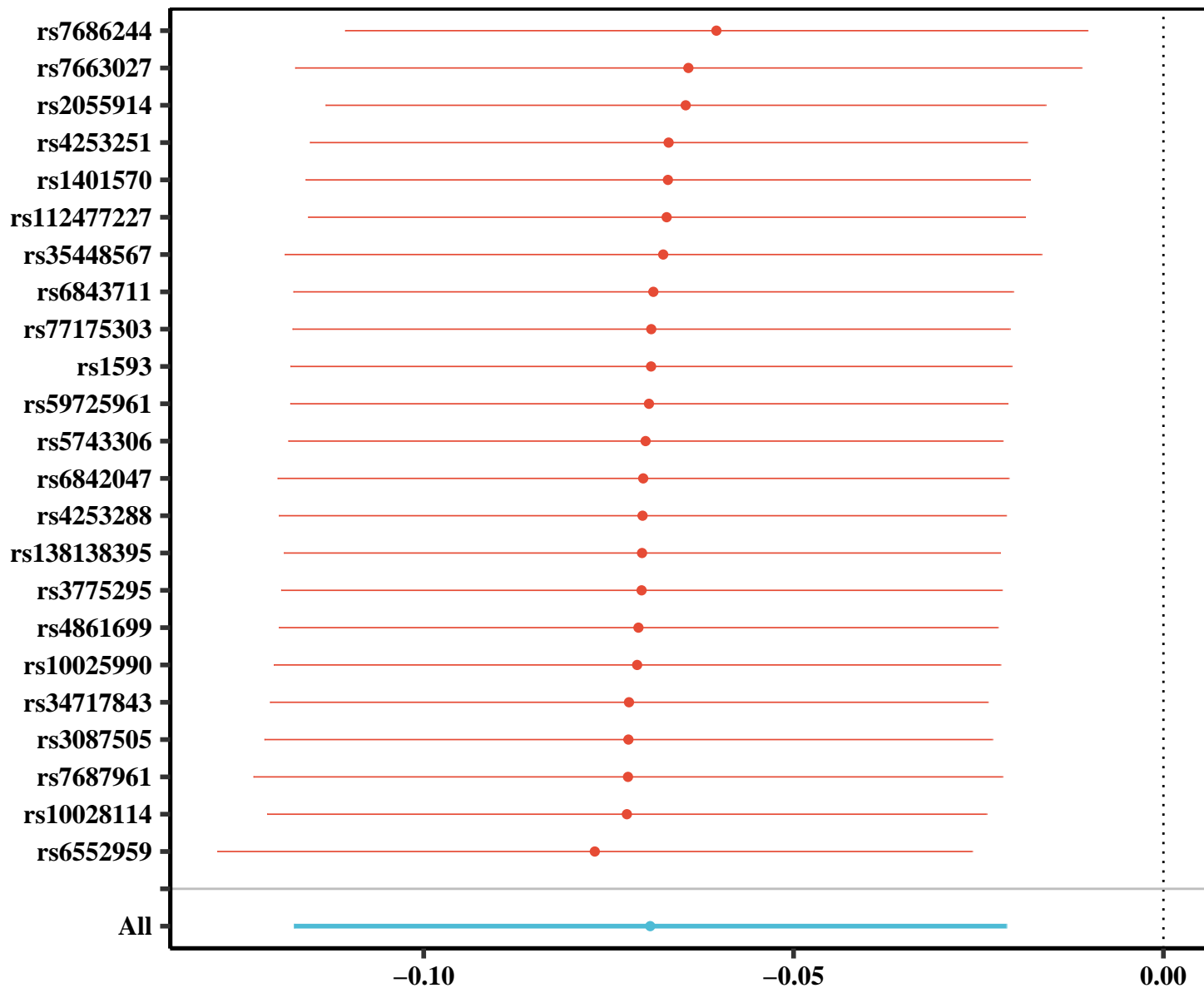

Supplement: Supplementary file 4 [file DataSheet4.zip › Supplementary Figure 4/TLR3.pdf]

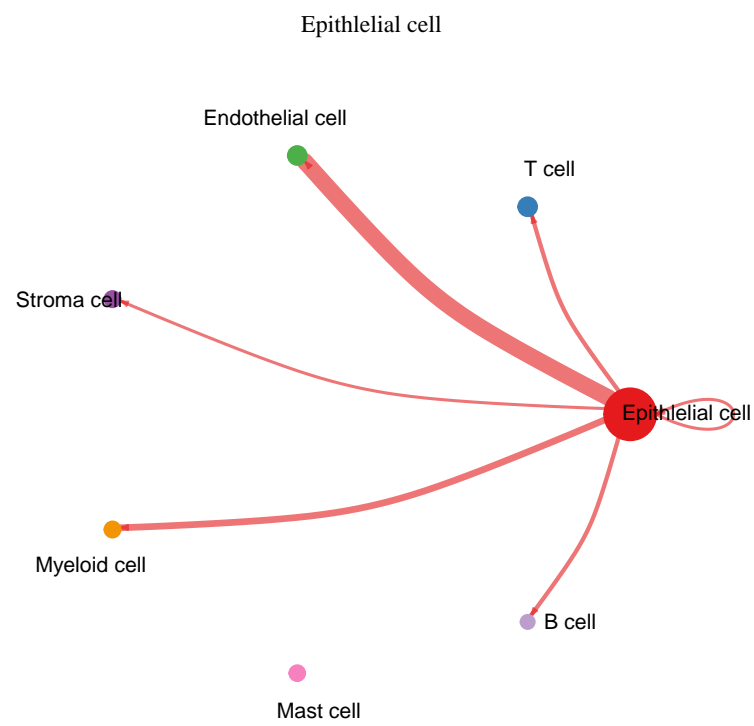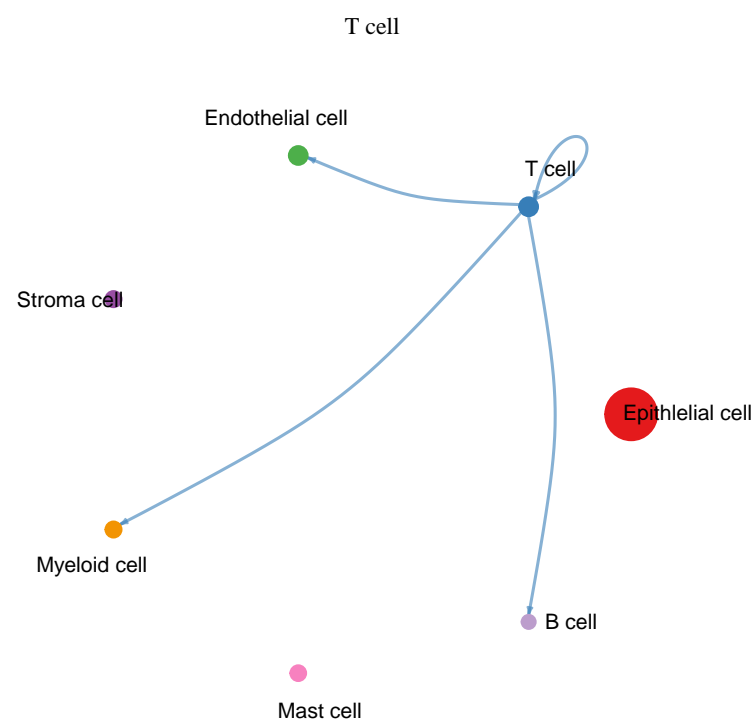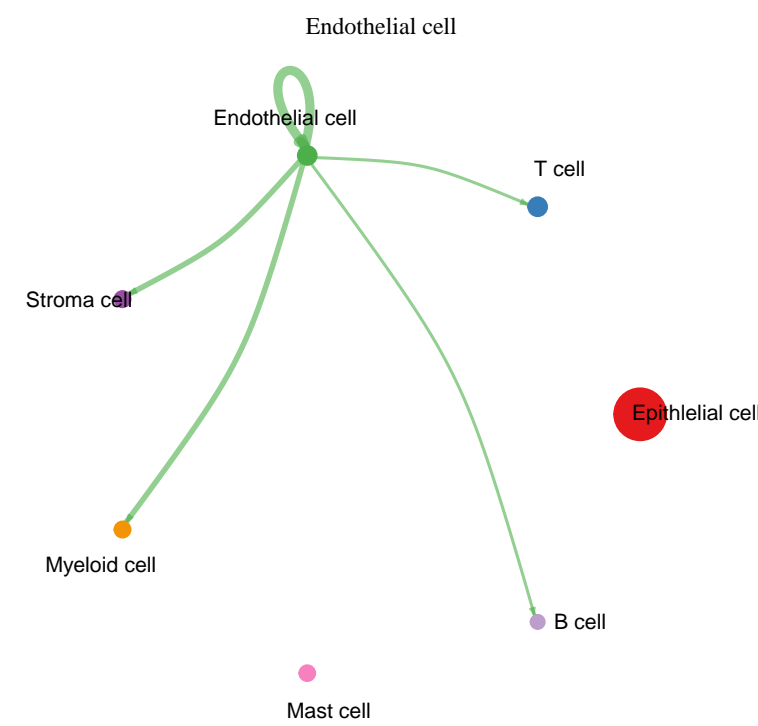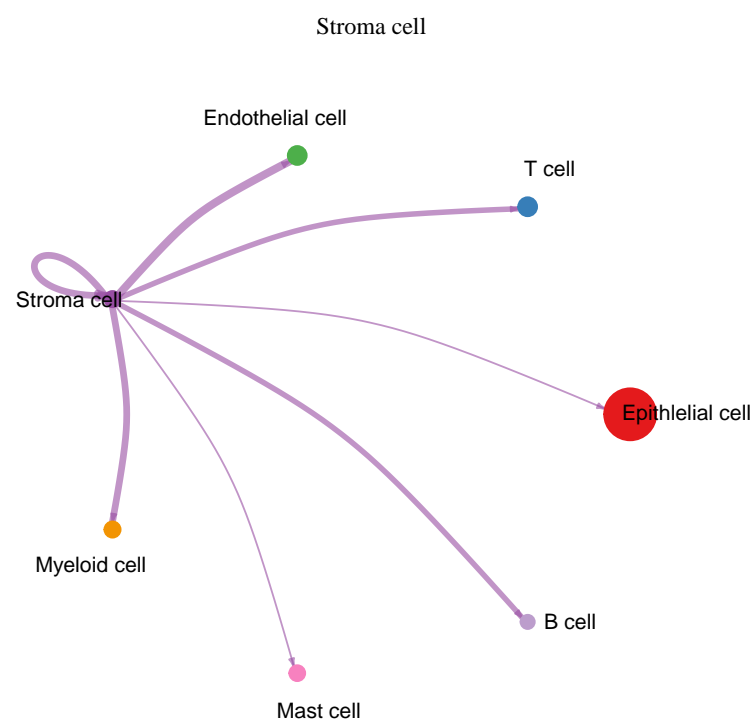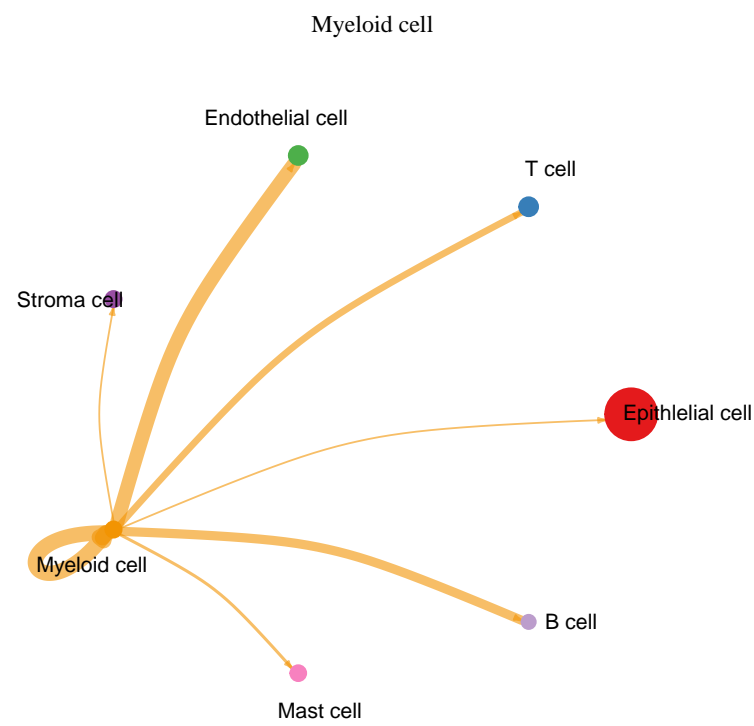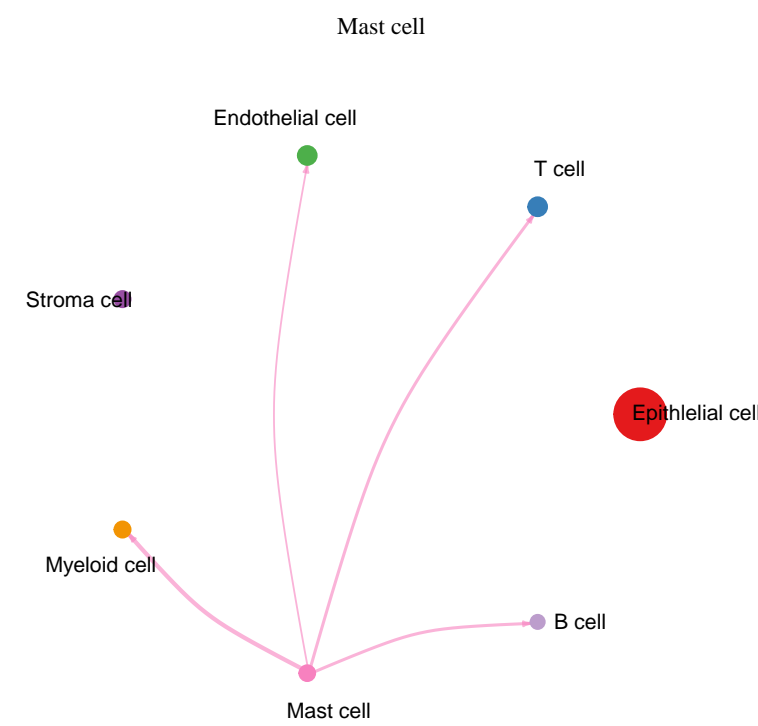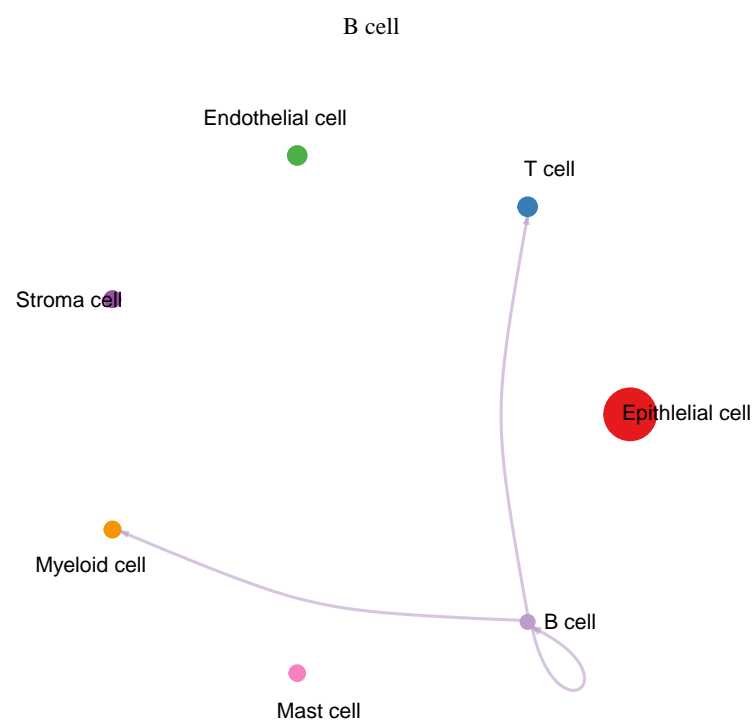

Supplement: Supplementary file 5 [file DataSheet5.zip › Supplementary Figure 5/Supplementary Figure 5A.pdf]

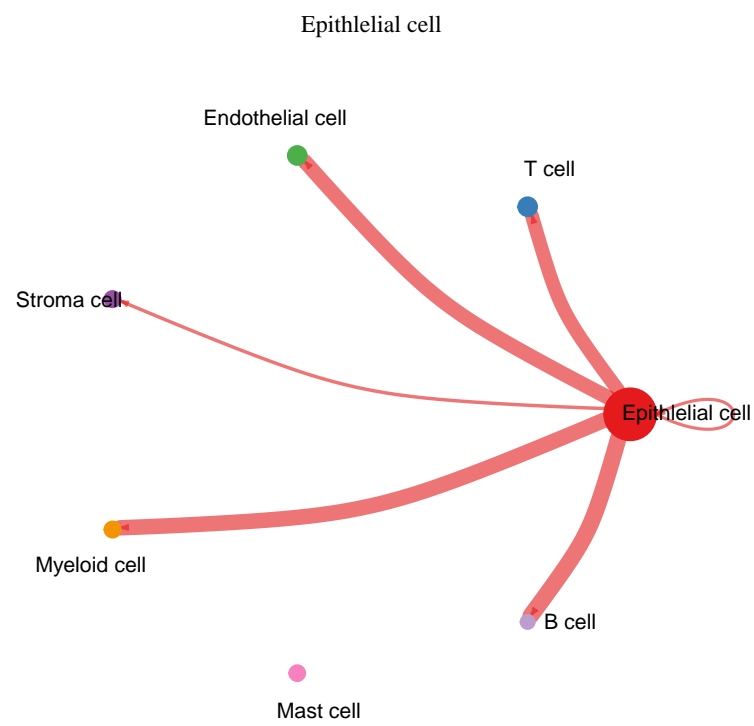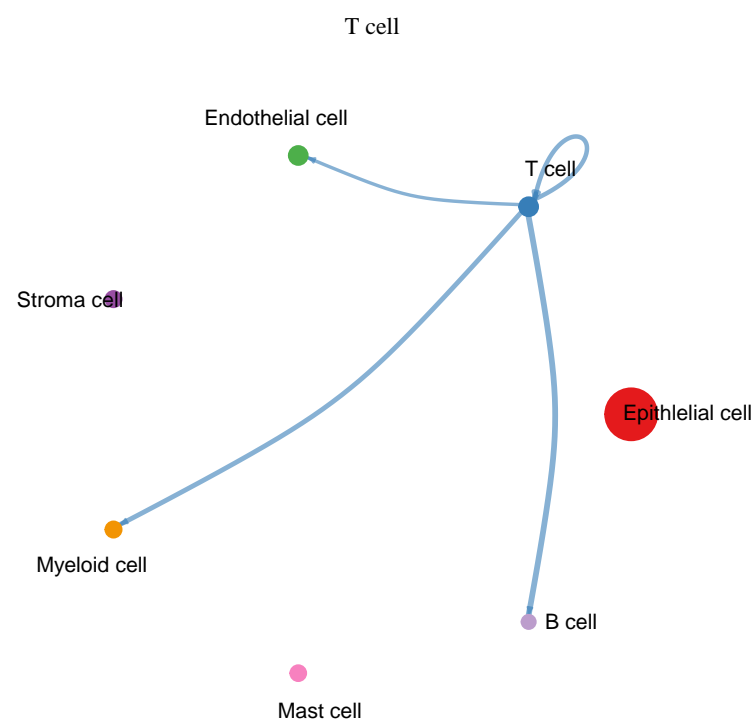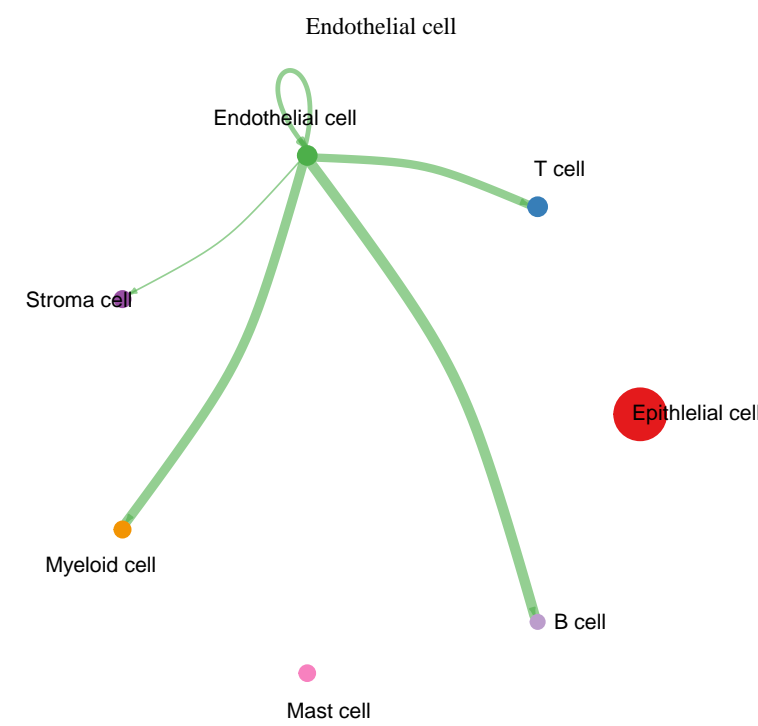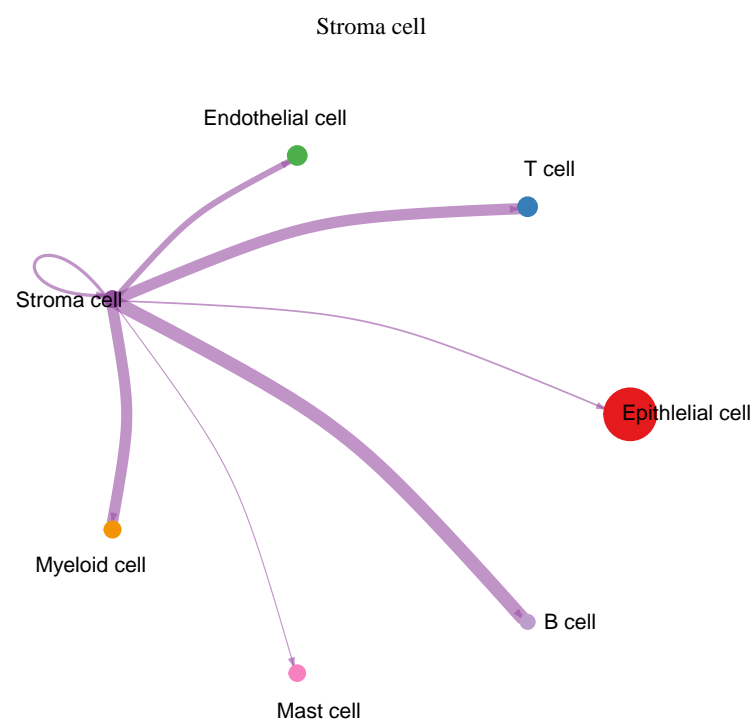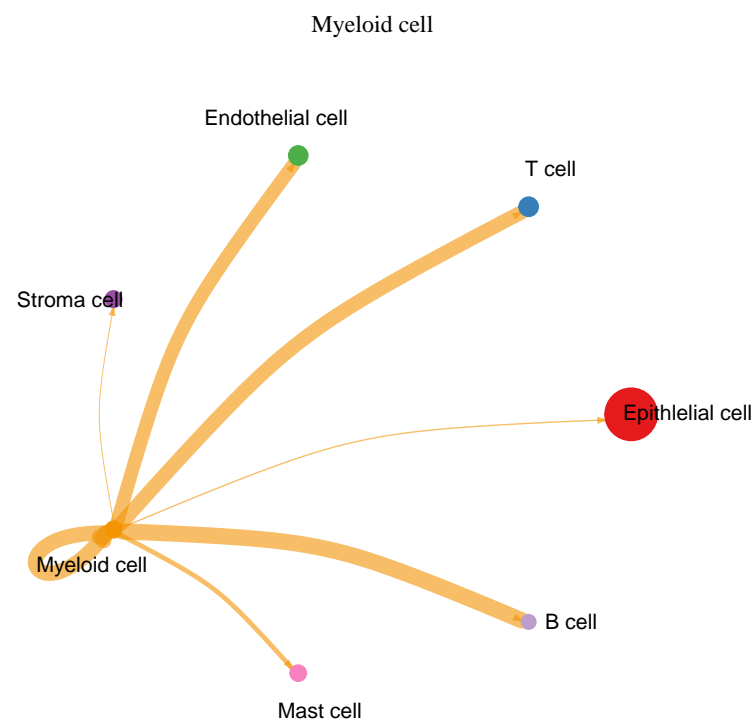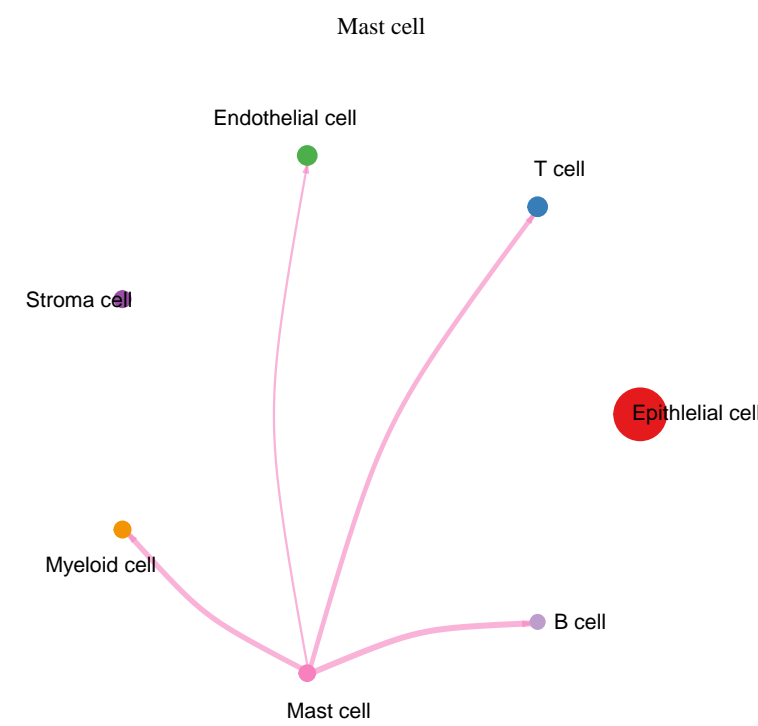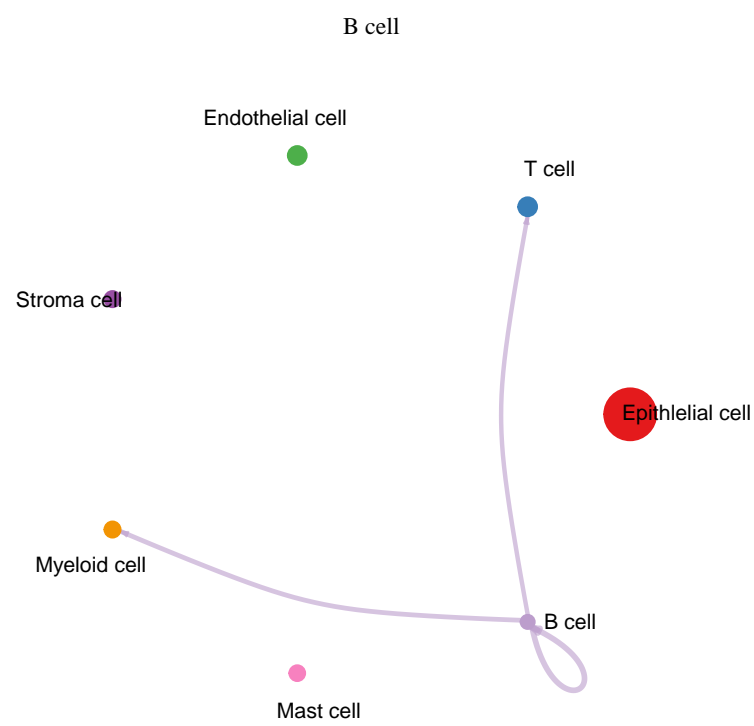

Supplement: Supplementary file 5 [file DataSheet5.zip › Supplementary Figure 5/Supplementary Figure 5B.pdf]

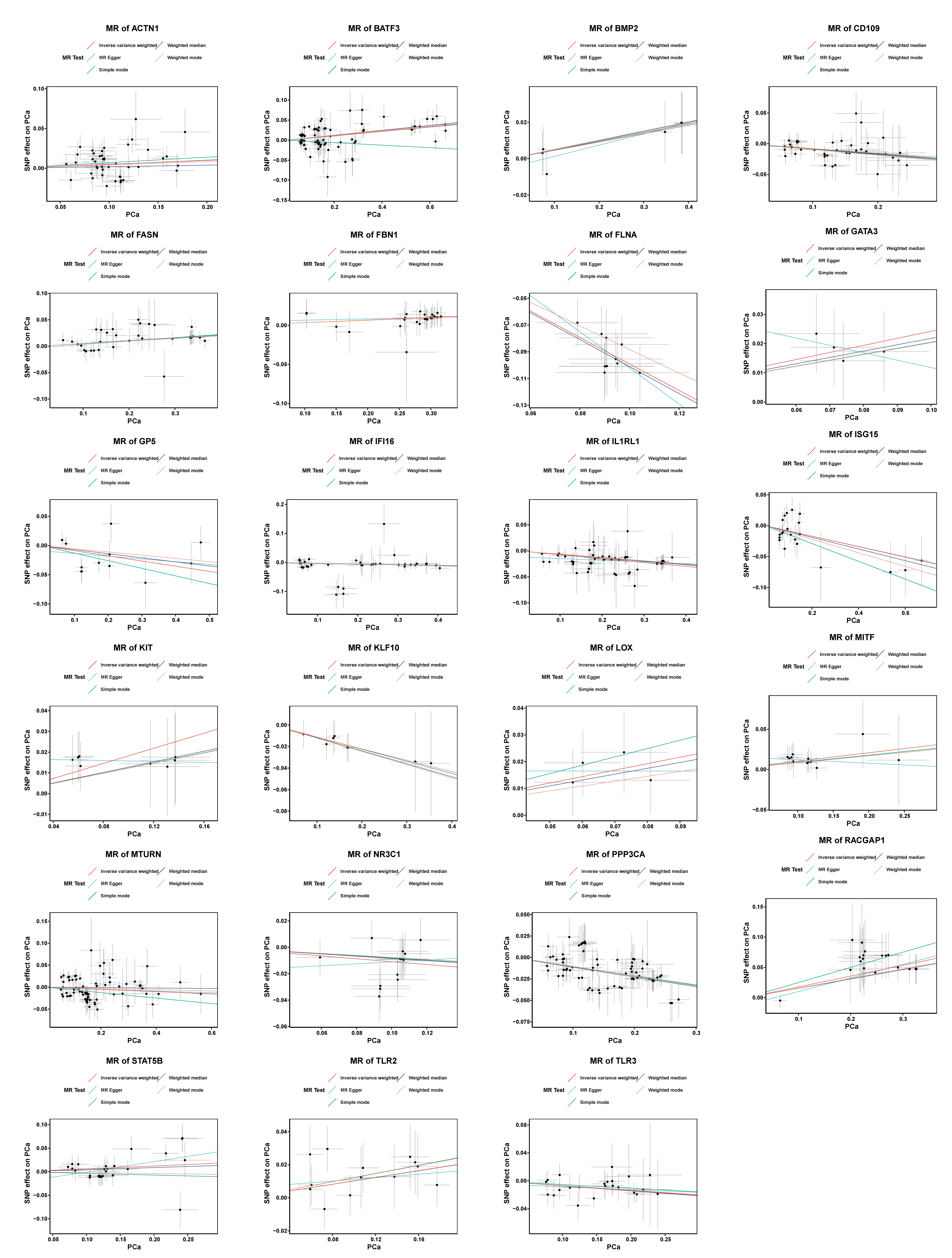

Supplement: Supplementary file 6 [file Image1.tif]

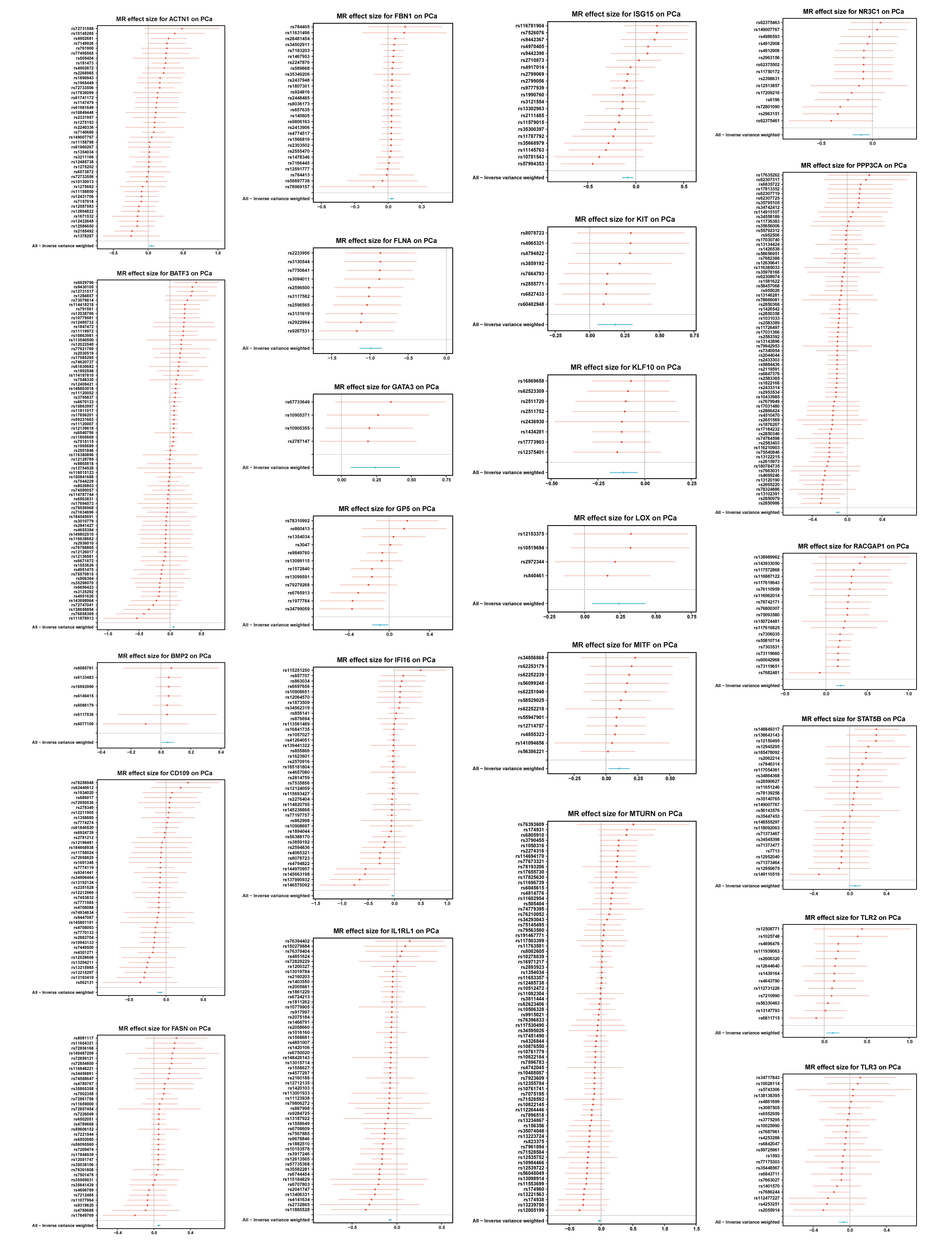

Supplement: Supplementary file 7 [file Image2.tif]

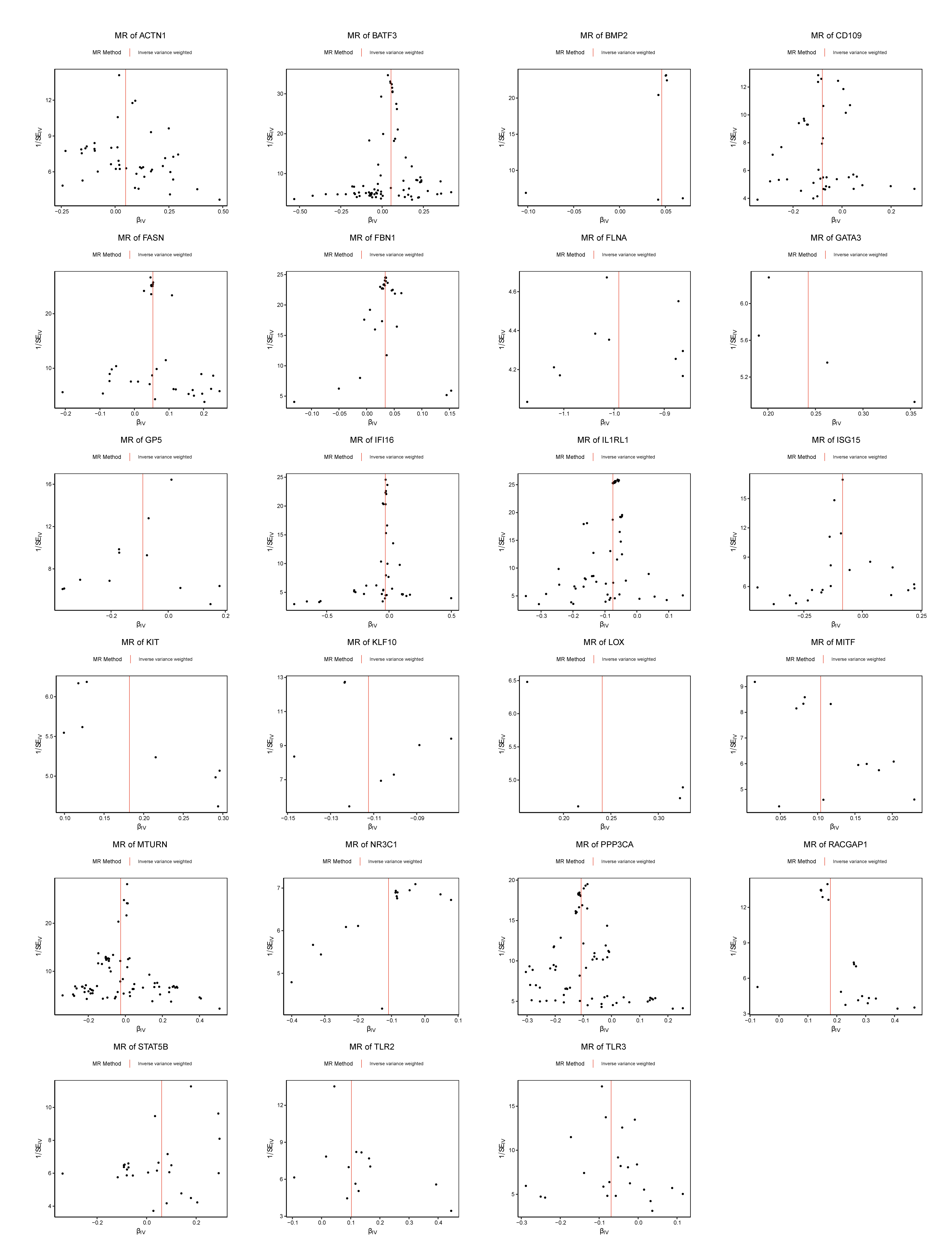

Supplement: Supplementary file 8 [file Image3.tif]

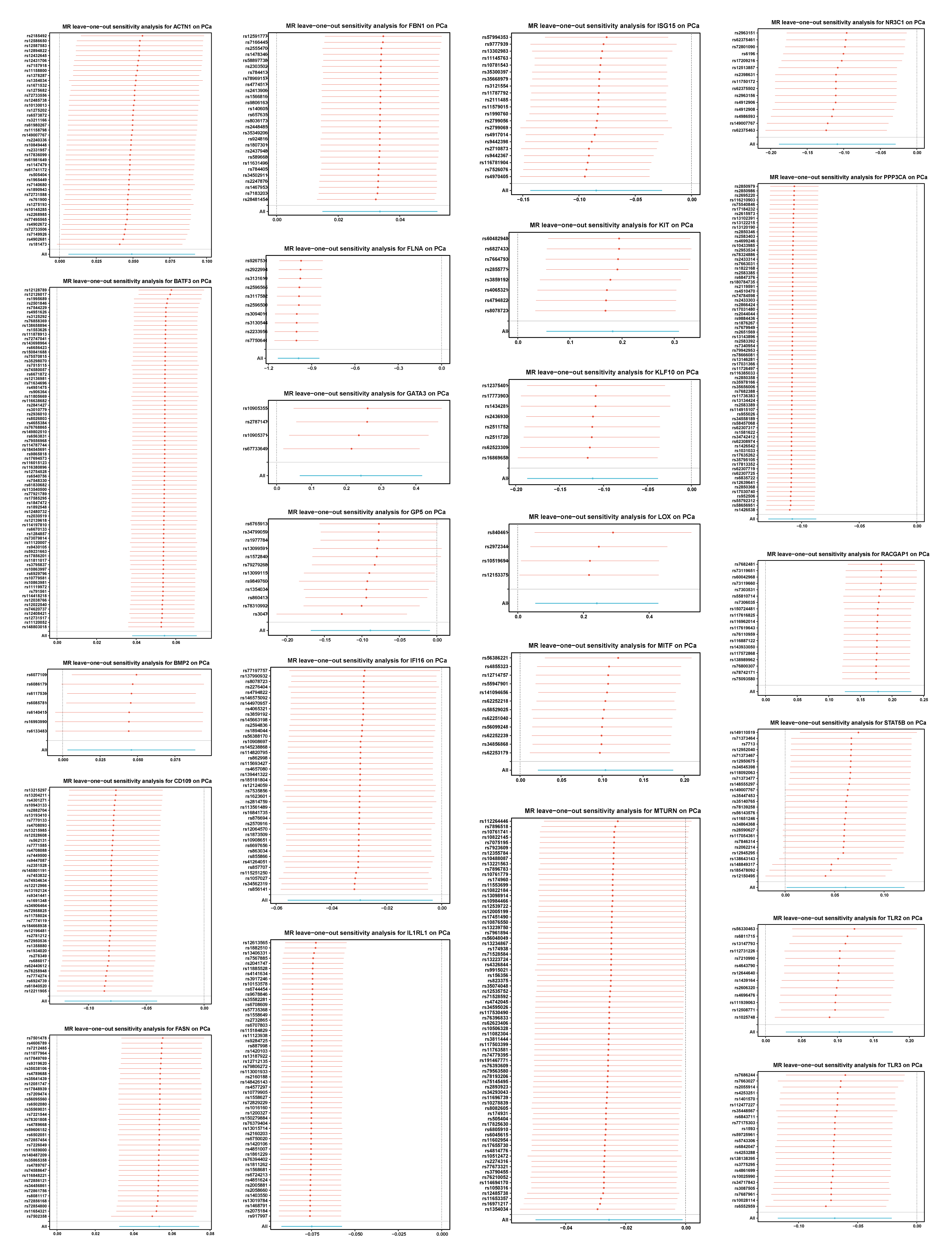

Supplement: Supplementary file 9 [file Image4.tif]

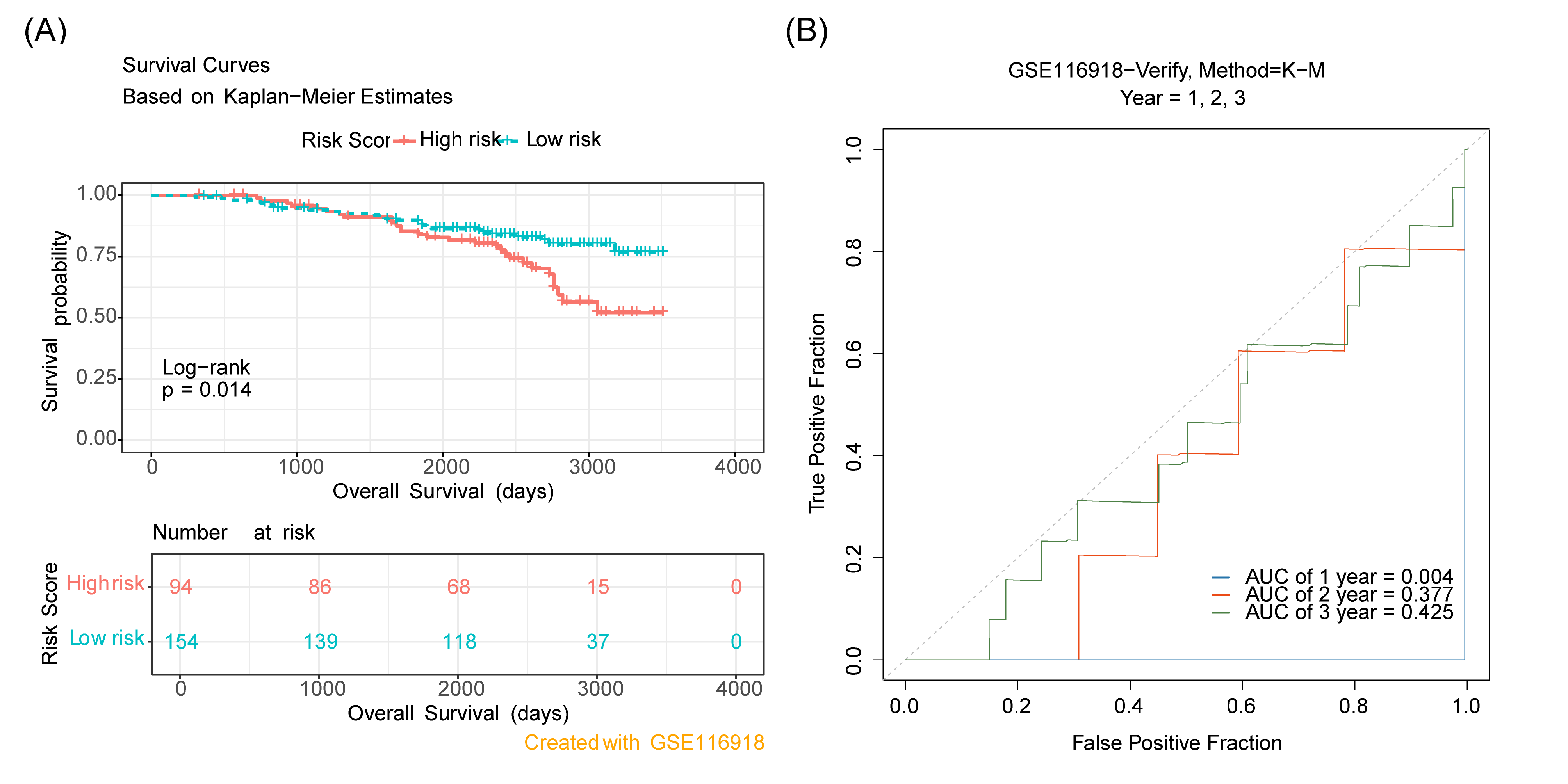

Supplement: Supplementary file 10 [file Image5.tif]

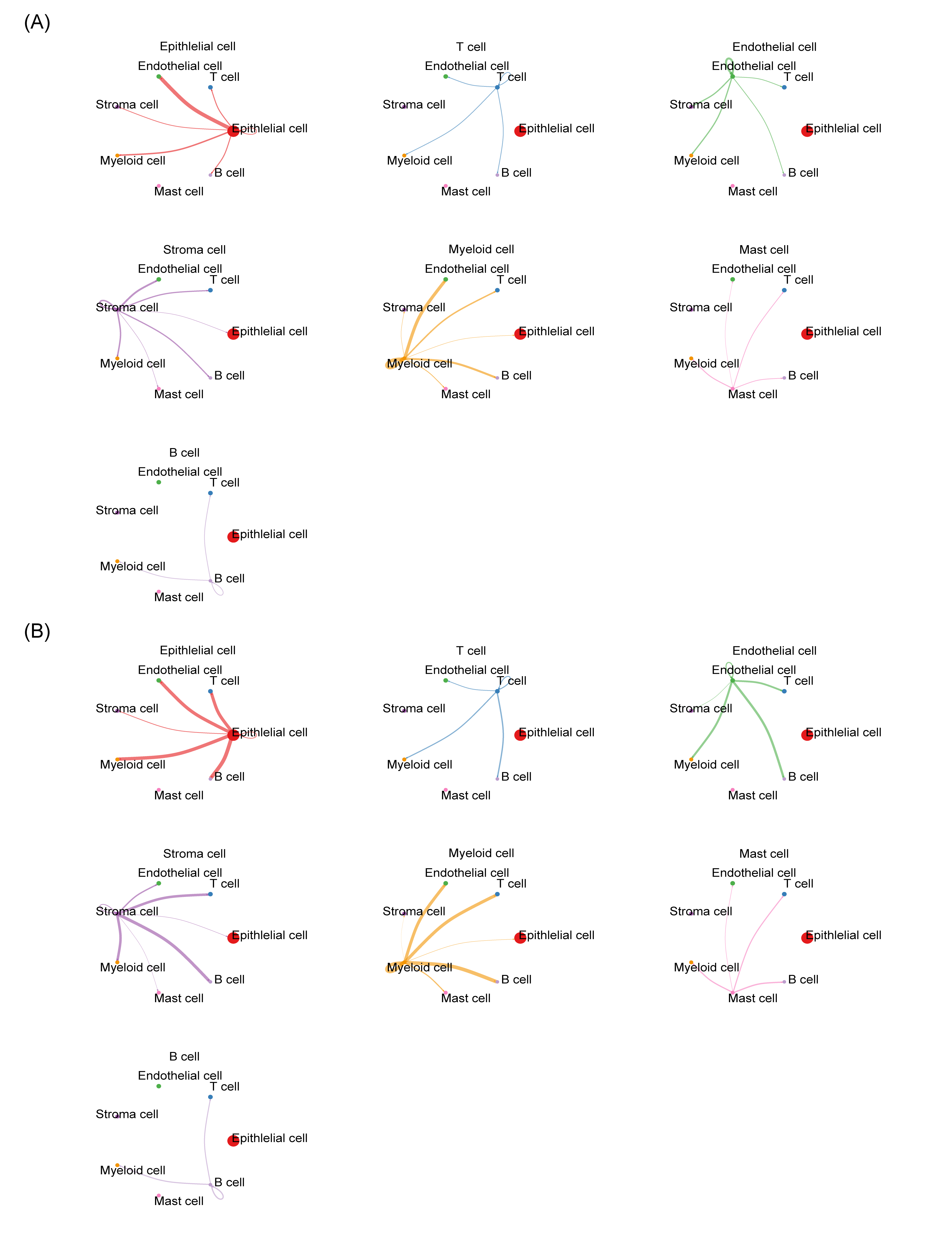

Supplement: Supplementary file 11 [file Image6.tif]

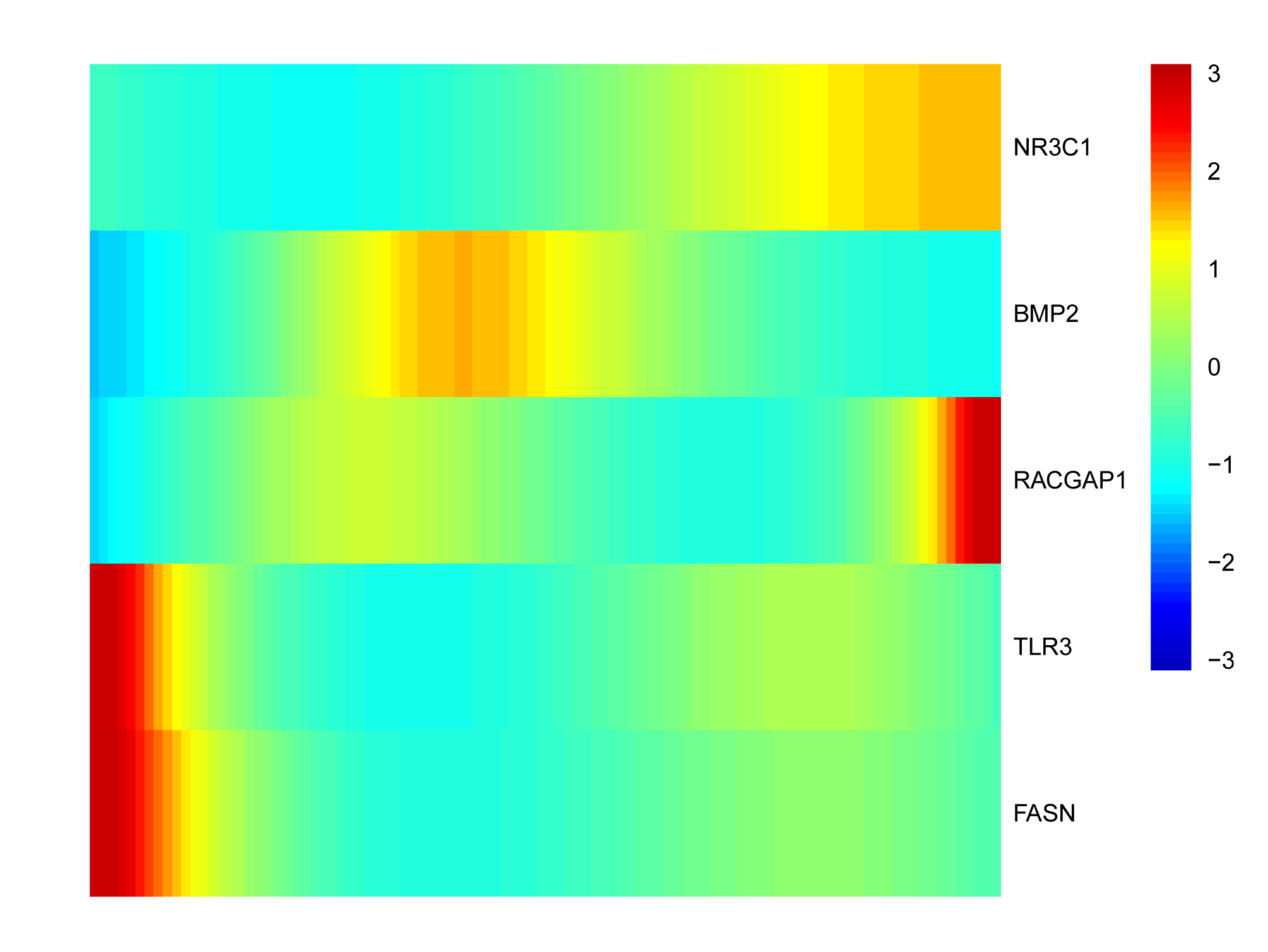

Supplement: Supplementary file 12 [file Image7.tif]
